# Ultrabright two-photon excitable red-emissive fluorogenic probes for fast and wash-free bioorthogonal labelling in live cells†

**DOI:** 10.1039/d3sc01754k

**Published:** 2023-07-04

**Authors:** Marie Auvray, Delphine Naud-Martin, Gaëlle Fontaine, Frédéric Bolze, Gilles Clavier, Florence Mahuteau-Betzer

**Affiliations:** a CNRS UMR9187, Inserm U1196, Chemistry and Modeling for the Biology of Cancer Institut Curie, Université PSL 91400 Orsay France marie.auvray1@gmail.com florence.mahuteau@curie.fr; b CNRS UMR9187, Inserm U1196, Chemistry and Modeling for the Biology of Cancer, Université Paris-Saclay 91400 Orsay France; c UMR7199, Faculté de Pharmacie 67401 Illkirch-Graffenstaden France; d PPSM ENS, Paris-Saclay 91190 Gif-sur-Yvette France

## Abstract

Fluorogenic bioorthogonal reactions are promising tools for tracking small molecules or biomolecules in living organisms. Two-photon excitation, by shifting absorption towards the red, significantly increases the signal-to-noise ratio and decreases photodamage, while allowing imaging about 10 times deeper than with a confocal microscope. However, efficient two-photon excitable fluorogenic probes are currently lacking. We report here the design and synthesis of fluorogenic probes based on a two-photon excitable fluorophore and a tetrazine quenching moiety. These probes react with bicyclo[6.1.0]no-4-yn-9ylmethanol (BCN) with a good to impressive kinetic rate constant (up to 1.1 × 10^3^ M^−1^ s^−1^) and emit in the red window with moderate to high turn-on ratios. TDDFT allowed the rationalization of both the kinetic and fluorogenic performance of the different probes. The best candidate displays a 13.8-fold turn-on measured by quantifying fluorescence intensities in live cells under one-photon excitation, whereas a value of 3 is sufficient for high contrast live-cell imaging. In addition, live-cell imaging under two-photon excitation confirmed that there was no need for washing to monitor the reaction between BCN and this probe since an 8.0-fold turn-on was measured under two-photon excitation. Finally, the high two-photon brightness of the clicked adduct (>300 GM) allows the use of a weak laser power compatible with *in vivo* imaging.

## Abstract 2

Our best fluorogenic probe enables live-cell imaging with high-contrast and no need for washing steps.

## Graphical abstract

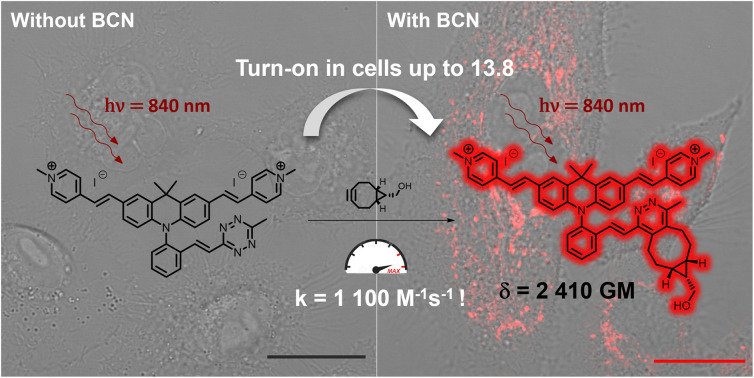

## Introduction

**Fig. 1:**
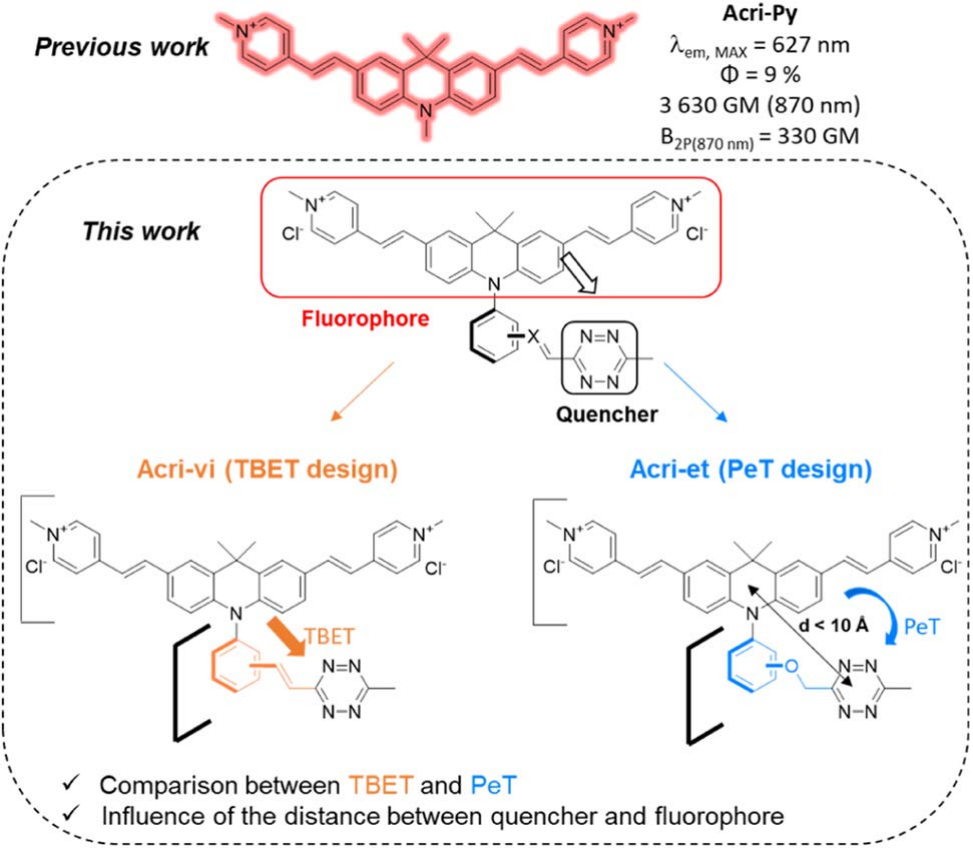
Design of the Acri-vi and Acri-et fluorogenic probes.

Fluorescence is a technique of choice among the various imaging methods available, due to its sensitivity and minimally invasive nature.^1–3^ Ideally, dyes should absorb and emit at wavelengths compatible with live-cell imaging (red to near IR), with high levels of brightness and photostability. However, the use of fluorescent probes is subject to several limitations, such as the intrinsic fluorescence of the tissue (autofluorescence) or a limited depth of penetration linked to the low transparency of tissues in the wavelength ranges classically used in fluorescence microscopy. Two-photon excitation fluorescence microscopy is a good alternative for overcoming these drawbacks, as it involves absorption in the near infrared (NIR) range, which minimizes the absorption and diffusion of light by biological samples. Moreover, excitation is confined to a small volume (around 1 fL) at the focal point, considerably decreasing the risk of photodamages.^4^ In recent decades, considerable effort has been devoted to developing two-photon excitable probes.^5^ In addition to photophysical criteria, fluorophores should ideally satisfy physico-chemical and biological criteria concerning solubility in water and cell permeability, for example. Probes meeting all these requirements can be conjugated to small molecules or biomolecules for labelling purposes. However, the fluorophore moiety may affect the distribution of the labelled molecule within the cell. The development of bioorthogonal reactions has been a major breakthrough, enabling the conjugation of drugs with fluorescent tags after target recognition.^6^ Nevertheless, distinguishing the fluorescence of the free fluorophore from that of the conjugated fluorophore can still be an issue, particularly because the reporter dye is often present in excess relative to the drug studied, to accelerate the kinetics of the bioorthogonal reaction. After incubation, it is therefore often necessary to fix the cells and wash them to remove the excess dye, which limits the usefulness of this technique for monitoring living cells. Conversely, dye washing in living cells can be time-consuming (up to several hours).^7,8^ This procedure can also result in the removal of the probe, if it is not sufficiently well bound to its target or organelle. Fluorogenic bioorthogonal probes have been developed to overcome these problems.^9^ The fluorescence of these probes is initially very weak or non-existent and is restored by the reaction. This so-called “turn-on” property enables unambiguous cell labelling and provides an excellent signal-to-noise ratio. The design of such probes is based on the profluorophore-quenching effect of the click handle. Azide,^10–12^ sydnone^13–15^ and tetrazine^16–22^ have been used to quench profluorophores *via* different photoinduced energy or electron transfer mechanisms. Tetrazines have been widely used, as they undergo rapid inverse electron demand Diels–Alder reactions (iEDDA) with strained dienophiles, with kinetic constants between 1 and 10^6^ M^−1^ s^−1^.^23^ Tetrazine fluorogenic probes with high turn-on ratios have been developed, some of which have been used for super-resolution imaging.^24–27^ Impressive fluorescence enhancements, up to several thousand-fold, have been reported for some fluorogenic probes,^17–19,28^ but much lower turn-on ratios have been reported for red-emissive probes.^29,30^ Before the study by Mao *et al.*,^30^ the best turn-on ratios reported for fluorogenic red-emitting bioorthogonal probes remained at about a 50-fold fluorescence enhancement.^26,29,31–35^ Finally, only a few recent examples of two-photon excitable fluorogenic probes have been reported.^36,37^ None of these probes has both a high turn-on ratio and a high two-photon absorption capacity (reported cross-sections < 65 GM) for red-emissive probes. We decided to fill this gap by designing a series of fluorogenic probes based on an acridan scaffold. Our recently reported optimized probe, Acri-Py, meets all the criteria for use in live-cell imaging, including high two-photon brightness and red emission (Fig. 1).^38^ Finally, it also has a high cross-section relative to its molecular weight (*δ*/*M* = 7.9) —an essential feature of biocompatible fluorophores. We then envisaged different tetrazine attachments to this scaffold to compare different quenching mechanisms (Fig. 1). We changed the nature of the spacer (conjugated Acri-vi series *versus* non-conjugated Acri-et series). We also modified the distance between the fluorophore and its quencher by preparing *ortho*-, *meta*- and *para*-derivatives for both designs. We found that the *ortho*-derivatives had higher fluorogenic efficiencies than their regioisomers. The reaction rate constants of iEDDA reactions were up to 10 times higher than those reported for this type of reaction with BCN. *Ortho* derivatives had impressively high kinetic constants that were explained by TDDFT calculations. We then demonstrated the potential of our best fluorogenic probe, Acri-*o*vi, for selective protein labelling experiments and for wash-free two-photon imaging in live A549 cells.

## Results and discussion

### Design and synthesis of fluorogenic probes

We based our design on the recently published Acri-Py (Fig. 1).^38^ The nitrogen atom of the acridan is an ideal anchor point for tetrazine attachment. We focused on the through-bond energy transfer (TBET) and photoinduced electron transfer (PeT) quenching mechanisms as we wanted to compare these designs on our two-photon excitable fluorophore. We therefore prepared two series of fluorogenic probes differing only in the link between the dye and the tetrazine. The distance between the two chromophores is known to affect PeT efficiency and should not be greater than 10 Å. TBET requires conjugation between the two chromophores. The unsaturated linker must also prevent the chromophores from being coplanar in the ground state, thereby enabling them to be electronically independent. We thus used a styryl linker (Acri-vi series, Fig. 1). For the PeT design, we replaced the styryl linker with an oxymethylphenyl linker, which should prevent TBET without affecting the distance between the fluorophore and the quencher. The Acri-vi and Acri-et series were therefore considered good candidates for a comparative study of these two quenching mechanisms. Finally, we investigated the effect of the distance between the two chromophores, by preparing *ortho*, *meta* and *para* derivatives. Such a study has been done on the rhodamine scaffold for the PeT design.^26^ In contrast, no systematic study has been reported on TBET design, as no *ortho* derivative with a styryl spacer has ever been described, even on well-studied rhodamine derivatives.^18^ However, some groups reported that *meta* substituted isomers exhibit higher turn-on ratios than *para* derivatives.^28,29,39^ Using TDDFT, we were also able to predict this distance on optimized structures (ESI, Fig. S1†). The *ortho* derivative was expected to be the most efficient for PeT, as it has the shortest distance between the two chromophores (7.6 Å), but all three regioisomers meet the distance criterion (<10 Å). The tetrazine is perpendicular to the acridine core, which is essential for TBET (ESI, Fig. S2†).

**Scheme 1:**
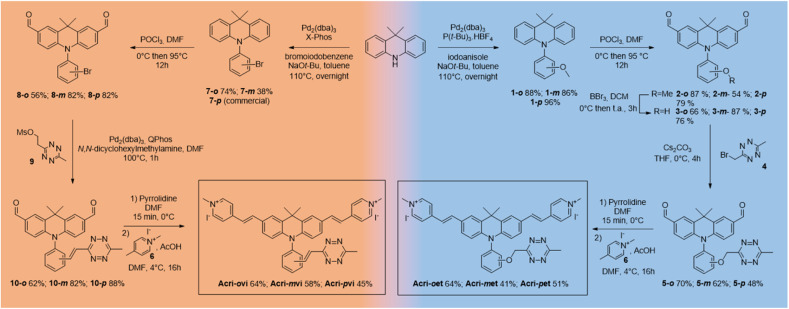
Synthesis of Acri-vi and Acri-et probes.

The syntheses of both series began from the same starting point, the commercially available 9,9-dimethylacridan, and followed a similar route. Buchwald–Hartwig amination coupling was used to generate *N*-methoxyphenyl-9-dimethylacridan 1 or *N*-bromophenyl-9,9-dimethylacridan 7 in moderate-to-high yields. A Vilsmeier–Haack reaction on acridans 1 and 7 yielded dialdehydes 2 and 8, respectively, in good yields. We then applied the Heck coupling conditions developed by Devaraj *et al.* to generate vinyltetrazine derivatives 10.^18^ This step required the use of the non-volatile 2-(1,2,4,5-tetrazine-3-yl)ethyl methanesulfonate 9 and gave good to excellent yields. We obtained the corresponding dialdehydes of the Acri-et series, by performing Williamson ether synthesis with bromomethyl tetrazine 4 as previously described.^26^ The final compounds, Acri-vi and Acri-et, were obtained through a Knoevenagel reaction. Conventional conditions for the Knoevenagel reaction were adapted to avoid an excess of nucleophilic base, given the sensitivity of tetrazine to nucleophiles. Indeed, mixing dialdehyde 10 and compound 6 in the presence of pyrrolidine under classical Knoevenagel conditions led to tetrazine degradation. Stoichiometric addition of pyrrolidine at 0 °C led to the formation of iminium salts without degradation. However, the addition of the *N*-methyl-4-methyl pyridinium salt 6 to the iminium salts releases pyrrolidine in the reaction medium. We then simultaneously added the *N*-methyl-4-methyl pyridinium salt 6 and various acids in order to trap the released pyrrolidine (ESI Table S1†). Finally, acetic acid was the best candidate. After simultaneous addition of acetic acid and compound 6, the reaction mixture was stirred at 4 °C for 16 h. This protocol prevents the degradation of the tetrazine moiety. This reaction led to the formation of the Knoevenagel products in moderate to good yields. Finally, we obtained the desired fluorogenic probes in a four- or five-step sequence, with overall yields between 10 and 22% (Scheme 1).

We then studied iEDDA adducts with (bicyclo[6.1.0]no-4-yn-9yl)methanol (BCN). BCN reacts with tetrazine more slowly than *trans*-cyclooctene (TCO), but it is a more pertinent partner, as its iEDDA cycloadduct with tetrazine (pyridazine) is no longer able to quench fluorescence whereas the TCO-cycloadduct (dihydropyridazine) could display a residual fluorescence quenching effect.^35^ We then performed the click reaction between BCN and the probes and checked the purity of the products by HPLC (ESI†).

### Turn-on ratio and kinetics

The one-photon absorption and emission properties of the unquenched derivative Acri-Py, the fluorogenic probes and their click adducts with BCN were characterized in aqueous buffer (ESI Table S2†). All displayed absorption at about 485 nm, with high molar absorption coefficients (up to 61 000 M^−1^ cm^−1^), and emission in the red window, with a maximal emission wavelength between 638 and 652 nm. All dyes are very weakly fluorescent in native forms.^38^ As they became fluorescent upon immobilization, their photophysical properties and those of their click adducts were determined in a buffer containing Bovine Serum Albumin (BSA) to mimic a constrained environment. We first showed that 100 equivalents of BSA were sufficient to reach the fluorescence plateau for each fluorogenic probe and its clicked product (ESI Fig. S3†). By performing this titration experiment, we were able to ensure that the turn-on factors measured with 100 equivalents of BSA were independent of the affinity of the molecules for BSA. The photophysical properties of the probes and their click adducts in BSA are summarized in ESI Table S3.† The absorption properties of the probes and their click adducts with BSA were similar to those in buffer alone. There was a slight red-shift in the absorption wavelength (495 nm *versus* 485 nm). Conversely, maximal emission wavelengths were blue-shifted between 590 and 605 nm. However, the spectra obtained were broad (from 550 nm to 750 nm), allowing detection in the far-red window. The fluorogenic probes had low fluorescence quantum yields in BSA, confirming the efficiency of fluorescence quenching. Moreover, the click adducts had fluorescence quantum yields of about 0.15, similar to that of Acri-Py, demonstrating the restoration of fluorescence upon the fluorogenic reaction (ESI, Table S3†).

**Fig. 2:**
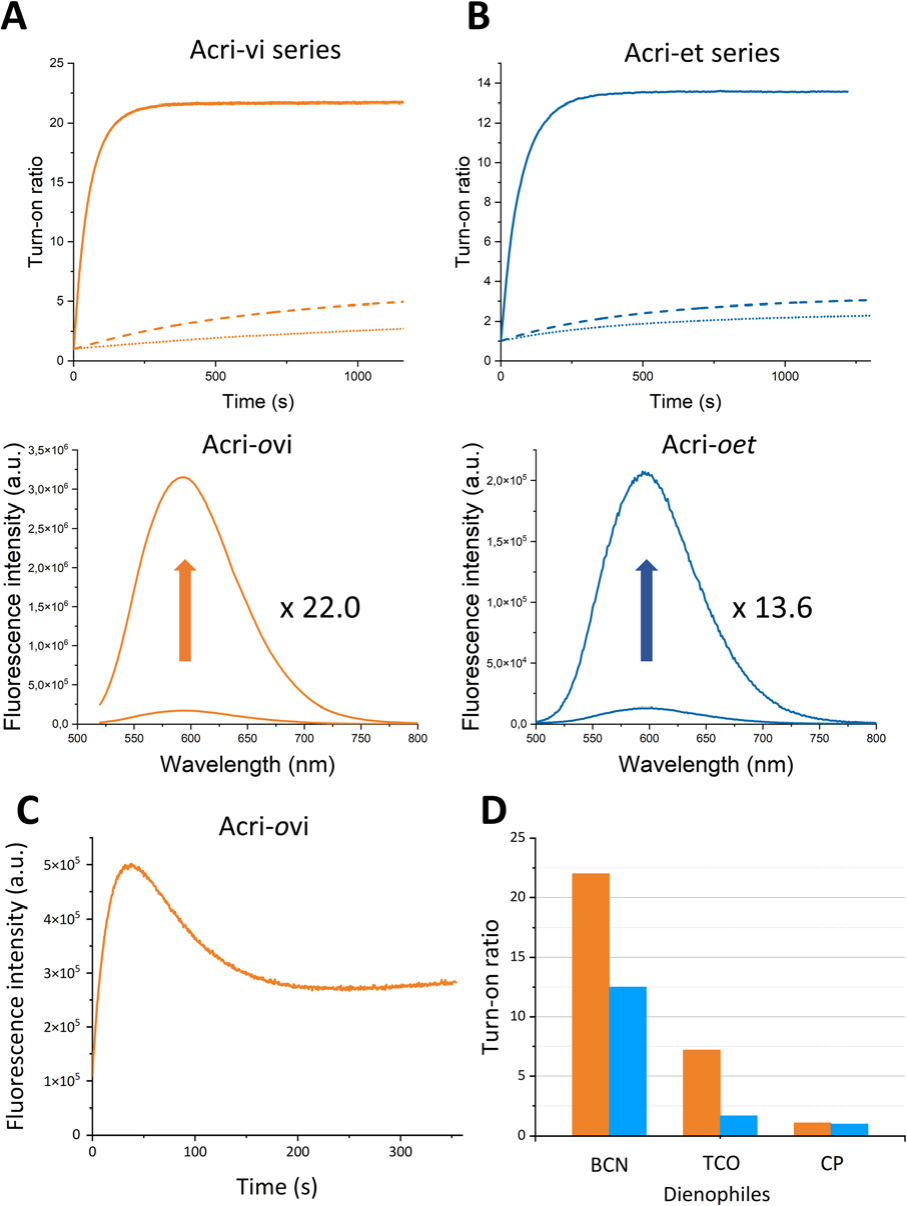
Fluorescence enhancement of (A) Acri-vi and (B) Acri-et fluorogenic dyes (2 μM) upon the addition of BCN (20 μM) in 10 mM sodium cacodylate buffer (100 mM NaCl and pH 7.4) containing BSA (200 μM). *Ortho*, *meta* and *para* derivatives are represented by solid, dashed and dotted lines, respectively. (C) Fluorescence enhancement of (C) Acri-*o*vi (0.5 μM) upon the addition of TCO (50 μM) and of (D) fluorogenic dyes Acri-*o*vi (in orange) and Acri-*o*et (in blue) (2 μM) upon the addition of BCN, TCO or CP (20 μM), in 10 mM sodium cacodylate buffer (100 mM NaCl and pH 7.4) containing BSA (200 μM).

In order to accurately determine turn-on ratios, we monitored fluorescence during the reaction between the probe (2 μM) and 10 equivalents of BCN, with 100 equivalents of BSA in the buffer (Fig. 2).

The turn-on ratios obtained were consistent with the ratios of fluorescence quantum yields, with the same ranking and similar values (Table 1). Turn-on ratios were systematically higher for the Acri-vi series than for the Acri-et series. The TBET strategy was more efficient than the PeT strategy under these conditions. Moreover, the position to which the tetrazine is attached appears to have a major effect on quenching efficiency, for both PeT and TBET designs: turn-on (*ortho*) ≫ turn-on (*meta*) > turn-on (*para*) for both series (Table 1). This ranking was predicted for the PeT design, as the distance between the tetrazine and the fluorophore moiety increases in the same order (Fig. S1†). However, it was more unexpected for the TBET design. Several previous studies have reported that *meta*-connected tetrazine gave a higher turn-on ratio than *para* isomers in TBET designs,^28,29,39^ but the reasons for this phenomenon have not been determined. A unique feature of our study is the comparison of *ortho*, *meta* and *para* substitutions on the phenyl spacer in the vinyl and ether series. Both *ortho* isomers displayed a high fluorescence enhancement for red-emitting probes—22.0 for Acri-*o*vi and 13.6 for Acri-*o*et—with the same ranking for kinetics. Under standard conditions for cellular imaging (micromolar concentration), both *ortho* derivatives reacted within 10 minutes, while the conversion of the *meta* and *para* derivatives remained incomplete after 30 minutes. Kinetic constant measurements confirmed this trend (Table 1). The rate constants for reactions between BCN and all probes were compatible with bioorthogonal reactions in live cells (*k* > 29 M^−1^ s^−1^, see Table 1), but the rate constant for Acri-*o*vi was particularly impressive (*k* = 1100 M^−1^ s^−1^). Unexpectedly, it was much higher than that for the other probes: 18 times greater than that of its *meta* analog and 38 times higher than that of its *para* analog (ESI Fig. S4†). Similarly, the reaction of Acri-*o*et with BCN was faster than that of its *meta* and *para* analogs (5 times faster). This *in vitro* study shows that Acri-*o*vi is clearly the most efficient fluorogenic probe of these series, in terms of both turn-on ratio and kinetic constant.

**Table 1:** Turn-on ratio^a^ and rate constant^b^ for the reaction of BCN and the fluorogenic probes Acri-et and Acri-vi

Probes	*Φ*_F_ (*Φ*_F–Click_)	*Φ*_F–Click/_ *Φ*_F_	Turn-on^a^	Rate constant^b^ [M^−1^ s^−1^]
Acri-*o*vi	0.007 (0.16)	22.8	22.0	1100
Acri-*m*vi	0.025 (0.19)	7.5	7.2	61
Acri-*p*vi	0.028 (0.14)	5.0	5.0	29
Acri-*o*et	0.01 (0.13)	12.5	13.6	514
Acri-*m*et	0.035 (0.15)	4.2	3.8	91
Acri-*p*et	0.053 (0.15)	2.9	2.7	98

a Turn-on experiments were performed with 10 equivalents of BCN in the presence of 100 equivalents of BSA in 10 mM sodium cacodylate buffer (100 mM NaCl and pH 7.4).

b See the ESI.

To confirm that the conditions (BSA in buffer) can reflect what happens in live cells, turn-on measurement of the reaction between Acri-*o*vi and BCN in cell lysate was performed. A 12-fold enhancement was observed for this fluorogenic reaction, demonstrating that measurements in BSA provide a relevant *in vitro* model (see ESI Fig. S5†).

We then wanted to investigate the turn-on of the probe with various dienophiles. We chose *trans*-cyclooctene (TCO), which is known to react fast and 2-methyl-*N*-(isopropyl)cycloprop-2-ene-1-carboxamide (CP), which, conversely, is a weak reactive partner. However, this small strained dienophile could be of interest as it can label small molecules or biomolecules without steric impact compared with large strained cycloalkynes.^40,41^ We selected Acri-*o*et and Acri-*o*vi as the most fluorogenic probes. We then measured their fluorescence at 2 μM in BSA-containing buffer, and their fluorescence two hours after addition of the dienophile at 20 μM (Fig. 2C). Two hours is a reasonable time beyond which the reaction is difficult to apply in a biological environment. As already reported in the literature,^26,28,35^ we observed that the increase in fluorescence is strongly dienophile-dependent. TCO-cycloadducts were less fluorescent than BCN-cycloadducts for both probes. While studying kinetics between Acri-*o*vi and TCO, we observed a rapid conversion to a fluorescent species (*k* = 1052 M^−1^ s^−1^, see ESI Fig. S6† and 2C) followed first by a decrease in fluorescence, which was followed by a slow restoration of fluorescence. This phenomenon has already been mentioned by others^28,35^ and has been studied in detail recently.^42^ We can therefore assume that the fluorescent 4,5-dihydropyridazine is formed rapidly by iEDDA and tautomerizes to 1,4-dihydropyridazine, which acts as a fluorescence quencher. The subsequent increase in fluorescence can be attributed to the fluorescent pyrazine resulting from the slow oxidation of 1,4-dihydropyridazine. TCO is therefore not a good candidate for these fluorogenic reactions, as it leads to different species. Furthermore, and unexpectedly, the rate constant of IEDDA between Acri-*o*vi and TCO was not higher than between Acri-*o*vi and BCN. Thus, BCN is undoubtedly the best partner for our probes and we have retained it for further biological applications. We also studied the fluorogenic reaction between Acri-*o*vi and Acri-*o*et with CP. In contrast, no or very weak fluorogenicity was detected. This is in accordance with the low kinetic constant determined for iEDDA between Acri-*o*vi and CP (*k* = 0.02 M^−1^ s^−1^, see ESI Fig. S6†) which does not allow the reaction to proceed within 2 h.

### Non-linear photophysical properties

The two-photon absorption spectra of the clicked adducts were recorded by the Two-Photon Excited Fluorescence (TPEF) method. TPEF requires highly concentrated samples (around 10^−4^ M). We were therefore unable to record the TP absorption spectra of these probes in buffer containing 100 equivalents of BSA as BSA is not soluble at 10^−2^ M in buffer. To circumvent this issue, measurements were performed in glycerol, a viscous solvent, which mimics a constrained environment (Table 2). Relative to their linear properties in aqueous buffer, we observed a slight red-shift in absorption and a blue-shift in emission. The probes emitted with a poor fluorescence quantum yield (between 1 and 5%), whereas the BCN-clicked adducts had higher fluorescence quantum yields (8 to 15%). Interestingly, the turn-on ratios of iEDDA reactions between BCN and probes follow the same order in glycerol and BSA: turn-on (*ortho*) ≫ turn-on (*meta*) > turn-on (*para*) and turn-on (Acri-*o*vi) = 15.3 > turn-on (Acri-*o*et) = 9.0, Acri-*o*vi remaining the most fluorogenic dye (Tables 1 and 2). Glycerol turned to be a good model for studying the fluorogenic reactions of these probes.

**Table 2:** Photophysical properties of fluorogenic probes Acri-et and Acri-vi and their clicked adducts with BCN in glycerol

Probe	*λ*_abs/_ *λ*_em_ [nm]	*Φ*_F_	*B*_1P_ = *Φ*_F_*ε* (turn-on_1P_)	*δ* [GM]	*B*_2P_ = *Φ*_F_*δ* (turn-on_2P_)
Acri-*o*vi	502/607	0.01	585	2800^a^	28
Clicked	505/612	0.15	8955 (15.3)	2410^a^	362 (12.9)
Acri-*m*vi	505/611	0.04	2100	2500^b^	100
Clicked	507/616	0.11	6006 (2.9)	2910^b^	320 (3.2)
Acri-*p*vi	504/613	0.05	2685	2700^a^	135
Clicked	506/616	0.11	6006 (2.2)	2800^a^	308 (2.4)
Acri-*o*et	509/610	0.015	873	3820^a^	57
Clicked	509/611	0.13	7878 (9.0)	3200^a^	416 (7.3)
Acri-*m*et	507/609	0.04	2248	3640^c^	145
Clicked	507/614	0.09	4563 (2.0)	2970^c^	267 (1.8)
Acri-*p*et	509/615	0.05	2355	3160^a^	158
Clicked	509/618	0.08	4024 (1.7)	3220^a^	257 (1.6)

a Excitation wavelength: 870 nm.

b Excitation wavelength: 880 nm.

c Excitation wavelength: 850 nm.

**Fig. 3:**
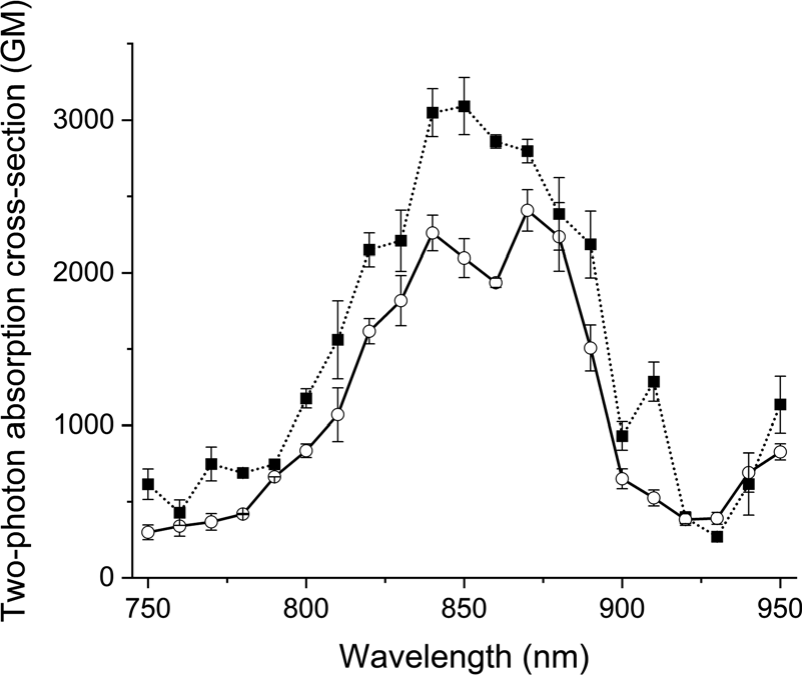
Two-photon excitation spectra of Acri-*o*vi (plain line) and Acri-*o*vi Clicked (dotted line) in glycerol.

The two-photon excitation spectra of the probes and their clicked adducts were recorded in glycerol at room temperature in duplicate. All compounds generated similar two-photon spectra (Fig. 3 and S7†), with two maxima: one at about 850 nm and an other at about 870 nm. All had high absorption cross-sections (>2400 GM), like Acri-Py.^38^ These values are particularly impressive for two-photon excitable red-emissive fluorogenic probes, as the highest values reported to date are below 65 GM.^37^ We also compared the performances of the dyes in terms of two-photon brightness (*δ* × *Φ*_F_), the most relevant property for imaging. The brightness values obtained (up to 416 GM) were more than 20 times higher than those reported in previous studies for red-emissive compounds.^36,37^ Depending on the respective absorption properties of the probes and their iEDDA products with BCN, we observed that 1P turn-on can be slightly higher or slightly lower than 2P-turn-on.

### Molecular modeling

**Fig. 4:**
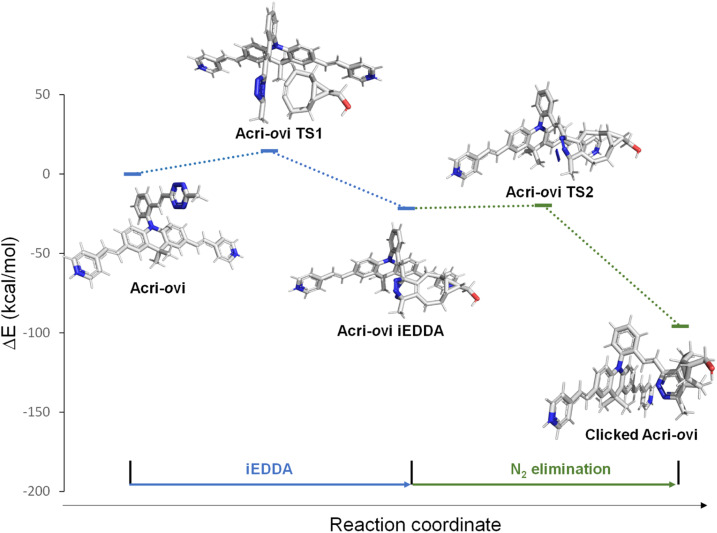
Schematic representations (energy *vs.* reaction coordinate) of the reaction between Acri-*o*vi and BCN.

The Acri-Xvi (X = *o*, *m* or *p*) series displayed striking variations in terms of rate constants of the click reaction and fluorescence turn-on ratios. We investigated this unexpected difference in behavior between the *ortho* derivative and the other two derivatives, by performing a series of quantum calculations. We first analyzed the reaction path fully with slightly simplified Acri-Xvi structures in which the methyl groups of the pyridinium were replaced by protons to facilitate the search for transition states. The click reaction proceeds in two steps: inverse electronic demand Diels–Alder (iEDDA) cycloaddition followed by a retro-Diels–Alder reaction (elimination of N_2_) to form the pyridazine ring. The overall reaction was strongly exergonic, releasing approximately 90 kcal mol^−1^ (Fig. 4, S8 and Table S5 ESI†). The first step was the rate-determining step, consistent with previous reports,^43^ whereas nitrogen elimination was almost barrier-less (calculated activation energy below 2 kcal mol^−1^). The calculated activation energy of the cycloaddition between BCN and Acri-*o*vi was 1.6–1.8 kcal mol^−1^ lower than that for Acri-*m*vi and Acri-*p*vi (Table 3). The conversion of these figures into reaction rates indicates a 15–20 times faster reaction for Acri-*o*vi, consistent with the experimentally observed variation (18- to 30-fold variation; Table 1). This difference can be explained by the lower energy of the LUMO of Acri-*o*vi than that of the other two derivatives, decreasing the energy of the early transition state (ESI Table S6†).

**Table 3:** Comparison of the relative calculated and experimental kinetic constants

Probe	AE (kcal mol^−1^)	*k*_relative calculated_	*k*_relative exp_
Acri-*o*vi	14.53	1	1
Acri-*m*vi	16.35	0.05	0.06
Acri-*p*vi	16.36	0.06	0.03

**Fig. 5:**
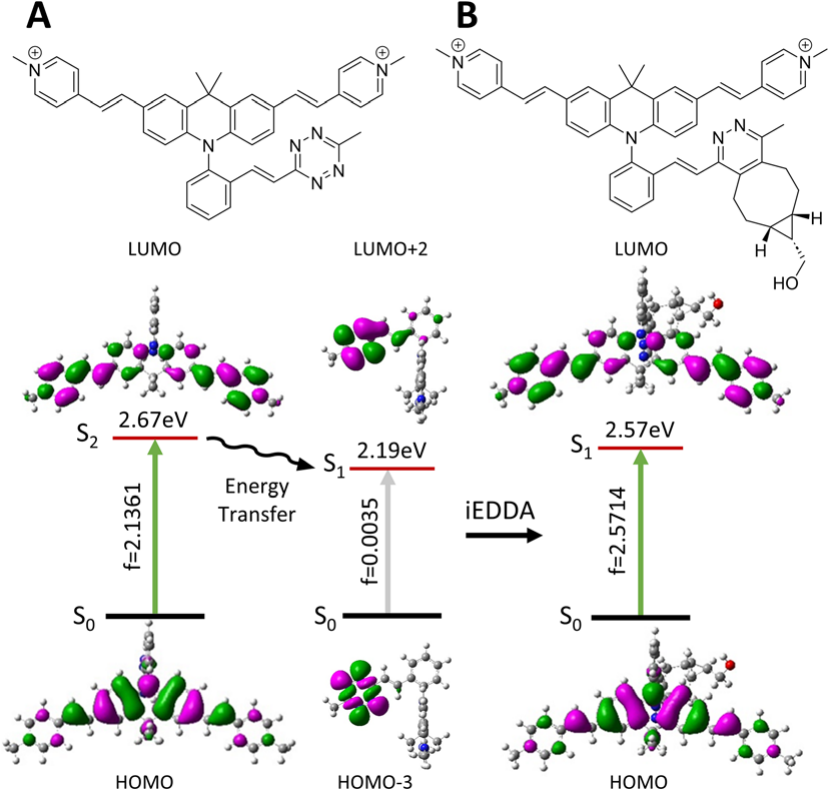
Molecular structures, optimized geometries, corresponding oscillator strength (f) and dominating frontier molecular orbitals involved during these transitions of (A) Acri-*o*vi and (B) Clicked Acri-*o*vi.

The photophysical properties of the initial Acri-Xvi and their clicked counterparts were then studied by TDDFT calculations. The theoretical and experimental absorption maxima were within the usual range for such models (mean averaged error <8%, ESI Table S7†). For all Acri-Xvi, the first calculated transition at 565 nm was located on the tetrazine and corresponded to a weakly allowed n–π* transition (low oscillator strength) (Fig. 5, ESI Fig. S9 and Table S8†). The second calculated vertical transition was a π–π* transition centered on the Acri chromophore, with a high oscillator strength, consistent with the high experimental *ε* values (Tables S2 and S3†). After the click reaction with BCN, only the Acri transition remained, allowing the restoration of fluorescence (ESI Table S8† and Fig. 5). The Acri-Xvi series was designed to undergo fluorescence quenching through excited state energy transfer (EET), which is highly dependent on the relative orientations of the chromophores involved. We hypothesized that the lower fluorescence quantum yield (and higher turn-on ratio following the click reaction) of Acri-*o*vi might be due to a more favorable interaction between the fluorophore and the quenching moiety than for the other two isomers. We quantified the interactions more accurately by evaluating the excitonic coupling between Acri and tetrazine with the transition charge from the electrostatic potential (TrESP) method (Table 4).^44^ The calculations revealed a much higher coupling for Acri-*o*vi (2.90 meV) than for Acri-*m*vi (1.35 meV) and Acri-*p*vi (0.93 meV), consistent with stronger quenching of emission by Acri-*o*vi. These data provide a rational basis for the greater reactivity and higher turn-on ratio of Acri-*o*vi.

**Table 4:** Calculated excitonic coupling energy of the Acri-vi series

Probe	Excitonic coupling energy (kcal mol^−1^)	Excitonic coupling energy (mEV)	*Φ*_F_
Acri-*o*vi	0.07	2.90	0.007
Acri-*m*vi	0.03	1.35	0.025
Acri-*p*vi	0.02	0.93	0.028

### Bioorthogonal labelling

**Fig. 6:**
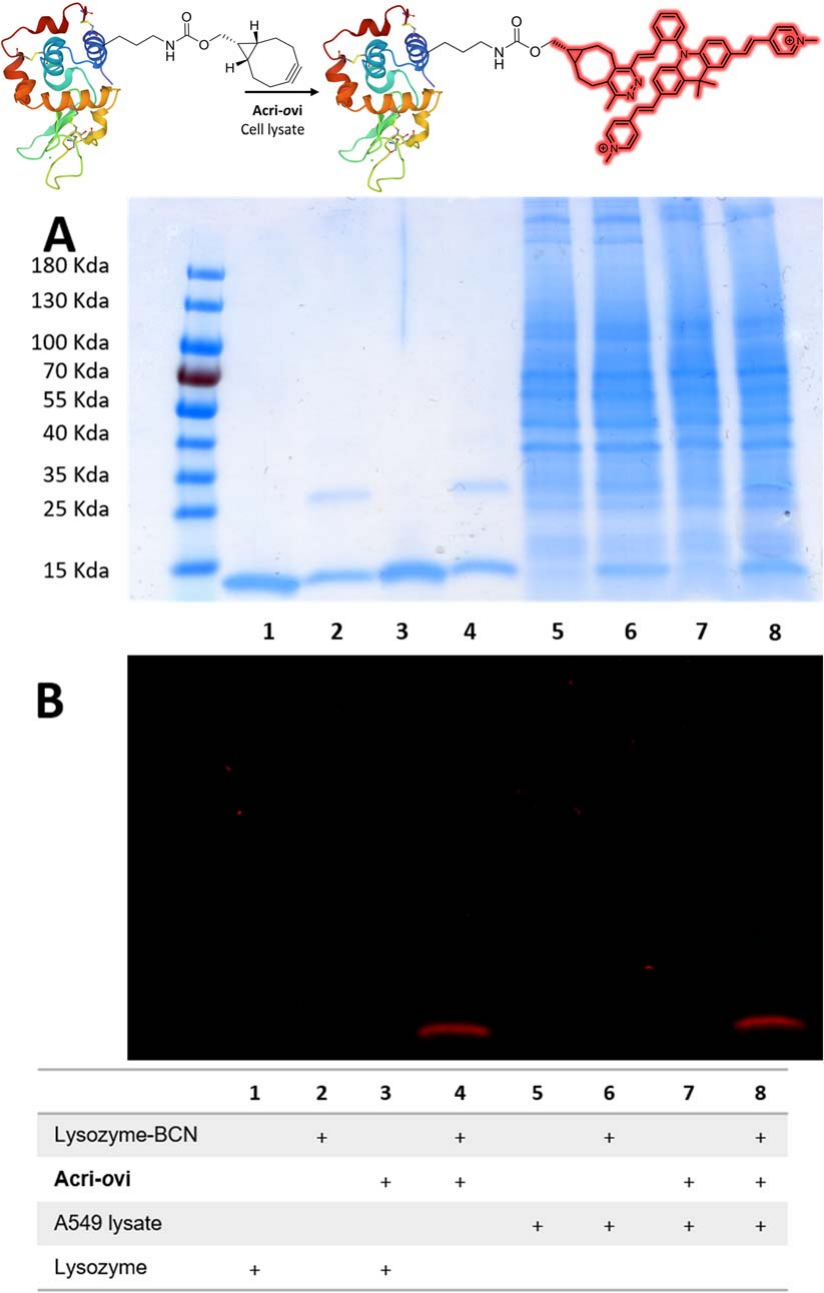
Reaction of Acri-*o*vi with lysozyme-BCN in the presence or absence of cell lysate. SDS-PAGE (10 μg per well). (A) Coomassie blue staining; (B) Fluorescence staining (*λ*_exc_ = 496 nm).

We then evaluated the ability of our best probe, Acri-*o*vi, to label a protein selectively in cell lysate. We first prepared and characterized human lysozyme modified with BCN by standard methods (ESI†). The reaction between BCN-tagged lysozyme and Acri-*o*vi proceeded rapidly at micromolar concentrations. After 10 minutes in aqueous buffer, the fluorogenic dye was completely converted to its click adduct with a turn-on ratio similar to that for BCN alone (turn-on = 25, ESI Fig. S11†). The crude reaction was analyzed by denaturing SDS-PAGE. The modified clicked lysozyme was detected by both Coomassie blue and fluorescence staining, whereas no fluorescence was detected for the mixture of native lysozyme protein and Acri-*o*vi (column 4 *versus* 3, Fig. 6). We then performed the reaction in A549 cell lysate, to investigate the behavior of our dye in a more complex cellular medium. Fluorescent labelling was observed for the reaction between Acri-*o*vi and the BCN-tagged lysozyme in the cell lysate, whereas no fluorescence was observed for Acri-*o*vi in the cell lysate (column 8 *versus* 7, Fig. 6). This finding confirmed that the fluorogenic click reaction between our probe and the BCN-tagged lysozyme occurred orthogonally to the lysate proteins, all of which remained unlabelled.

### Fluorescence microscopy

**Fig. 7:**
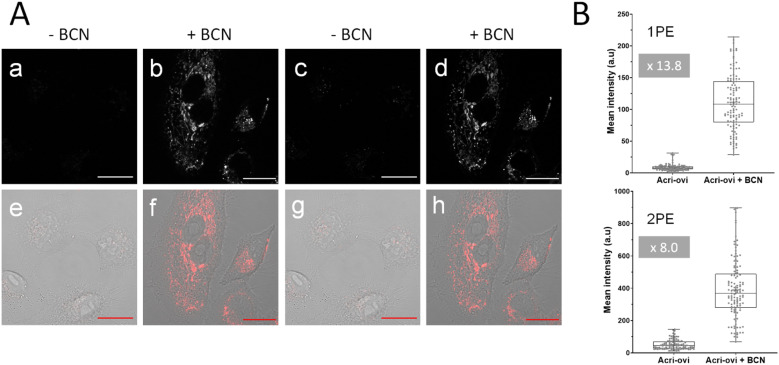
(A) Confocal microscopy imaging of live A549 cells incubated with Acri-*o*vi at 2 μM for 3 h and then with (b and d) or without (a and b) BCN at 50 μM for 30 min under one (496 nm, a and b) or two-photon excitation (840 nm, c and d). (e), (f), (g) and (h) The corresponding merged images (fluorescence and brightfield). Emission slit settings: 500–700 nm. Scale bar: 20 μm. (B) Fluorescence quantification in live A549 cells with Acri-*o*vi (2 μM) and BCN (10 μM). Box plot represents the fluorescence intensity obtained by measuring pixel means using ImageJ software under one (1PE) (up) or two-photon excitation (2PE) (down). Data come from three independent experiments, and settings and processing were identical for all images. ****p < 0.0001.

We then evaluated the potential of Acri-*o*vi for use in fluorogenic two-photon bioimaging. We first confirmed the biocompatibility of this probe by performing an MTT assay. Acri-*o*vi, like all the Acri-vi and Acri-et probes, had no significant effect on the viability of A549 cells, as the IC_80_ exceeded 10 μM (ESI Fig. S12†). Live A549 cells were incubated with 2 μM Acri-*o*vi for 3 h and imaged by one- and two-photon fluorescence microscopy (Fig. 7A, panels a and c). No staining was observed. After the addition of BCN and incubation for 30 min, fluorescence microscopy under the same imaging conditions (laser power) revealed clear cellular labelling, demonstrating the fluorogenicity of Acri-*o*vi in live cells (Fig. 7A, panels b and d). The click adduct exhibits high brightness and contrast under one and two-photon excitations. We then quantified cellular fluorescence intensities and determined the turn-on ratios in living cells. Fluorescence enhancements of 13.8-fold and 8.0-fold were measured under single and two-photon excitations, respectively. The difference between 1 PE and 2 PE turn-on ratios can be partly explained by the difference in one-photon and two-photon absorption properties between Acri-*o*vi and its clicked product. Indeed, in glycerol, we observed that the single-photon absorption of the clicked product was higher than that of Acri-*o*vi while the two-photon absorption of the clicked product was lower than the probe (Table 2 and Fig. 3). However, for both one photon and two-photon excitations, these turn-on values are high and unequivocally allow fluorogenic reactions without washing and with satisfactory contrast,^45^ especially since a value of 3 is known to be sufficient.^46^ Moreover, the high two-photon brightness of the clicked adduct allows the use of a low laser power for 2 PE (2.2 mW), which is considered far below that of the standard non-invasive *in vivo* two-photon microscopy studies.^47^ Thus, the combination of the low required laser power, the NIR excitation wavelength (840 nm) and the red-emitted light (600–700 nm) makes this probe a promising probe for *in vivo* two-photon excited microscopy.

## Conclusion

Two series of fluorogenic probes were synthesized in this comparative study of PeT and TBET designs on the same scaffold. The synthesis of these probes required methodological developments, in particular in order to overcome the sensitivity of tetrazine to nucleophiles. Both series of probes had interesting fluorogenic properties, with good-to-excellent reaction rate constants with BCN, a crucial criterion for bioorthogonal reactions. BCN was the best partner for fluorogenic reactions in terms of both turn-on ratio (22 for Acri-*o*vi) and rate constant (*k* = 1100 M^−1^ s^−1^ for Acri-*o*vi) as TCO led to different fluorescent and nonfluorescent species. Molecular modeling provided a rational explanation for the unexpectedly much higher rate constant for Acri-*o*vi than for its regioisomers. The iEDDA was identified as the rate-determining step and activation energy was lower for the *ortho* derivative. Finally, we found that the TBET design was more efficient that the PeT design on this scaffold. We also investigated the effect of the distance between the tetrazine moiety and the fluorophore, by connecting the fluorophore in the *ortho*, *meta* and *para* positions. Our data indicate that the efficiency of tetrazine quenching depends on this distance, with greater quenching activity for shorter distances. This effect of the distance was already known for PeT designs, but had not been reported for TBET designs. TDDFT calculations were performed to evaluate excitonic coupling, which was found to be much greater for Acri-*o*vi than for Acri-*m*vi and Acri-*p*vi, consistent with the stronger quenching observed with the *ortho* isomer. Acri-*o*vi is, therefore, our optimized fluorogenic probe and suitable for use in bioorthogonal reactions, as demonstrated by its unequivocal labelling of BCN-labelled lysozyme in a cell lysate. Moreover, these probes are the best two-photon excitable fluorogenic probes obtained to date, with impressive values for both cross-section and brightness (up to 20 times higher than previously reported).^37^ The high turn-on measured in cells (13.8 for 1P excitation and 8.0 for 2P) allows its use without any washing steps. Live-cell imaging by two-photon microscopy showed that Acri-*o*vi was an efficient fluorogenic probe that could be used with a very low laser power. This work paves the way for the further development of fluorogenic probes based on the Acri-*o*vi scaffold including for *in vivo* two-photon microscopy.

## Data availability

The datasets supporting this article have been uploaded as part of the ESI.†

## Author contributions

Chemical synthesis, characterization of compounds and kinetic studies were performed by M. A. and D. N.-M. Two-photon photophyiscal properties of the compounds were determined by F. B. Molecular modeling was performed by G. C. and M. A. Cellular imaging was performed by M. A. and G. F. F. M.-B. supervised the project. F. M.-B. and M. A. conceived the study and wrote the original draft. All authors commented on the manuscript and gave approval to the final version of the manuscript.

## Conflicts of interest

There are no conflicts to declare.

## Supplementary Material

SC-014-D3SC01754K-s001
