# Supplementary material for: Ultrabright two-photon excitable red-emissive fluorogenic probes for fast and wash-free bioorthogonal labelling in live cells
Source: Chem Sci. 2023 Jul 4;14(30):8119–28. doi: 10.1039/d3sc01754k (PMC10395273; doi:10.1039/d3sc01754k)

Electronic Supplementary Material (ESI) for Chemical Science.
This journal is © The Royal Society of Chemistry 2023

Ultrabright two-photon excitable fluorogenic probes for fast and wash-free biorthogonal labelling in live cells

Marie Auvray*, Delphine Naud-Martin, Gaëlle Fontaine, Gilles Clavier, Frédéric Bolze, Florence Mahuteau-Betzer*

I. Design of fluorogenic probes 2
II. Experimental procedures 4
III. NMR spectra of new compounds 21
IV. LC/MS of key intermediates 70
V. LC/MS of final compounds 76
VI. Stability of Acri-ovi in PBS at 37 °C 83
VII. LC/MS of clicked products 84
VIII. Photophysical measurements 90
X. BSA Titration 91
XII. Kinetics 94
XIII. Two-photon excitation spectra 95
XIV. Molecular modeling 97
XV. Cartesian coordinates of calculated structures 100
XVI. Click in cell lysate 157
XVII. MTT Assay 158
XVIII. Live-cell imaging 159

I. Design of fluorogenic probes

Figure S1: PeT design

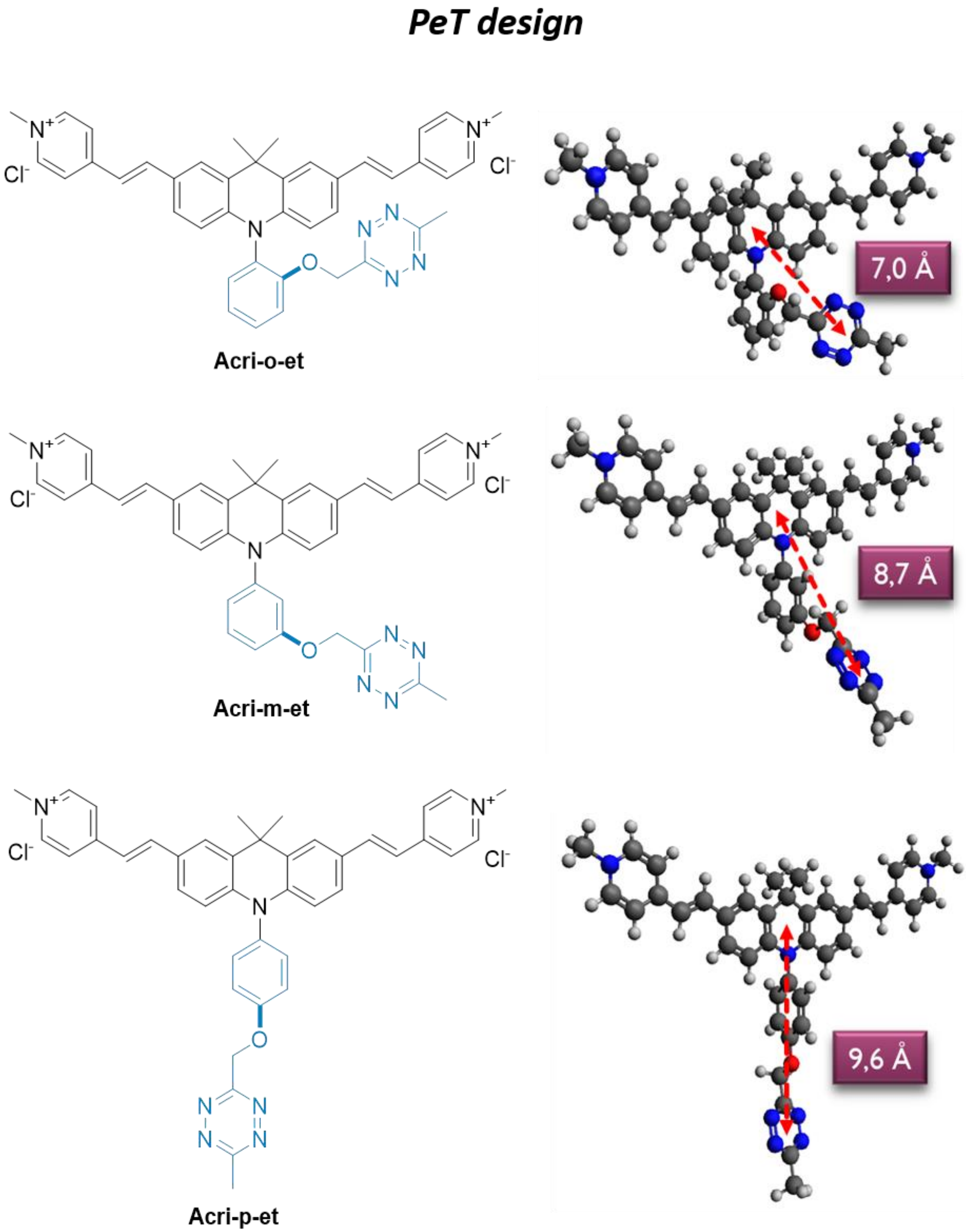

Figure S2: TBET design

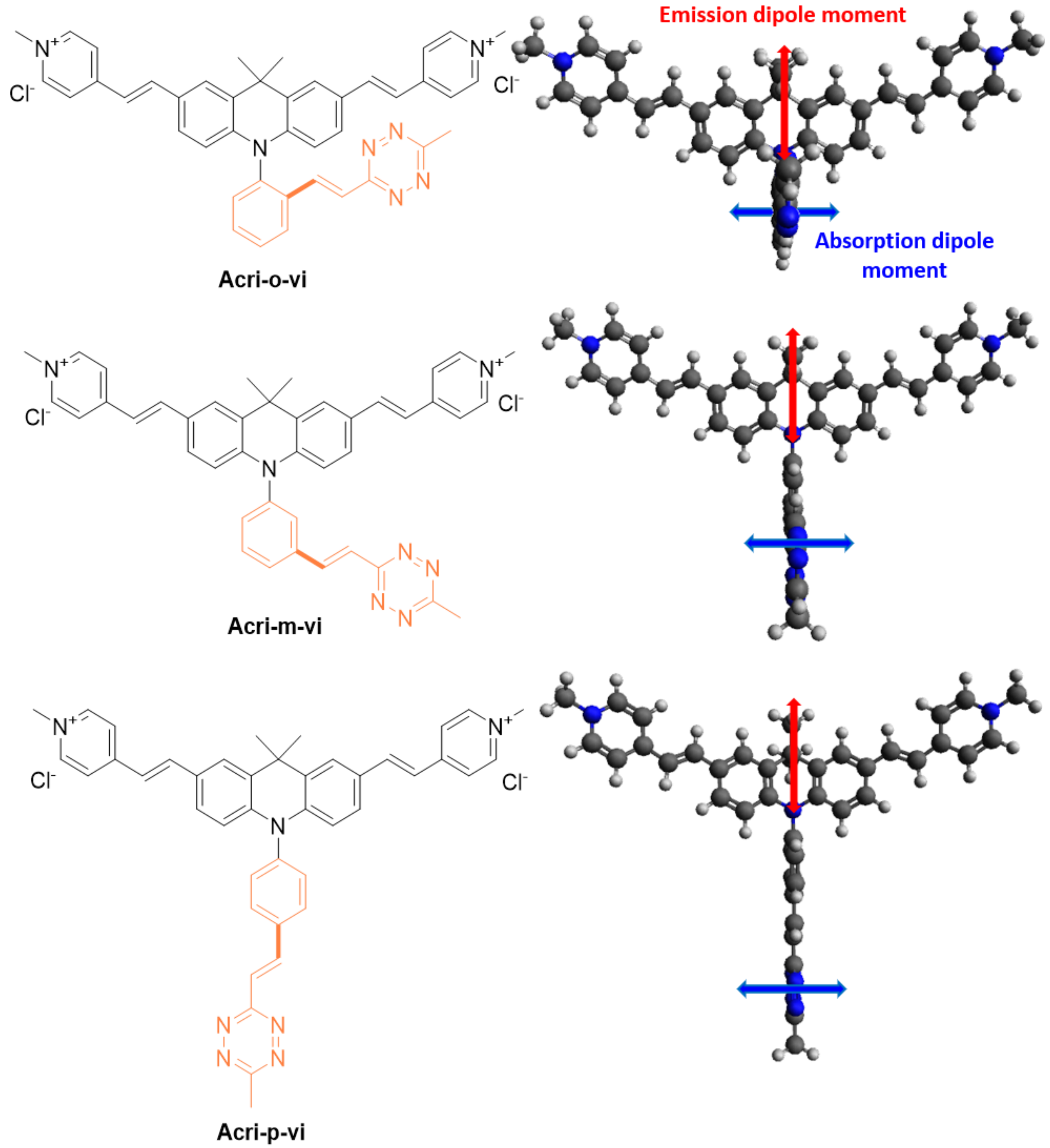

II. Experimental procedures

Reagents were purchased as reagent-grade and used without further purification unless otherwise stated. THF, DMF, DCM, Et_3N and pyrrolidine were freshly distilled before use. The concentration of *n*-BuLi in hexanes was determined by titration performed in triplicate prior to use. Analytical thin-layer chromatographies (TLC) were carried out on Macherey Nagel Alugram Xtra SIL G/UV254 plates using UV light (λ = 254 nm) as visualizing agent. Flash chromatographies were carried out with Macherey Nagel silica gel (40-63 µm) on CombiFlash Companion from Teledyne Isco equipped with packed silica cartridges from Macherey Nagel. Preparative TLC were carried out on Macherey Nagel TLC plates SIL G-200 UV254. NMR spectra were recorded on Bruker AV300 spectrometer at room temperature. Chemical shifts (δ), which are expressed in part per million (ppm), were determined relative to residual non-deuterated solvent as an internal reference respectively CD_3OD, $CDCl_3$, $(CD_3)_2SO$ (^{13}C NMR: δ = 49.00; 77.23; 39.52 ppm; 1H NMR: δ = 3.31; 7.26; 2.50 ppm). Coupling constant(s) in hertz (Hz) were measured from one-dimensional spectra and multiplicities were abbreviated as following: s (singlet), d (doublet), t (triplet), q (quadruplet), m (multiplet). Melting points were measured with a Melting Point Apparatus SMP30 (Stuart). LC-MS spectra (ESI in the positive ion mode) were recorded on a Waters Micromass ZQ instrument coupled to a Waters Alliance Separations Module 2695, equipped with a Luna Omega C18 - 3 µm 100 Å column (3.0 x 50 mm) and a Waters PDA 2998, using the following gradient (Solvent A: H_2O + 0.1% Formic Acid, Solvent B: CH_3CN + 0.1% Formic Acid):

Time (min)	*A (%)*	*B (%)*	*Flow (mL/min)*
0	95	5	0.5
1	95	5	0.5
6	0	100	0.5
8	0	100	0.5
8.5	95	5	0.6
10.5	95	5	0.6
11	95	5	0.5

10-(2-methoxyphenyl)-9,9-dimethyl-9,10-dihydroacridine, **1-*o***:

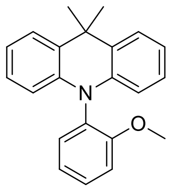

Chemical Formula: $C_{22}H_{21}NO$
Exact Mass: 315,1623

Under Ar atmosphere, 9,9-dimethyl-9,10-dihydroacridine (300 mg, 1.43 mmol, 1.0 eq.), 2-iodoanisole (0.28 mL, 2.15 mmol, 1.5 eq.), *t*-BuONa (413 mg, 4.30 mmol, 3.0 eq.), $Pd_2(dba)_3$ (131 mg, 0.14 mmol, 0.1 eq.) and $P(t\text{-}Bu)_3.HBF_4$ (125 mg, 0.43 mmol, 0.3 eq.) were dissolved in freshly degassed (by Ar bubbling for 15 min) dry toluene (8 mL). The solution was stirred at 110 °C overnight. AcOEt was introduced. The organic layer was washed once with brine, and dried over Na_2SO_4. The crude was purified by flash chromatography on silica gel (Cyclohexane/AcOEt, 100:0 to 90:10) to give the expected product as a white solid (400 mg, 1.27 mmol, **η = 88 %**). **TLC**: R_f (Cyclohexane/AcOEt, 9:1) = 0.59; **Melting point**: 112 °C; **^{1}H NMR** (300 MHz, $CDCl_3$): δ = 7.52 – 7.44 (m, 3H), 7.29 (dd, *J* = 8.0 Hz, 1.5 Hz, 1H), 7.18 – 7.13 (m, 2H), 6.97 (td, *J* = 7.5 Hz, 1.5 Hz, 2H), 6.91 (td, *J* = 7.5 Hz, 1.5 Hz, 2H), 6.26 (dd, *J* = 8.0 Hz, 1.0 Hz, 2H), 3.70 (s, 3H), 1.69 (s, 6H); **^{13}C NMR** (75 MHz,

CDCl$_3$): δ = 157.7 (C_q), 140.5 (C_q), 132.9 (CH), 130.3 (C_q), 129.9 (CH), 129.0 (C_q), 126.5 (CH), 125.1 (CH), 122.1 (CH), 120.5 (CH), 113.7 (CH), 113.1 (CH), 55.8 (CH), 36.1 (C_q), 31.1 (CH_3); **LC/MS** (ES+): *m/z* = 316.3 ($[M+H]^+$), tr = 7.93 min; **HRMS** (ES+): *m/z* calculated for $C_{22}H_{22}NO^+$: 316.1696, found: 316.1689.

10-(2-methoxyphenyl)-9,9-dimethyl-9,10-dihydroacridine-2,7-dicarbaldehyde, **2-*o***:

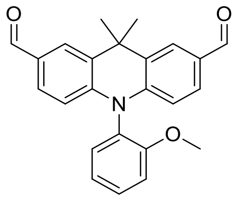

Chemical Formula: $C_{24}H_{21}NO_3$
Exact Mass: 371,1521

Under Ar atmosphere and at 0°C, $POCl_3$ (7.39 mL, 79.3 mmol, 50 eq.) was added dropwise to dry DMF (20 mL). After 1h stirring at 0°C, compound **1-*o*** (500 mg, 1.59 mmol, 1.0 eq.) was introduced. The solution was stirred at 95 °C for 24h. The dark mixture was poured into an ice bath and this solution was quenched by NaOH 3M. The aqueous layer was extracted three times with AcOEt. Organic layers were combined, washed with brine and dried over Na_2SO_4. The crude was purified by flash chromatography on silica gel (Cyclohexane/AcOEt, 100:0 to 70:30) to give the expected product as a pale-yellow solid (513 mg, 1.38 mmol, **η = 87 %**). **TLC**: R_f (Cyclohexane/AcOEt, 7:3) = 0.43; **Melting point**: 105 °C; **^{1}H NMR** (300 MHz, CDCl$_3$): δ = 9.86 (s, 2H), 8.01 (d, *J* = 1.5 Hz, 2H), 7.58 (td, *J* = 8.0 Hz, 2.0 Hz, 1H), 7.51 (dd, *J* = 8.5 Hz, 1.5 Hz, 2H), 7.29 (td, *J* = 7.5 Hz, 1.5 Hz, 1H,), 7.20 (m, 2H), 6.40 (d, *J* = 8.5 Hz, 2H), 3.72 (s, 3H), 1.79 (s, 3H), 1.74 (s, 3H); **^{13}C NMR** (75 MHz, CDCl$_3$): δ = 190.9 (CH), 156.7 (C_q), 144.5 (C_q), 131.7 (CH), 131.2 (CH), 131.0 (C_q), 130.8 (C_q), 129.7 (CH), 127.5 (CH), 127.3 (C_q), 122.4 (CH), 114.7 (CH), 113.3 (CH), 55.9 (CH_3), 36.3 (C_q), 31.8 (CH_3), 31.4 (CH_3); **LC/MS** (ES+): *m/z* = 372.3 ($[M+H]^+$), tr = 7.21 min; **HRMS** (ES+): *m/z* calculated for $C_{24}H_{22}NO_3^+$: 372.1594, found: 372.1590.

10-(2-hydroxyphenyl)-9,9-dimethyl-9,10-dihydroacridine-2,7-dicarbaldehyde, **3-*o***:

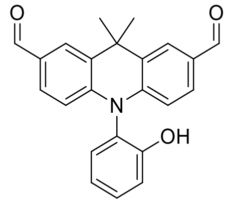

Chemical Formula: $C_{23}H_{19}NO_3$
Exact Mass: 357,1365

At 0°C and under Ar atmosphere, ether **2-*o*** (340 mg, 0.92 mmol, 1.0 eq.) was dissolved in dry DCM (10 mL) and BBr_3 (0.43 mL, 4.58 mmol, 5.0 eq.) was added dropwise. The solution was then stirred for 3h. Water was introduced. The aqueous layer was extracted once with DCM. The organic layer was washed with brine and dried over Na_2SO_4. The crude was purified by flash chromatography on silica gel (Cyclohexane/AcOEt, 90:10 to 50:50) to give the expected product as a yellow solid (217 mg, 0.61 mmol, **η = 66 %**). **TLC**: R_f (Cyclohexane/AcOEt, 1:1) = 0.56; **Melting point**: > 240 °C ; **^{1}H NMR**

(300 MHz, $CDCl_3$): δ = 9.80 (s, 2H), 7.99 (d, *J* = 1.5 Hz, 2H), 7.55 – 7.47 (m, 3H), 7.26 – 7.15 (m, 3H), 6.47 (d, *J* = 8.5 Hz, 2H), 5.89 (s, 1H), 1.81 (s, 3H), 1.69 (s, 3H); **^{13}C NMR** (75 MHz, $CDCl_3$): δ = 191.0 (CH), 153.6 (C_q), 143.9 (C_q), 131.5 (CH), 131.2 (C_q), 131.1 (C_q), 130.8 (CH), 130.2 (CH), 127.8 (CH), 125.6 (C_q), 123.0 (CH), 118.1 (CH), 114.9 (CH), 36.3 (C_q), 33.2 (CH_3), 30.8 (CH_3); **LC/MS** (ES+): *m/z* = 358.2 ($[M+H]^+$), tr = 6.73 min; **HRMS** (ES+): *m/z* calculated for $C_{23}H_{20}NO_3^+$: 358.1438, found: 358.1451.

3-(methoxymethyl)- 6-methyl-1,2,4,5-tetrazine:

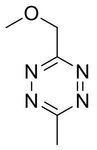

Chemical Formula: $C_5H_8N_4O$
Exact Mass: 140,0698

Under Ar atmosphere, a solution of 2-methoxyacetonitrile (1.0 mL, 6.37 mmol, 1.0 eq.), ACN (5.66 mL, 108 mmol, 8.0 eq.), EtOH (2.31 mL, 39.6 mmol, 3.0 eq.) and 3-mercaptopropionic acid (1.17 mL, 13.5 mmol, 1.0 eq) was cooled to 0°C. Hydrazine hydrate (7.83 mL, 161 mmol, 12.0 eq.) was then added dropwise. The solution was then stirred at r.t. for 72h. A saturated aqueous solution of sodium nitrite (13.9 g, 202 mmol, 15 eq.) was added into the reaction mixture, which was cooled to 0°C. A solution of HCl 1M was then added dropwise until gas release ceased. The pink aqueous layer was extracted with DCM. Organic was washed with brine and dried over Na_2SO_4. The crude was purified by flash chromatography on silica gel (Cyclohexane/AcOEt, 100:0 to 70:30) to give the expected product as a pink solid (620 mg, 4.42 mmol, **η = 33 %**). **TLC**: R_f (Pentane/Et_2O, 1:1) = 0.51; **CAS number** [2067322-21-6]; **^{1}H NMR** (300 MHz, $CDCl_3$): δ = 5.05 (s, 2H), 3.61 (s, 3H), 3.10 (s, 3H); **LC/MS** (ES+): *m/z* = 141.3 ($[M+H]^+$), tr = 2.31 min.

Spectroscopic data were in accordance with those previously reported in the literature.[1]

(6-methyl-1,2,4,5-tetrazin-3-yl)methanol and 3-(bromomethyl)- 6-methyl-1,2,4,5-tetrazine were then synthesized following published procedures.[1]

9,9-dimethyl-10-{2-[(6-methyl-1,2,4,5-tetrazin-3yl)methoxy]phenyl}-9,10-dihydroacridine-2,7-dicarbaldehyde, **5-*o***:

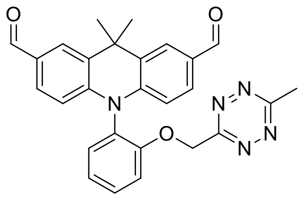

Chemical Formula: $C_{27}H_{23}N_5O_3$
Exact Mass: 465,1801

[1] Werther, P.; Yserentant, K.; Braun, F.; Grußmayer, K.; Navikas, V.; Yu, M.; Zhang, Z.; Ziegler, M. J.; Mayer, C.; Gralak, A. J.; Busch, M.; Chi, W.; Rominger, F.; Radenovic, A.; Liu, X.; Lemke, E. A.; Buckup, T.; Herten, D.-P.; Wombacher, R., Bio-orthogonal Red and Far-Red Fluorogenic Probes for Wash-Free Live-Cell and Super-resolution Microscopy. *ACS central science* **2021**.

Under Ar atmosphere, phenol **3-*o*** (110 mg, 0.31 mmol, 1.0 eq.) and bromotetrazine **4** (116 mg, 0.62 mmol, 2.0 eq.) were dissolved in dry THF/DMF 1:1 mixture (12 mL). This solution was cooled to 0°C and Cs_2CO_3 (150 mg, 0.46 mmol, 1.5 eq.) was added. This solution was then stirred at 50 °C for 4h. A saturated NH_4Cl aqueous solution was introduced. The aqueous layer was extracted twice with AcOEt. Organic layers were combined, washed with brine and dried over Na_2SO_4. The crude was purified by preparative TLC (2 * Thickness: 0.5 mm, Cyclohexane/AcOEt, 50:50) to give the expected product as a red solid (101 mg, 0.22 mmol, **η = 70 %**). **TLC**: R_f (Cyclohexane/AcOEt, 1:1) = 0.50; **^{1}H NMR** (300 MHz, $CDCl_3$): δ = 9.84 (s, 2H), 7.97 (d, *J* = 1.5 Hz, 2H), 7.61 – 7.53 (m, 1H), 7.48 (dd, *J* = 8.5 Hz, 1.5 Hz, 2H), 7.38 – 7.26 (m, 3H), 6.40 (d, *J* = 8.5 Hz, 2H), 5.60 (s, 2H), 2.99 (s, 3H), 1.72 (s, 3H), 1.68 (s, 3H) ; **^{13}C NMR** (75 MHz, $CDCl_3$): δ = 190.9 (CH), 168.8 (C_q), 165.0 (C_q), 155.0 (C_q), 144.2 (C_q), 132.3 (CH), 131.1 (CH), 130.9 (C_q), 130.8 (C_q), 129.6 (CH), 128.2 (C_q), 127.5 (CH), 123.9 (CH), 115.2 (CH), 114.8 (CH), 68.1 (CH_2), 36.2 (C_q), 31.7 (CH_3), 31.6 (CH_3), 21.4 (CH_3) ; **LC/MS** (ES+): *m/z* = 466.4 ($[M+H]^+$), tr = 6.99 min ; **HRMS** (ES+): *m/z* calculated for $C_{27}H_{24}N_5O_3^+$: 466.1874, found: 466.1867.

Fluorogenic probe **Acri-*o*et**:

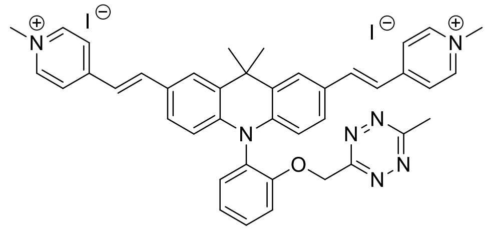

Chemical Formula: $C_{41}H_{39}N_7O^{2+}$
Exact Mass: 645,3205

Aldehyde ***5-o*** (25 mg, 54 µmol, 1.0 eq.) was solubilized in dry DMF (1 mL). This solution was cooled to 0°C and pyrrolidine (9.3 µL, 0.11 mmol, 2.1 eq.) was then added. After 15 min stirring, AcOH (31 µL, 0.54 mmol, 10 eq.) and 1,4-dimethylpyridinium iodide **6** (24 mg, 0.10 mmol, 1.9 eq.) were introduced. The resulting mixture was stirred at 4°C for 18h. Diethyl ether (3 mL) was added: a precipitate appeared. It was filtered and washed with diethyl ether to give the expected product as a red solid (28 mg, 31 µmol, **η = 64 %**); **^{1}H NMR** (300 MHz, DMSO-d_6): δ = 8.79 (d, *J* = 6.5 Hz, 4H), 8.15 (d, *J* = 6.5 Hz, 4H), 8.00 (d, *J* = 16.0 Hz, 2H, H_{11}), 7.94 (d, *J* = 1.0 Hz, 2H), 7.66 – 7.57 (m, 2H), 7.47 – 7.31 (m, 6H), 6.25 (d, *J* = 8.5 Hz, 2H), 5.72 (s, 2H), 4.22 (s, 6H), 2.90 (s, 3H), 1.74 (s, 3H), 1.68 (s, 3H); **^{13}C NMR** (75 MHz, DMSO-d_6): δ = 168.1 (C_q), 164.7 (C_q), 154.9 (C_q), 153.0 (C_q), 144.8 (CH), 140.9 (CH), 140.8 (C_q), 131.9 (CH), 130.9 (CH), 130.5 (C_q), 128.3 (C_q), 127.6 (C_q), 127.5 (CH), 126.3 (CH), 123.5 (CH), 122.8 (CH), 119.9 (CH), 116.0 (CH), 114.5 (CH), 67.8 (CH_2), 46.7 (CH_3), 35.8 (C_q), 32.0 (CH_3), 31.7 (CH_3), 21.0 (CH_3); **LC/MS** (ES+): *m/z* = 322.9 ($[M-2I^-/2]^+$), tr = 5.30 min; **HRMS** (ES+): *m/z* calculated for $C_{41}H_{39}N_7OI^+$: 772.2255, found: 772.2278.

10-(3-methoxyphenyl)-9,9-dimethyl-9,10-dihydroacridine, **1-*m***:

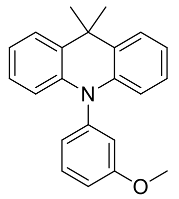

Chemical Formula: $C_{22}H_{21}NO$
Exact Mass: 315,1623

Under Ar atmosphere, 9,9-dimethyl-9,10-dihydroacridine (500 mg, 2.39 mmol, 1.0 eq.), 3-bromoanisole (0.46 mL, 3.58 mmol, 1.5 eq.), *t*-BuONa (689 mg, 7.17 mmol, 3.0 eq.), $Pd_2(dba)_3$ (219 mg, 0.24 mmol, 0.1 eq.) and P(*t*-Bu)$_3$.HBF_4 (208 mg, 0.72 mmol, 0.3 eq.) were dissolved in freshly degassed (by Ar bubbling for 15 min) dry toluene (25 mL). The solution was stirred at 110 °C overnight. AcOEt was introduced. The organic layer was washed once with brine and dried over Na_2SO_4. The crude was purified by flash chromatography on silica gel (Cyclohexane/AcOEt, 100:0 to 90:10) to give the expected product as a white solid (650 mg, 2.06 mmol, **η = 86 %**). **TLC**: R_f (Cyclohexane/AcOEt, 9:1) = 0.83 , Rf (Cyclohexane) = 0.14; **Melting point**: 96 °C; **^{1}H NMR** (300 MHz, $CDCl_3$): δ = 7.53 (t, *J* = 8.0 Hz, 1H), 7.46 (dd, *J* = 7.5 Hz, 1.5 Hz, 2H), 7.08 – 7.04 (m, 1H), 7.03 – 6.89 (m, 5H), 6.89 – 6.86 (m, 1H), 6.33 (dd, *J* = 8.0 Hz, 1.5 Hz, 2H), 3.83 (s, 3H), 1.70 (s, 6H); **^{13}C NMR** (75 MHz, $CDCl_3$): δ = 161.9 (C_q), 142.4 (C_q), 140.9 (C_q), 131.6 (CH), 130.0 (C_q), 126.5 (CH), 125.4 (CH), 123.4 (CH), 120.6 (CH), 116.2 (CH), 114.5 (CH), 114.2 (CH) , 55.6 (CH_3), 36.1 (C_q), 31.6 (CH_3); **LC/MS** (ES+): *m/z* = 316.4 ([M+H]$^+$), tr = 8.50 min; **HRMS** (ES+): *m/z* calculated for $C_{22}H_{22}NO^+$: 316.1696, found: 316.1698.

10-(3-methoxyphenyl)-9,9-dimethyl-9,10-dihydroacridine-2,7-dicarbaldehyde, **2-*m***:

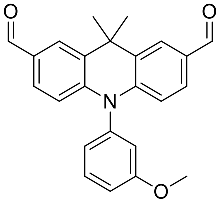

Chemical Formula: $C_{24}H_{21}NO_3$
Exact Mass: 371,1521

Under Ar atmosphere and at 0°C, $POCl_3$ (5.0 mL, 53.9 mmol, 25 eq.) was added dropwise to dry DMF (15 mL). After 1h stirring at 0°C, compound **1-*m*** (680 mg, 2.16 mmol, 1.0 eq.) was introduced. The solution was stirred at 95 °C for 24h. The dark mixture was poured into an ice bath and this solution was quenched by NaOH 3M. The aqueous layer was extracted three times with AcOEt. Organic layers were combined, washed with brine and dried over Na_2SO_4. The crude was purified by flash chromatography on silica gel (Cyclohexane/AcOEt 100:0 to 70:30) to give the expected product as a pale-yellow solid (435 mg, 1.17 mmol, **η = 54 %**). **TLC**: R_f (Cyclohexane/AcOEt, 7:3) = 0.45; **Melting point**: 180 °C; **^{1}H NMR** (300 MHz, $CDCl_3$): δ = 9.86 (s, 2H), 8.02 (d, *J* = 1.5 Hz, 2H), 7.60 (t, *J* = 8.0 Hz, 1H), 7.51 (dd, *J* = 8.5, 1.5 Hz, 2H), 7.14 (dd, *J* = 8.0, 2.0 Hz, 1H), 6.92 – 6.90 (m, 1H), 6.84 (t, *J* = 2.0 Hz, 1H), 6.46 (d, *J* = 8.5 Hz, 2H), 3.87 (s, 3H), 1.77 (s, 6H); **^{13}C NMR** (75 MHz, $CDCl_3$): δ = 190.9 (CH), 162.2 (C_q), 144.5 (C_q), 140.7 (C_q), 132.2 (CH), 130.8 (C_q), 130.6 (C_q), 129.7 (CH), 127.6 (CH), 122.1

(CH), 115.5 (CH), 115.3 (CH), 115.2 (CH), 55.7 (CH_3), 36.2 (C_q), 32.2 (CH_3); **LC/MS** (ES+): *m/z* = 372.3 ($[M+H]^+$), tr = 7.93 min; **HRMS** (ES+): *m/z* calculated for $C_{24}H_{22}NO_3^+$: 372.1594, found: 372.1593

10-(3-hydroxyphenyl)-9,9-dimethyl-9,10-dihydroacridine-2,7-dicarbaldehyde, **3-*m***:

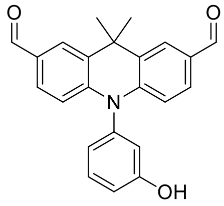

Chemical Formula: $C_{23}H_{19}NO_3$
Exact Mass: 357,1365

At 0°C and under Ar atmosphere, ether **2-*m*** (340 mg, 1.13 mmol, 1.0 eq.) was dissolved in dry DCM (10 mL) and BBr_3 (1.32 mL, 14.0 mmol, 12.0 eq.) was added dropwise. The solution was then stirred for 3h. Water was introduced. The aqueous layer was extracted once with DCM. The organic layer was washed with brine and dried over Na_2SO_4. The crude was purified by flash chromatography on silica gel (Cyclohexane/AcOEt, 90:10 to 50:50) to give the expected product as a yellow solid (352 mg, 0.98 mmol, **η = 87 %**). **TLC**: R_f (Cyclohexane/AcOEt, 1:1) = 0.50; **Melting point**: 208 °C; **^{1}H NMR** (300 MHz, $CDCl_3$): δ = 9.85 (s, 2H), 8.00 (d, *J* = 1.0 Hz, 2H), 7.56 (t, *J* = 8.0 Hz, 1H), 7.50 (dd, *J* = 8.5 Hz, 1.5 Hz, 2H), 7.09 (dd, *J* = 8.0 Hz, 1.5 Hz, 1H), 6.89 (d, *J* = 8.0 Hz, 1H), 6.82 (s, 1H), 6.47 (d, *J* = 8.5 Hz, 2H), 5.73 (s, 1H), 1.74 (s, 6H); **^{13}C NMR** (75 MHz, $CDCl_3$): δ = 191.0 (CH), 158.4 (C_q), 144.6 (C_q), 140.8 (C_q), 132.5 (CH), 130.8 (C_q), 130.7 (C_q), 129.7 (CH), 127.7 (CH), 122.2 (CH), 117.2 (CH), 116.8 (CH), 115.3 (CH), 36.3 (C_q), 32.1 (CH_3); **LC/MS** (ES+): *m/z* = 358.3 ($[M+H]^+$), tr = 7.10 min; **HRMS** (ES+): *m/z* calculated for $C_{23}H_{20}NO_3^+$: 358.1438, found: 358.1450.

9,9-dimethyl-10-{2-[(6-methyl-1,2,4,5-tetrazin-3yl)methoxy]phenyl}-9,10-dihydroacridine-2,7-dicarbaldehyde, **5-*m***:

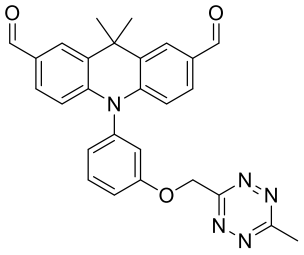

Chemical Formula: $C_{27}H_{23}N_5O_3$
Exact Mass: 465,1801

Under Ar atmosphere, phenol **3-*m*** (100 mg, 0.28 mmol, 1.0 eq.) and bromotetrazine **4** (106 mg, 0.56 mmol, 2.0 eq.) were dissolved in dry THF (5.5 mL). This solution was cooled to 0°C and Cs_2CO_3 (137 mg, 0.42 mmol, 1.5 eq.) was added. This solution was then stirred at r.t. for 3h. A saturated NH_4Cl aqueous solution was introduced. The aqueous layer was extracted twice with AcOEt. Organic layers were combined, washed with brine and dried over Na_2SO_4. The crude was purified by flash chromatography on silica gel (Cyclohexane/AcOEt, 100:0 to 50:50) and then by preparative TLC (Thickness: 2.0 mm, Cyclohexane/AcOEt 40:60) to give the expected product as a red solid (80 mg, 0.17 mmol, **η = 62 %**).

TLC: R_f (Cyclohexane/AcOEt, 1:1) = 0.51; **^{1}H NMR** (300 MHz, $CDCl_3$): δ = 9.87 (s, 2H), 8.01 (d, *J* = 1.5 Hz, 2H), 7.65 (t, *J* = 8.5 Hz, 1H), 7.51 (dd, *J* = 8.5 Hz, 1.5 Hz, 2H), 7.30 (dd, *J* = 8.5 Hz, 1.5 Hz, 1H), 7.05 – 6.96 (m, 2H), 6.42 (d, *J* = 8.5 Hz, 2H), 5.71 (s, 2H), 3.12 (s, 3H), 1.76 (s, 6H); **^{13}C NMR** (75 MHz, $CDCl_3$): δ = 190.9 (CH), 169.1 (C_q), 165.3 (C_q), 160.4 (C_q), 144.4 (C_q), 140.9 (C_q), 132.6 (CH), 130.9 (C_q), 130.7 (C_q), 129.7 (CH), 127.6 (CH), 123.6 (CH), 117.0 (CH), 116.1 (CH), 115.2 (CH), 68.3 (CH_2), 36.3 (C_q), 32.1 (CH_3), 21.5 (CH_3); **LC/MS** (ES+): *m/z* = 466.2 ($[M+H]^+$), tr = 7.68 min; **HRMS** (ES+): *m/z* calculated for $C_{27}H_{24}N_5O_3^+$: 466.1874, found: 466.1872.

Fluorogenic probe **Acri-*m*et**:

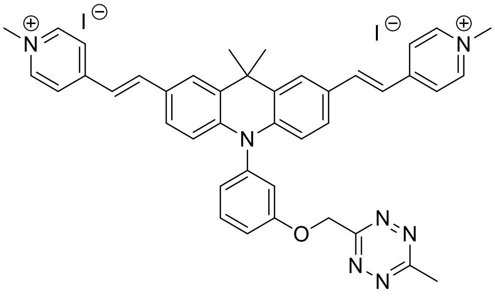

Chemical Formula: $C_{41}H_{39}N_7O^{2+}$
Exact Mass: 645,3205

Aldehyde **5-*m*** (26 mg, 57 µmol, 1.0 eq.) was solubilized in dry DMF (1 mL). This solution was cooled to 0°C and pyrrolidine (12 µL, 0.15 mmol, 2.6 eq.) was then added. After 15 min stirring, AcOH (41 µL, 0.71 mmol, 12 eq.) and 1,4-dimethylpyridinium iodide **6** (32 mg, 0.14 mmol, 2.5 eq.) were introduced. The resulting mixture was stirred at 4°C for 18h. Diethyl ether (3 mL) was added: a precipitate appeared. It was filtered and washed with diethyl ether to give the expected product as a red solid (21 mg, 23 µmol, **η = 41 %**). **^{1}H NMR** (300 MHz, MeOD-d_4): δ = 8.62 (d, *J* = 6.5 Hz, 4H), 8.08 (d, *J* = 6.5 Hz, 4H), 7.94 – 7.89 (m, 4H), 7.69 (t, *J* = 8.0 Hz, 1H), 7.45 (d, *J* = 8.5 Hz, 2H), 7.38 (dd, *J* = 8.0, 2.0 Hz, 1H), 7.29 (d, *J* = 16.0 Hz, 2H), 7.11 (s, 1H), 7.01 (d, *J* = 7.5 Hz, 1H), 6.38 (d, *J* = 8.5 Hz, 2H), 5.75 (s, 2H), 4.27 (s, 6H), 3.04 (s, 3H), 1.81 (s, 6H); **^{13}C NMR** (75 MHz, CD_3CN/MeOD-d_4 1:1): δ = 169.7 (C_q), 166.3 (C_q), 161.4 (C_q), 155.0 (C_q), 145.3 (CH), 142.6 (C_q), 142.3 (CH), 142.0 (C_q), 133.1 (CH), 131.6 (C_q), 129.4 (C_q), 128.1 (CH), 127.7 (CH), 124.1 (CH), 124.0 (CH), 123.9 (CH), 120.5 (CH), 117.7 (CH), 116.9 (CH), 116.1 (CH), 68.8 (CH_2), 47.5 (CH_3), 36.8 (C_q), 32.4 (CH_3), 21.2 (CH_3); **LC/MS** (ES+): *m/z* = 323.0 ($[M/2]^+$), tr = 5.18 min; **HRMS** (ES+): *m/z* calculated for $C_{41}H_{39}N_7OI^+$: 772.2255, found: 772.2296.

10-(4-methoxyphenyl)-9,9-dimethyl-9,10-dihydroacridine, **1-*p***:

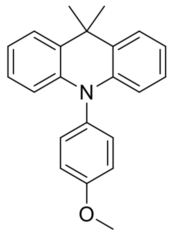

Chemical Formula: $C_{22}H_{21}NO$
Exact Mass: 315,1623

Under Ar atmosphere, 9,9-dimethyl-9,10-dihydroacridine (500 mg, 2.39 mmol, 1.0 eq.), 4-bromoanisole (0.45 mL, 3.58 mmol, 1.5 eq.), *t*-BuONa (689 mg, 7.17 mmol, 3.0 eq.), $Pd(dba)_2$ (137 mg, 0.24 mmol, 0.1 eq.) and $P(t\text{-}Bu)_3.HBF_4$ (208 mg, 0.72 mmol, 0.3 eq.) were dissolved in freshly degassed (by Ar bubbling for 15 min) dry toluene (13.5 mL). The solution was stirred at 110 °C overnight. AcOEt was introduced. The organic layer was washed once with brine and dried over Na_2SO_4. The crude was purified by flash chromatography on silica gel (Cyclohexane/AcOEt 100:0 to 90:10) to give the expected product as a white solid (720 mg, 2.28 mmol, **η = 96 %**). **CAS number** [2095203-20-4]; **TLC**: R_f (Cyclohexane/AcOEt, 9:1) = 0.66; **Melting point**: 145 °C; **^{1}H NMR** (300 MHz, $CDCl_3$): δ = 7.44 (dd, *J* = 7.5, 1.5 Hz, 2H), 7.22 (d, *J* = 9.0 Hz, 2H), 7.12 (d, *J* = 9.0 Hz, 2H), 7.00 – 6.94 (m, 2H), 6.91 (td, *J* = 7.5, 1.5 Hz, 2H), 6.30 (dd, *J* = 8.0, 1.0 Hz, 2H), 3.91 (s, 3H), 1.68 (s, 6H); **LC/MS** (ES+): *m/z* = 316.4 ($[M+H]^+$), tr = 8.67 min.

Spectroscopic data were in accordance with those previously reported in the literature.[2]

<u>10-(4-methoxyphenyl)-9,9-dimethyl-9,10-dihydroacridine-2,7-dicarbaldehyde, **2-*p***:</u>

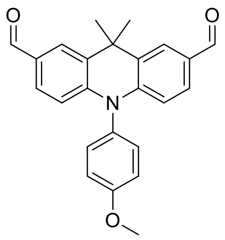

Chemical Formula: $C_{24}H_{21}NO_3$
Exact Mass: 371,1521

Under Ar atmosphere and at 0°C, $POCl_3$ (21.3 mL, 228 mmol, 100 eq.) was added dropwise to dry DMF (35 mL). After 1h stirring at 0°C, compound **1-*p*** (720 mg, 2.28 mmol, 1.0 eq.) was introduced. The solution was stirred at 95 °C for 72h. The dark mixture was poured into an ice bath and this solution was quenched by a 3M NaOH aqueous solution. The aqueous layer was extracted four times with AcOEt. Organic layers were combined, washed with brine and dried over Na_2SO_4. The crude was purified by flash chromatography on silica gel (Cyclohexane/AcOEt, 100:0 to 70:30) to give the expected product as a yellow solid (667 mg, 1.80 mmol, **η = 79 %**). **TLC**: R_f (Cyclohexane/AcOEt, 7:3) = 0.42; **Melting point:** 165 °C; **^{1}H NMR** (300 MHz, $CDCl_3$): δ = 9.86 (s, 2H), 8.01 (d, *J* = 1.5 Hz, 2H), 7.51 (dd, *J* = 8.5 Hz, 1.5 Hz, 2H), 7.22 (d, *J* = 9.0 Hz, 2H), 7.18 (d, *J* = 9.0 Hz, 2H), 6.45 (d, *J* = 8.5 Hz, 2H), 3.94 (s, 3H), 1.76 (s, 6H); **^{13}C NMR** (75 MHz, $CDCl_3$): δ = 190.9 (CH), 160.1 (C_q), 145.1 (C_q), 132.0 (C_q), 131.3 (CH), 130.7 (C_q), 129.6 (CH), 127.6 (CH), 116.6 (CH), 115.3 (CH), 55.8 (CH_3), 36.2 (C_q), 32.0 (CH_3); **LC/MS** (ES+): *m/z* = 372.3 ($[M+H]^+$), tr = 7.30 min ; **HRMS** (ES+): *m/z* calculated for $C_{24}H_{22}NO_3^+$: 372.1594, found: 372.1613.

[2] Han,M.; Xu,Z; Lu, J.; Xie, Y.; Li, Q.; Li, Z. Intramolecular-locked triphenylamine derivatives with adjustable room temperature phosphorescence properties by the substituent effect *Mater. Chem. Front.*, **2022**, 6, 33–39

10-(4-hydroxyphenyl)-9,9-dimethyl-9,10-dihydroacridine-2,7-dicarbaldehyde, **3-*p*:**

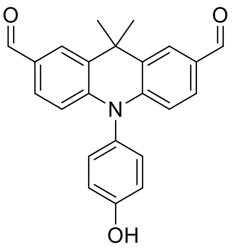

Chemical Formula: $C_{23}H_{19}NO_3$
Exact Mass: 357,1365

At 0°C and under Ar atmosphere, ether **2-*p*** (665 mg, 1.79 mmol, 1.0 eq.) was dissolved in dry DCM (12 mL) and BBr_3 (0.85 mL, 8.95 mmol, 5.0 eq.) was added dropwise. The solution was then stirred for 3h. Water was introduced. The aqueous layer was extracted once with DCM. The organic layer was washed with brine and dried over Na_2SO_4. The crude was purified by flash chromatography on silica gel (Cyclohexane/AcOEt 90:10 to 50:50) to give the expected product as a yellow solid (485 mg, 8.88 mmol, **η = 76 %**). **TLC**: R_f (Cyclohexane/AcOEt, 1:1) = 0.53; **Melting point**: 217 °C ; **^{1}H NMR** (300 MHz, $CDCl_3$): δ = 9.86 (s, 2H), 8.01 (d, *J* = 2.0 Hz, 2H), 7.52 (dd, *J* = 8.5 Hz, 2.0 Hz, 2H), 7.19 (d, *J* = 9.0 Hz, 2H), 7.13 (d, *J* = 9.0 Hz, 2H), 6.46 (d, *J* = 8.5 Hz, 2H), 5.64 (s, 1H), 1.75 (s, 6H); **^{13}C NMR** (75 MHz, $CDCl_3$): δ = 191.1 (CH), 156.5 (C_q), 145.2 (C_q), 132.0 (C_q), 131.5 (CH), 130.8 (C_q), 130.7 (C_q), 129.7 (CH), 127.7 (CH), 118.2 (CH), 115.3 (CH), 36.2 (C_q), 32.0 (CH_3); **LC/MS** (ES+): *m/z* = 358.3 ($[M+H]^+$), tr = 6.64 min; **HRMS** (ES+): *m/z* calculated for $C_{23}H_{20}NO_3^+$: 358.1438, found: 358.1441.

9,9-dimethyl-10-{4-[(6-methyl-1,2,4,5-tetrazin-3yl)methoxy]phenyl}-9,10-dihydroacridine-2,7-dicarbaldehyde, **5-*p***:

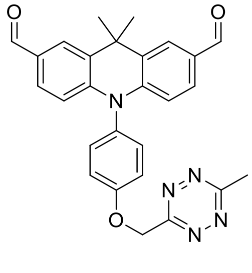

Chemical Formula: $C_{27}H_{23}N_5O_3$
Exact Mass: 465,1801

Under Ar atmosphere, phenol ***3-p*** (100 mg, 0.28 mmol, 1.0 eq.) and bromotetrazine **4** (178 mg, 0.84 mmol, 3.0 eq.) were dissolved in dry THF (4 mL). This solution was cooled to 0°C and Cs_2CO_3 (137 mg, 0.42 mmol, 1.5 eq.) was added. This solution was then stirred at r.t. for 4h. A saturated NH_4Cl aqueous solution was introduced. The aqueous layer was extracted three times with AcOEt. Organic layers were combined, washed with brine and dried over Na_2SO_4. The crude was purified by flash chromatography on silica gel (Cyclohexane/AcOEt, 100:0 to 50:50) to give the expected product as a red solid (62 mg, 0.13 mmol, **η = 48 %**). **TLC**: R_f (Cyclohexane/AcOEt, 1:1) = 0.53; **^{1}H NMR** (300 MHz, $CDCl_3$): δ = 9.87 (s, 2H), 8.01 (d, *J* = 1.5 Hz, 2H), 7.51 (dd, *J* = 8.5 Hz, 1.5 Hz, 2H), 7.36 (d, *J* = 9.0 Hz, 2H), 7.28 (d, *J* = 9.0 Hz, 2H), 6.43 (d, *J* = 8.5 Hz, 2H), 5.78 (s, 2H), 3.17 (s, 3H), 1.76 (s, 6H); **^{13}C NMR** (75 MHz, $CDCl_3$): δ = 190.9 (CH), 169.2 (C_q), 165.4 (C_q), 158.4 (C_q), 144.9 (C_q), 133.3 (C_q), 131.7 (CH), 130.9 (C_q), 130.8 (C_q), 129.7 (CH), 127.6 (CH), 117.6 (CH), 115.2 (CH), 68.2 (CH_2), 36.2

(C_q), 32.0 (CH_3), 21.6 (CH_3); **LC/MS** (ES+): *m/z* = 466.3 ($[M+H]^+$), tr = 7.11 min; **HRMS** (ES+): *m/z* calculated for $C_{27}H_{24}N_5O_3^+$: 466.1874, found: 466.1866.

Fluorogenic probe **Acri-*p*et**:

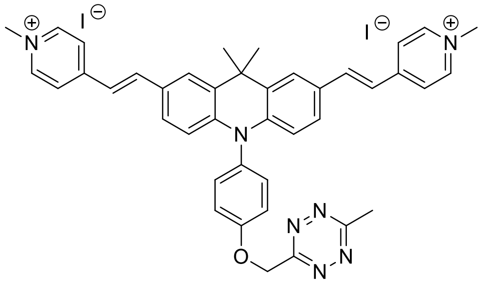

Chemical Formula: $C_{41}H_{39}N_7O^{2+}$
Exact Mass: 645,3205

Aldehyde **5-*p*** (25 mg, 54 µmol, 1.0 eq.) was solubilized in dry DMF (1 mL). This solution was cooled to 0°C and pyrrolidine (9.3 µL, 0.11 mmol, 2.1 eq.) was then added. After 15 min stirring, AcOH (31 µL, 0.54 mmol, 10 eq.) and 1,4-dimethylpyridinium iodide **6** (24 mg, 0.10 mmol, 1.9 eq.) were introduced. The resulting mixture was stirred at 4°C for 19h. Diethyl ether (3 mL) was added: a precipitate appeared. It was filtered and washed with diethyl ether to give the expected product as a red solid (22 mg, 24 µmol, **η = 51 %**). **^{1}H NMR** (300 MHz, DMSO-d_6): δ = 8.79 (d, *J* = 6.5 Hz, 4H, H_{12}), 8.13 (d, *J* = 6.5 Hz, 4H, H_{11}), 8.03 – 7.93 (m, 4H, H_1, H_9), 7.48 – 7.36 (m, 8H, H_2, H_4, H_5, H_{10}), 6.30 (d, *J* = 8.5 Hz, 2H, H_3), 5.85 (s, 2H, H_7), 4.22 (s, 6H, H_{13}), 3.05 (s, 3H, H_8), 1.78 (s, 6H, H_6); **^{13}C NMR** (75 MHz, DMSO-d_6): δ = 168.4 (C_q), 165.1 (C_q), 158.0 (C_q), 152.9 (C_q), 144.8 (CH), 141.5 (C_q), 140.9 (CH), 132.7 (C_q), 131.6 (CH), 130.4 (C_q), 128.4 (C_q), 127.5 (CH), 126.5 (CH), 122.8 (CH), 120.1 (CH), 117.5 (CH), 114.8 (CH), 67.7 (CH_2), 46.7 (CH_3), 35.8 (C_q), 32.3 (CH_3), 21.1 (CH_3); **LC/MS** (ES+): *m/z* = 322.9 ($[M\text{-}2I^-/2]^+$), tr = 5.48 min; **HRMS** (ES+): *m/z* calculated for $C_{41}H_{39}N_7OI^+$: 772.2255, found: 772.2257.

10-(2-bromophenyl)-9,9-dimethyl-9,10-dihydroacridine, **7-*o***:

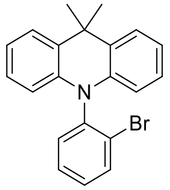

Chemical Formula: $C_{21}H_{18}BrN$
Exact Mass: 363,0623

Under Ar atmosphere, 9,9-dimethyl-9,10-dihydroacridine (100 mg, 0.48 mmol, 1.0 eq.), 2-bromoiodobenzene (0.25 mL, 1.91 mmol, 4.0 eq.), *t*-BuONa (92 mg, 0.96 mmol, 2.0 eq.), $Pd_2(dba)_3$ (9 mg, 10 µmol, 0.02 eq.) and XPhos (14 mg, 29 µmol, 0.06 eq.) were dissolved in freshly degassed (by Ar bubbling for 15 min) dry toluene (4.8 mL). The solution was stirred at 110 °C overnight. The crude was filtered through a Celite pad and purified by flash chromatography on silica gel (Cyclohexane) to give the expected product as a white solid (129 mg, 0.35 mmol, **η = 74 %**). **CAS number** [2142571-63-7]; **^{1}H NMR** (300 MHz, $CDCl_3$): δ = 7.89 (dd, *J* = 8.5, 1.5 Hz, 1H), 7.57 (td, *J* = 8.0, 1.5 Hz, 1H), 7.50 (dd, *J* = 7.5, 2.0 Hz, 2H), 7.44

– 7.36 (m, 2H), 7.03 – 6.92 (m, 4H), 6.13 (dd, *J* = 8.0, 1.5 Hz, 2H), 1.79 (s, 3H), 1.69 (s, 3H); **LC/MS** (ES+): *m/z* = 364.2 ($[M+H]^+$), tr = 8.03 min.

Spectroscopic data were in accordance with those previously reported in the literature.[3]

<u>10-(2-bromophenyl)-9,9-dimethyl-9,10-dihydroacridine-2,7-dicarbaldehyde, **8-*o***:</u>

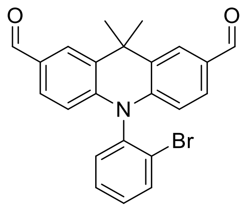

Chemical Formula: $C_{23}H_{18}BrNO_2$
Exact Mass: 419,0521

Under Ar atmosphere and at 0°C, $POCl_3$ (2.69 mL, 28.8 mmol, 70 eq.) was added dropwise to dry DMF (5 mL). After 1h stirring at 0°C, compound **7-*o*** (150 mg, 0.41 mmol, 1.0 eq.) was introduced. The solution was stirred at 95 °C overnight. The dark mixture was poured into an ice bath and this solution was quenched by NaOH 3M. The aqueous layer was extracted three times with AcOEt. Organic layers were combined, washed with brine and dried over Na_2SO_4. The crude was purified by flash chromatography on silica gel (Cyclohexane/AcOEt, 100:0 to 70:30) to give the expected product as a yellow solid (97 mg, 0.23 mmol, **η = 56 %**). **TLC**: R_f (Cyclohexane/AcOEt, 7:3) = 0.49 ; **Melting point**: 185 °C ; **^{1}H NMR** (300 MHz, $CDCl_3$): δ = 9.87 (s, 2H), 8.05 (d, *J* = 1.5 Hz, 2H), 7.93 (dd, *J* = 8.0 Hz, 1.0 Hz, 1H), 7.65 (td, *J* = 7.5 Hz, 1.0 Hz, 1H), 7.56 – 7.47 (m, 3H), 7.41 (dd, *J* = 8.0 Hz, 1.5 Hz, 1H), 6.26 (d, *J* = 8.5 Hz, 2H), 1.84 (s, 3H), 1.76 (s, 3H); **^{13}C NMR** (75 MHz, $CDCl_3$): δ = 190.8 (CH), 143.2 (C_q), 138.2 (C_q), 135.5 (CH), 132.5 (CH), 131.2 (CH), 131.1 (C_q), 130.7 (C_q), 130.4 (CH), 129.8 (CH), 128.0 (CH), 125.4 (C_q), 114.5 (CH), 36.7 (C_q), 33.8 (CH_3), 31.8 (CH_3); **LC/MS** (ES+): *m/z* = 422.4 ($[M+H]^+$), tr = 7.30 min; **HRMS** (ES+): *m/z* calculated for $C_{23}H_{19}BrNO_2^+$: 420.0594, found 420.0612.

<u>3-(2-methanesulfonylethyl)-6-methyl-1,2,4,5-tetrazine, **9**:</u>

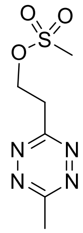

Chemical Formula: $C_6H_{10}N_4O_3S$
Exact Mass: 218,0474

Under Ar atmosphere, a solution of 3-hydroxypropionitrile (1.5 mL, 22.0 mmol, 1.0 eq.), ACN (9.23 mL, 176 mmol, 8.0 eq.), EtOH (3.77 mL, 64.7 mmol, 3.0 eq.) and 3-mercaptopropionic acid (1.91 mL, 22.0 mmol, 1.0 eq) was cooled to 0°C.

[3] WO2018038938 (Spiroacridine derivatives useful as oleds)

Hydrazine hydrate (9.23 mL, 263 mmol, 12.0 eq.) was then added dropwise. The solution was then stirred at r.t. for 16h. A saturated aqueous solution of sodium nitrite (12.8 g, 329 mmol, 15 eq.) was added into the reaction mixture, which was cooled to 0°C. A solution of HCl 12M was then added dropwise until gas release ceased. The pink aqueous layer was extracted once with DCM. The organic layer was washed with brine. Et_3N (3.27 mL, 23.6 mmol, 1.1 eq.) and methanesulfonyl chloride (1.82 mL, 23.6 mmol, 1.1 eq.) were added and the solution was stirred at r.t. for 10 min. The organic layer was washed once with water. The crude was purified by flash chromatography on silica gel (Cyclohexane/AcOEt, 90:10 to 50:50) to give the expected product as a pink solid (970 mg, 4.44 mmol, **η = 21 %**). **CAS number** [1616736-04-9]; **TLC**: R_f (Cyclohexane/AcOEt, 1:1) = 0.27; **^{1}H NMR** (300 MHz, $CDCl_3$): δ = 4.87 (t, *J* = 6.0 Hz, 2H), 3.77 (t, *J* = 6.0 Hz, 2H), 3.08 (s, 3H), 3.02 (s, 3H); **LC/MS** (ES+): *m/z* = 219.2 ($[M+H]^+$), tr = 4.53 min.

Spectroscopic data were in accordance with those previously reported in the literature.[4]

<u>9,9-dimethyl-10-{2-[(E)-2-(6-methyl-1,2,4,5-tetrazin-3-yl)ethenyl]phenyl}-9,10-dihydroacridine-2,7-dicarbaldehyde, **10-*o***:</u>

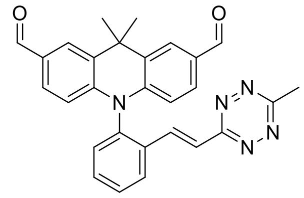

Chemical Formula: $C_{28}H_{23}N_5O_2$
Exact Mass: 461,1852

Under Ar atmosphere, bromine derivative **8-*o*** (60 mg, 0.14 mmol, 1.0 eq.), tetrazine **9** (39 mg, 0.18 mmol, 1.25 eq.), $Pd_2(dba)_3$ (7 mg, 7 µmol, 0.05 eq.) and QPhos (5 mg, 7 µmol, 0.05 eq.) were solubilized in dry DMF (1 mL). The solution was degassed for 5 min, *N,N*-dicyclohexylmethylamine (76 µL, 0.36 mmol, 2.5 eq.) was then added. The solution was stirred at 100 °C for 1h30. AcOEt was introduced. The organic layer was washed with brine and dried over Na_2SO_4. The crude was purified by preparative TLC (Thickness: 2 mm, Cyclohexane/AcOEt 60:40) to give the expected product as a red solid (41 mg, 89 µmol, **η = 62 %**). **TLC**: R_f (Cyclohexane/AcOEt, 7:3) = 0.26; **^{1}H NMR** (300 MHz, $CDCl_3$): δ = 9.85 (s, 2H), 8.20 – 8.16 (m, 1H), 8.11 (d, *J* = 16.5 Hz, 1H), 8.05 (d, *J* = 1.5 Hz, 2H), 7.77 – 7.71 (m, 2H), 7.52 – 7.46 (m, 3H), 7.39 – 7.34 (m, 1H), 6.31 (d, *J* = 8.5 Hz, 2H), 2.95 (s, 3H), 1.88 (s, 3H), 1.82 (s, 3H); **^{13}C NMR** (75 MHz, $CDCl_3$): δ = 190.8 (CH), 166.7 (C_q), 164.2 (C_q), 144.0 (C_q), 138.8 (C_q), 135.3 (C_q), 134.3 (CH), 133.0 (CH), 131.6 (CH), 131.1 (C_q), 130.9 (C_q), 130.4 (CH), 129.8 (CH), 128.9 (CH), 128.2 (CH), 124.5 (CH), 115.0 (CH), 36.3 (C_q), 34.1 (CH_3), 31.8 (CH_3), 21.3 (CH_3); **LC/MS** (ES+): *m/z* = 462.4 ($[M+H]^+$), tr = 7.30 min. **HRMS** (ES+): *m/z* calculated for $C_{28}H_{24}N_5O_2^+$: 462.1925, found: 462.1925.

[4] Kozma, E.; Estrada Girona, G.; Paci, G.; Lemke, E. A.; Kele, P., Bioorthogonal double-fluorogenic siliconrhodamine probes for intracellular super-resolution microscopy. *Chemical Communications* **2017,** *53* (50), 6696-6699.

Fluorogenic probe **Acri-*o*vi**:

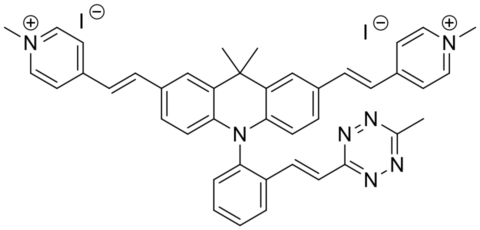

Chemical Formula: $C_{42}H_{39}N_7^{2+}$
Exact Mass: 641,3256

Aldehyde **10-*o*** (25 mg, 54 µmol, 1.0 eq.) was solubilized in dry DMF (1 mL). This solution was cooled to 0°C and pyrrolidine (9.3 µL, 0.11 mmol, 2.1 eq.) was then added. After 15 min stirring, AcOH (31 µL, 0.54 mmol, 10 eq.) and 1,4-dimethylpyridinium iodide **6** (24 mg, 0.10 mmol, 1.9 eq.) were introduced. The resulting mixture was stirred at 4°C for 17h. Diethyl ether (3 mL) was added: a precipitate appeared. It was filtered and washed with diethyl ether to give the expected product as a red solid (31 mg, 35 µmol, **η = 64 %**). **^{1}H NMR** (300 MHz, DMSO-d_6): δ = 8.78 (d, *J* = 6.5 Hz, 4H), 8.53 (dd, *J* = 7.5 Hz, 1.5 Hz, 1H), 8.12 (d, *J* = 6.5 Hz, 4H), 8.05 – 7.90 (m, 5H), 7.88 – 7,73 (m, 3H), 7.54 (dd, *J* = 7.5 Hz, 1.5 Hz, 1H), 7.42 – 7.37 (m, 4H), 6.17 (d, *J* = 8.5 Hz, 2H), 4.21 (s, 6H), 2.82 (s, 3H), 1.91 (s, 3H), 1.84 (s, 3H); **^{13}C NMR** (75 MHz, DMSO-d_6): δ = 166.3 (C_q), 163.7 (C_q), 152.9 (C_q), 144.8 (CH), 140.6 (CH), 140.5 (C_q), 138.4 (C_q), 134.2 (C_q), 133.2 (CH), 132.8 (CH), 131.6 (CH), 130.3 (C_q), 130.2 (CH), 129.2 (CH), 128.7 (C_q), 127.9 (CH), 127.1 (CH), 124.1 (CH), 122.8 (CH), 120.4 (CH), 114.6 (CH), 46.7 (CH_3), 35.9 (C_q), 34.5 (CH_3), 32.2 (CH_3), 20.8 (CH_3); **LC/MS** (ES+): *m/z* = 320.9 ([M-2I$^-$/2]$^+$), tr = 6.10 min; **HRMS** (ES+): *m/z* calculated for $C_{42}H_{39}N_7I^+$: 768.2306, found: 768.2289.

10-(3-bromophenyl)-9,9-dimethyl-9,10-dihydroacridine, **7-*m***:

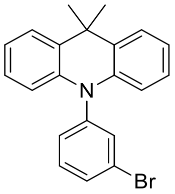

Chemical Formula: $C_{21}H_{18}BrN$
Exact Mass: 363,0623

Under Ar atmosphere, 9,9-dimethyl-9,10-dihydroacridine (300 mg, 1.43 mmol, 1.0 eq.), 3-bromoiodobenzene (0.55 mL, 4.30 mmol, 3.0 eq.), *t*-BuONa (275 mg, 2.87 mmol, 2.0 eq.), $Pd_2(dba)_3$ (26 mg, 29 µmol, 0.02 eq.) and XPhos (41 mg, 86 µmol, 0.06 eq.) were dissolved in freshly degassed (by Ar bubbling for 15 min) dry toluene (13.5 mL). The solution was stirred at 110 °C overnight. The crude was filtered through a Celite pad and purified by flash chromatography on silica gel to give the expected product as a white solid (197 mg, 0.54 mmol, **η = 38 %**). **TLC**: R_f (Cyclohexane) = 0.35; **Melting point**: 156 °C; **^{1}H NMR** (300 MHz, $CDCl_3$): δ = 7.63 – 7.61 (m, 1H), 7.54 – 7.41 (m, 4H), 7.30 – 7.27 (m, 1H), 7.00 – 6.89 (m, 4H), 6.25 (dd, *J* = 8.0 Hz, 1.5 Hz, 2H), 1.67 (s, 6H); **^{13}C NMR** (75 MHz, $CDCl_3$): δ = 142.7 (C_q), 140.6 (C_q), 134.7 (CH), 132.2 (CH),

131.6 (CH), 130.4 (CH), 130.2 (C_q), 126.6 (CH), 125.4 (CH), 124.0 (C_q), 121.0 (CH), 114.1 (CH), 36.1 (C_q), 31.4 (CH_3); **LC/MS** (ES+): *m/z* = 366.3 ($[M+H]^+$), tr = 8.48 min; **HRMS** (ES+): *m/z* calculated for $C_{21}H_{19}BrN^+$: 364.0695, found: 364.0693.

10-(3-bromophenyl)-9,9-dimethyl-9,10-dihydroacridine-2,7-dicarbaldehyde, **8-*m***:

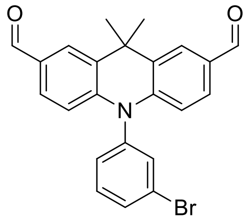

Chemical Formula: $C_{23}H_{18}BrNO_2$
Exact Mass: 419,0521

Under Ar atmosphere and at 0°C, $POCl_3$ (12.5 mL, 134 mmol, 250 eq.) was added dropwise to dry DMF (20 mL). After 1h stirring at 0°C, 10-(3-bromophenyl)-9,9-dimethyl-9,10-dihydroacridine **7-*m*** (197 mg, 0.54 mmol, 1.0 eq.) was introduced. The solution was stirred at 95 °C overnight. The dark mixture was poured into an ice bath and this solution was quenched by NaOH 3M. The aqueous layer was extracted three times with AcOEt. Organic layers were combined, washed with brine and dried over Na_2SO_4. The crude was purified by flash chromatography on silica gel (Cyclohexane/AcOEt, 100:0 to 70:30) to give the expected product as a yellow solid (179 mg, 0.44 mmol, **η = 82 %**). **TLC**: R_f (Cyclohexane/AcOEt, 7:3) = 0.51; **Melting point**: 188 °C; **^{1}H NMR** (300 MHz, $CDCl_3$): δ = 9.87 (s, 2H), 8.03 (d, *J* = 1.5 Hz, 2H), 7.80 – 7.74 (m, 1H), 7.61 (t, *J* = 8.0 Hz, 1H), 7.56 – 7.50 (m, 3H), 7.33 (m, 1H), 6.40 (d, *J* = 8.5 Hz, 2H), 1.77 (s, 6H); **^{13}C NMR** (75 MHz, $CDCl_3$): δ = 190.7 (CH), 144.2 (C_q), 140.9 (C_q), 133.6 (CH), 132.8 (CH), 132.7 (CH), 131.0 (C_q), 130.7 (C_q), 129.7 (CH), 129.3 (CH), 127.6 (CH), 124.6 (C_q), 115.0 (CH), 36.2 (C_q), 32.0 (CH_3); **LC/MS** (ES+): *m/z* = 422.2 ($[M+H]^+$), tr = 8.05 min; **HRMS** (ES+): *m/z* calculated for $C_{23}H_{19}BrNO_2^+$: 420.0594, found: 420.0613.

9,9-dimethyl-10-{3-[(E)-2-(6-methyl-1,2,4,5-tetrazin-3-yl)ethenyl]phenyl}-9,10-dihydroacridine-2,7-dicarbaldehyde, **10-*m***:

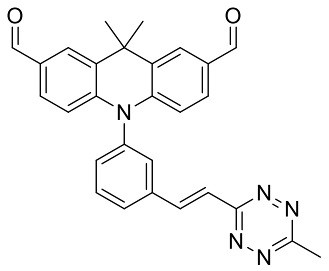

Chemical Formula: $C_{28}H_{23}N_5O_2$
Exact Mass: 461,1852

Under Ar atmosphere, bromine derivative **8-*m*** (115 mg, 0.59 mmol, 1.0 eq.), tetrazine **9** (75 mg, 0.34 mmol, 1.25 eq.), $Pd_2(dba)_3$ (13 mg, 14 µmol, 0.05 eq.) and QPhos (10 mg, 14 µmol, 0.05 eq.) were solubilized in dry DMF (2 mL). The solution was degassed for 5 min, *N,N*-dicyclohexylmethylamine (150 µL, 0.68 mmol, 2.5 eq.) was then added. The solution was stirred at 100 °C for 1h. AcOEt was introduced. The organic layer was washed with brine and dried over Na_2SO_4. During

evaporation, a precipitate appeared. It was filtered and washed with Et_2O to give the expected product as a red solid (103 mg, 0.22 mmol, **η = 82 %**). **TLC**: R_f (Cyclohexane/AcOEt, 1:1) = 0.67; **^{1}H NMR** (300 MHz, $CDCl_3$): δ = 9.88 (s, 2H), 8.37 (d, *J* = 16.5 Hz, 1H), 8.05 (d, *J* = 1.5 Hz, 2H), 7.93 (dd, *J* = 8.0 Hz, 0.5 Hz, 1H), 7.80 (t, *J* = 7.9 Hz, 1H), 7.66 (s, 1H), 7.57 – 7.49 (m, 3H), 7.43 – 7.38 (m, 1H), 6.44 (d, *J* = 8.5 Hz), 3.07 (s, 3H), 1.80 (s, 6H); **^{13}C NMR** (75 MHz, $CDCl_3$): δ = 190.8 (CH), 166.8 (C_q), 164.5 (C_q), 144.5 (C_q), 140.5 (C_q), 139.0 (CH), 138.9 (C_q), 132.2 (CH), 132.0 (CH), 131.0 (C_q), 130.8 (C_q), 129.7 (CH), 129.0 (CH), 127.7 (CH), 122.7 (CH), 115.1 (CH), 36.3 (C_q), 32.1 (CH_3), 21.4 (CH_3); **LC/MS** (ES+): *m/z* = 462.2 ($[M+H]^+$), tr = 7.91 min; **HRMS** (ES+): *m/z* calculated for $C_{28}H_{24}N_5O_2^+$: 462.1925, found: 462.1913.

Fluorogenic probe **Acri-*m*vi**:

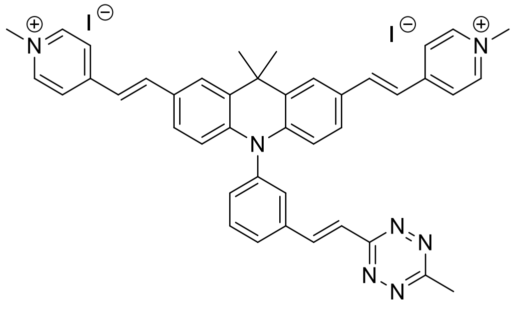

Chemical Formula: $C_{42}H_{39}N_7^{2+}$
Exact Mass: 641,3256

Compound **10-*m*** (30 mg, 65 µmol, 1.0 eq.) was solubilized in dry DMF (1,3 mL). This solution was cooled to 0°C and pyrrolidine (11 µL, 0.14 mmol, 2.1 eq.) was then added. After 15 min stirring, AcOH (37 µL, 0.65 mmol, 10 eq.) and 1,4-dimethylpyridinium iodide **6** (39 mg, 0.12 mmol, 1.9 eq.) were introduced. The resulting mixture was stirred at 4°C for 20h. Diethyl ether (3 mL) was added: a precipitate appeared. It was filtered and washed with diethyl ether to give the expected product as a red solid (30 mg, 33 µmol, **η = 58 %**). **^{1}H NMR** (300 MHz, DMSO-d_6): δ = 8.81 (d, *J* = 3.0 Hz, 4H), 8.35 (d, *J* = 16.5 Hz, 1H), 8.25 – 7.75 (m, 12H), 7.55 – 7.40 (m, 5H), 6.32 (d, *J* = 7.5 Hz, 2H), 4.22 (s, 6H), 2.96 (s, 3H), 1.82 (s, 6H); **^{13}C NMR** (75 MHz, DMSO-d_6): δ = 166.3 (C_q), 164.1 (C_q), 152.9 (C_q), 144.8 (CH), 141.2 (C_q), 140.9 (CH), 140.2 (C_q), 138.6 (C_q), 138.5 (CH), 132.1 (2 x CH), 130.4 (C_q), 130.0 (CH), 129.2 (CH), 128.5 (C_q), 127.6 (CH), 126.5 (CH), 122.9 (CH), 122.7 (CH), 120.2 (CH), 114.9 (CH), 46.7 (CH_3), 35.9 (C_q), 32.3 (CH_3), 20.9 (CH_3); **LC/MS** (ES+): *m/z* = 320.9 ($[M-2I^-/2]^+$), tr = 6.14 min; **HRMS** (ES+): *m/z* calculated for $C_{42}H_{39}N_7I^+$: 768.2306, found: 768.2326.

10-(4-bromophenyl)-9,9-dimethyl-9,10-dihydroacridine-2,7-dicarbaldehyde, **8-*p***:

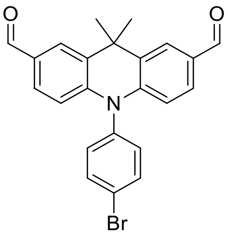

Chemical Formula: $C_{23}H_{18}BrNO_2$
Exact Mass: 419,0521

Under Ar atmosphere and at 0°C, $POCl_3$ (17.9 mL, 192 mmol, 100 eq.) was added dropwise to dry DMF (30 mL). After 1h stirring at 0°C, 10-(4-bromophenyl)-9,9-dimethyl-9,10-dihydroacridine **7-*p*** (700 mg, 1.92 mmol, 1.0 eq.) was introduced. The solution was stirred at 95 °C overnight. The dark mixture was poured into an ice bath and this solution was quenched by NaOH 3M. The aqueous layer was extracted four times with AcOEt. Organic layers were combined, washed with brine and dried over Na_2SO_4. The crude was purified by flash chromatography on silica gel (Cyclohexane/AcOEt, 100:0 to 80:20) to give the expected product as a yellow solid (666 mg, 1.58 mmol, **η = 82 %**). **TLC**: R_f (Cyclohexane/AcOEt, 7:3) = 0.55; **Melting point**: 191 °C; **^{1}H NMR** (300 MHz, $CDCl_3$): δ = 9.87 (s, 2H), 8.02 (d, *J* = 1.5 Hz, 2H), 7.84 (d, *J* = 8.5 Hz, 2H), 7.51 (dd, *J* = 8.5 Hz, 1.5 Hz, 2H), 7.23 (d, *J* = 8.5 Hz, 2H), 6.39 (d, *J* = 8.5 Hz, 2H), 1.76 (s, 6H); **^{13}C NMR** (75 MHz, $CDCl_3$): δ = 190.8 (CH), 144.4 (C_q), 138.6 (C_q), 134.9 (CH), 132.2 (CH), 131.0 (C_q), 130.8 (C_q), 129.7 (CH), 127.6 (CH), 123.6 (C_q), 115.0 (CH), 36.3 (C_q), 32.0 (CH_3); **LC/MS** (ES+): *m/z* = 422.2 ($[M+H]^+$), tr = 7.66 min; **HRMS** (ES+): *m/z* calculated for $C_{23}H_{19}BrNO_2^+$: 420.0594, found: 420.0589.

<u>9,9-dimethyl-10-{4-[(E)-2-(6-methyl-1,2,4,5-tetrazin-3-yl)ethenyl]phenyl}-9,10-dihydroacridine-2,7-dicarbaldehyde, **10-*p***:</u>

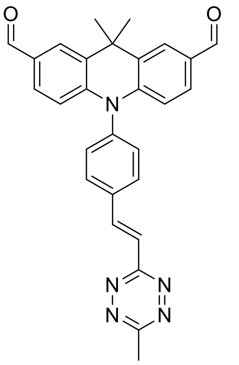

Chemical Formula: $C_{28}H_{23}N_5O_2$
Exact Mass: 461,1852

Under Ar atmosphere, bromine derivative **8-*p*** (250 mg, 0.59 mmol, 1.0 eq.), tetrazine **9** (162 mg, 0.74 mmol, 1.25 eq.), $Pd_2(dba)_3$ (27 mg, 30 µmol, 0.05 eq.) and QPhos (21 mg, 30 µmol, 0.05 eq.) were solubilized in dry DMF (4 mL). The solution was degassed for 5 min, *N,N*-dicyclohexylmethylamine (315 µL, 1.49 mmol, 2.5 eq.) was then added. The solution was stirred at 100 °C for 1h. AcOEt was introduced. The organic layer was washed with brine and dried over Na_2SO_4. During evaporation, a precipitate appeared. It was filtered and washed with Et_2O to give the expected product as a red solid (241 mg, 0.52 mmol, **η = 88 %**). **^{1}H NMR** (300 MHz, $CDCl_3$): δ = 9.88 (s, 2H), 8.43 (d, *J* = 16.5 Hz, 1H), 8.04 (d, *J* = 1.5 Hz, 2H), 8.01 (d, *J* = 8.5 Hz, 2H), 7.60 (d, *J* = 16.5 Hz, 1H), 7.53 (dd, *J* = 8.5 Hz, *J* = 1.5 Hz, 2H), 7.43 (d, *J* = 8.5 Hz, 2H), 6.45 (d, *J* = 8.5 Hz, 2H), 3.10 (s, 3H), 1.78 (s, 6H). **TLC**: R_f (Cyclohexane/AcOEt, 7:3) = 0.23; **^{13}C NMR** (75 MHz, $CDCl_3$): δ = 190.8 (CH), 166.9 (C_q), 164.6 (C_q), 144.4 (C_q), 141.0 (C_q), 139.2 (CH), 136.4 (C_q), 131.2 (CH), 131.0 (CH), 131.0 (C_q), 130.9 (C_q), 129.7 (CH), 127.7 (CH), 122.8 (CH), 115.1 (CH), 36.3 (C_q), 32.1 (CH_3), 21.4 (CH_3) .**LC/MS** (ES+): *m/z* = 462.3 ($[M+H]^+$), tr = 7.43 min. **HRMS** (ES+): *m/z* calculated for $C_{28}H_{24}N_5O_2^+$: 462.1925, found: 462.1909.

Fluorogenic probe **Acri-*p*vi**:

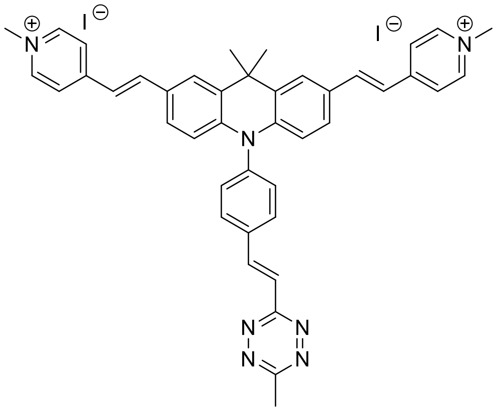

Chemical Formula: $C_{42}H_{39}N_7^{2+}$
Exact Mass: 641,3256

Aldehyde **10-*p*** (25 mg, 54 µmol, 1.0 eq.) was solubilized in dry DMF (1 mL). This solution was cooled to 0°C and pyrrolidine (9.3 µL, 0.11 mmol, 2.1 eq.) was then added. After 15 min stirring, AcOH (31 µL, 0.54 mmol, 10 eq.) and 1,4-dimethylpyridinium iodide **6** (24 mg, 0.10 mmol, 1.9 eq.) were introduced. The resulting mixture was stirred at 4°C for 16h. Diethyl ether (3 mL) was added: a precipitate appeared. It was filtered and washed with diethyl ether to give the expected product as a red solid (22 mg, 25 µmol, **η = 45 %**). **^{1}H NMR** (300 MHz, DMSO-d$_6$): δ = 8.80 (d, *J* = 6.5 Hz, 4H), 8.41 (d, *J* = 16.5 Hz, 1H), 8.27 (d, *J* = 8.5 Hz, 2H), 8.15 (d, *J* = 6.5 Hz, 4H), 8.07 – 7.95 (m, 4H), 7.81 (d, *J* = 16.5 Hz, 1H), 7.57 (d, *J* = 8.5 Hz, 2H), 7.49 – 7.36 (m, 4H), 6.33 (d, *J* = 8.5 Hz, 2H), 4.22 (s, 6H), 3.00 (s, 3H), 1.81 (s, 6H); **^{13}C NMR** (75 MHz, DMSO-d$_6$): δ = 166.4 (C$_q$), 164.1 (C$_q$), 152.9 (C$_q$), 144.8 (CH), 141.0 (C$_q$), 140.8 (CH), 140.7 (C$_q$), 138.6 (CH), 135.8 (C$_q$), 131.2 (CH), 131.1 (CH), 130.4 (C$_q$), 128.6 (C$_q$), 127.6 (CH), 126.6 (CH), 122.8 (CH), 122.6 (CH), 120.2 (CH), 114.8 (CH), 46.7 (CH$_3$), 35.9 (C$_q$), 32.3 (CH$_3$), 21.0 (CH$_3$); **LC/MS** (ES+): *m/z* = 320.9 ([M-2I$^-$/2]$^+$), tr = 6.35 min; **HRMS** (ES+): *m/z* calculated for $C_{42}H_{39}N_7I^+$ 768.2306, found: 768.2335.

Table S1. Optimization of the Knoevenagel reaction

Pyrrolidine trap	Results
HCl	No reaction
PTSA	No reaction
4-nitrophenylisocyanate	Product degradation
Trimethylsilylisocyanat	Product degradation
2-nitrobenzoic acid	Side products
AcOH	Less than 5% of degradation

III. NMR spectra of new compounds

^{1}H NMR spectra of 1-*o* in $CDCl_3$ (300 MHz):

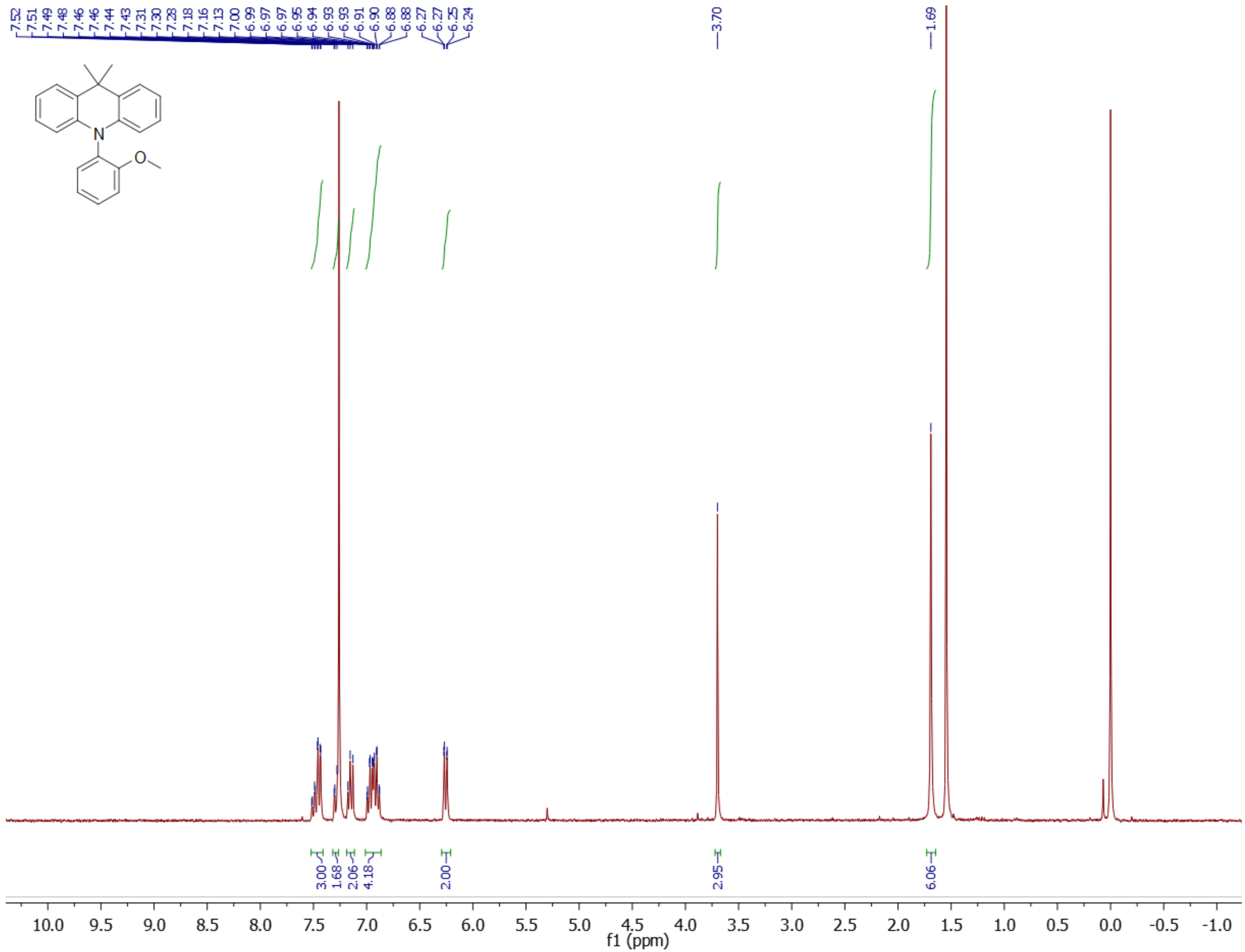

APT NMR spectra of 1-*o* in $CDCl_3$ (75 MHz):

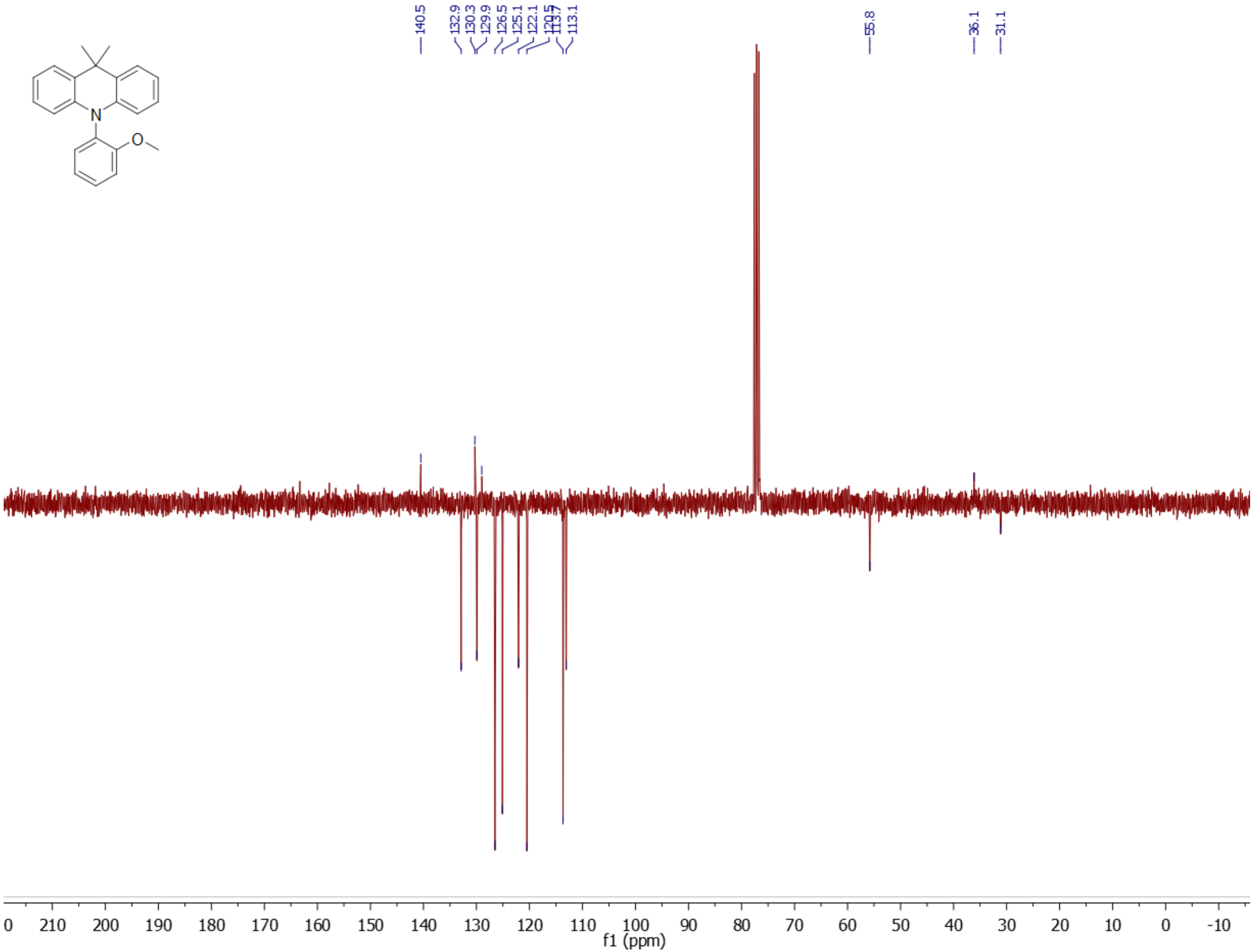

^{1}H NMR spectra of 2-*o* in $CDCl_3$ (300 MHz):

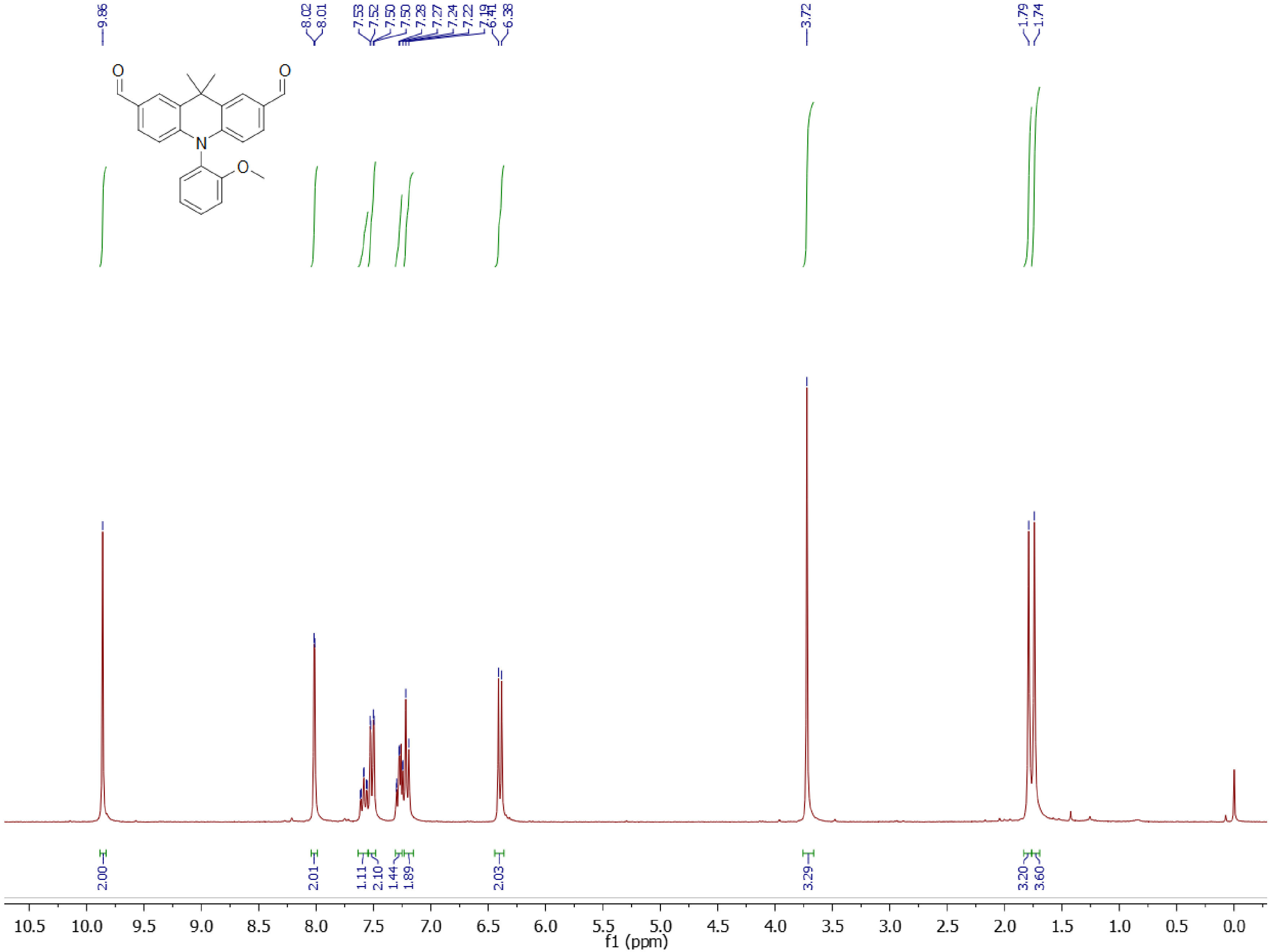

APT NMR spectra of 2-*o* in $CDCl_3$ (75 MHz):

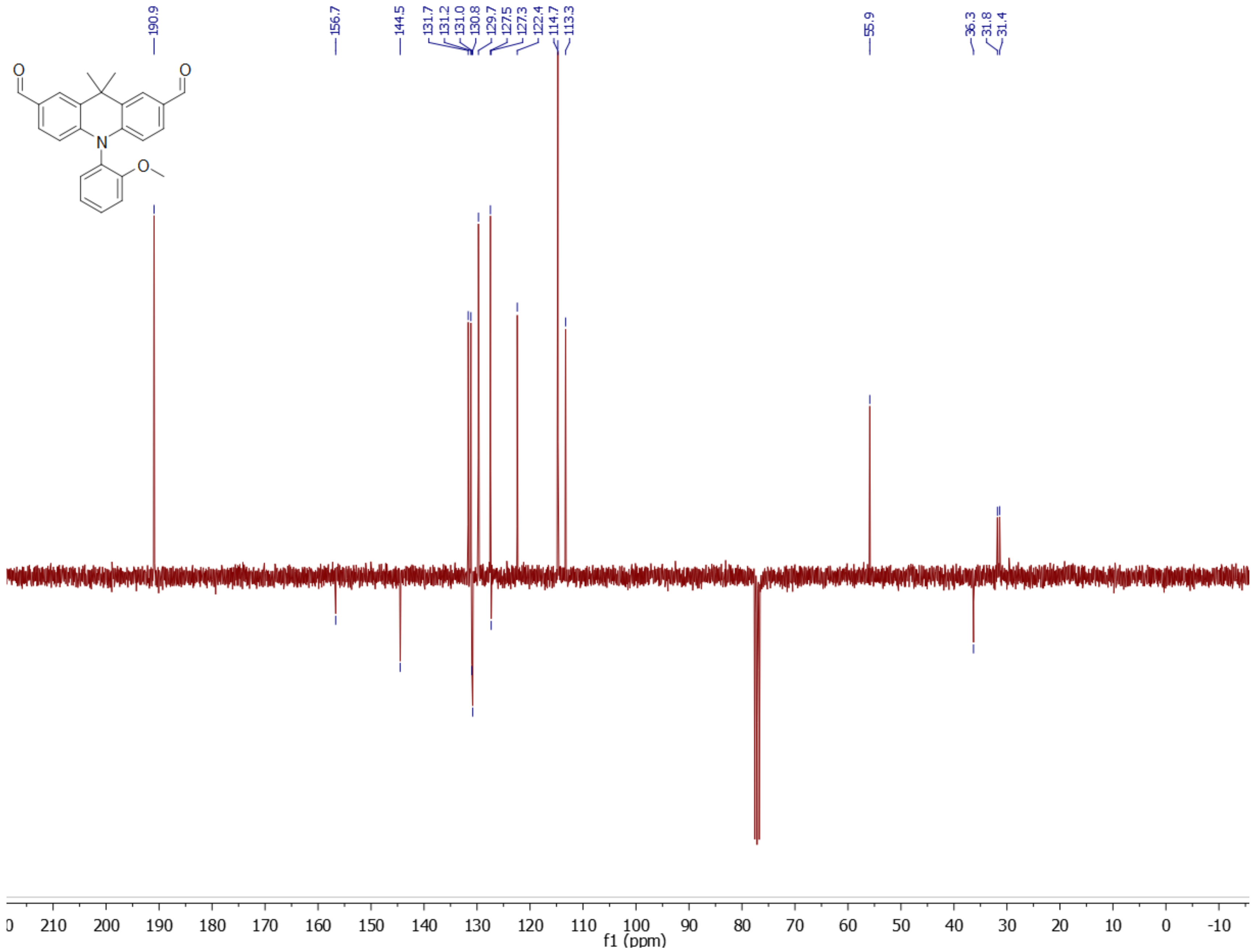

^{1}H NMR spectra of 3-*o* in $CDCl_3$ (300 MHz):

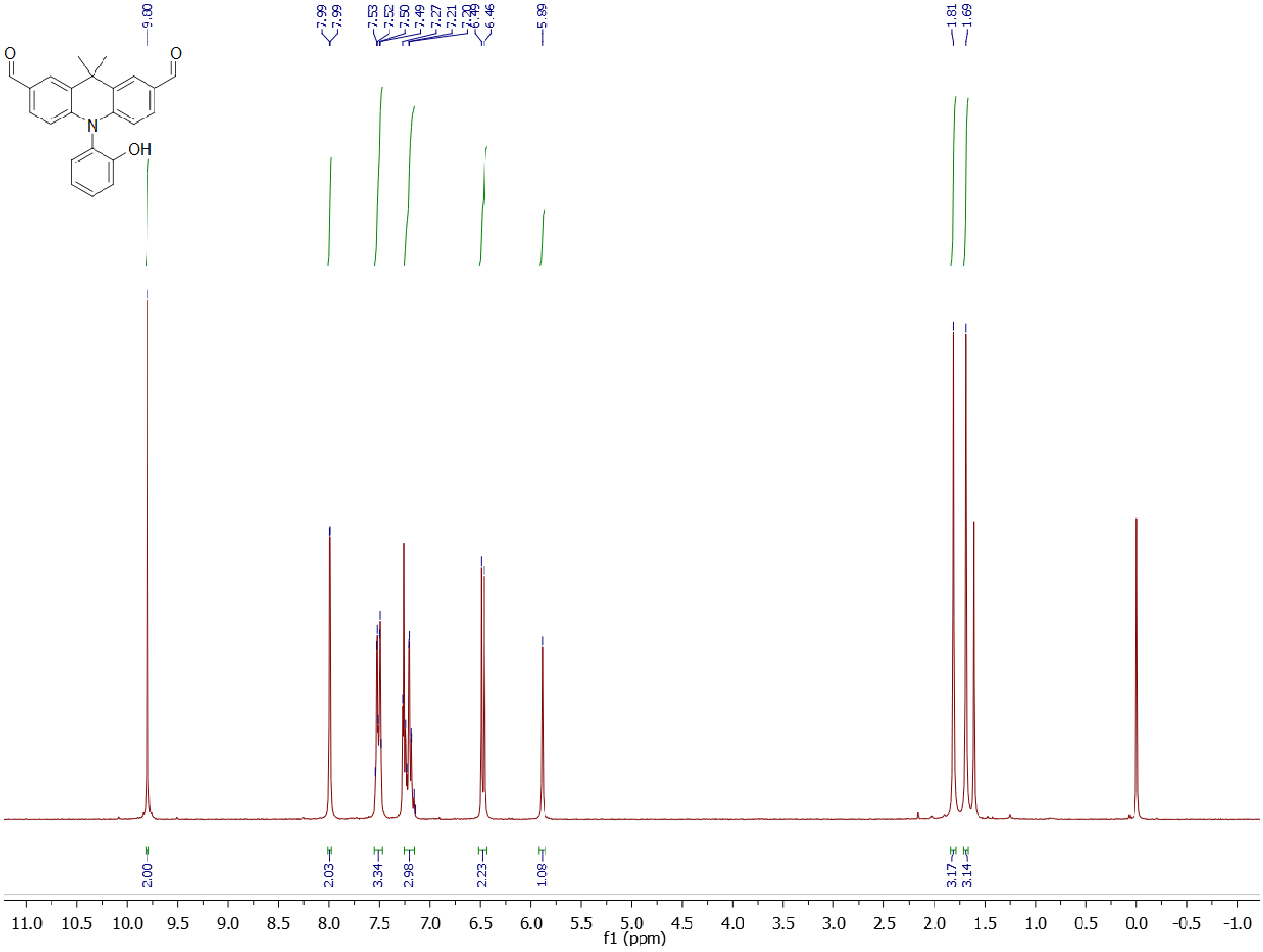

APT NMR spectra of 3-*o* in $CDCl_3$ (75 MHz):

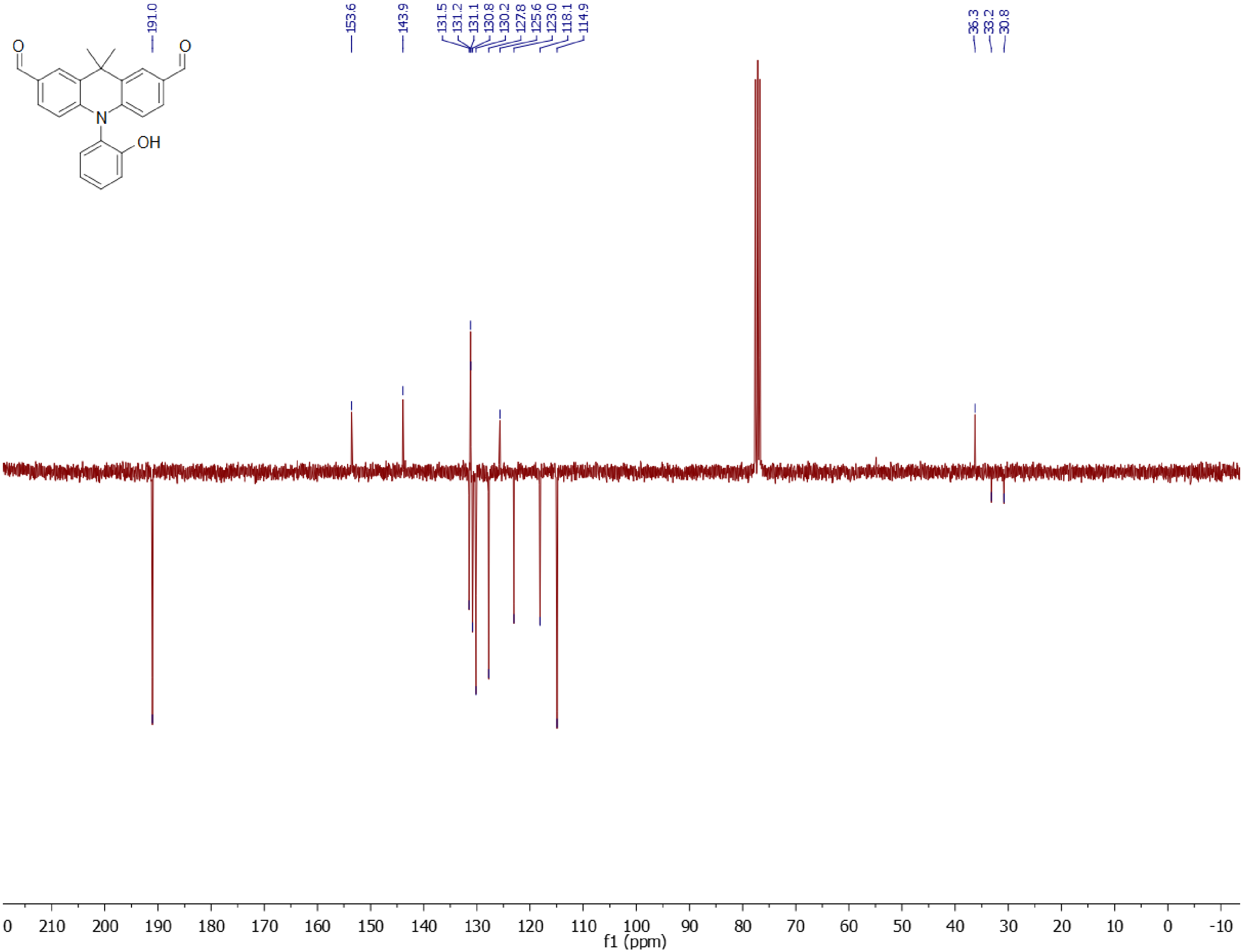

^{1}H NMR spectra of 5-*o* in $CDCl_3$ (300 MHz):

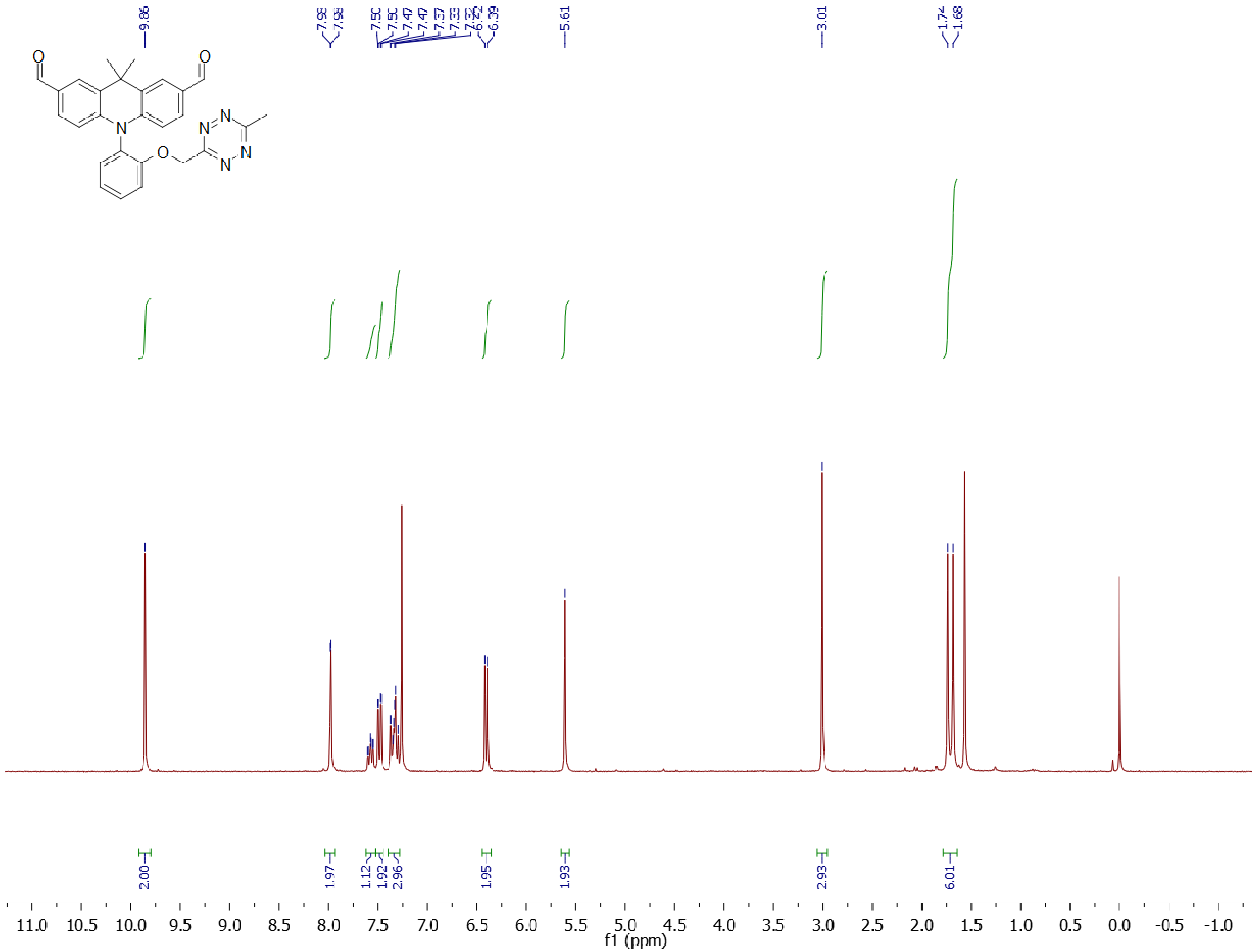

APT NMR spectra of 5-*o* in $CDCl_3$ (75 MHz):

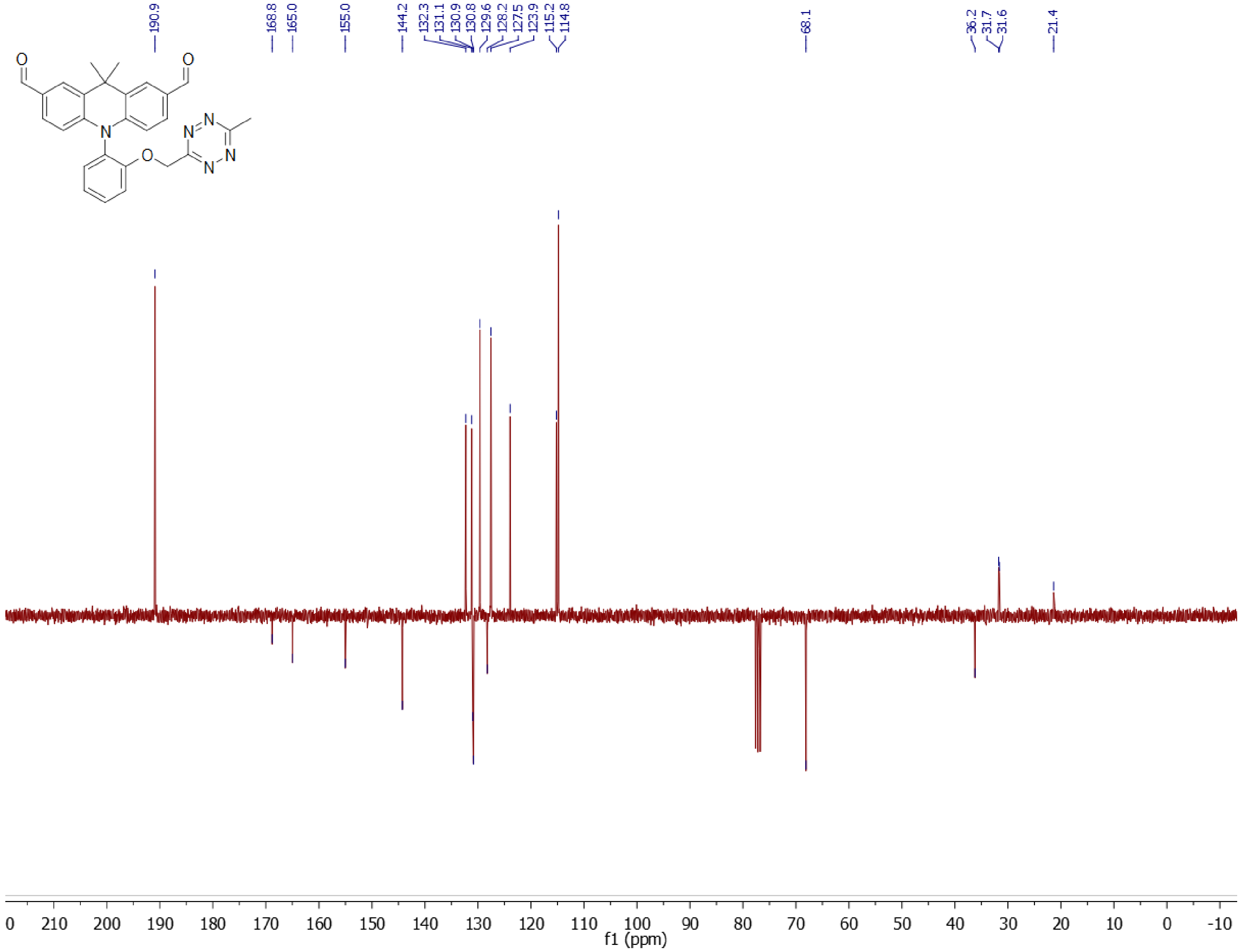

^{1}H NMR spectra of Acri-*o*et in DMSO-d_6 (300 MHz):

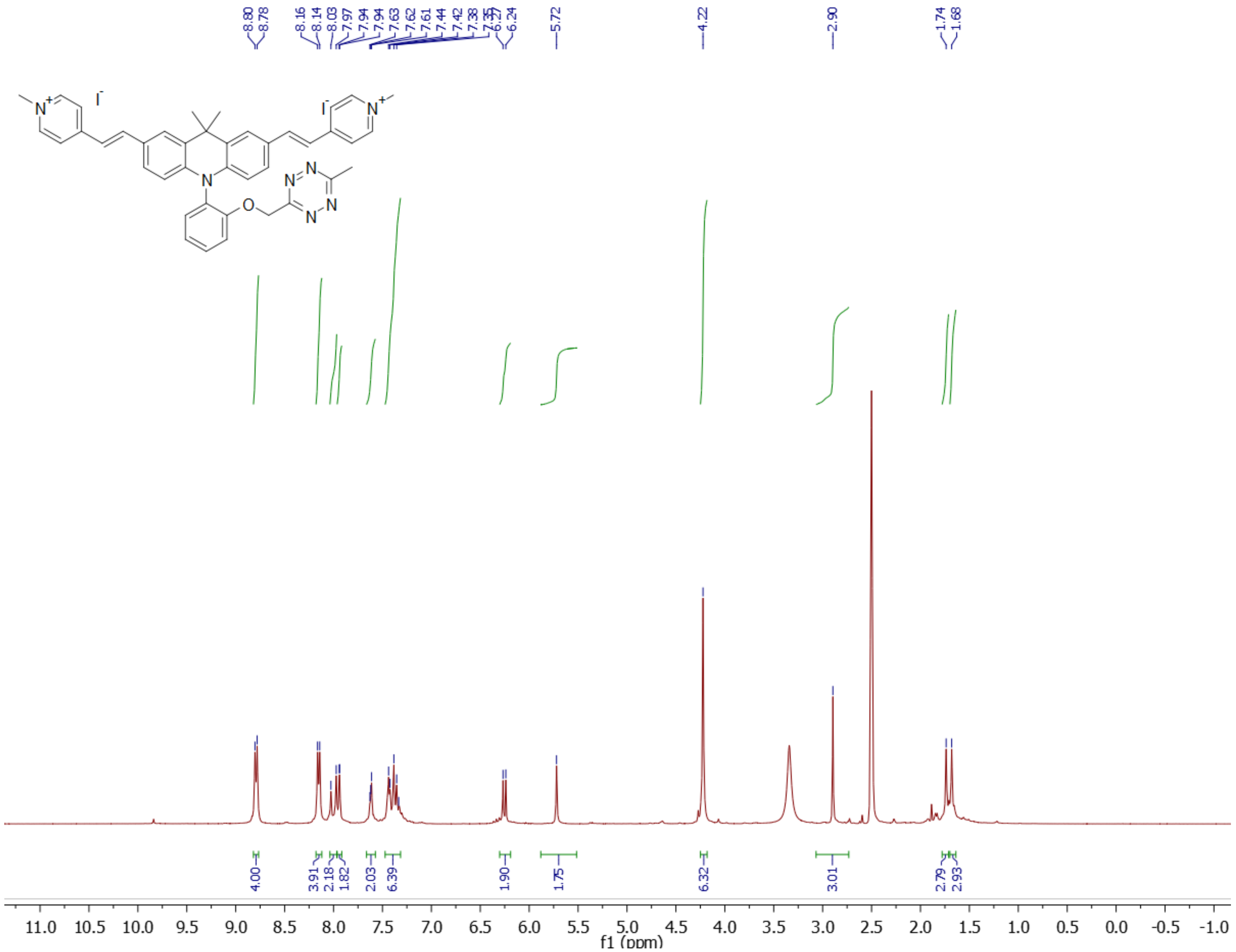

APT NMR spectra of Acri-*o*et in DMSO-d_6 (75 MHz):

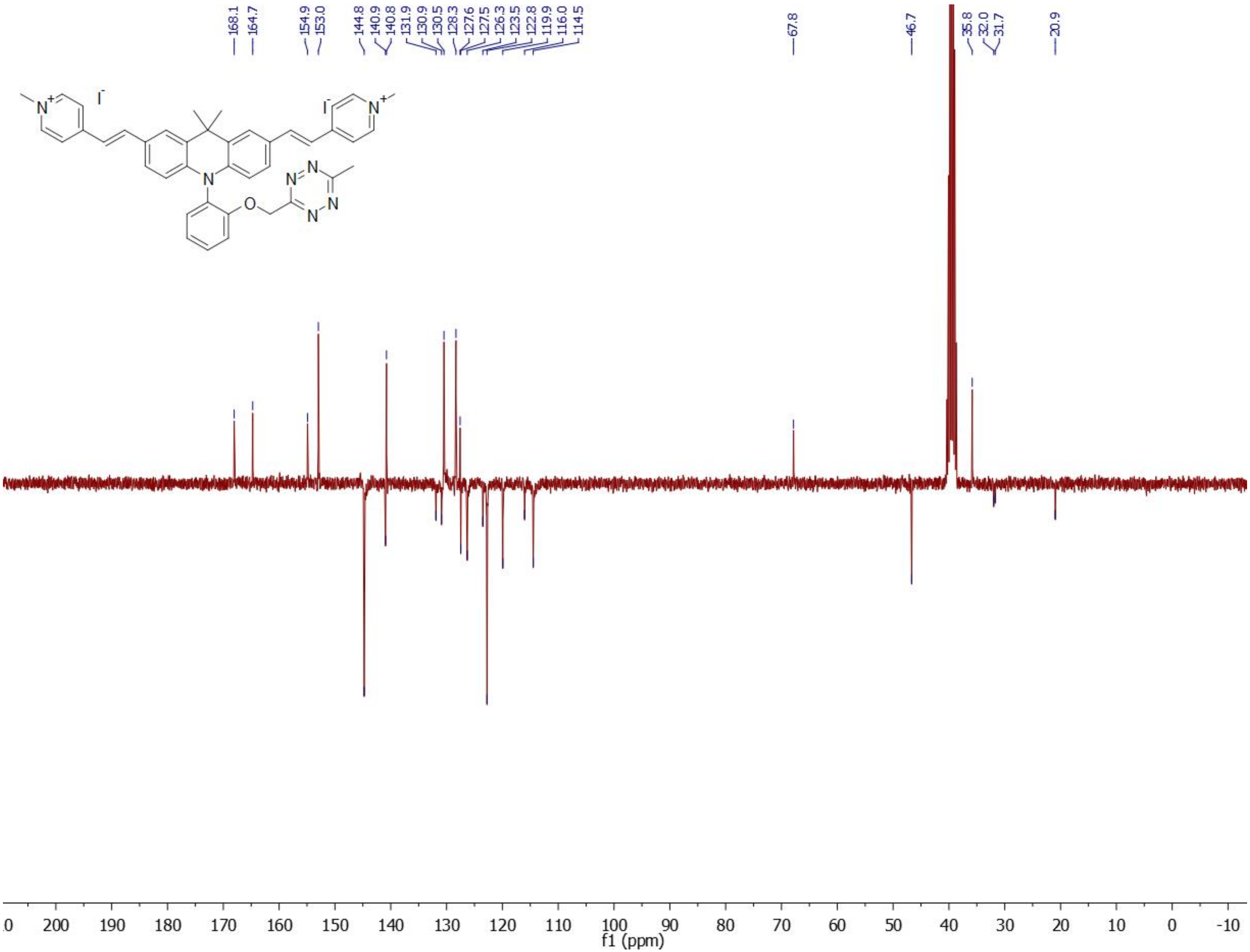

^{1}H NMR spectra of 1-*m* in $CDCl_3$ (300 MHz):

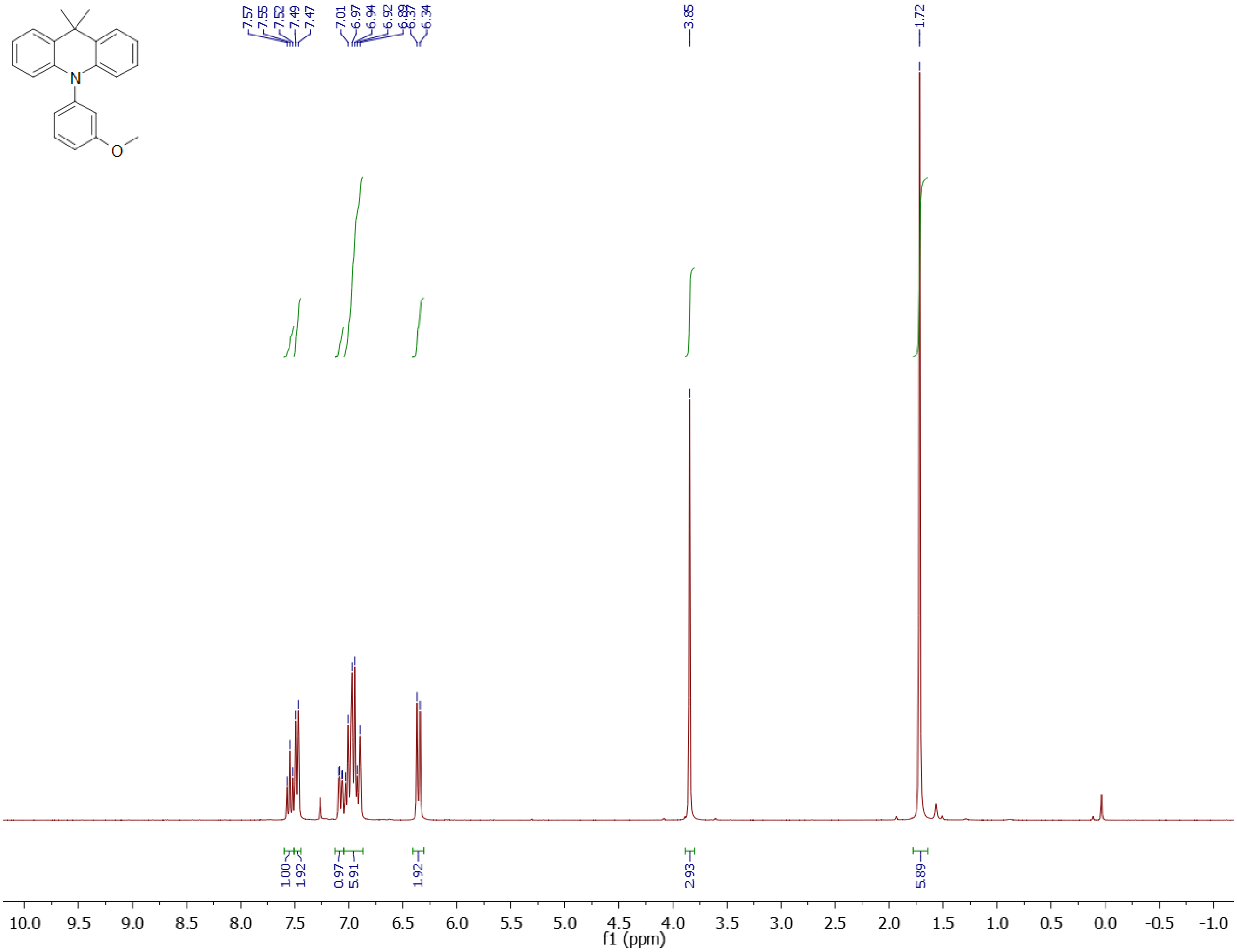

APT NMR spectra of 1-*m* in $CDCl_3$ (75 MHz):

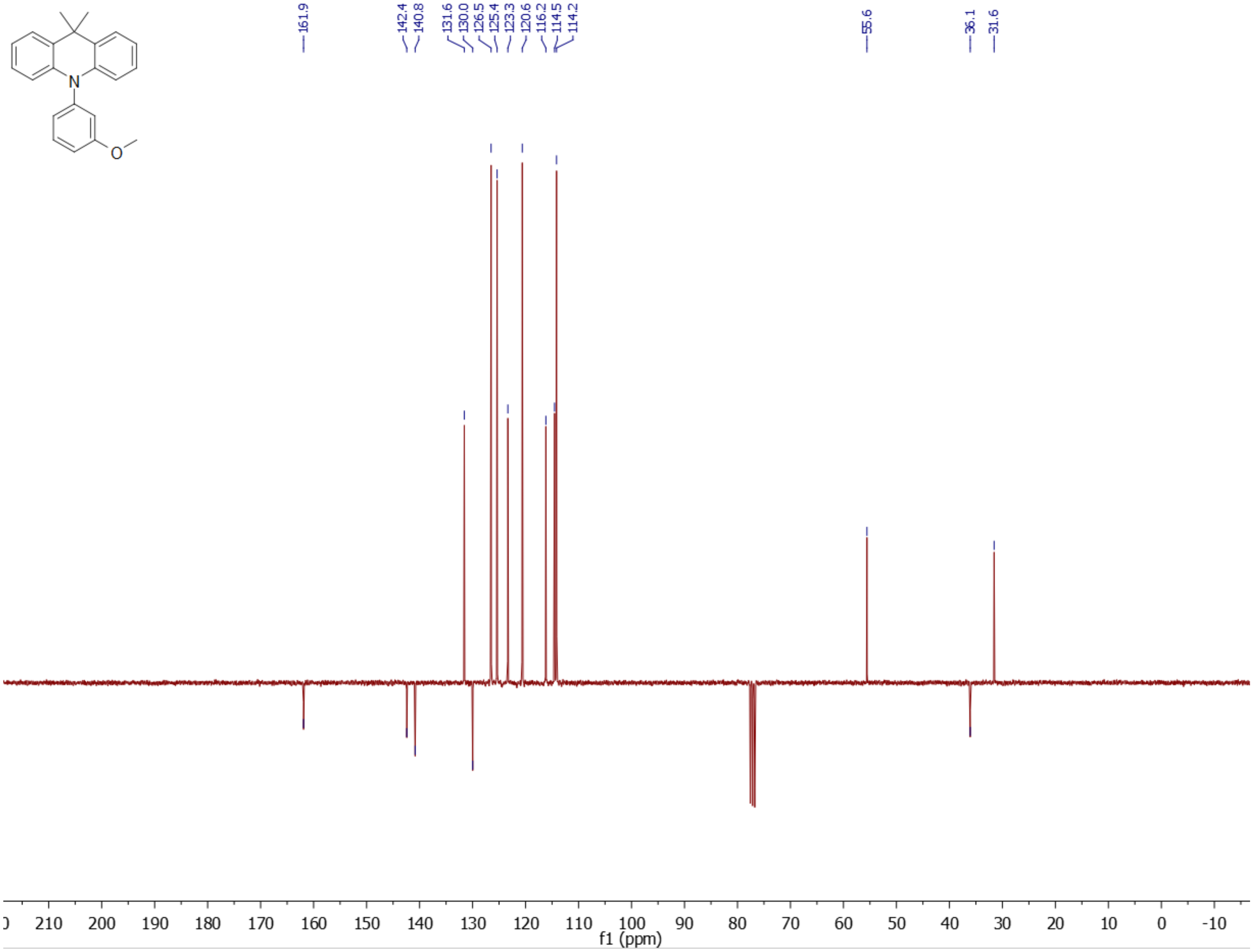

^{1}H NMR spectra of 2-*m* in $CDCl_3$ (300 MHz):

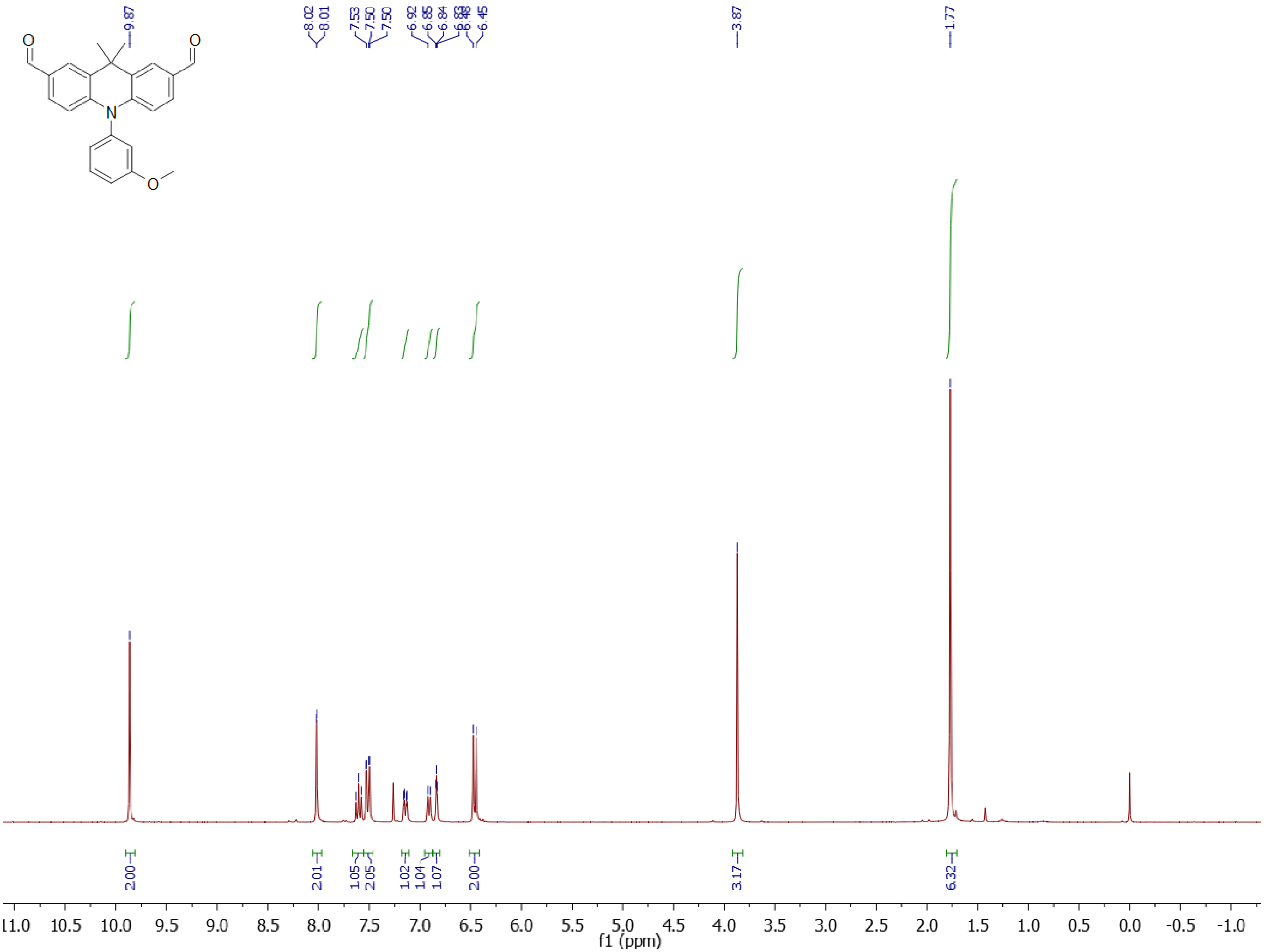

APT NMR spectra of 2-*m* in $CDCl_3$ (75 MHz):

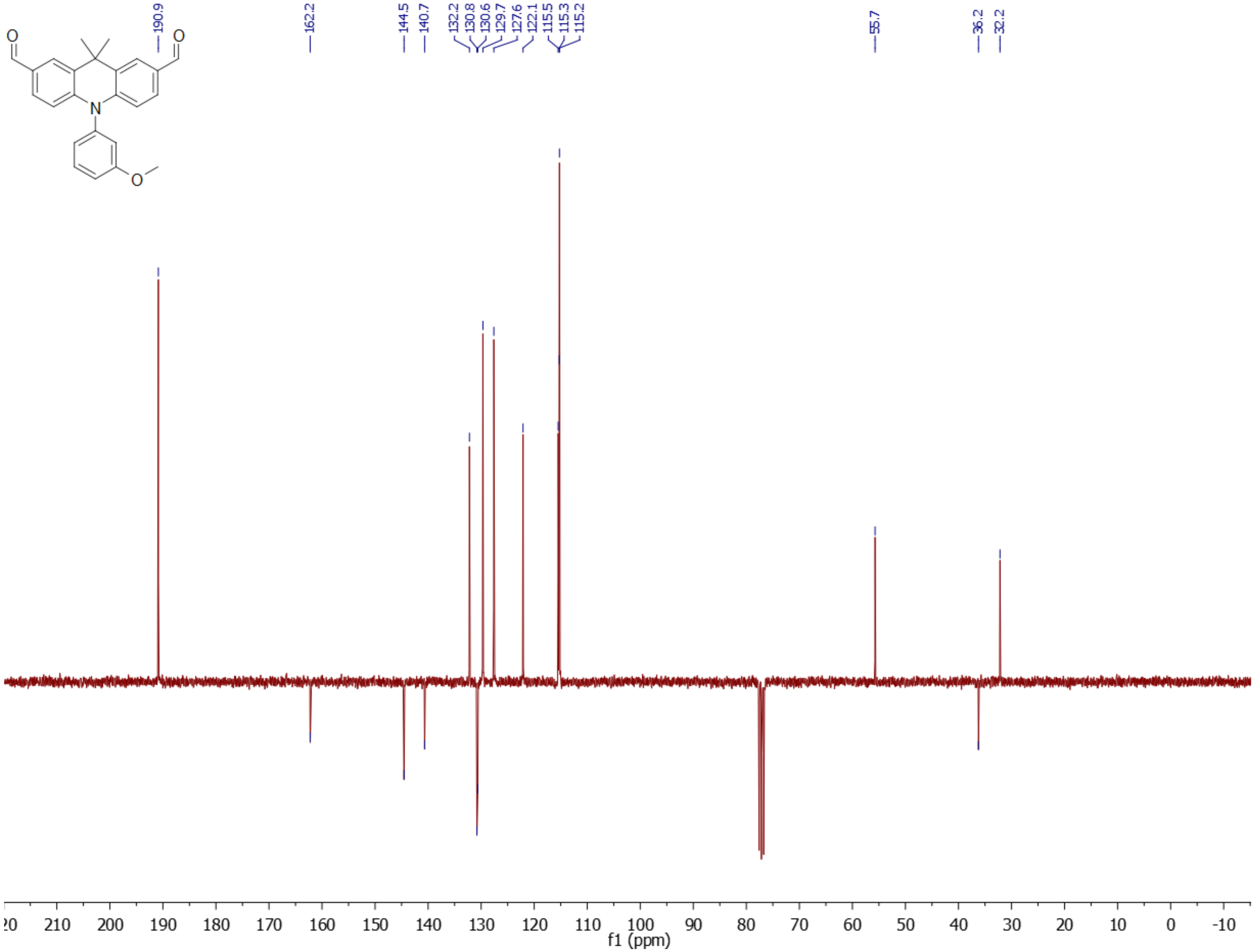

^{1}H NMR spectra of 3-*m* in $CDCl_3$ (300 MHz):

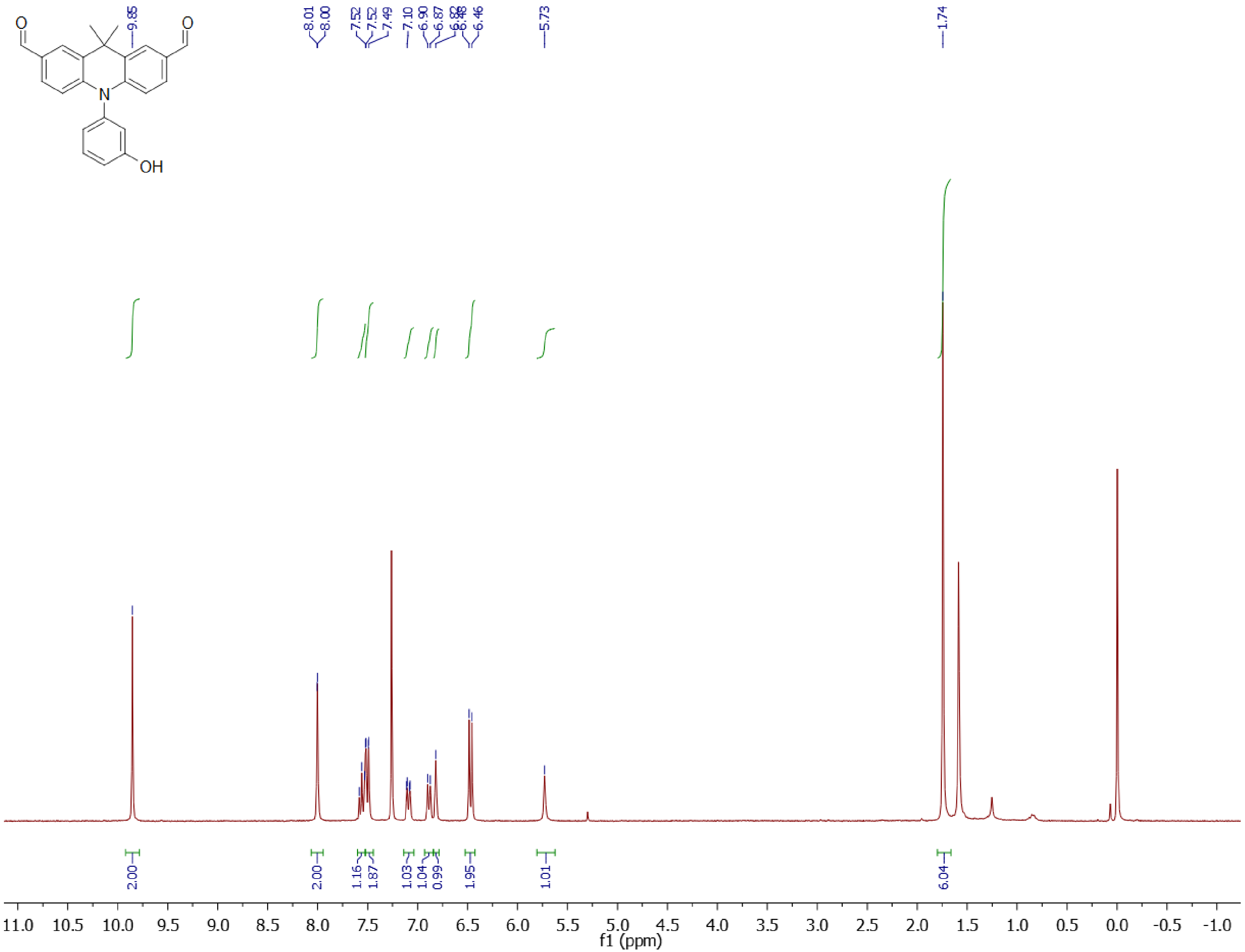

APT NMR spectra of 3-*m* in $CDCl_3$ (75 MHz):

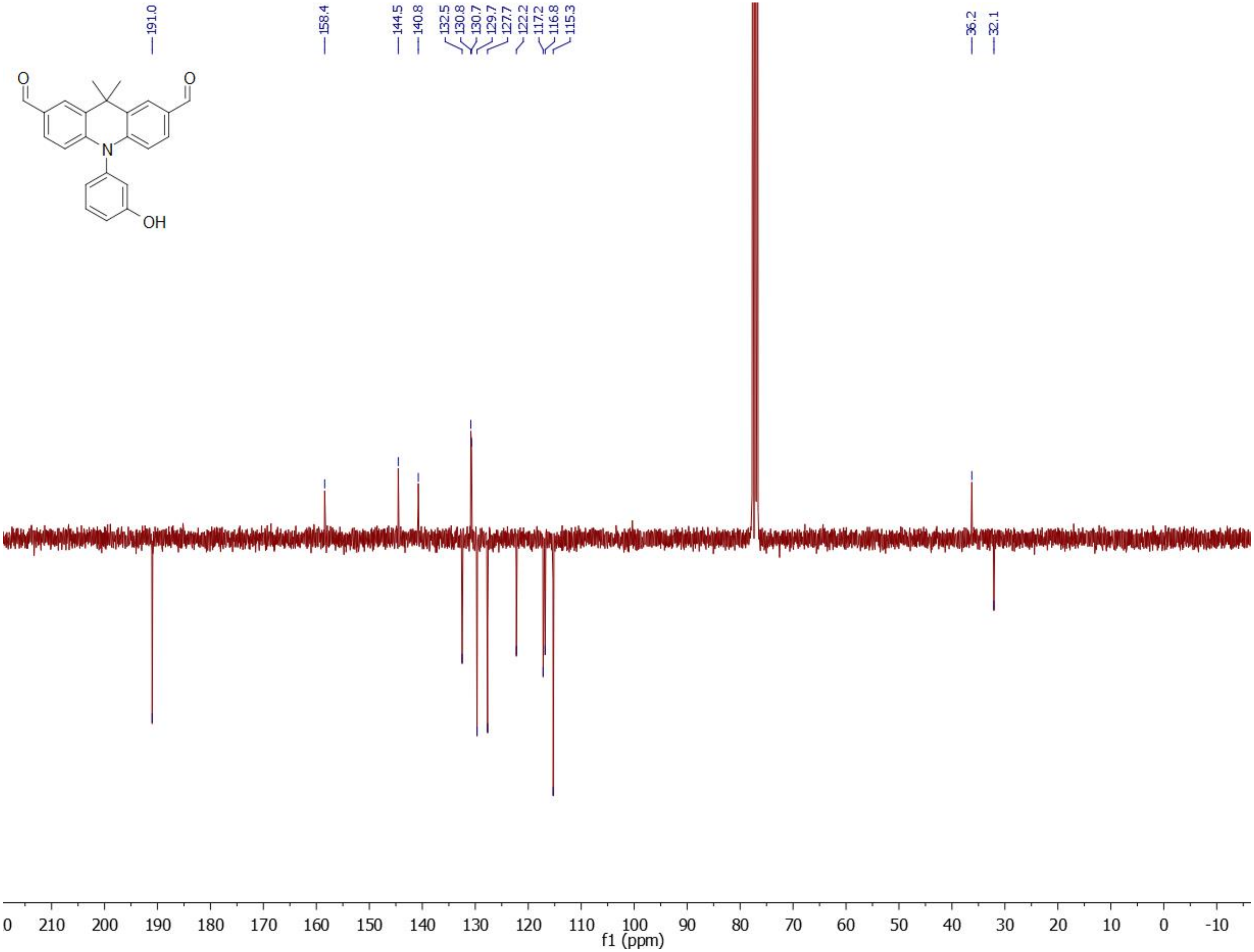

¹H NMR spectra of 5-*m* in $CDCl_3$ (300 MHz):

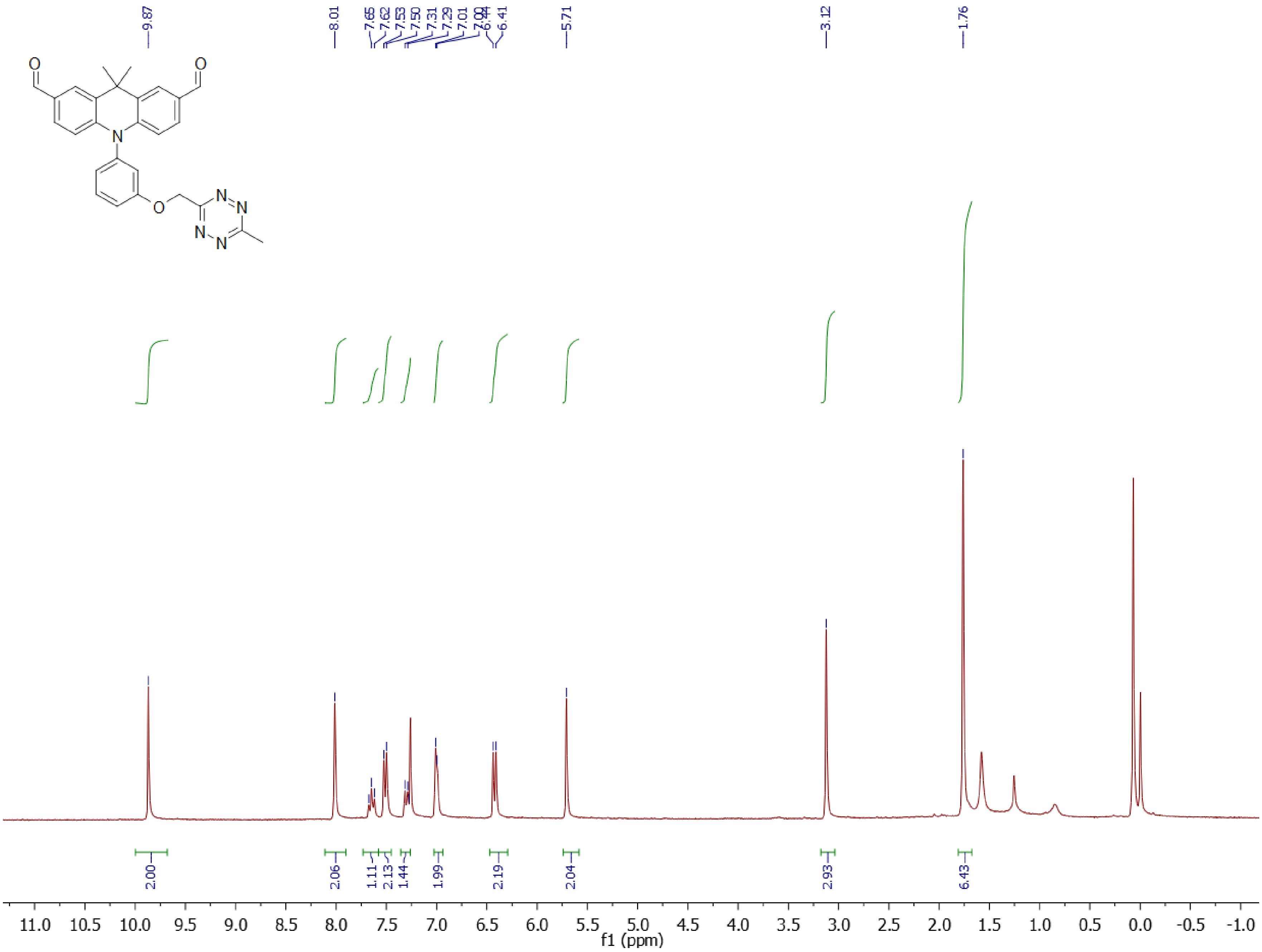

APT NMR spectra of 5-*m* in $CDCl_3$ (75 MHz):

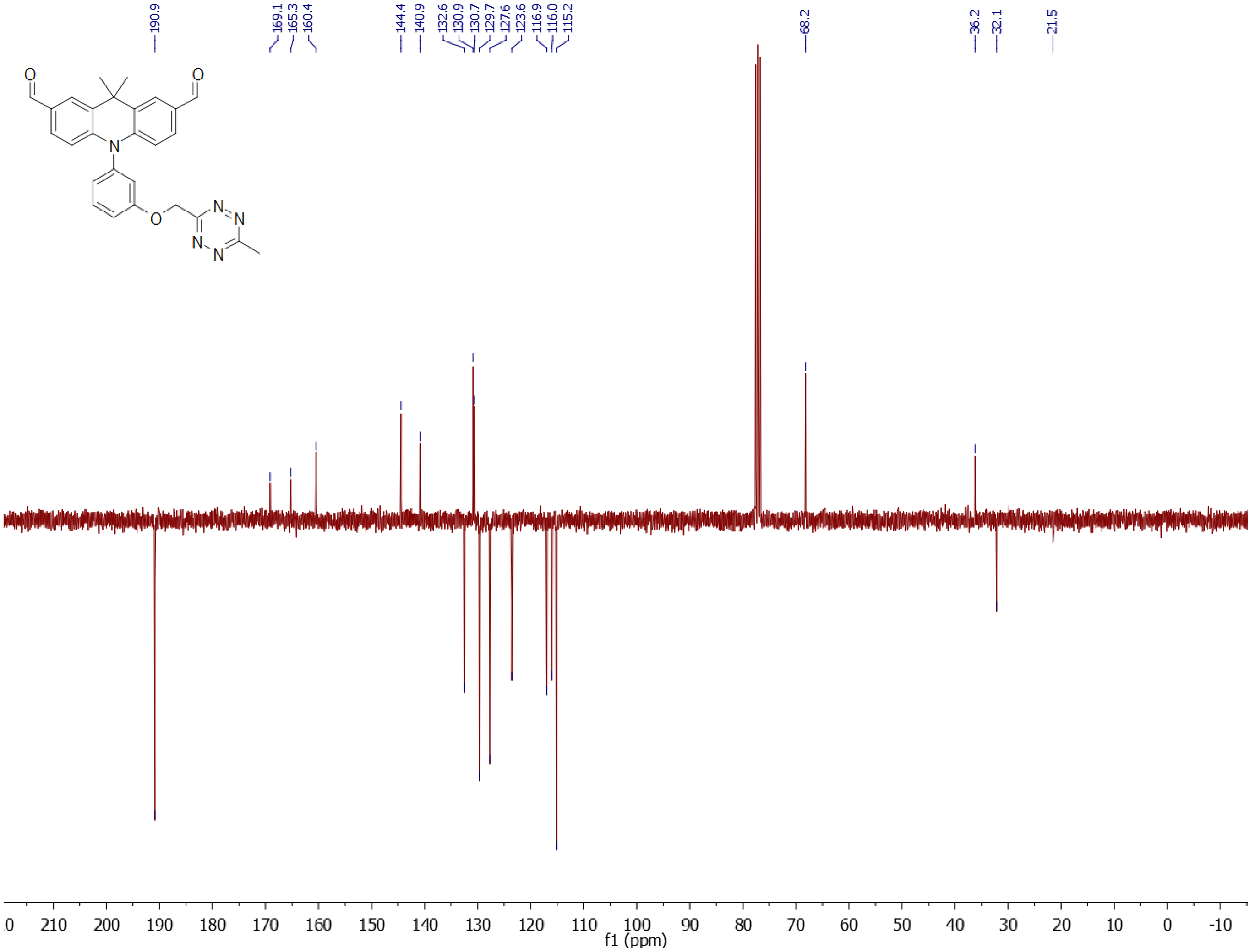

^{1}H NMR spectra of Acri-*m*et in MeOD-d_4 (300 MHz):

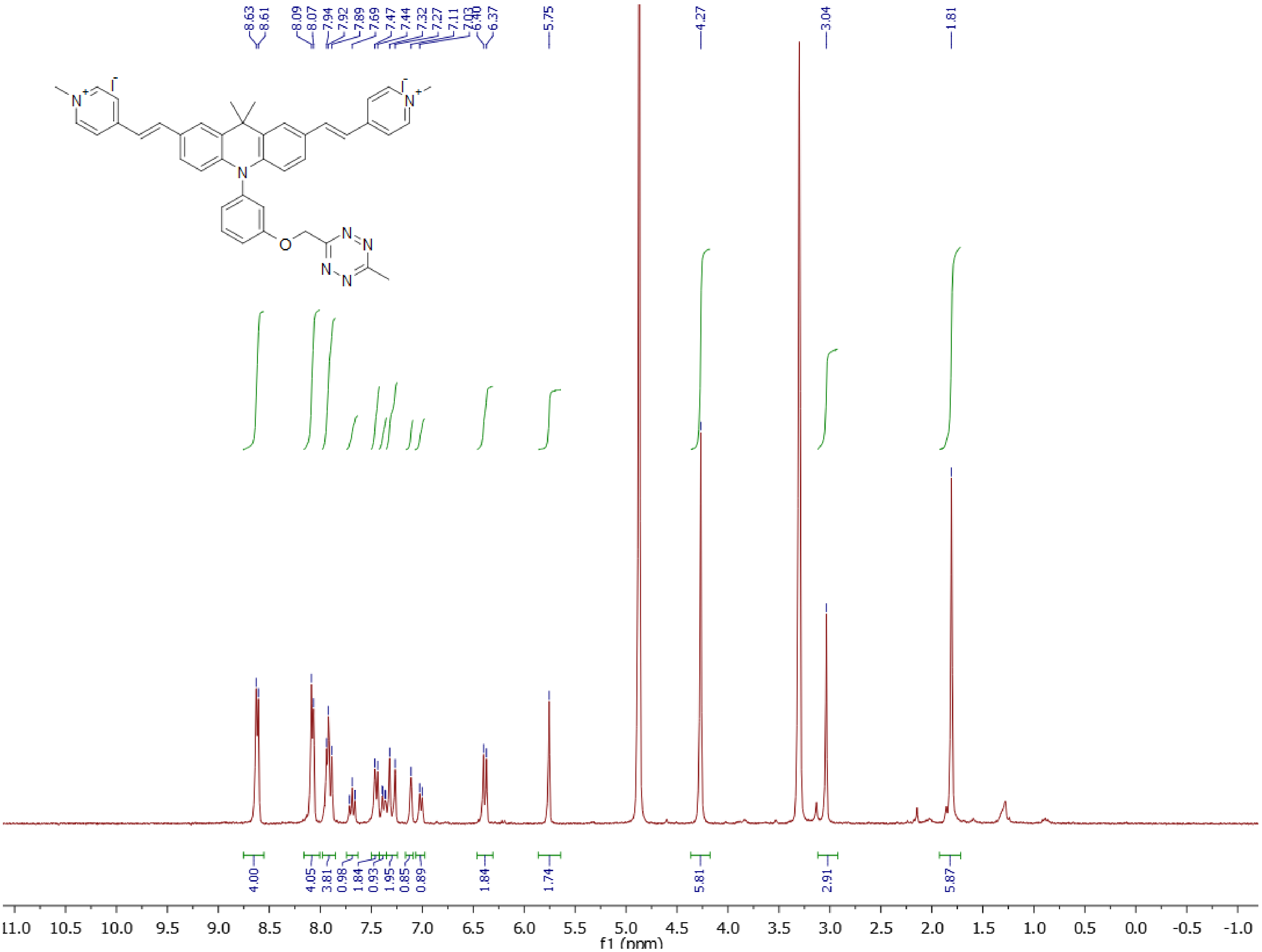

APT NMR spectra of Acri-*m*et in MeOD-d_4 (75 MHz):

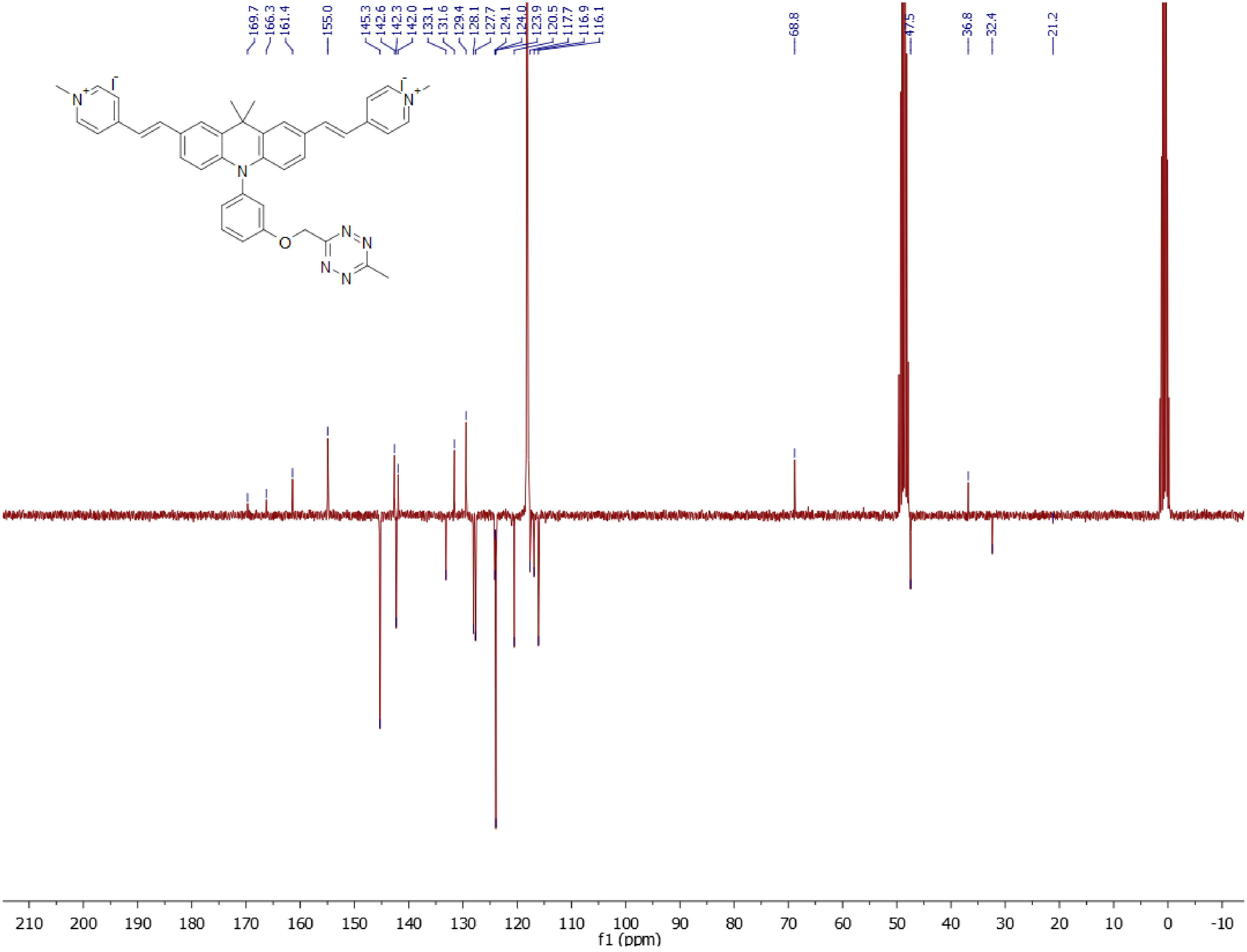

HSQC NMR spectra of Acri-*m*et in MeOD-d_4:

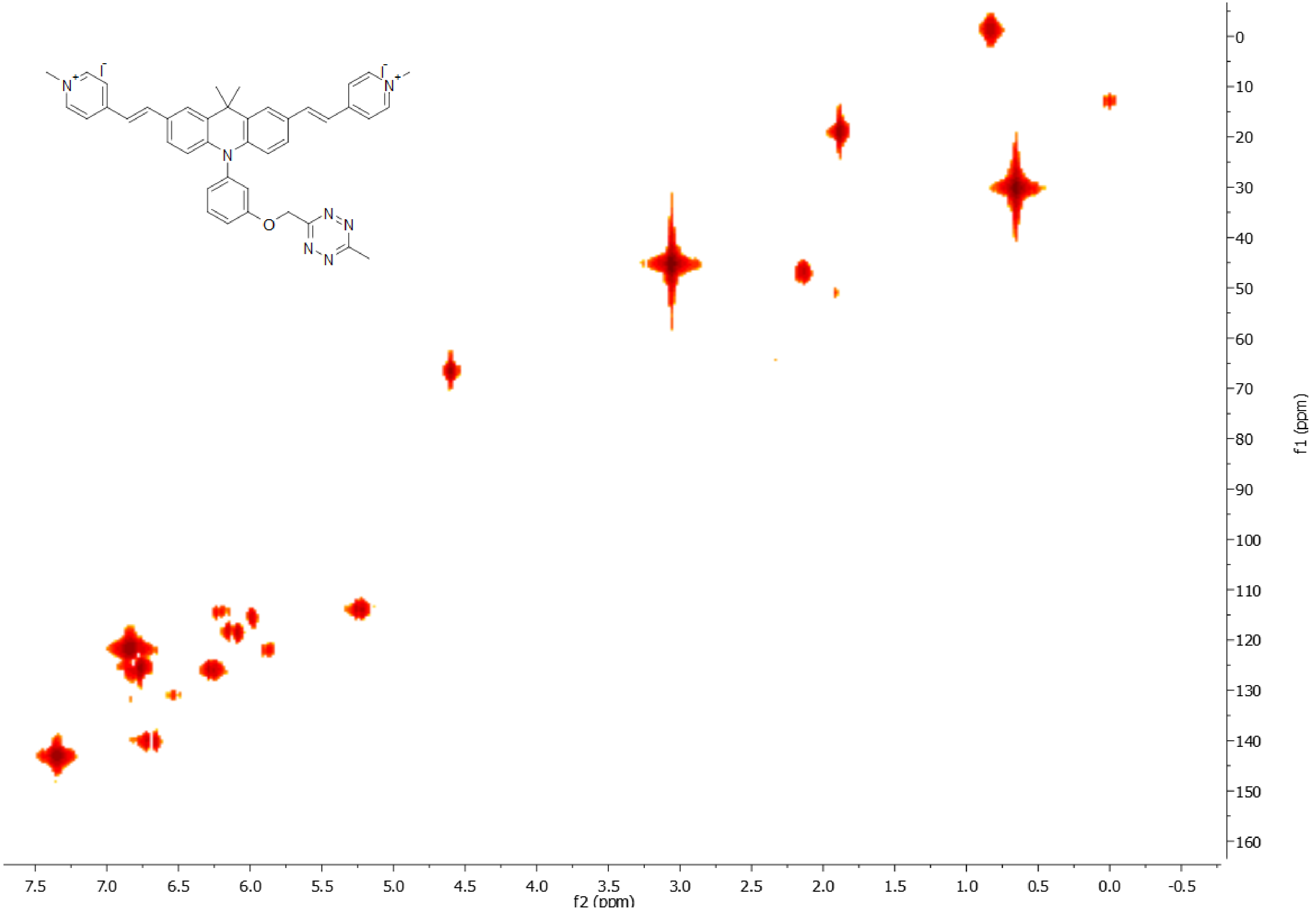

^{1}H NMR spectra of 2-*p* in $CDCl_3$ (300 MHz):

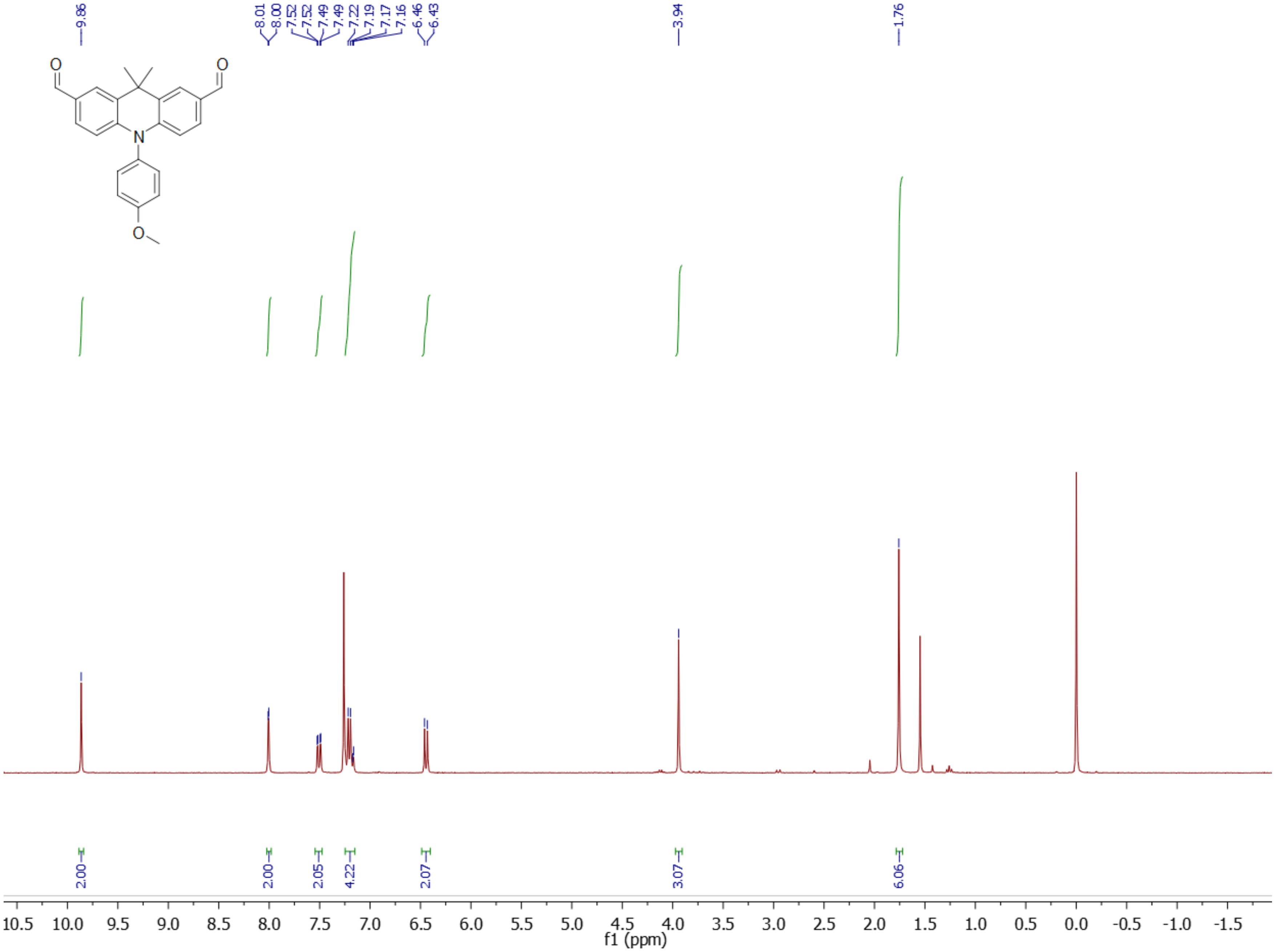

APT NMR spectra of 2-*p* in $CDCl_3$ (75 MHz):

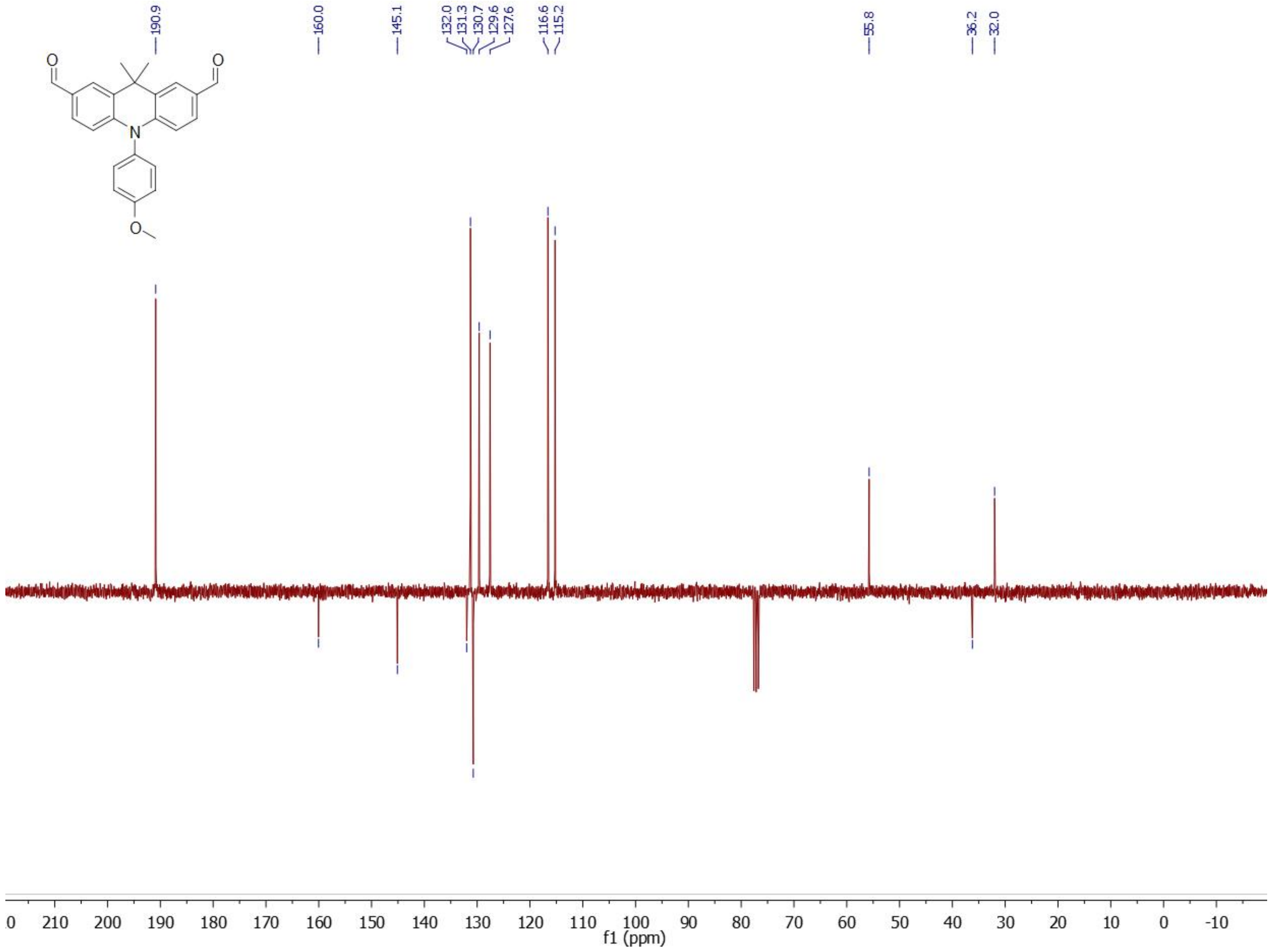

^{1}H NMR spectra of 3-*p* in $CDCl_3$ (300 MHz):

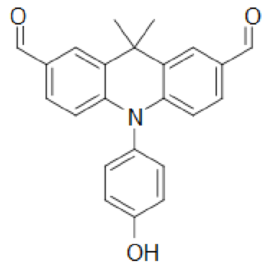

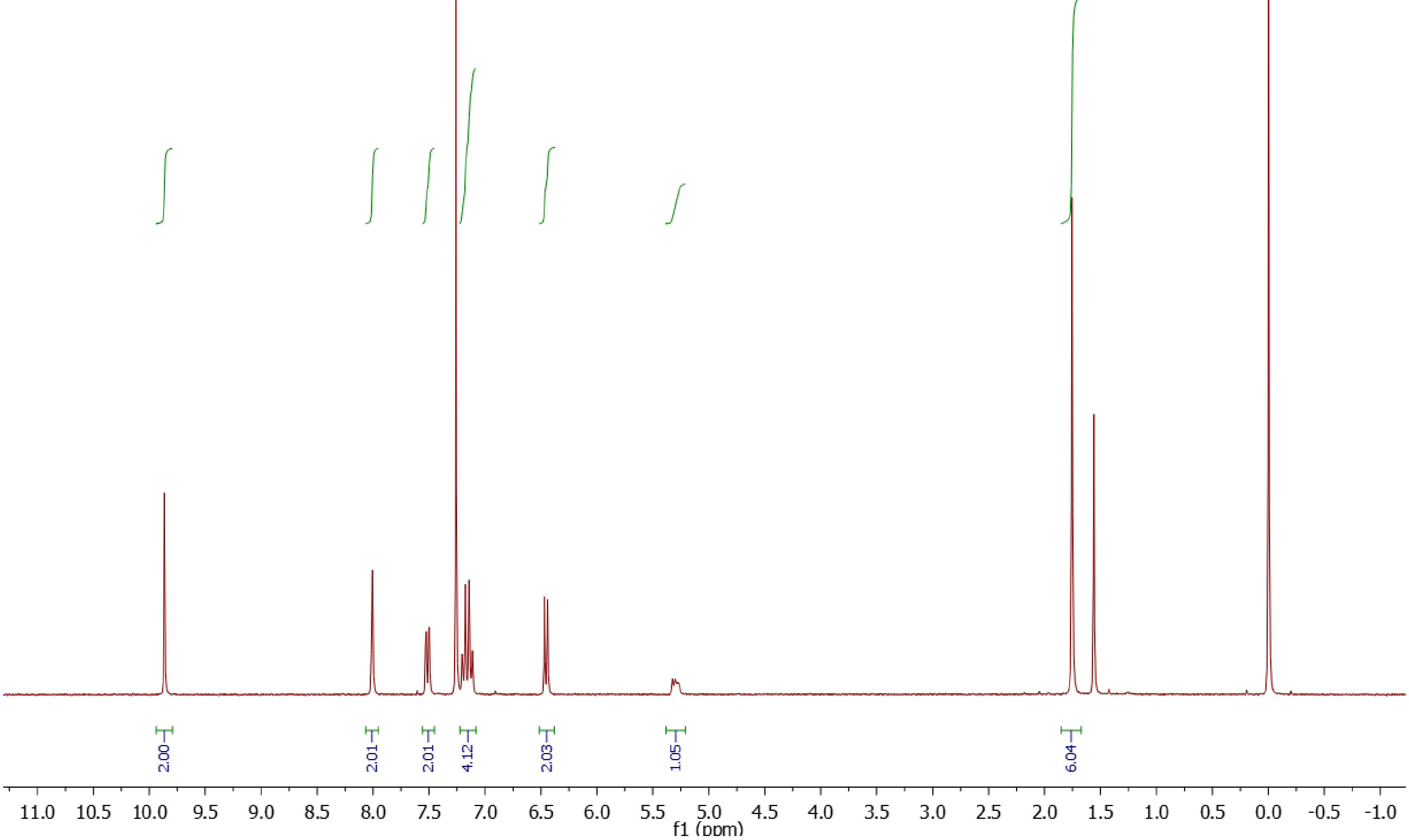

APT NMR spectra of 3-*p* in $CDCl_3$ (75 MHz):

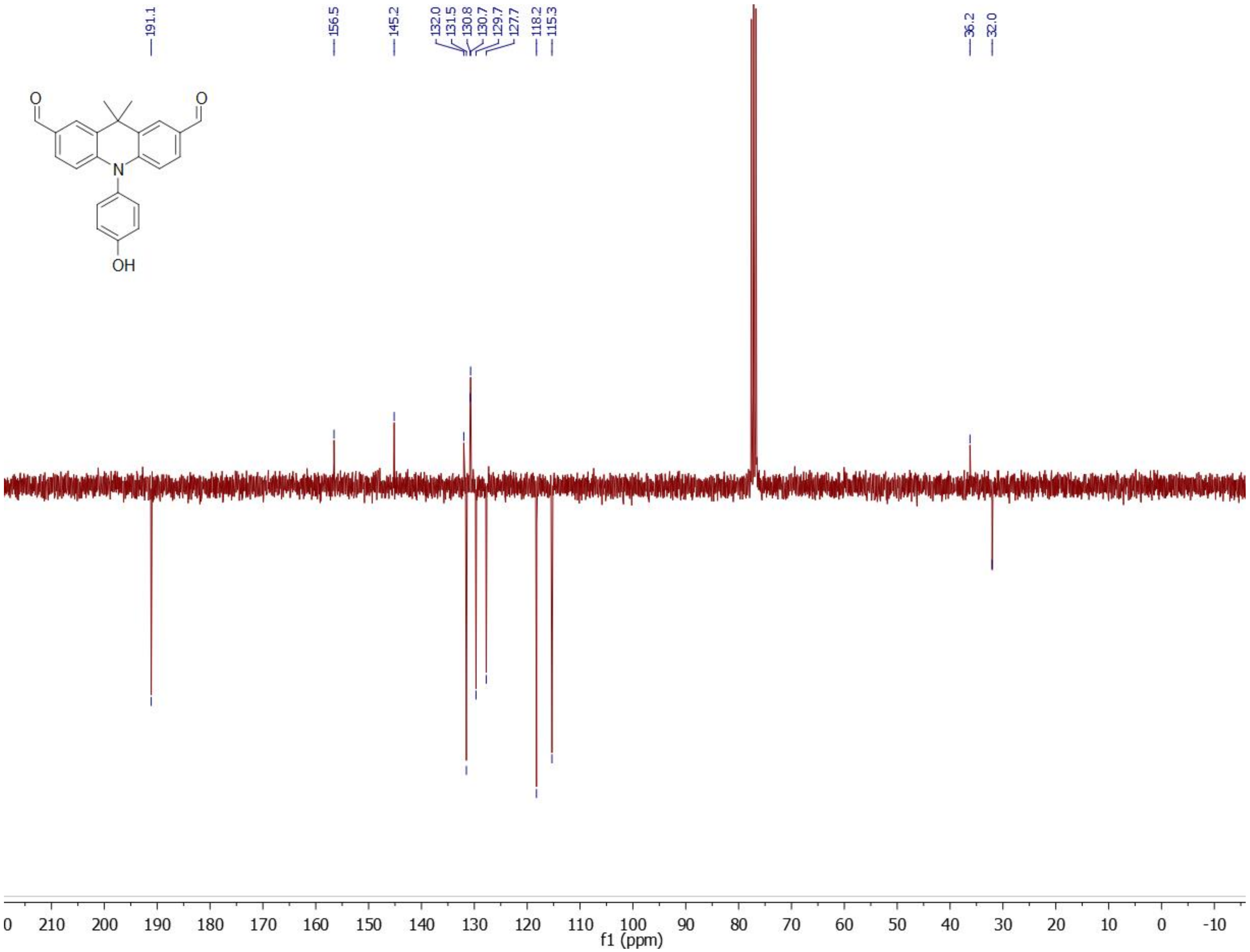

^{1}H NMR spectra of 5-*p* in $CDCl_3$ (300 MHz):

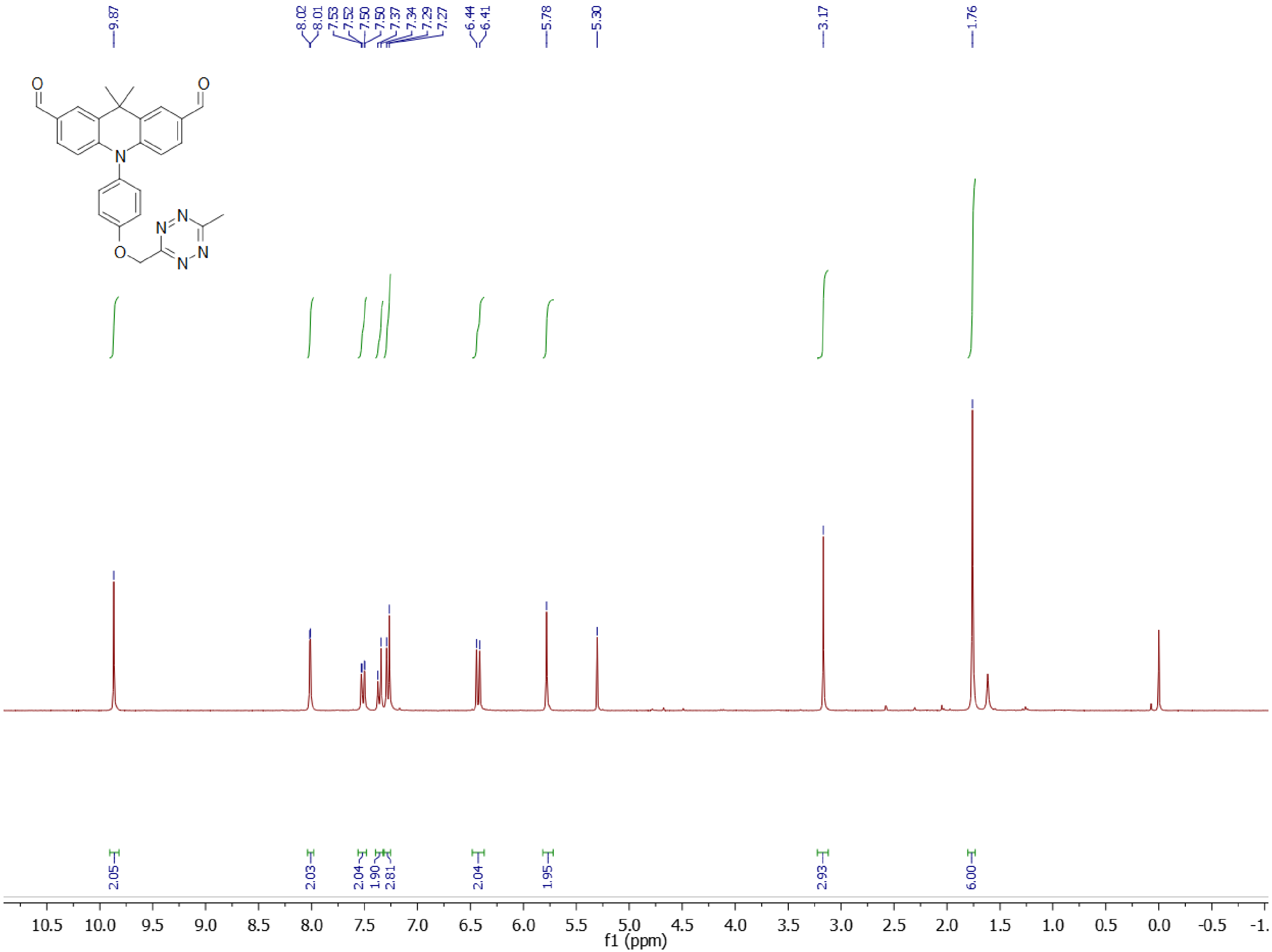

APT NMR spectra of 5-*p* in $CDCl_3$ (75 MHz):

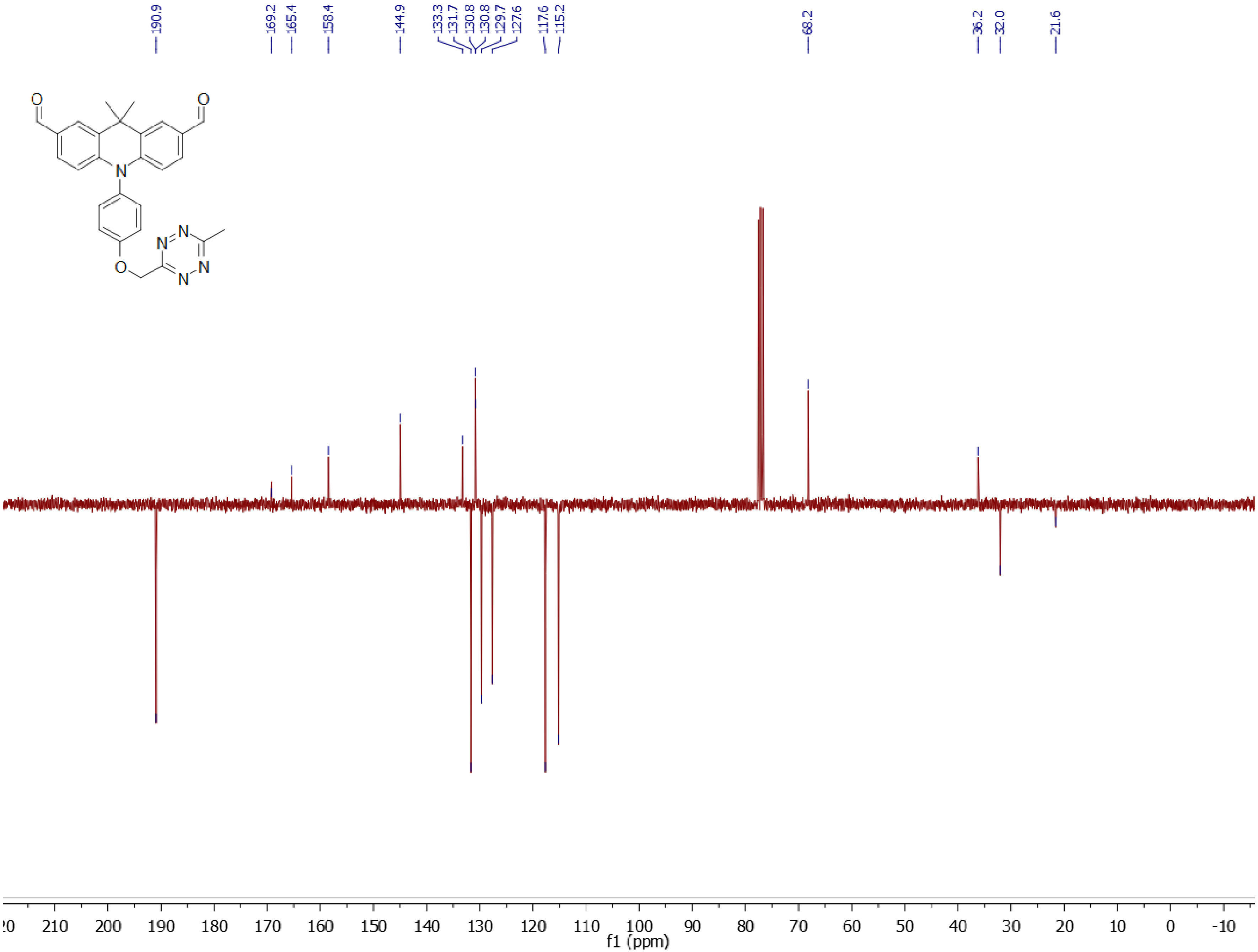

^{1}H NMR spectra of Acri-*pet* in DMSO-d_6 (300 MHz):

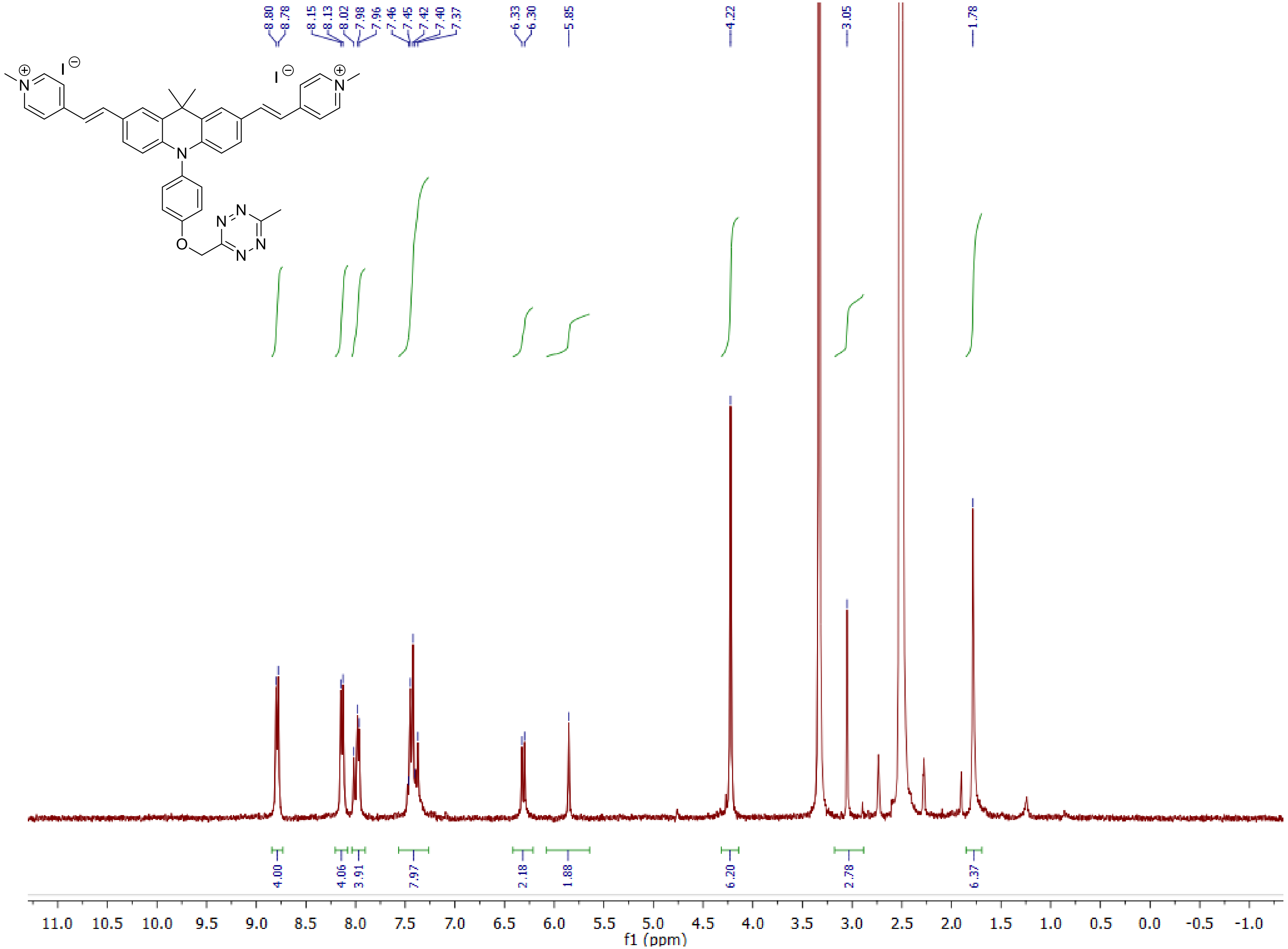

APT NMR spectra Acri-*pet* in DMSO-d_6 (75 MHz):

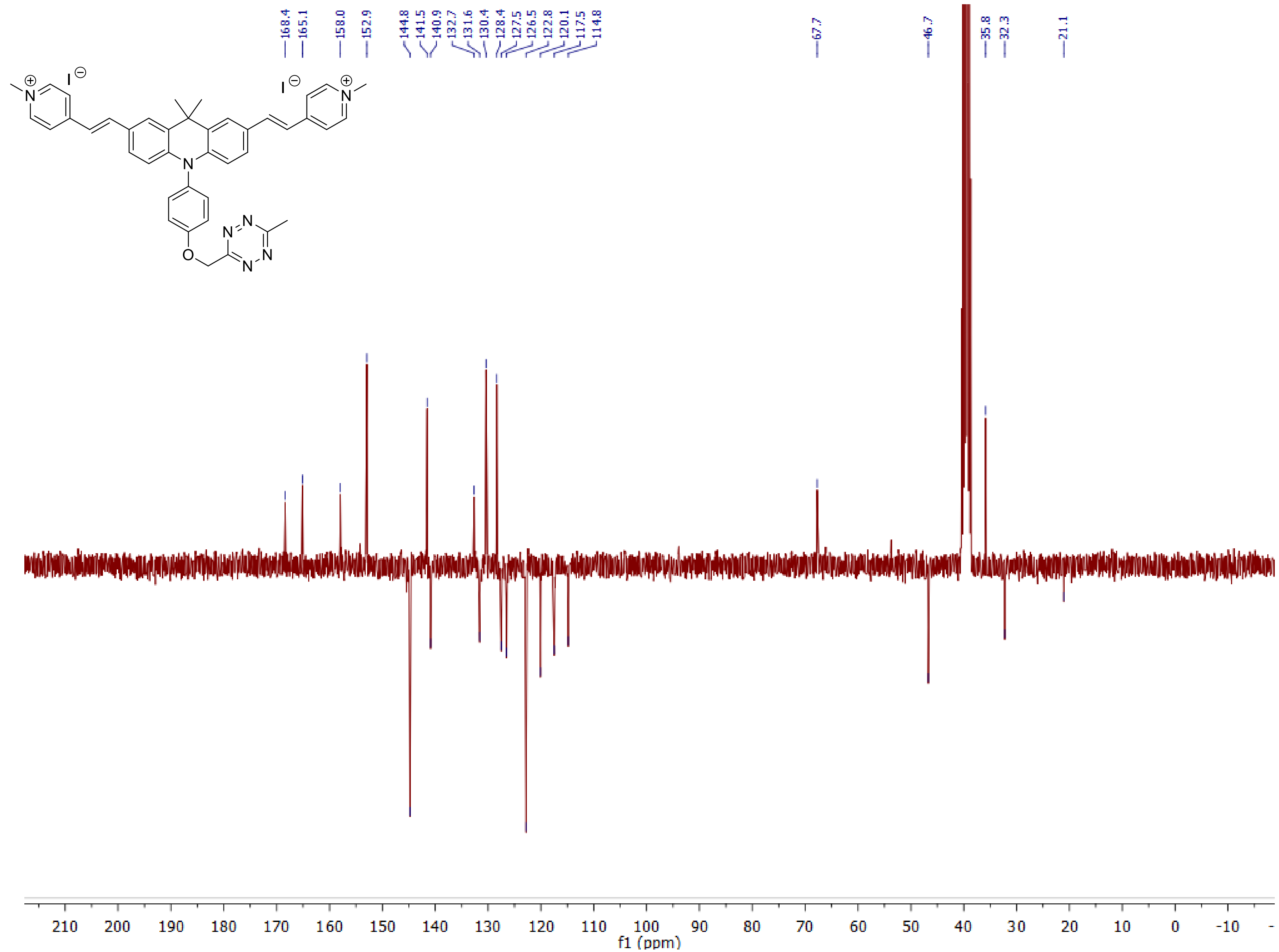

^{1}H NMR spectra of 8-*o* in $CDCl_3$ (300 MHz):

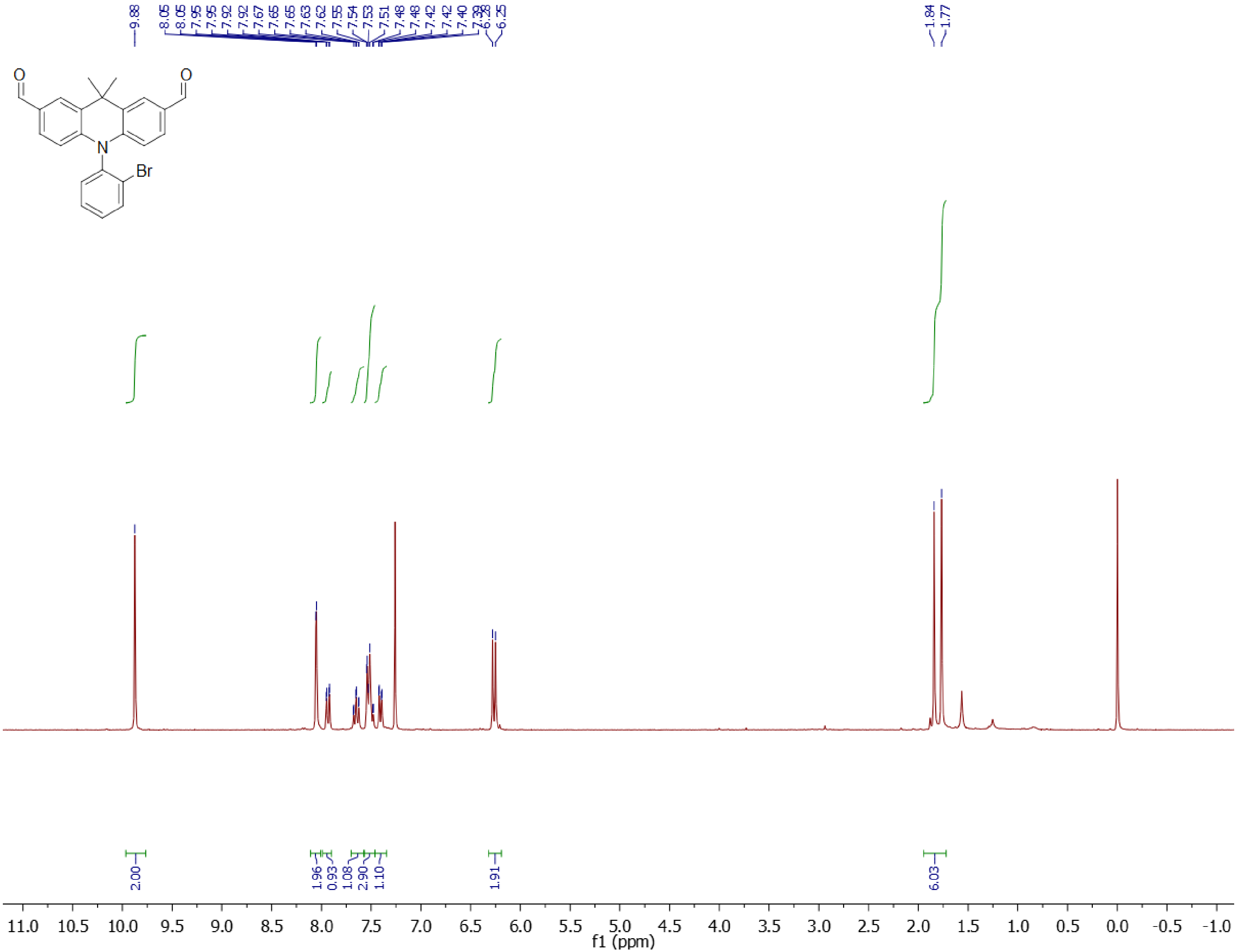

APT NMR spectra of 8-*o* in $CDCl_3$ (75 MHz):

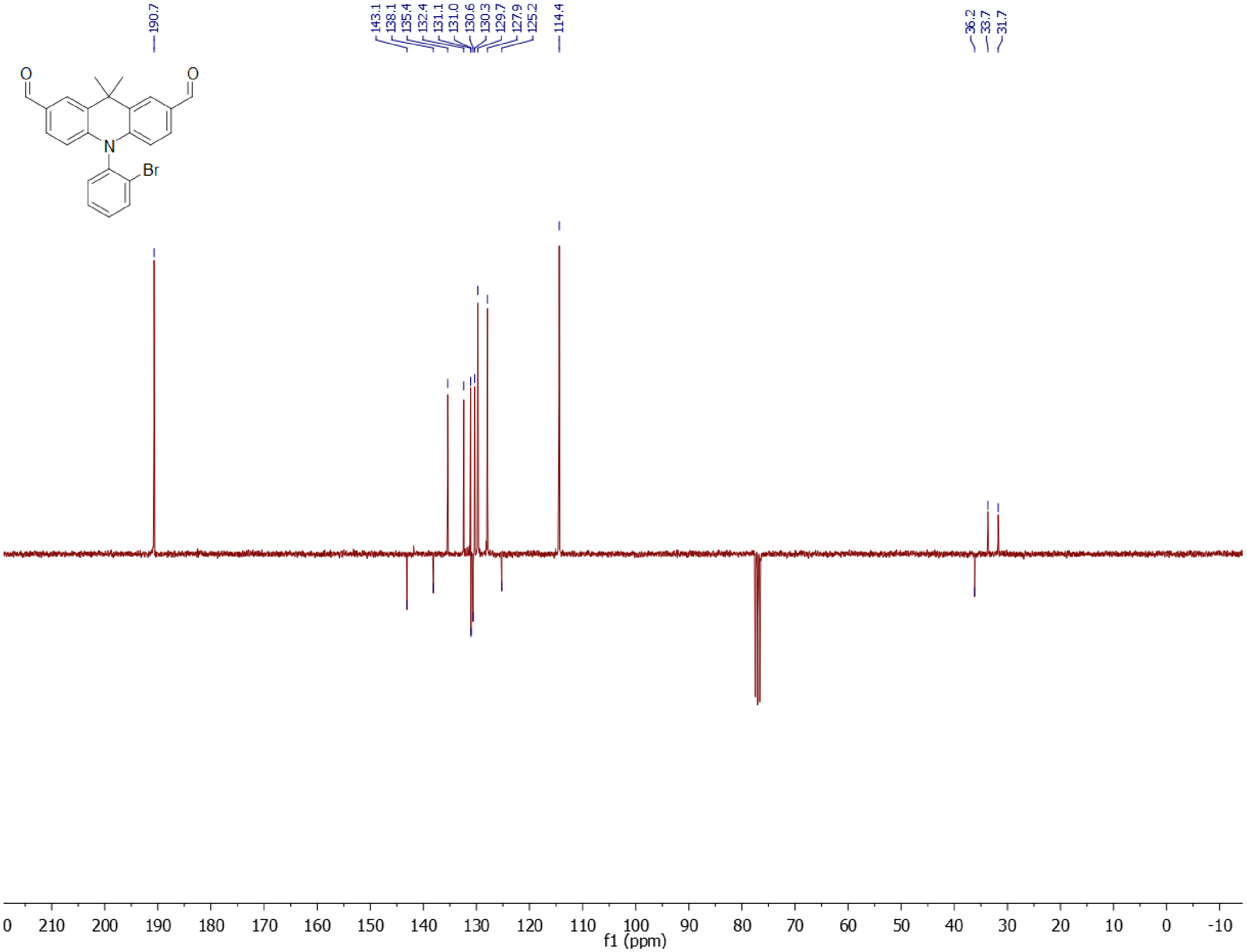

^{1}H NMR spectra of 10-*o* in $CDCl_3$ (300 MHz):

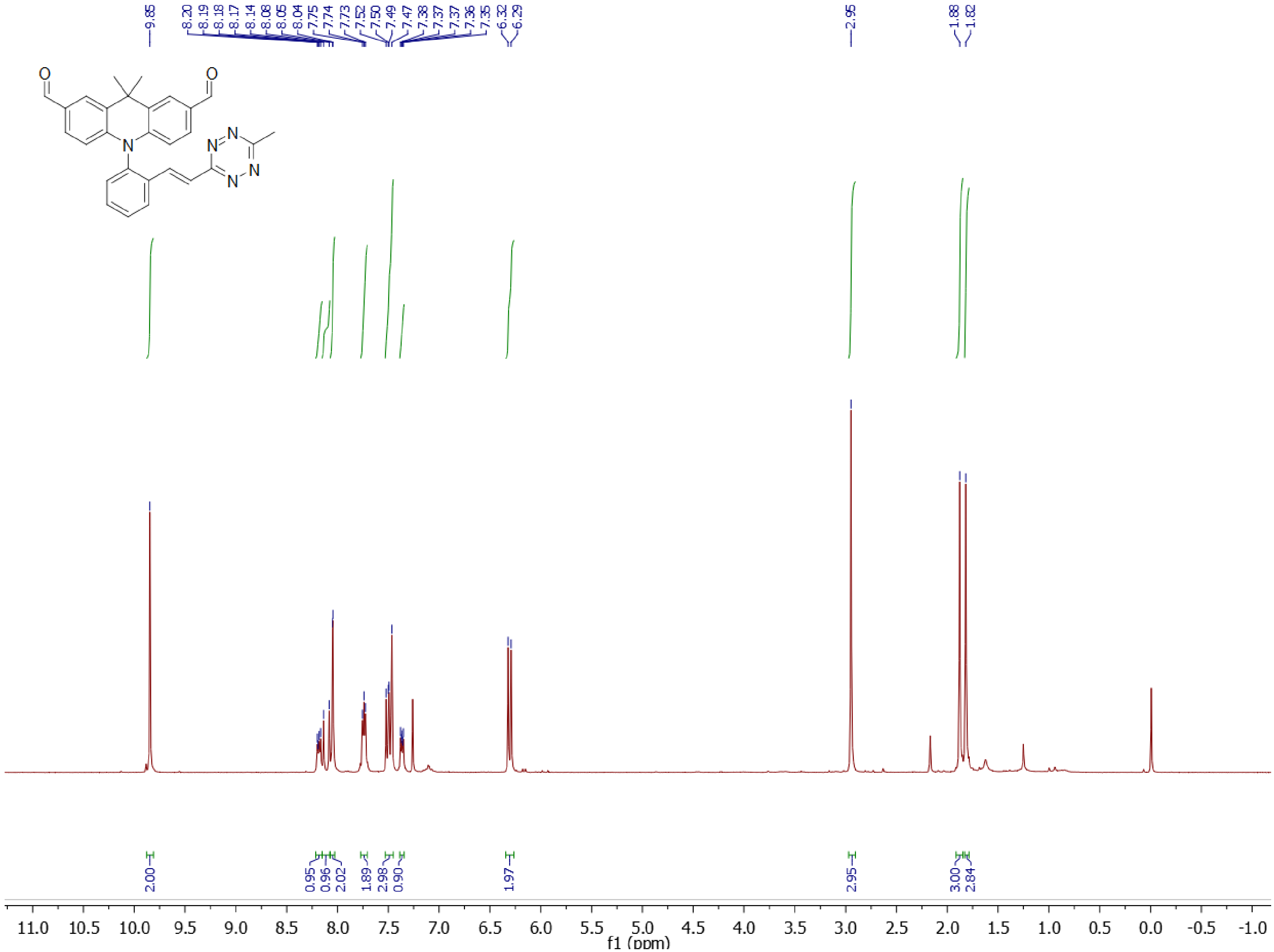

APT NMR spectra of 10-*o* in $CDCl_3$ (75 MHz):

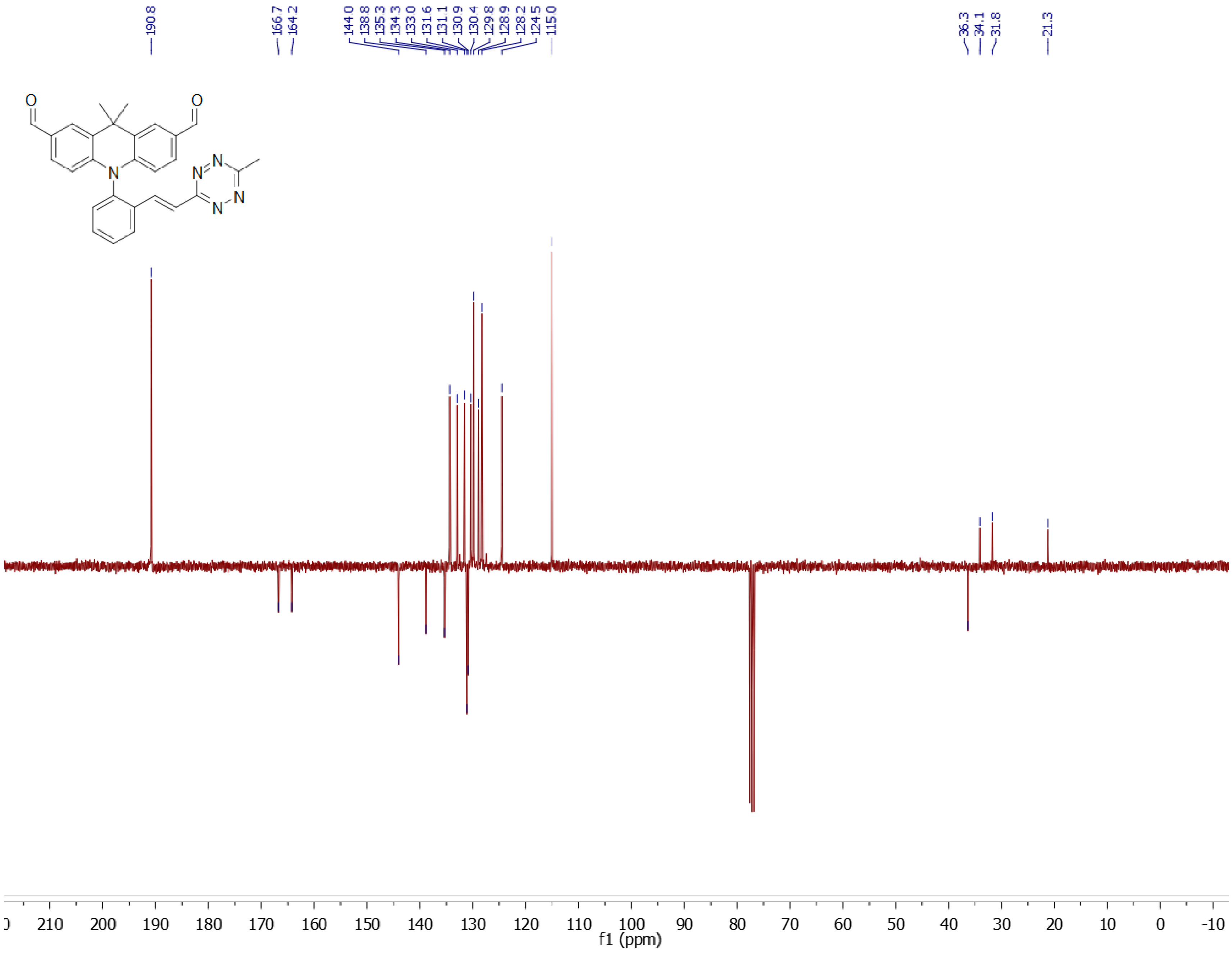

^{1}H NMR spectra of Acri-*ovi* in DMSO-d_6 (300 MHz):

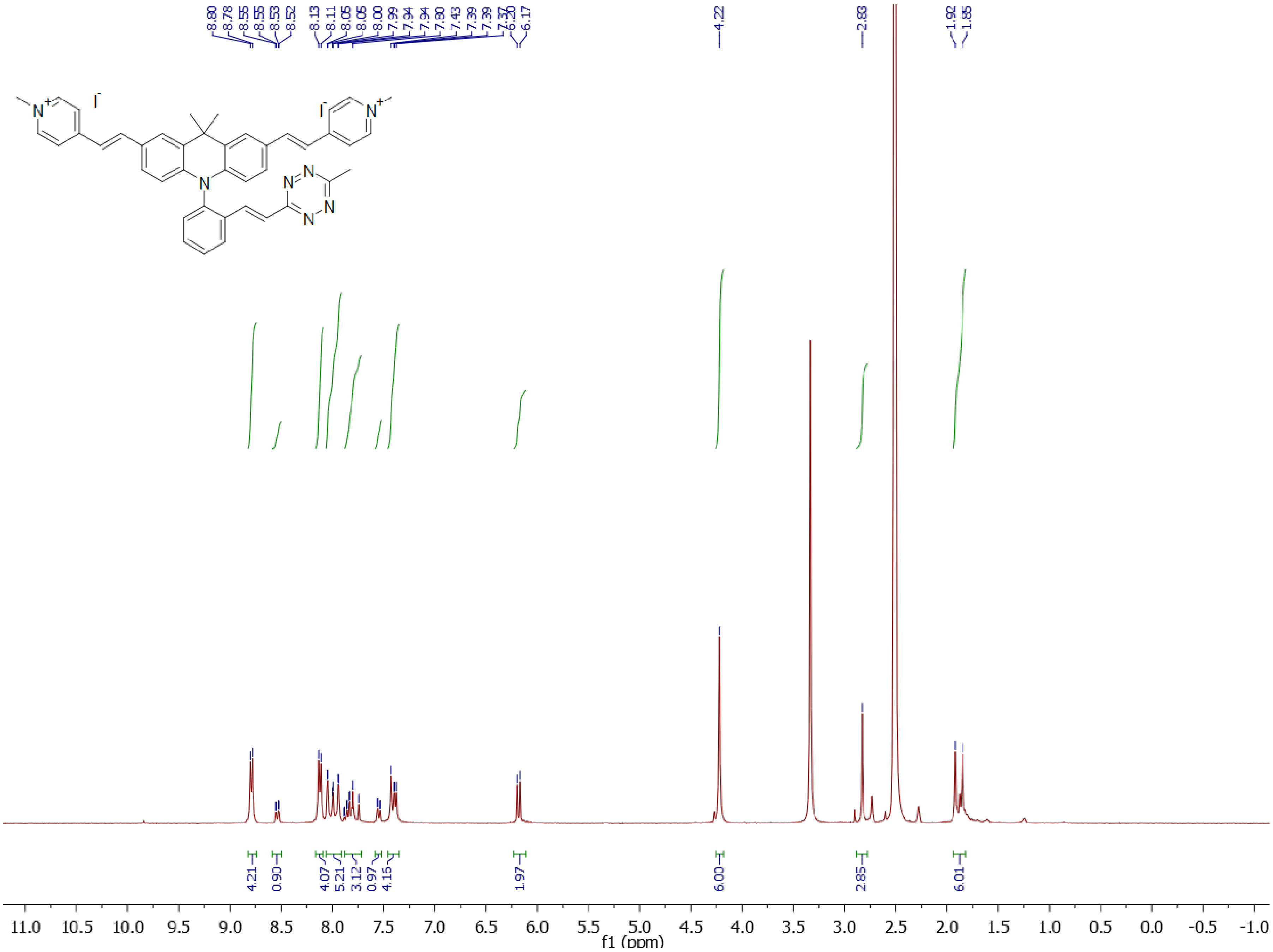

APT NMR spectra of Acri-*ovi* in DMSO-d_6 (75 MHz):

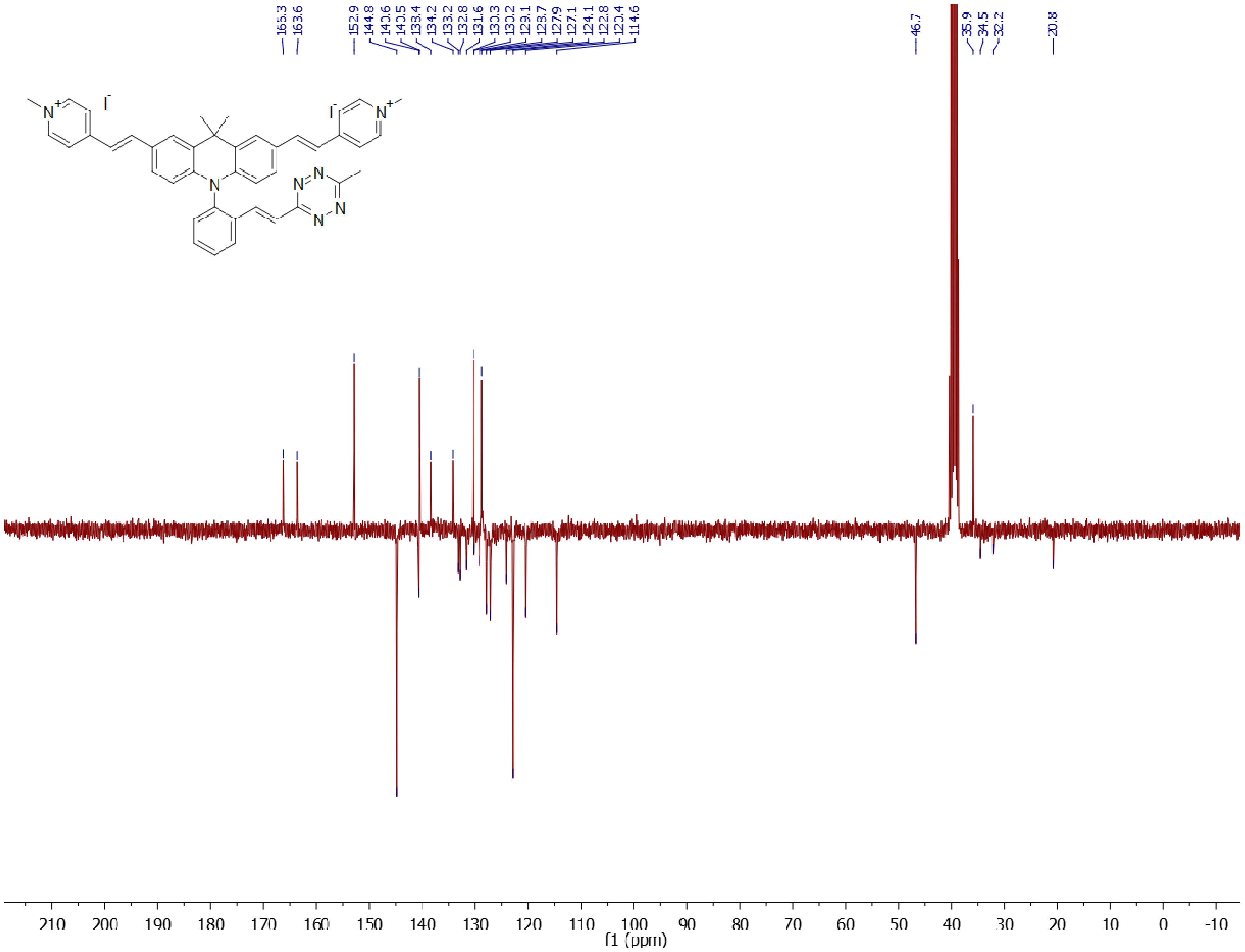

^{1}H NMR spectra of 7-*m* in $CDCl_3$ (300 MHz):

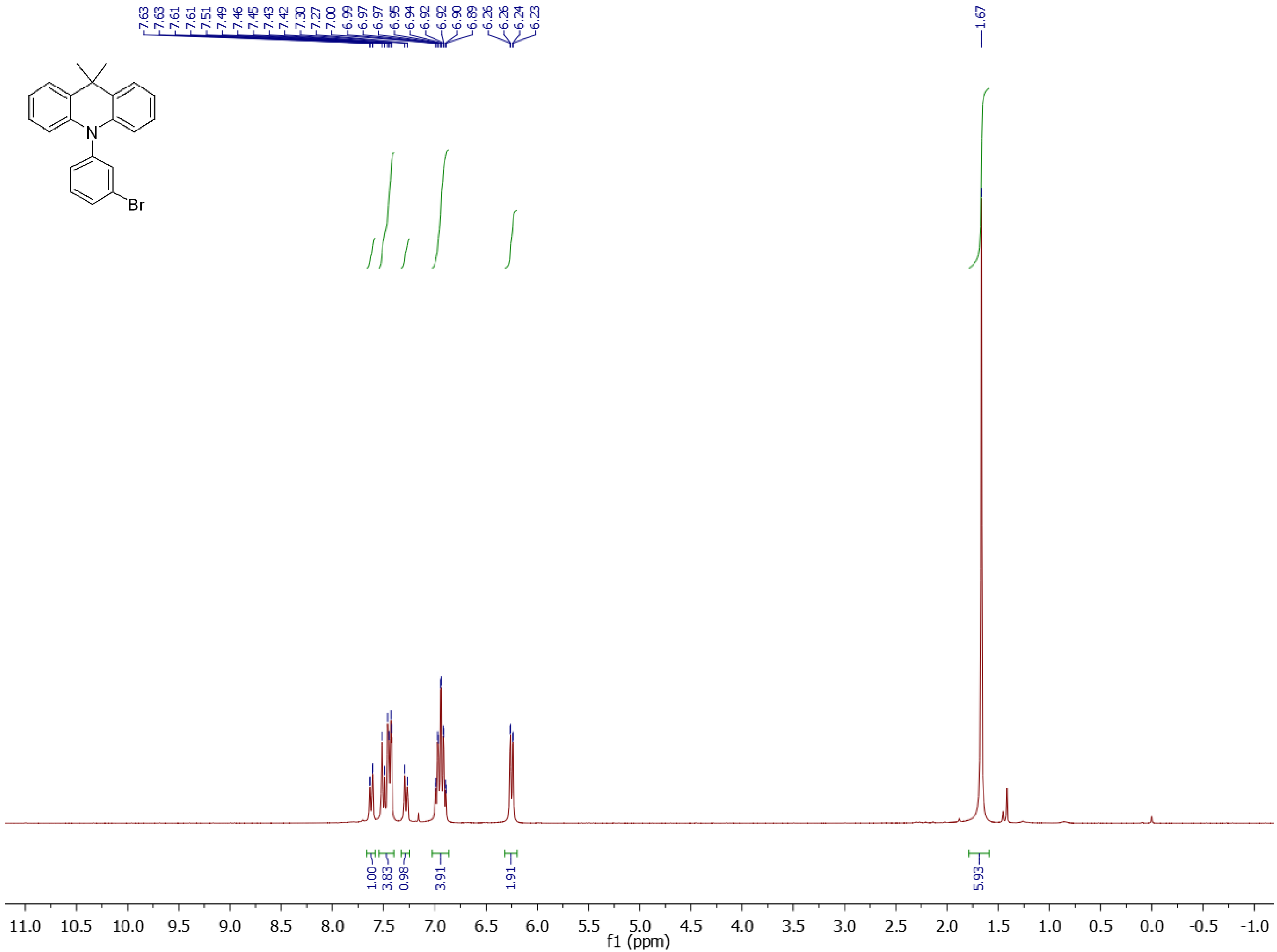

APT NMR spectra of 7-*m* in $CDCl_3$ (75 MHz):

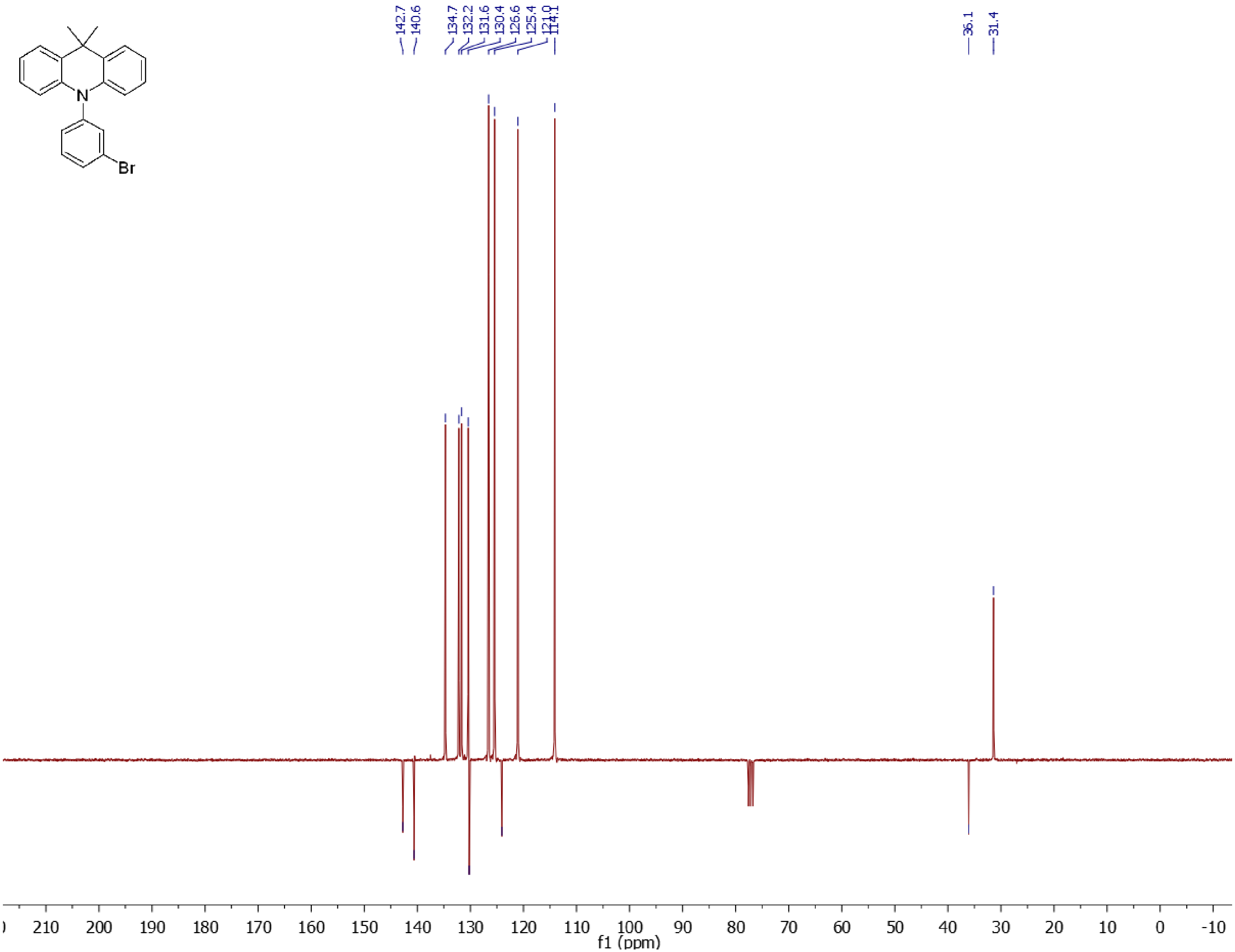

^{1}H NMR spectra of 8-*m* in $CDCl_3$ (300 MHz):

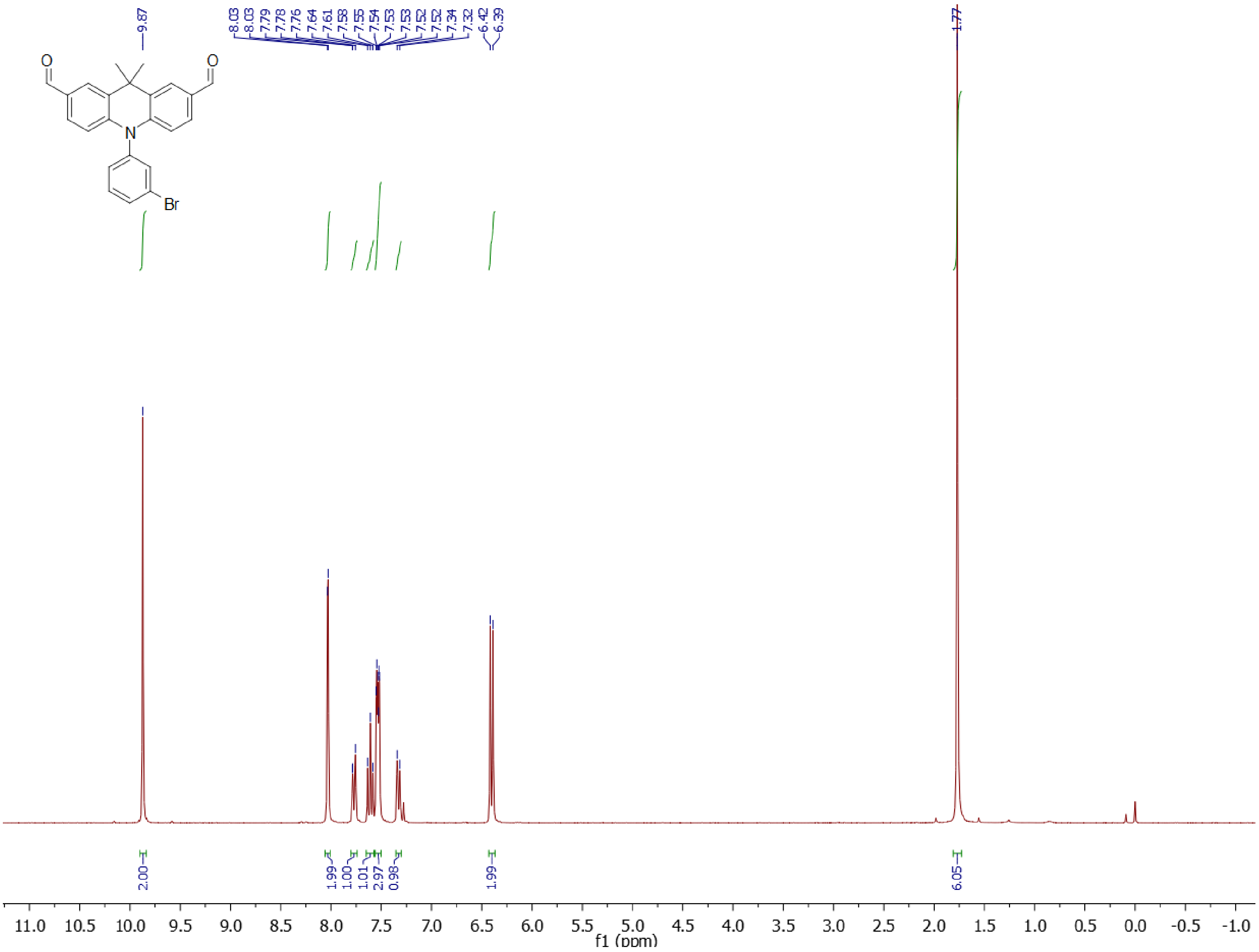

APT NMR spectra of 8-*m* in $CDCl_3$ (75 MHz):

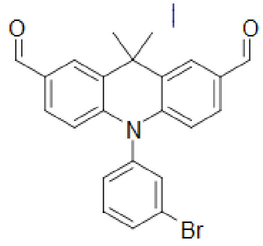

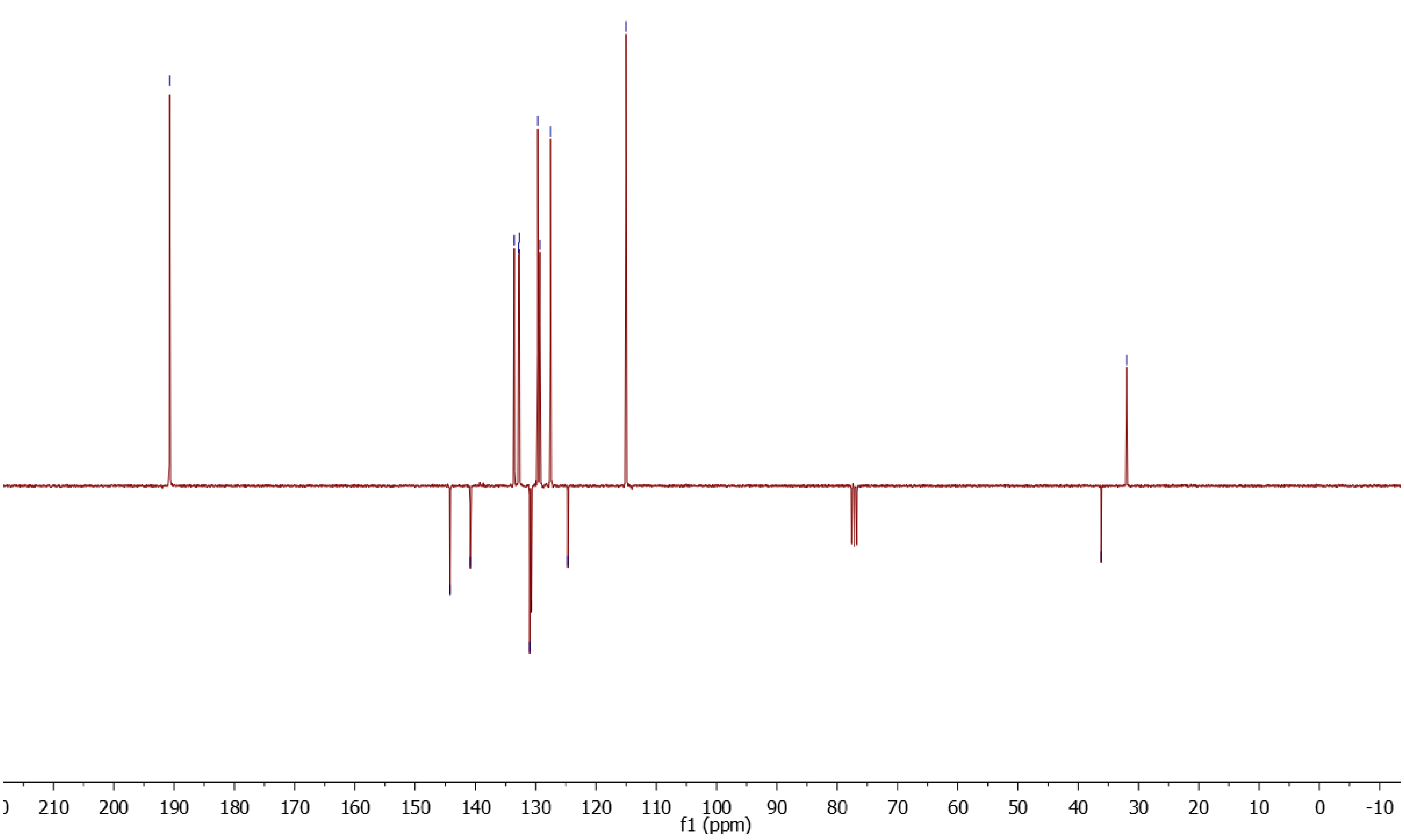

^{1}H NMR spectra of 10-*m* in $CDCl_3$ (300 MHz):

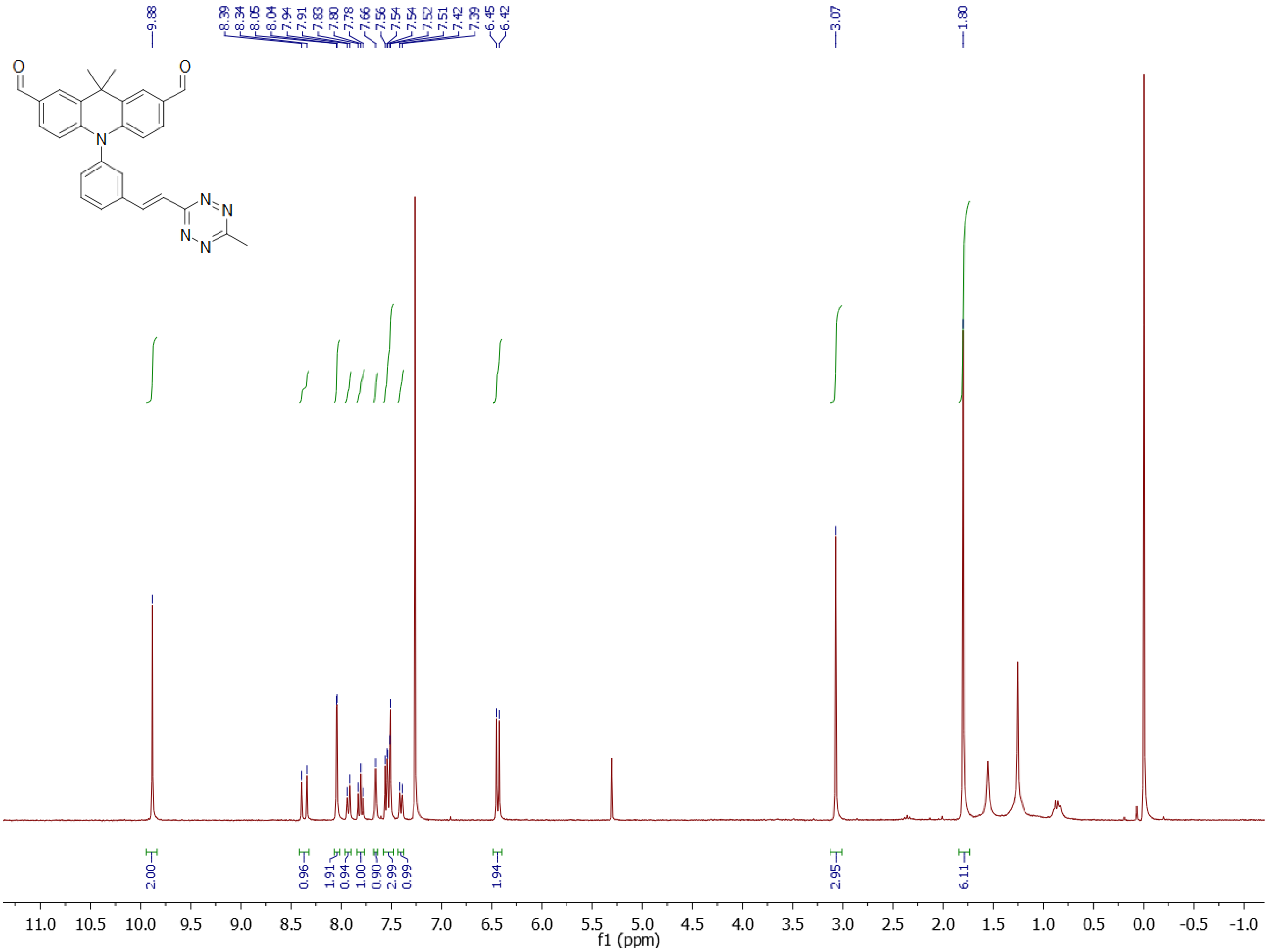

APT NMR spectra of 10-*m* in $CDCl_3$ (75 MHz):

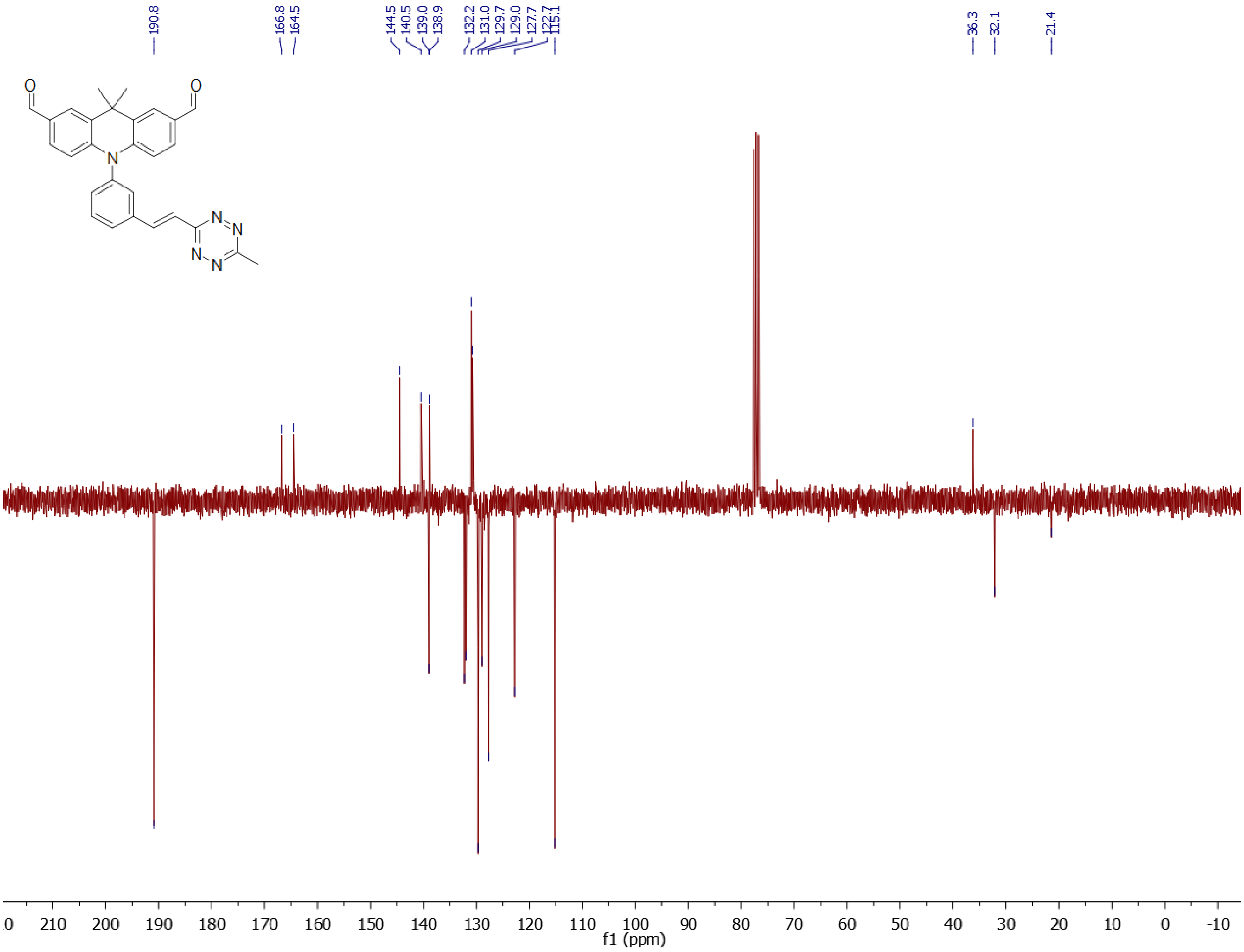

^{1}H NMR spectra of Acri-*m*vi in DMSO-d$_6$ (300 MHz):

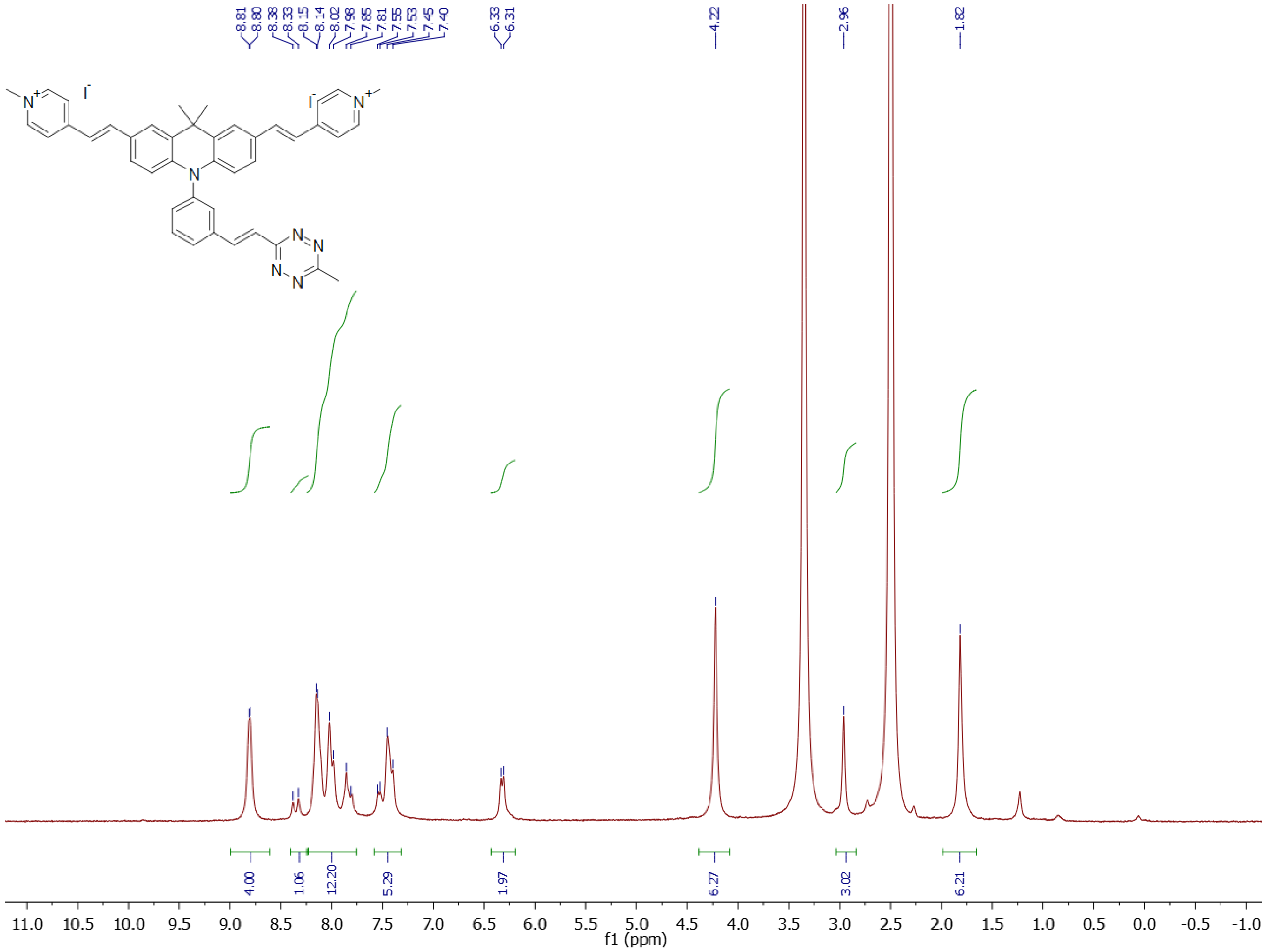

APT NMR spectra of Acri-*m*vi in DMSO-d_6 (75 MHz):

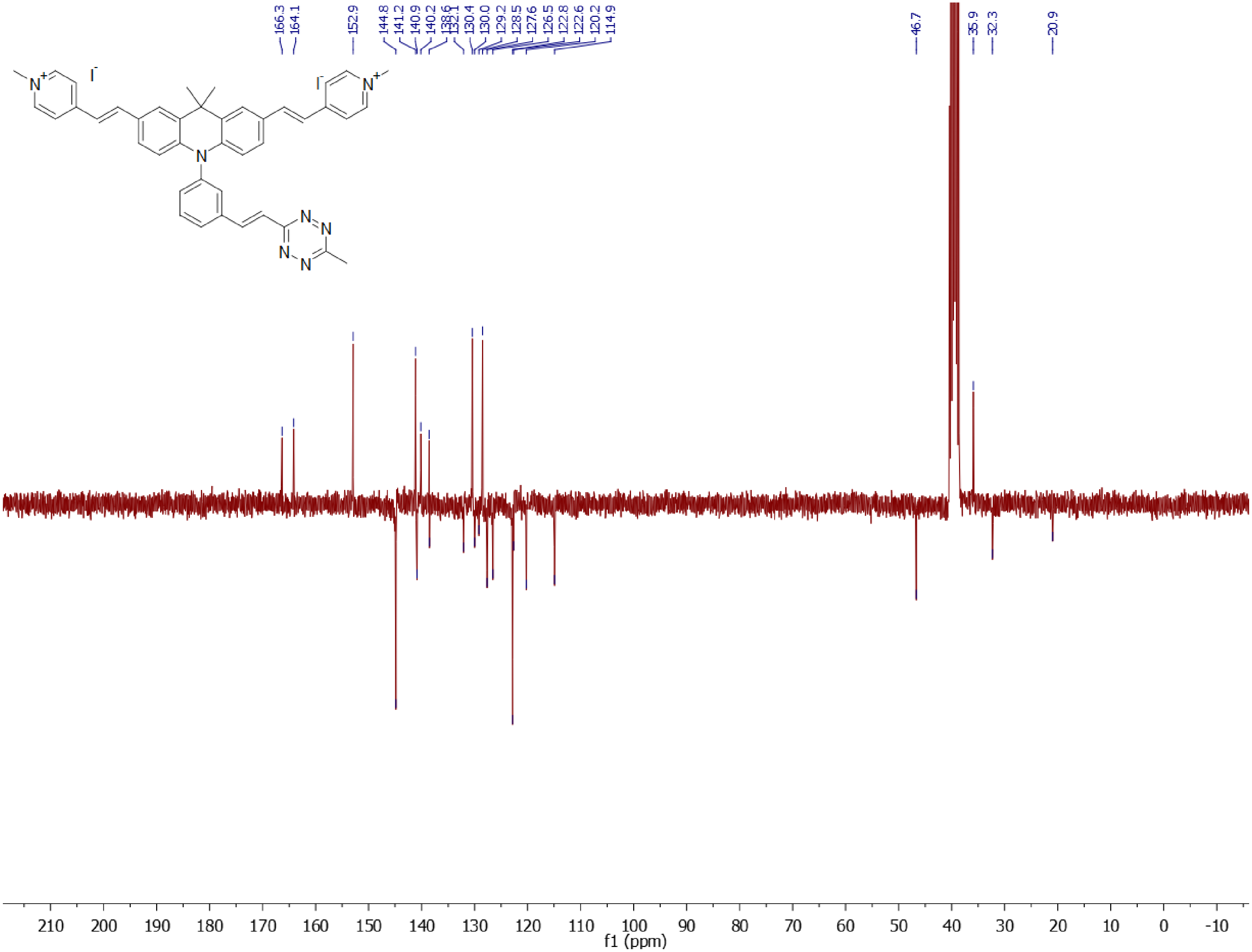

^{1}H NMR spectra of 8-*p* in $CDCl_3$ (300 MHz):

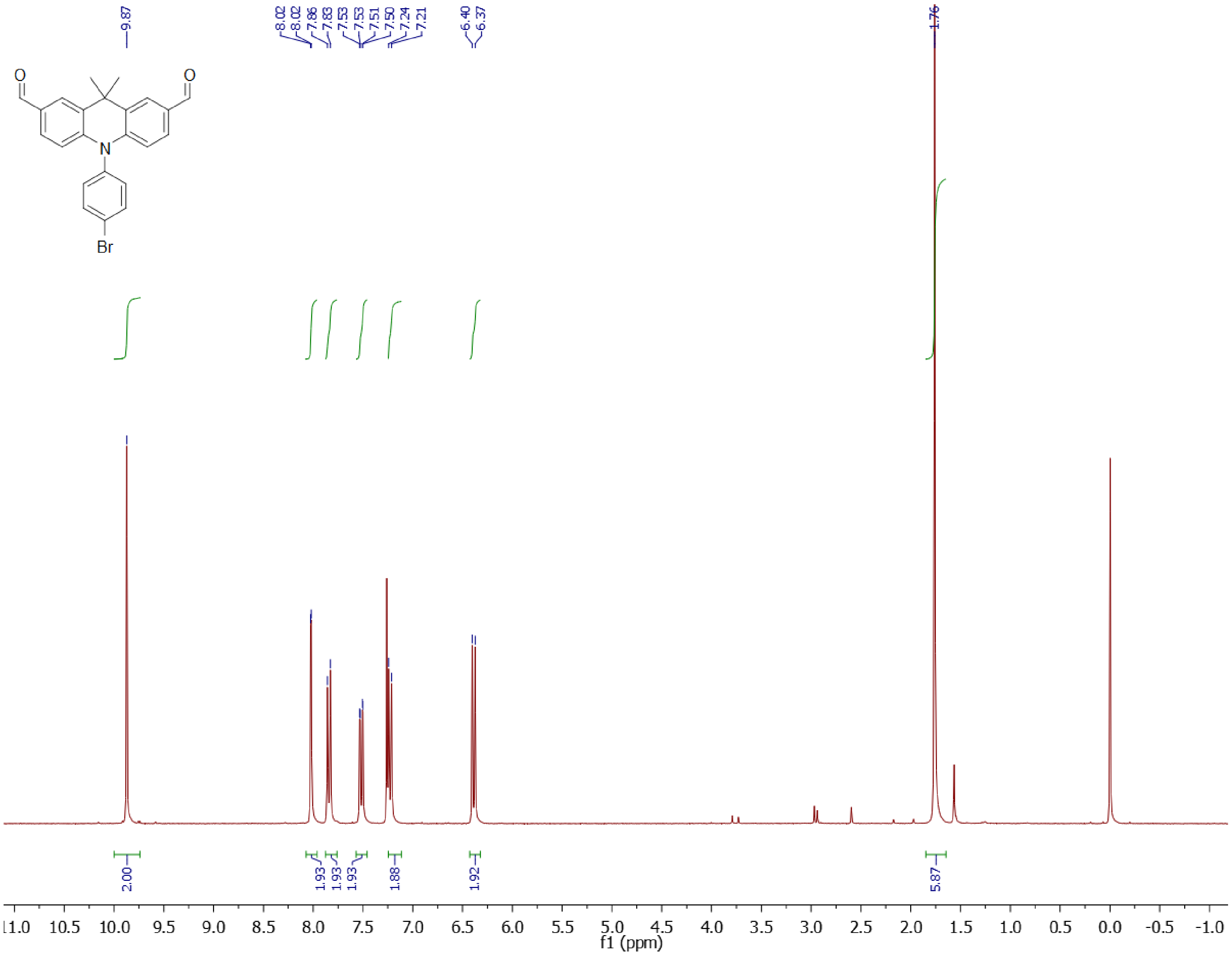

APT NMR spectra of 8-*p* in $CDCl_3$ (75 MHz):

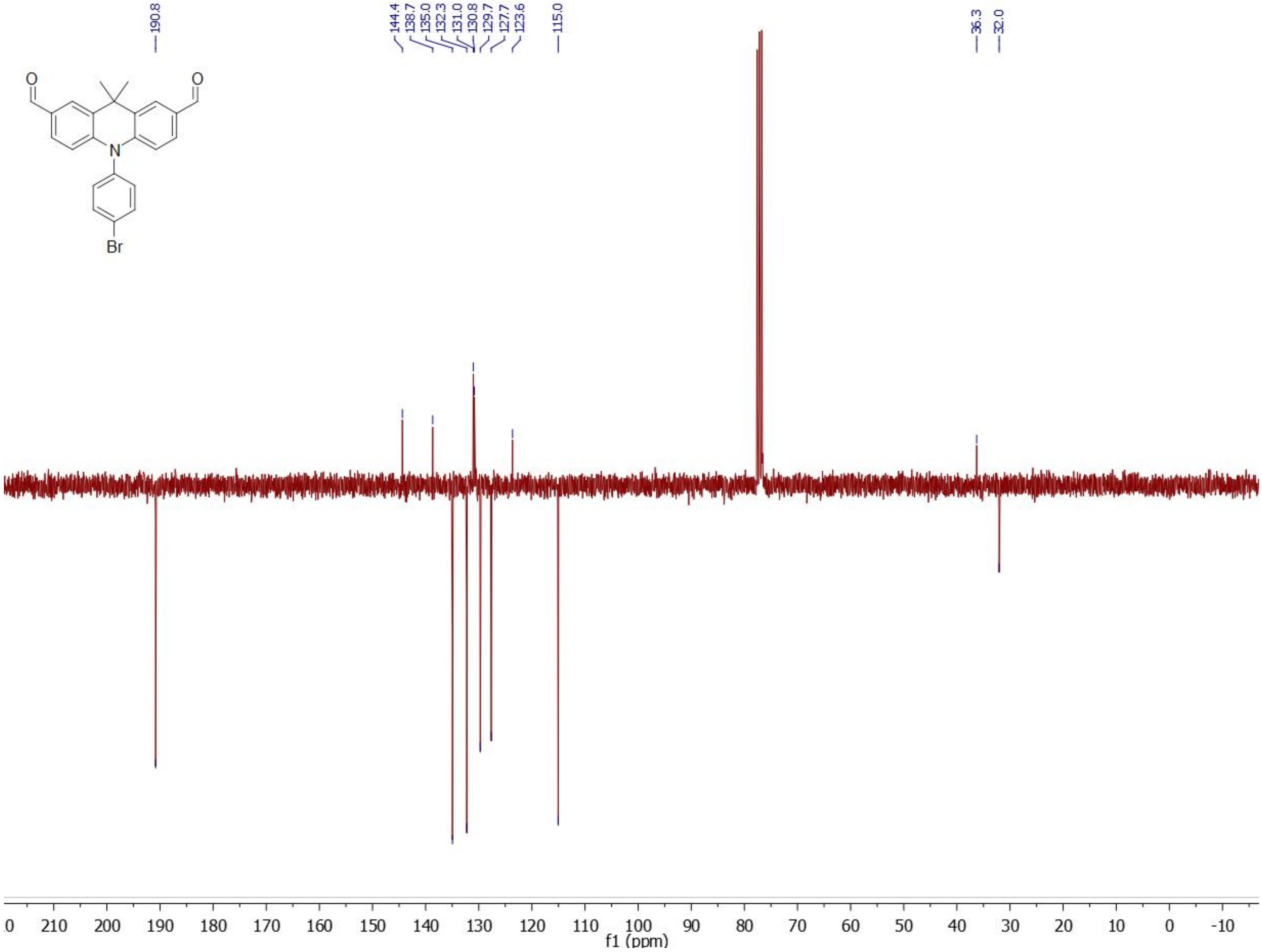

^{1}H NMR spectra of 10-*p* in $CDCl_3$ (300 MHz):

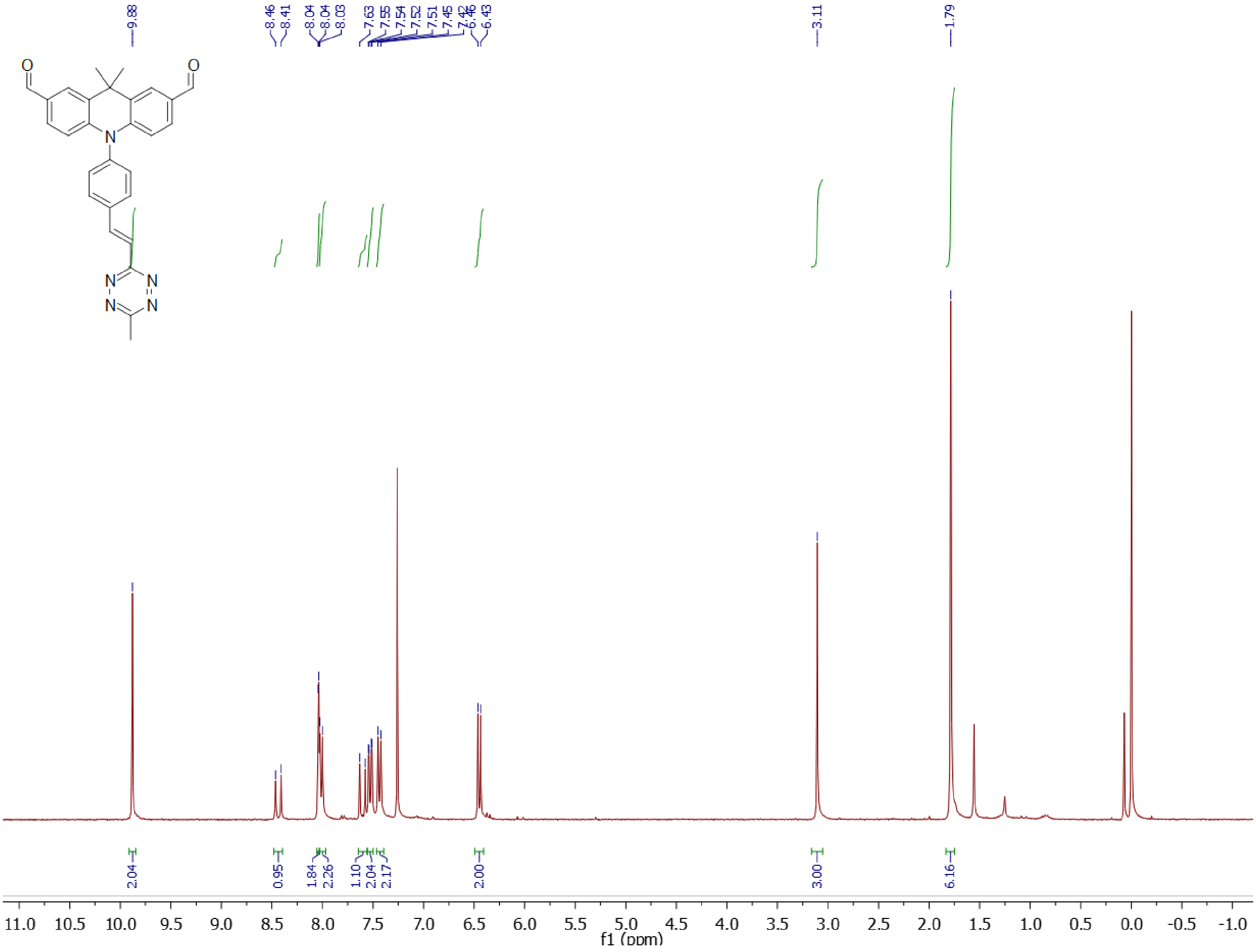

APT NMR spectra of 10-*p* in $CDCl_3$ (75 MHz):

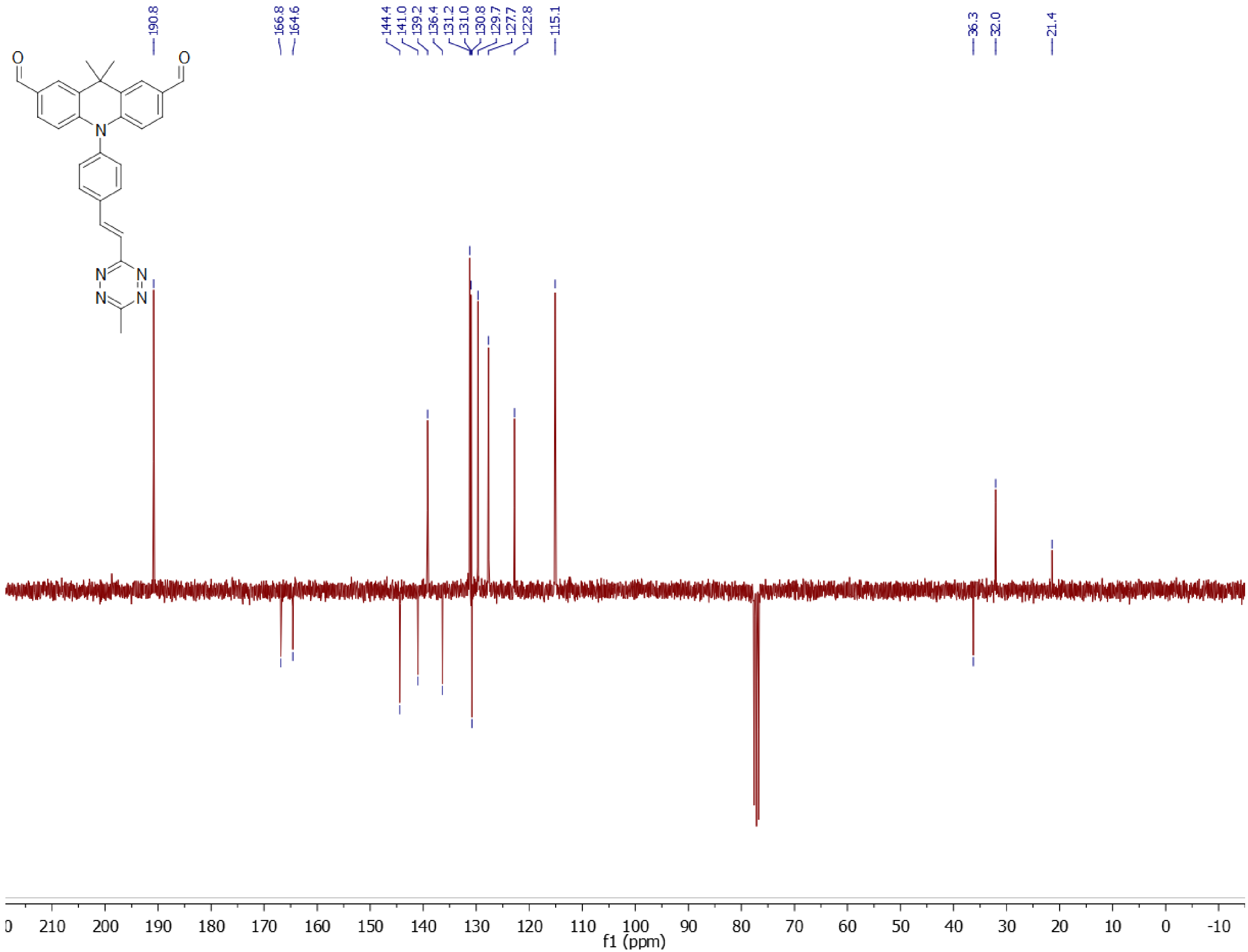

^{1}H NMR spectra of Acri-*p*vi in DMSO-d_6 (300 MHz):

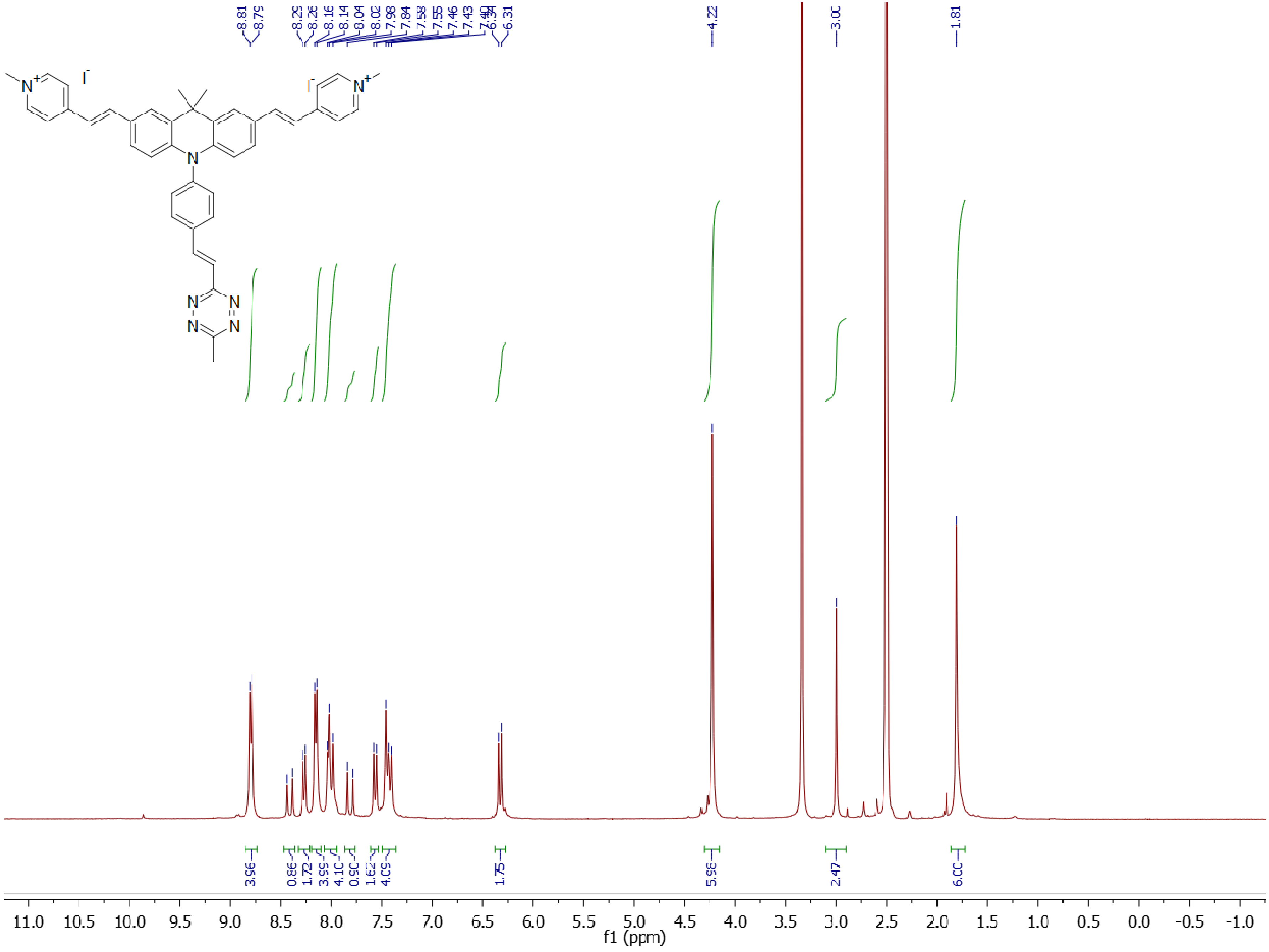

APT NMR spectra of Acri-*p*vi in DMSO-d_6 (75 MHz):

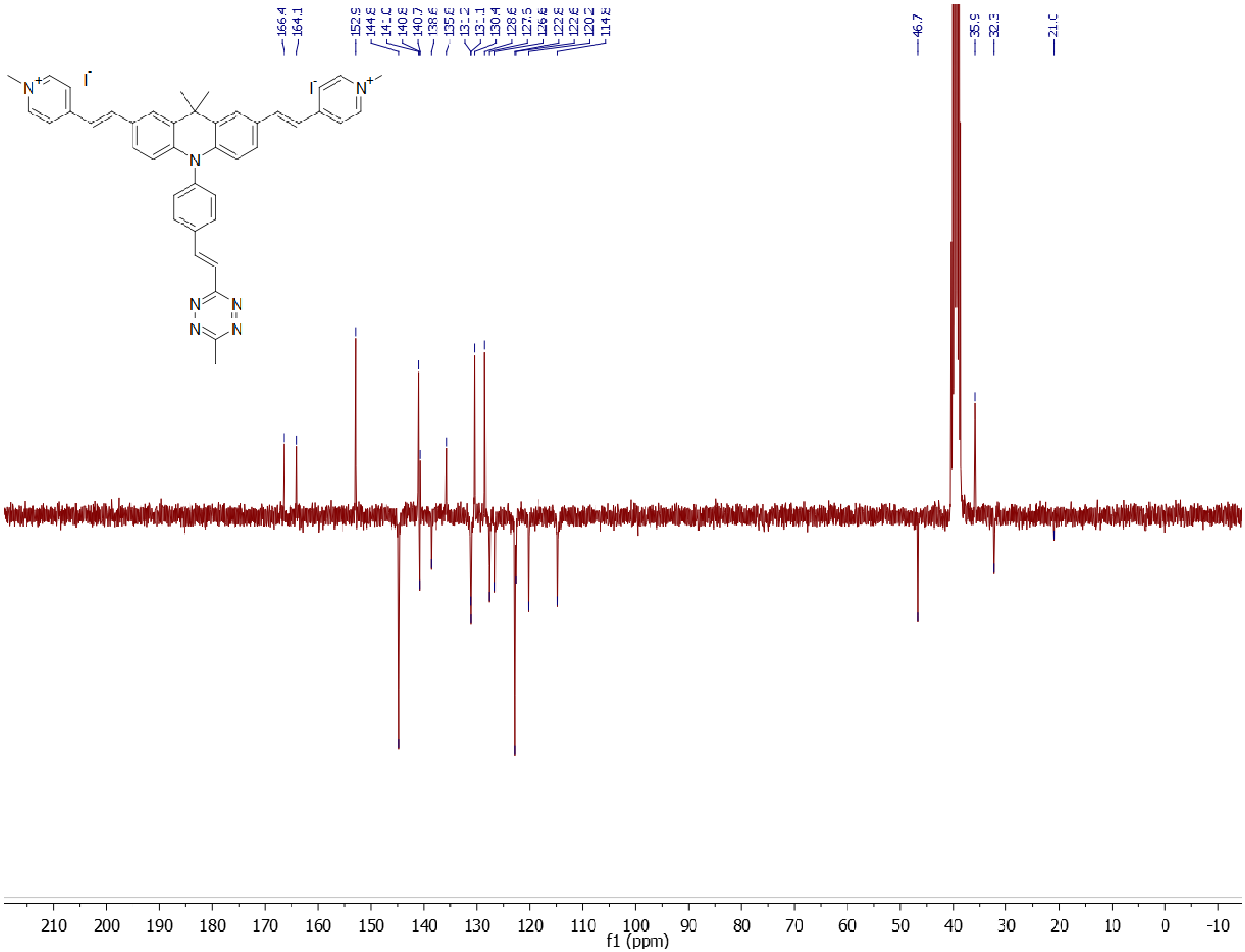

IV. LC/MS of key intermediates

LC-MS spectra (ESI in the positive ion mode) were recorded on a Waters Micromass ZQ instrument coupled to a Waters Alliance Separations Module 2695, equipped with a Luna Omega C18 - 3 µm 100 Å column (3.0 x 50 mm) and a Waters PDA 2998, using the following gradient (Solvent A: H_2O + 0.1% Formic Acid, Solvent B: CH_3CN + 0.1% Formic Acid):

Time (min)	*A (%)*	*B (%)*	*Flow (mL/min)*
0	95	5	0.5
1	95	5	0.5
6	0	100	0.5
8	0	100	0.5
8.5	95	5	0.6
10.5	95	5	0.6
11	95	5	0.5

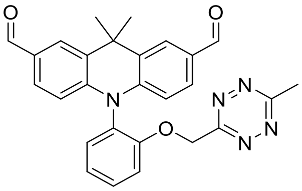

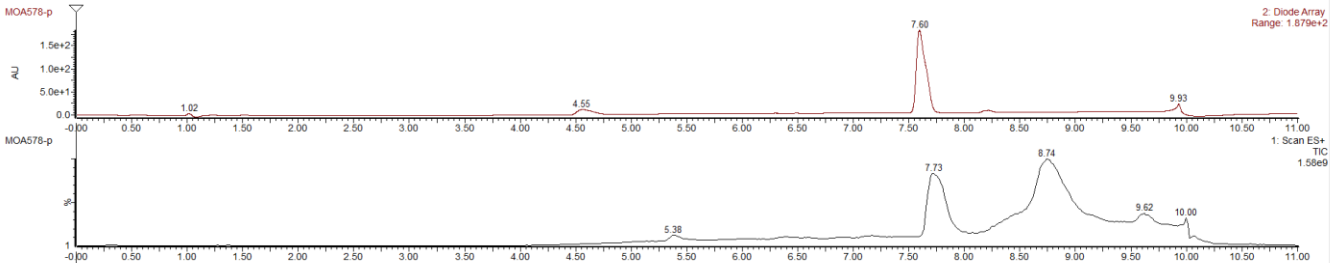

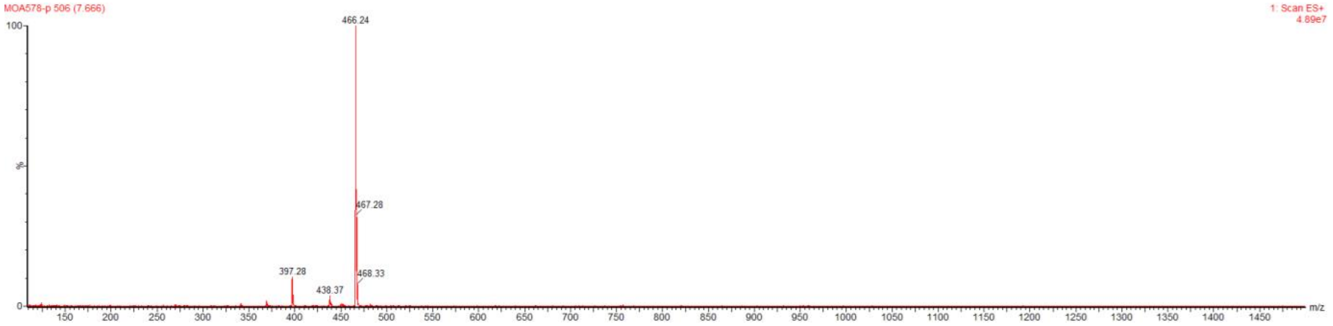

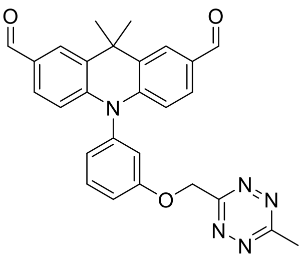

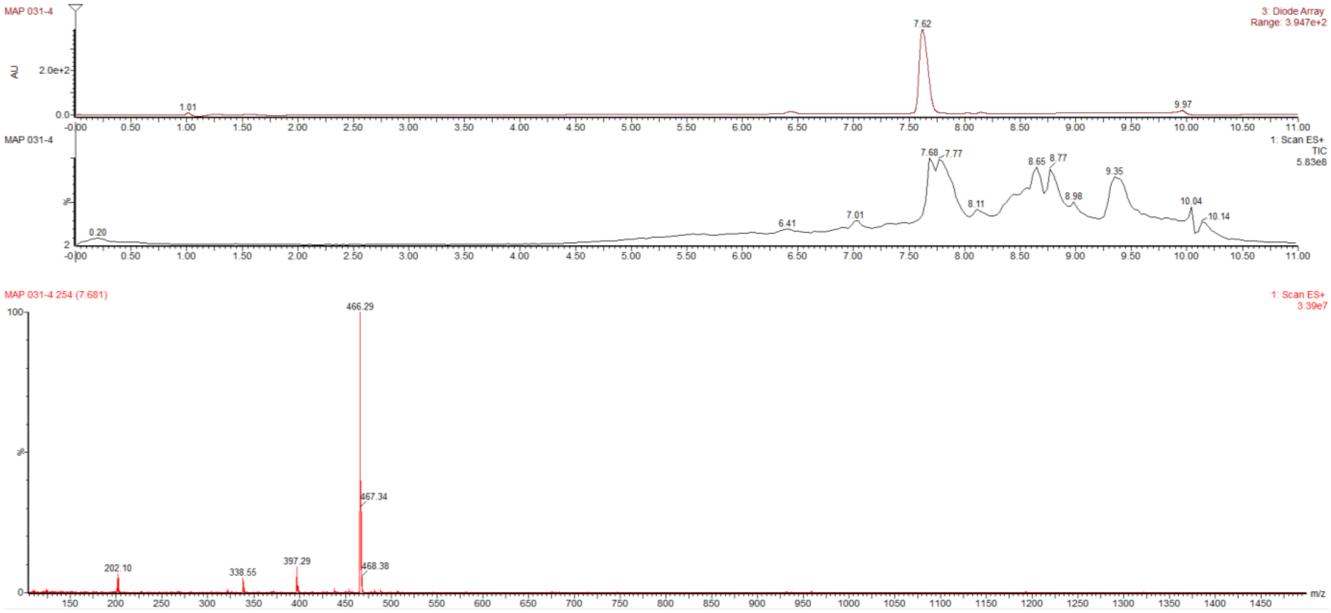
MAP 031-4
3: Diode Array
Range: 3.947e+2
AU
2.0e+2
0.0
7.62
1.01
9.97
MAP 031-4
1: Scan ES+
TIC
5.83e8
7.68
7.77
8.65
8.77
9.35
8.11
8.98
10.04
10.14
6.41
7.01
0.20
MAP 031-4 254 (7.681)
1: Scan ES+
3.39e7
466.29
467.34
468.38
202.10
338.55
397.29
m/z

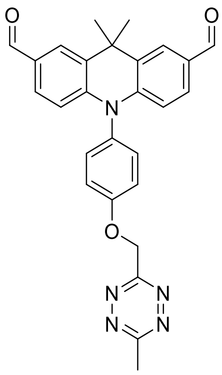

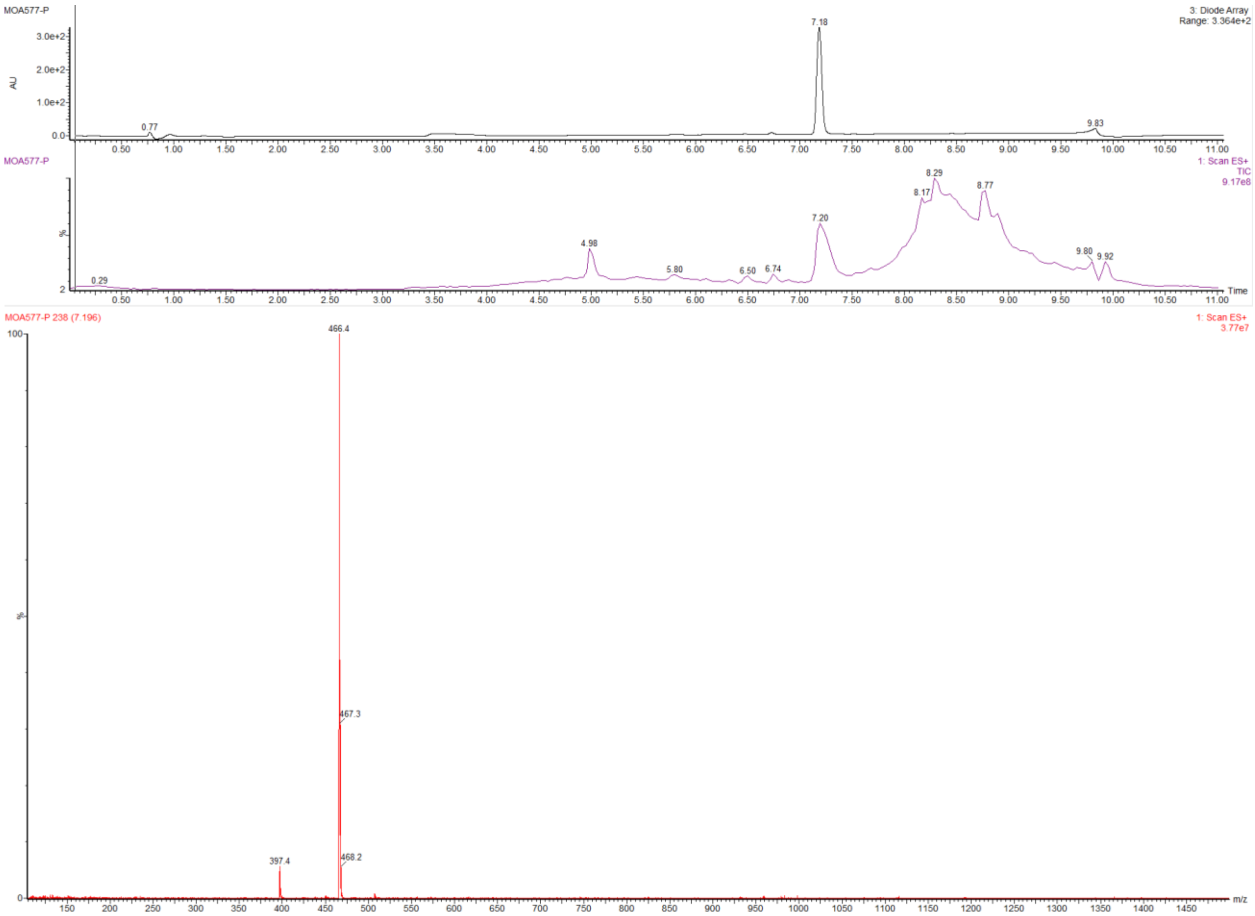
MOA577-P
3: Diode Array
Range: 3.364e+2
AU
7.18
0.77
9.83
MOA577-P
1: Scan ES+
TIC
9.17e8
8.29
8.17
8.77
7.20
4.98
5.80
6.50
6.74
9.80
9.92
0.29
Time
MOA577-P 238 (7.196)
1: Scan ES+
3.77e7
466.4
467.3
397.4
468.2
m/z

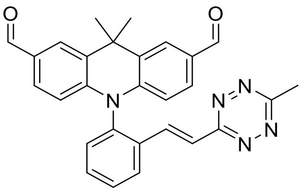
O
O
N
N
N
N
N

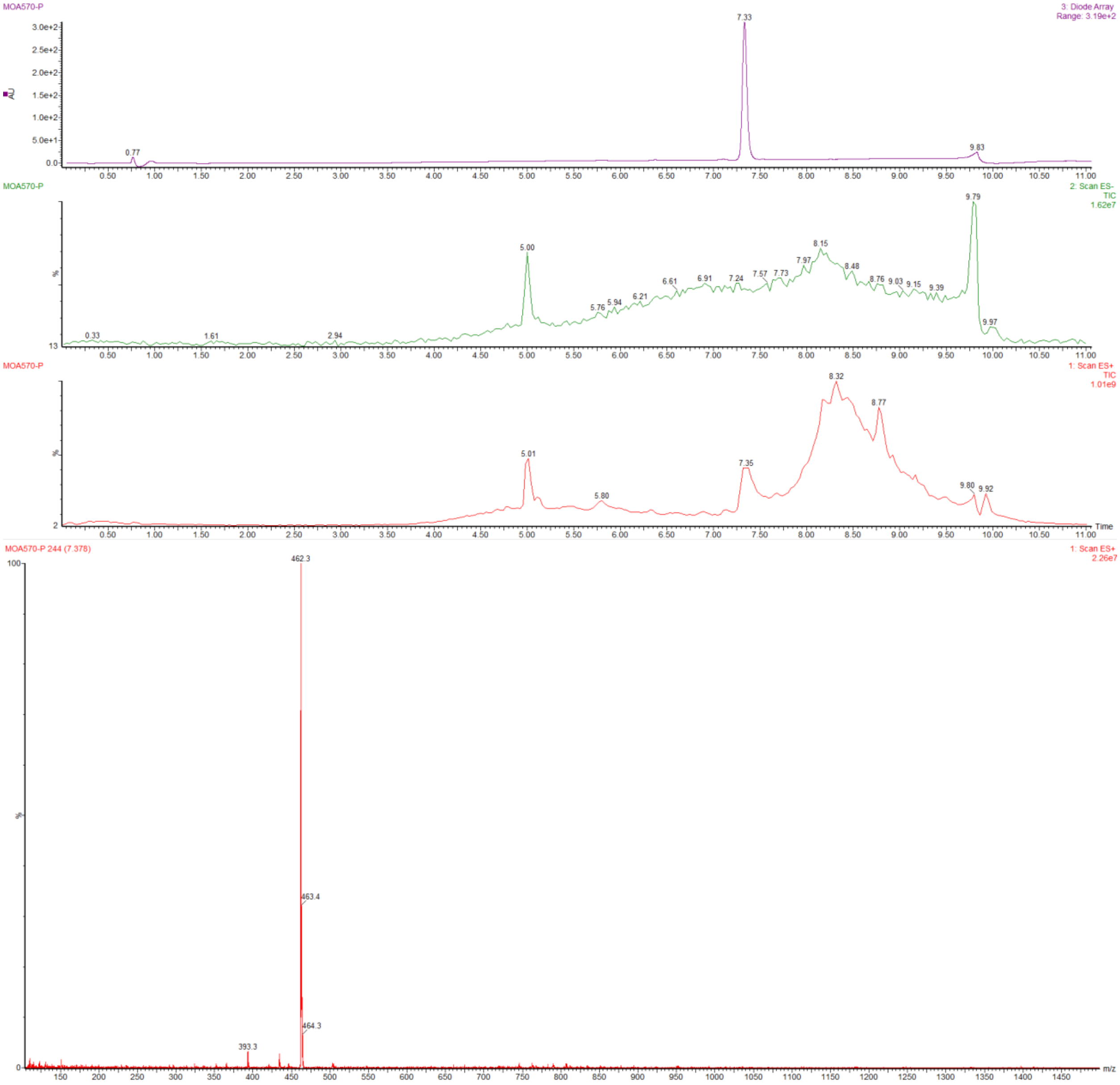
MOA570-P
3: Diode Array
Range: 3.19e+2
AU
7.33
0.77
9.83
2: Scan ES-
TIC
1.62e7
9.79
5.00
8.15
1: Scan ES+
TIC
1.01e9
8.32
8.77
5.01
7.35
5.80
9.80
9.92
Time
MOA570-P 244 (7.378)
1: Scan ES+
2.26e7
462.3
463.4
464.3
393.3
m/z

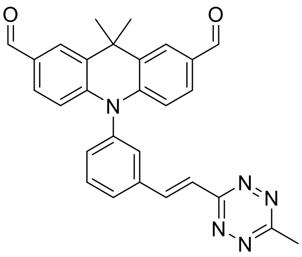
O
O
N
N
N
N
N

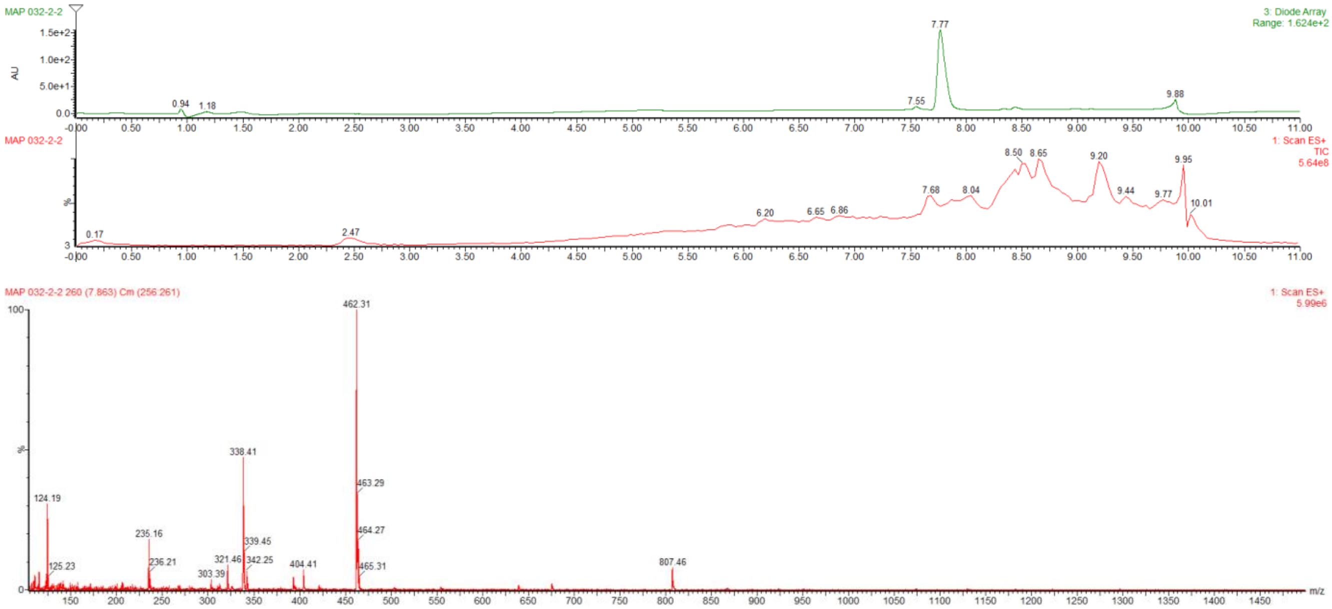
MAP 032-2-2
3: Diode Array
Range: 1.624e+2
AU
7.77
0.94
1.18
7.55
9.88
MAP 032-2-2
1: Scan ES+
TIC
5.64e8
8.50
8.65
9.20
9.95
7.68
8.04
9.44
9.77
10.01
6.20
6.65
6.86
0.17
2.47
MAP 032-2-2 260 (7.863) Cm (256:261)
1: Scan ES+
5.99e6
462.31
338.41
463.29
124.19
235.16
339.45
464.27
321.46
342.25
236.21
404.41
807.46
125.23
303.39
465.31
m/z

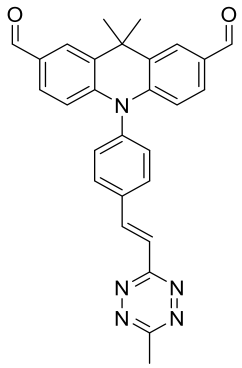

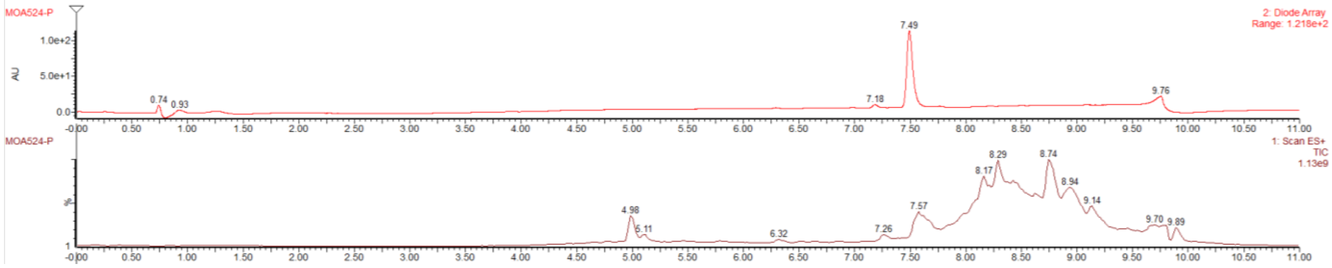
MOA524-P
2: Diode Array
Range: 1.218e+2
AU
7.49
0.74
0.93
7.18
9.76
MOA524-P
1: Scan ES+
TIC
1.13e9
4.98
5.11
6.32
7.26
7.57
8.17
8.29
8.74
8.94
9.14
9.70
9.89

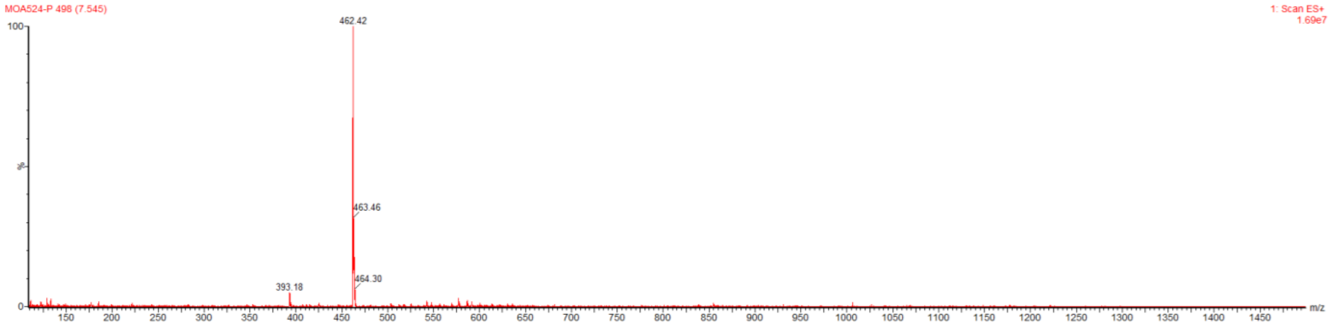
MOA524-P 498 (7.545)
1: Scan ES+
1.69e7
462.42
463.46
464.30
393.18
m/z

V. LC/MS of final compounds

All the fluorogenic probes were repurified by preparative HPLC before fluorescence experiments to ensure a purity > 98 %.

Before HPLC investigations the samples were solubilized in a 1:1 ACN/MeOH mixture and filtered with a 0.45 µm Nylon syringe filter. Preparative HPLC were performed with a quaternary gradient module Waters 2525 coupled with a Sample Manager Injector/Collector (Water W2767, Waters, USA). The W2996 PDA Detector record spectrum between 200 to 500 nm at 20Hz with resolution at 2.4 nm. A XBridge Prep C18 column (30 x 150 mm, 5 µm Waters) was used for the separation with the following gradient (Solvent A: H_2O + 0.1% TFA, Solvent B: ACN + 0.1% TFA, Flow: 40 mL/min):

Time (min)	A (%)	B (%)
**0**	98	2
**2**	98	2
**15**	0	100
**20**	0	100
**20.5**	98	2
**22.5**	98	2

After purification, solvents were removed under vacuum (keeping the bath at room temperature). Compounds were then solubilized in a ACN/MeOH mixture (1:1) and eluted through a small column containing Amberlite IRA402-Cl to give the expected product with chloride counter ions.

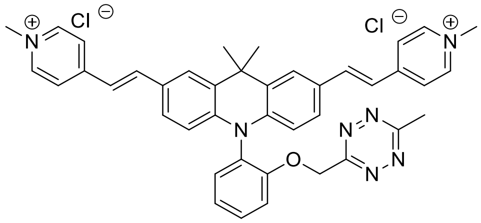
Cl
Cl
N
N
N
N
O
N
N
N

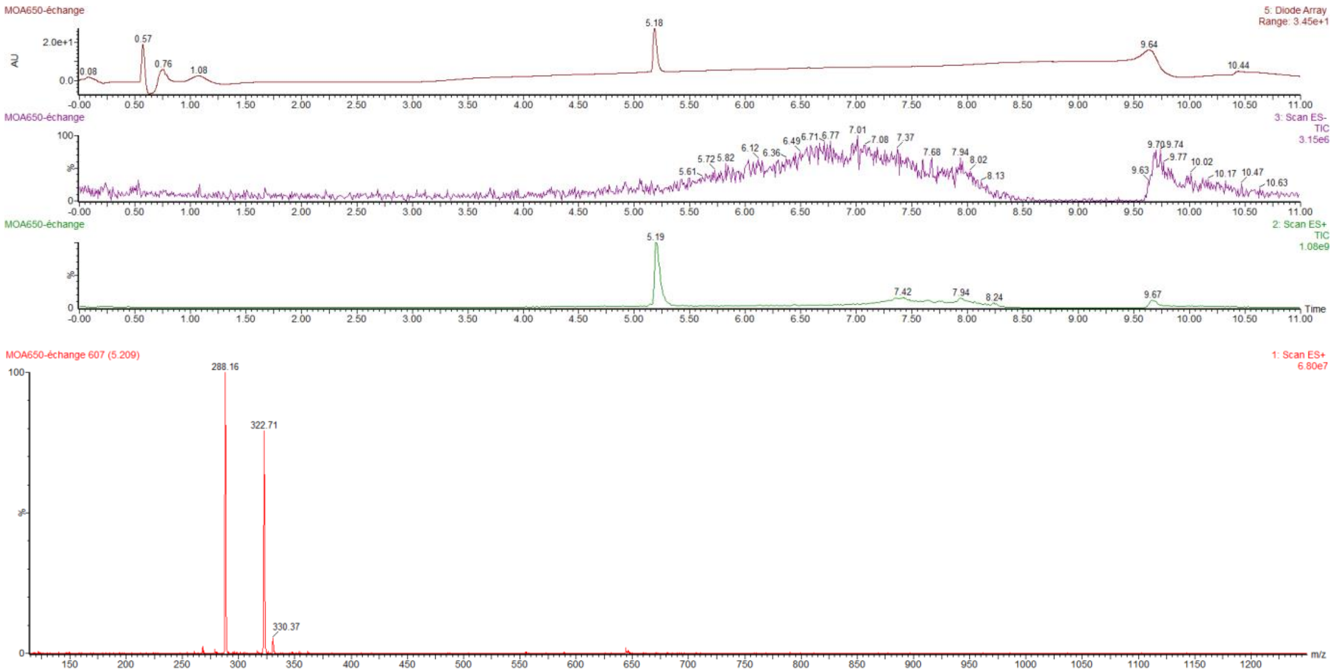
MOA650-échange
5: Diode Array
Range: 3.45e+1
AU
5.18
9.64
10.44
0.57
0.76
1.08
MOA650-échange
3: Scan ES-
TIC
3.15e6
MOA650-échange
2: Scan ES+
TIC
1.08e9
5.19
7.42
7.94
8.24
9.67
Time
MOA650-échange 607 (5.209)
1: Scan ES+
6.80e7
288.16
322.71
330.37
m/z

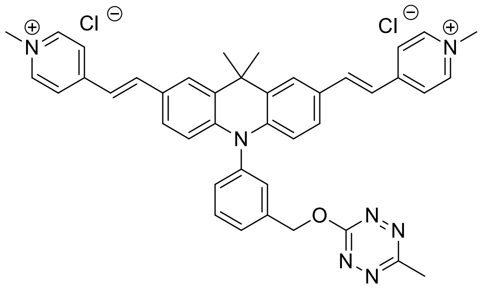
Cl
Cl
N
N
N
O
N
N
N
N
N

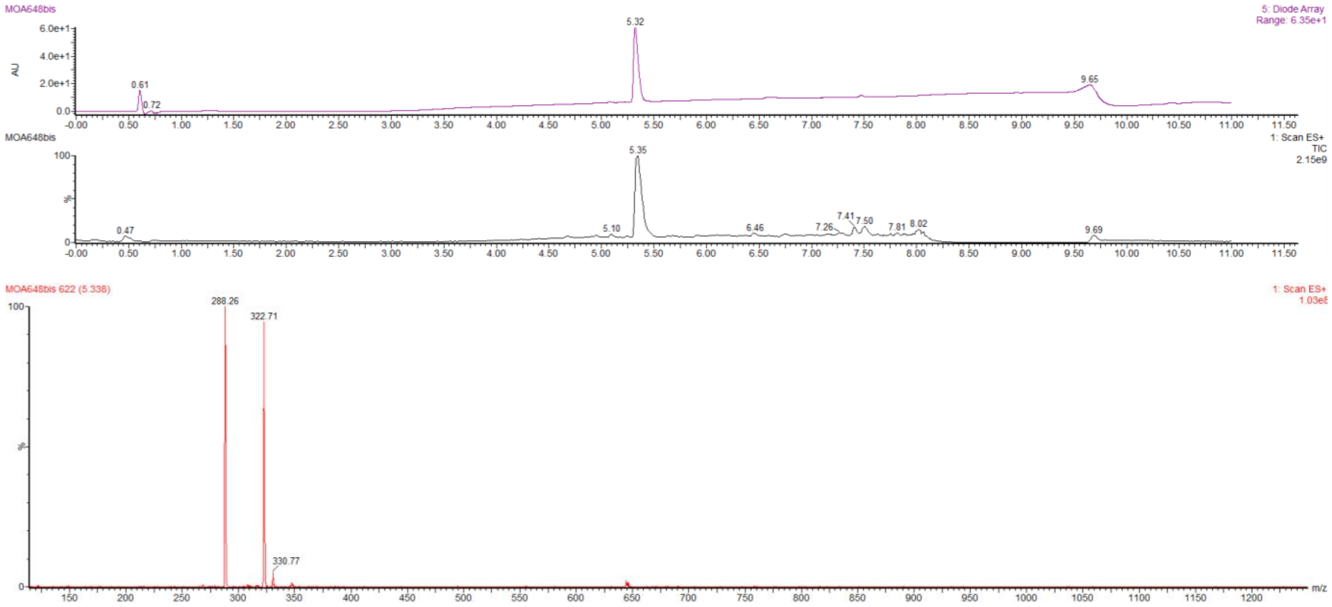
MOA648bis
5: Diode Array
Range: 6.35e+1
AU
5.32
0.61
0.72
9.65
MOA648bis
1: Scan ES+
TIC
2.15e9
5.35
0.47
5.10
6.46
7.26
7.41
7.50
7.81
8.02
9.69
MOA648bis 622 (5.338)
1: Scan ES+
288.26
322.71
330.77
m/z

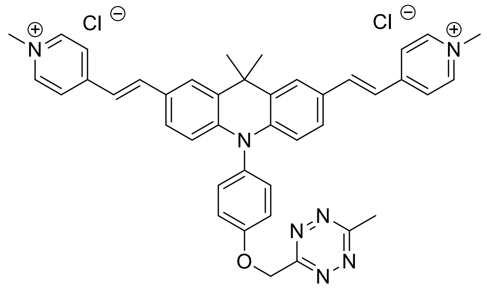
Cl
Cl
N
N
O
N
N
N
N
N
N

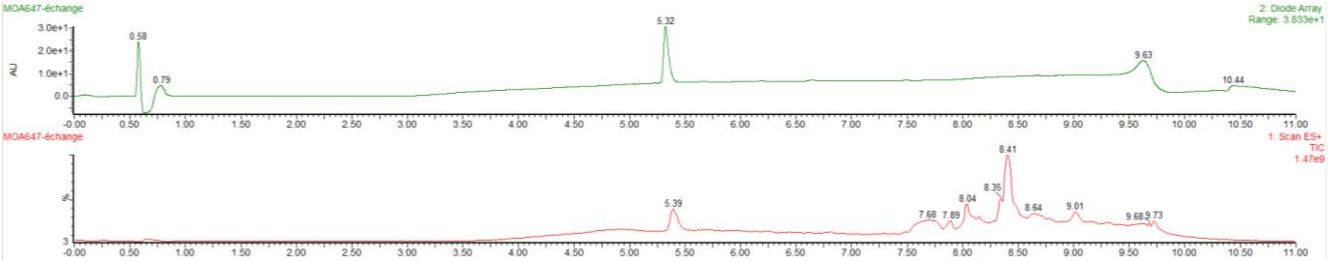
MOA647-échange
2: Diode Array
Range: 3.833e+1
AU
0.58
0.79
5.32
9.63
10.44
MOA647-échange
1: Scan ES+
TIC
1.47e9
%
5.39
7.68
7.89
8.04
8.35
8.41
8.64
9.01
9.68
9.73

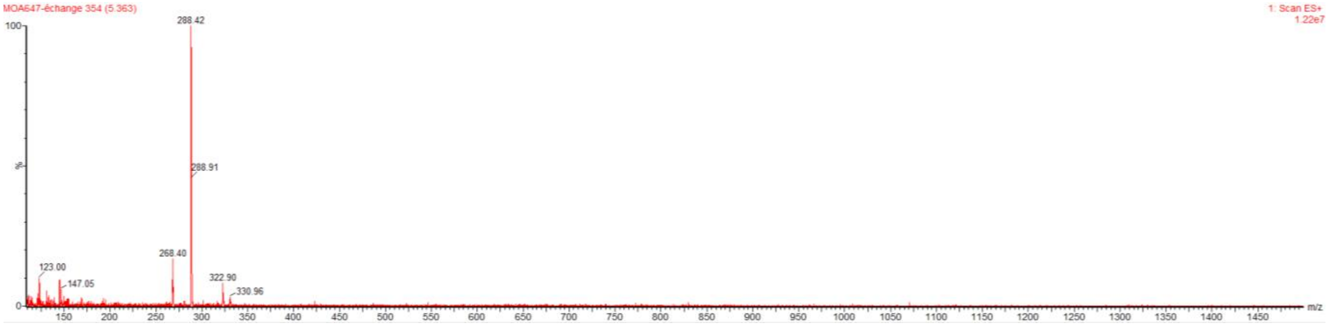
MOA647-échange 354 (5.363)
1: Scan ES+
1.22e7
%
288.42
288.91
268.40
123.00
147.05
322.90
330.96
m/z

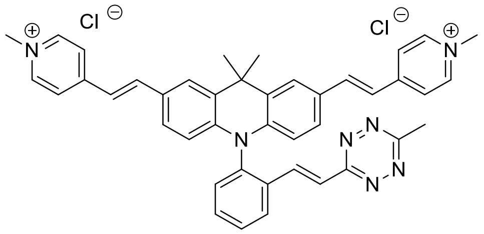
Cl ⊖
Cl ⊖
N⊕
N⊕
N
N N
N N

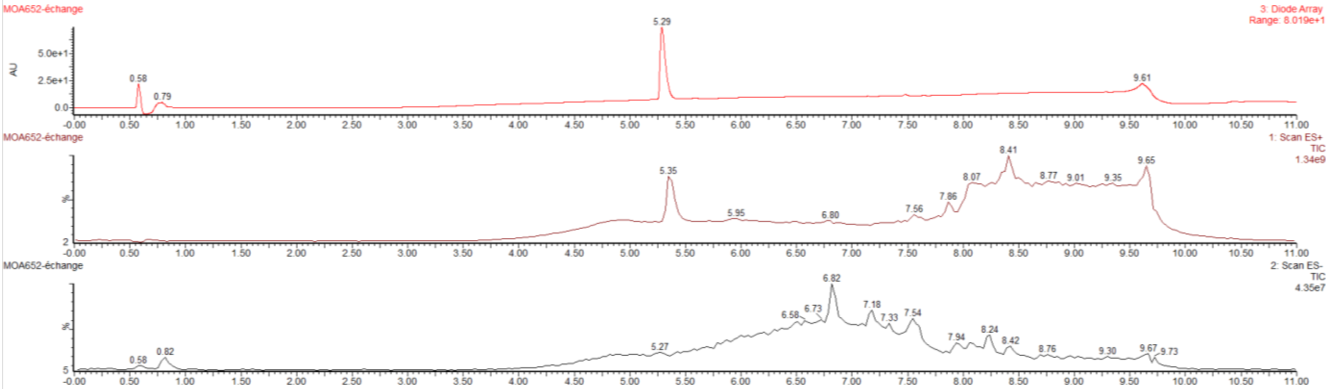
MOA652-échange
3: Diode Array
Range: 8.019e+1
AU
5.29
0.58
0.79
9.61
MOA652-échange
1: Scan ES+
TIC
1.34e9
5.35
5.95
6.80
7.56
7.86
8.07
8.41
8.77
9.01
9.35
9.65
MOA652-échange
2: Scan ES-
TIC
4.35e7
0.58
0.82
5.27
6.58
6.73
6.82
7.18
7.33
7.54
7.94
8.24
8.42
8.76
9.30
9.67
9.73

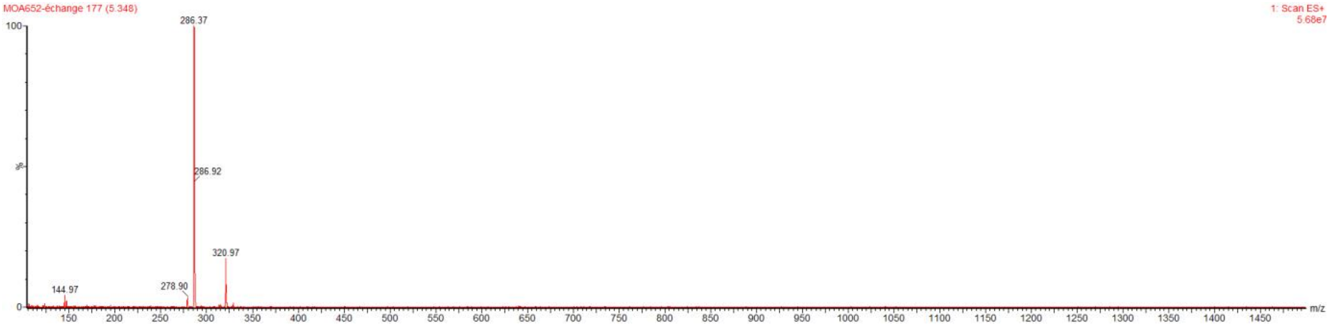
MOA652-échange 177 (5.348)
1: Scan ES+
5.68e7
286.37
286.92
320.97
278.90
144.97
m/z

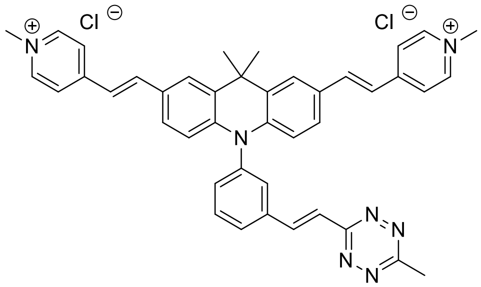
Cl ⊖
Cl ⊖
N
N
N
N
N
N
N
N

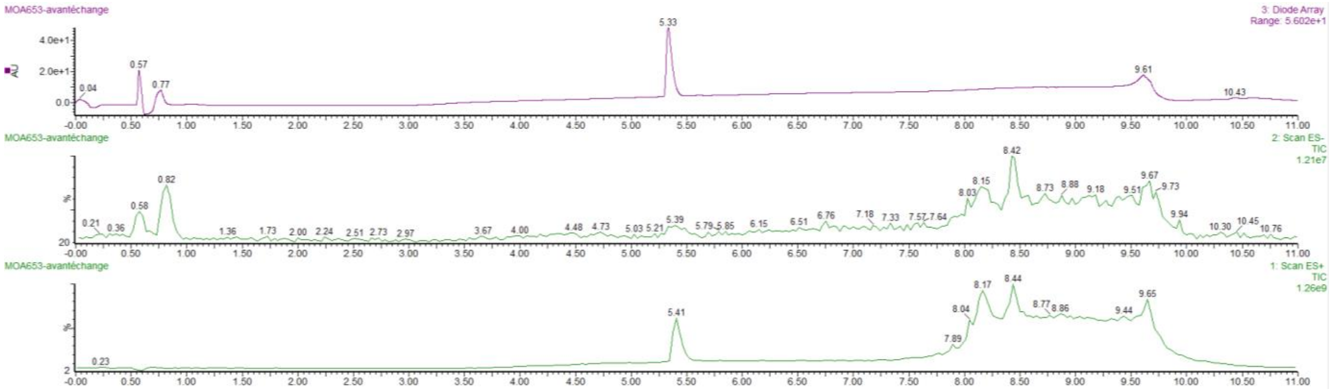
MOA653-avantéchange
3: Diode Array
Range: 5.602e+1
5.33
0.57
0.77
0.04
9.61
10.43
MOA653-avantéchange
2: Scan ES-
TIC
1.21e7
8.42
0.82
0.58
MOA653-avantéchange
1: Scan ES+
TIC
1.26e9
5.41
8.17
8.44
9.65

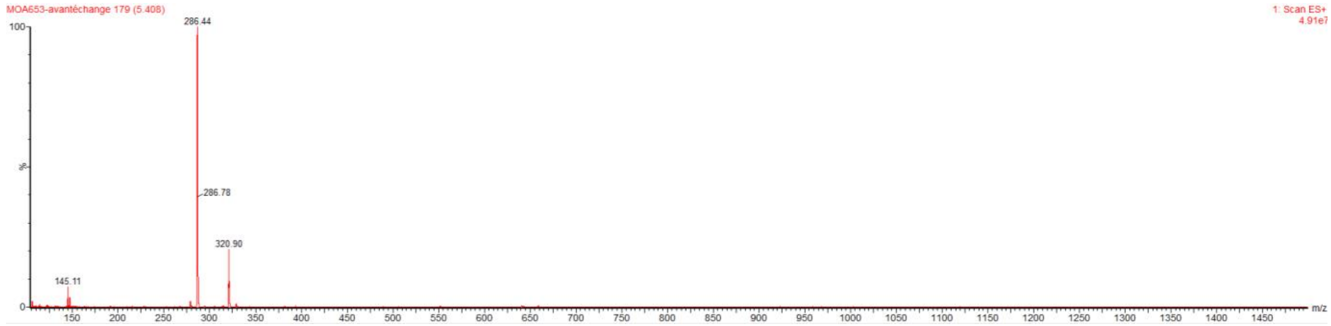
MOA653-avantéchange 179 (5.408)
1: Scan ES+
4.91e7
286.44
286.78
320.90
145.11
m/z

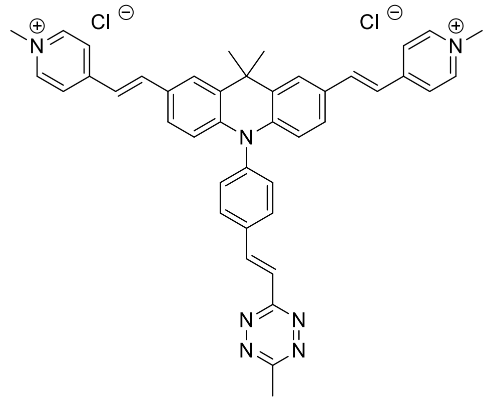
Cl ⊖
Cl ⊖
N
N
N
N
N
N
N

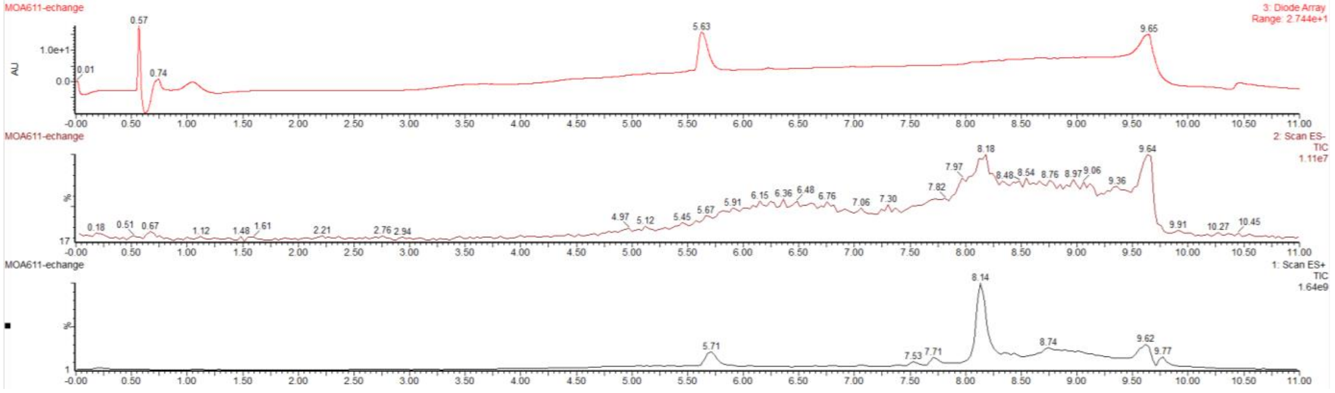
MOA611-echange
3: Diode Array
Range: 2.744e+1
AU
1.0e+1
0.0
0.01
0.57
0.74
5.63
9.65
MOA611-echange
2: Scan ES-
TIC
1.11e7
%
17
0.18
0.51
0.67
1.12
1.48
1.61
2.21
2.76
2.94
4.97
5.12
5.45
5.67
5.91
6.15
6.36
6.48
6.76
7.06
7.30
7.82
7.97
8.18
8.48
8.54
8.76
8.97
9.06
9.36
9.64
9.91
10.27
10.45
MOA611-echange
1: Scan ES+
TIC
1.64e9
%
1
5.71
7.53
7.71
8.14
8.74
9.62
9.77

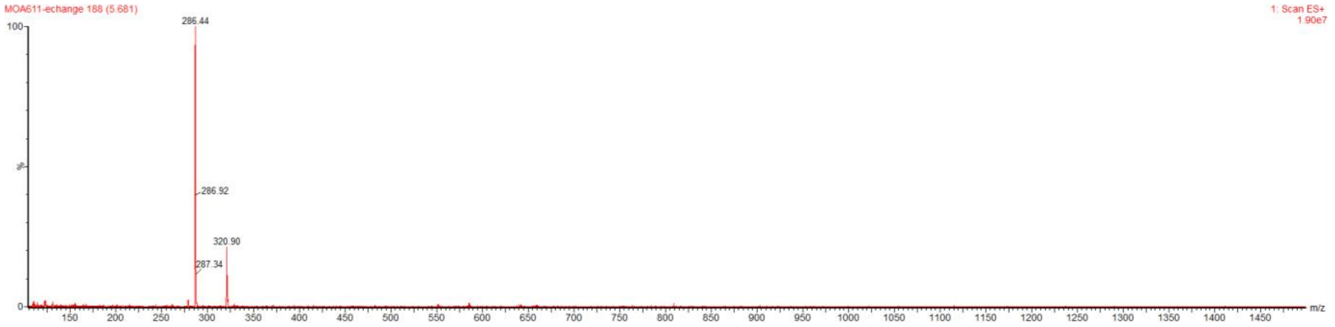
MOA611-echange 188 (5.681)
1: Scan ES+
1.90e7
100
%
0
286.44
286.92
287.34
320.90
m/z

VI. Stability of Acri-*ovi* in PBS at 37 °C

t = 0 min

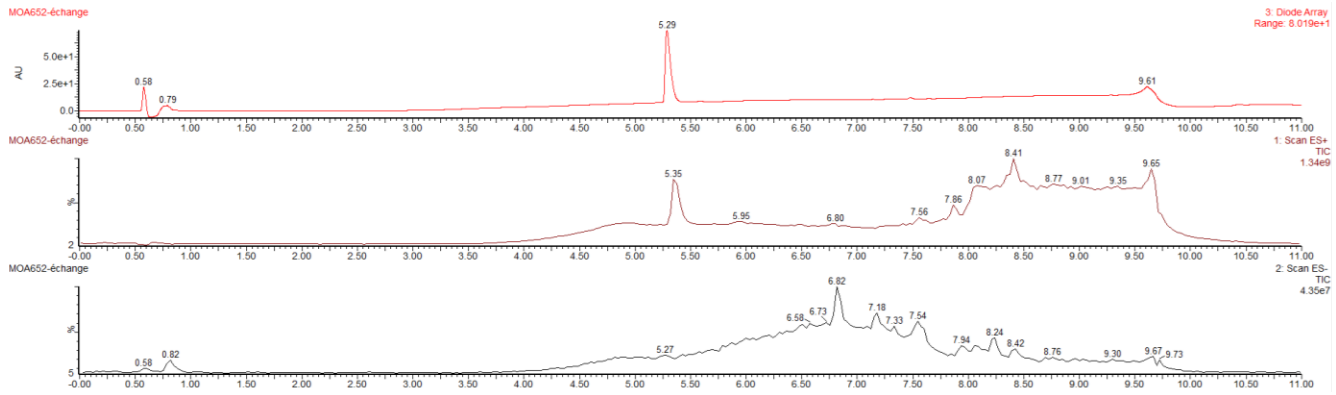

t = 18 h

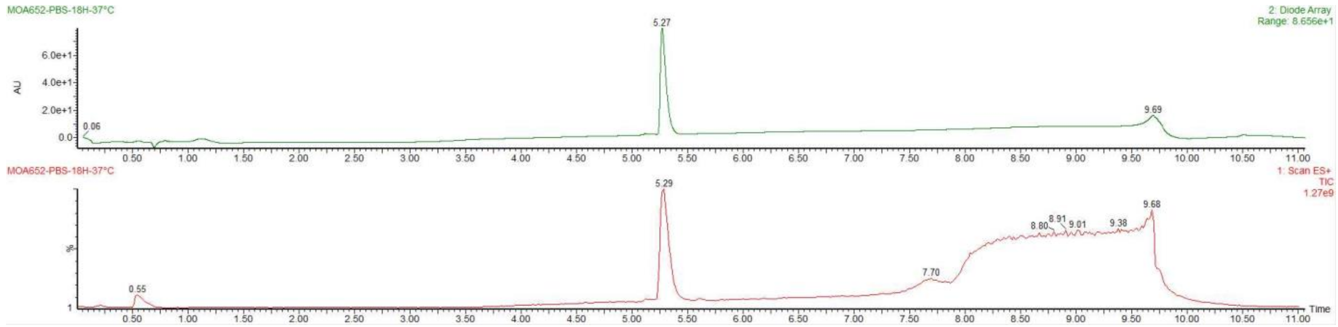

VII. LC/MS of clicked products

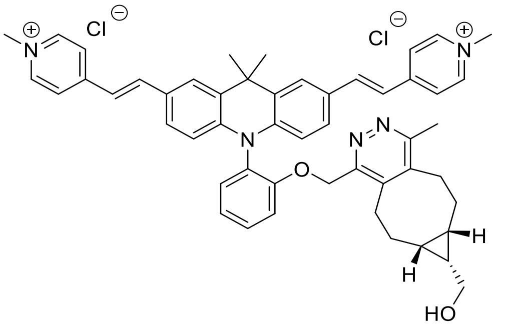

Chemical Formula: $C_{51}H_{53}N_5O_2^{2+}$
m/z: 383.7094

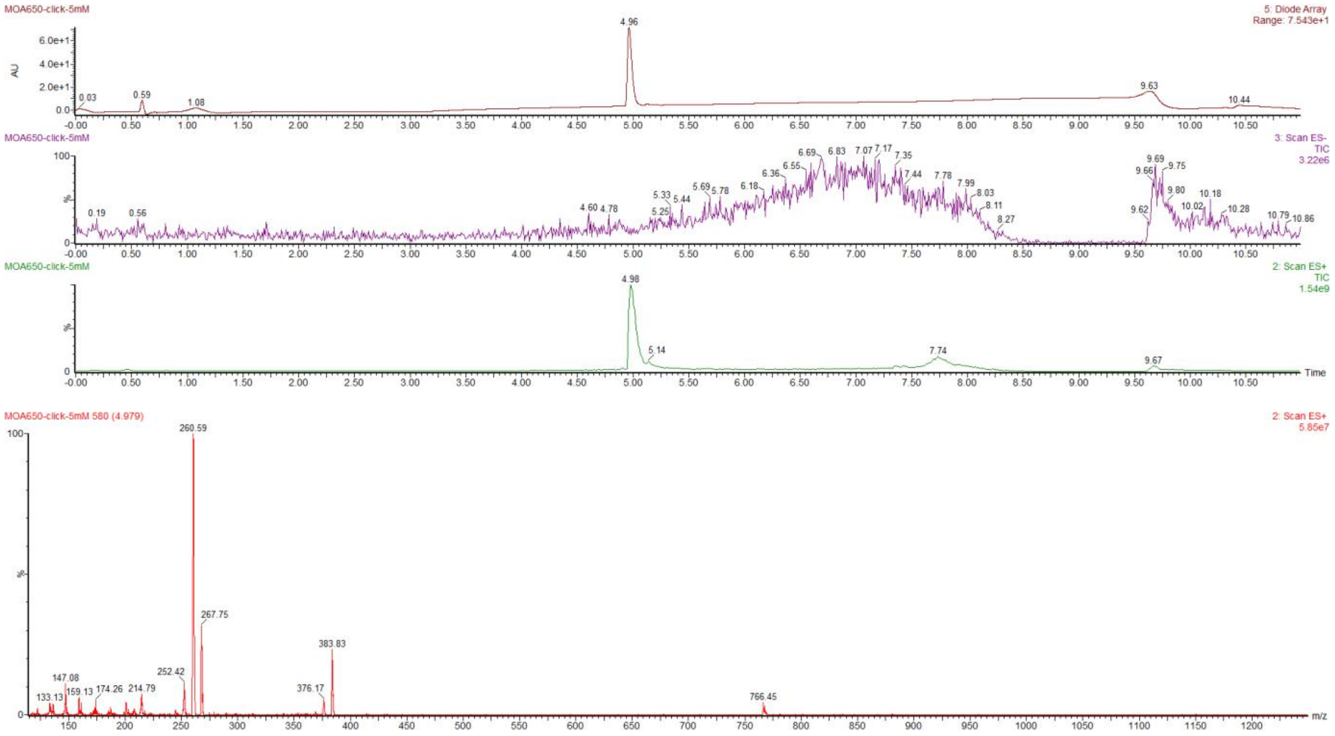

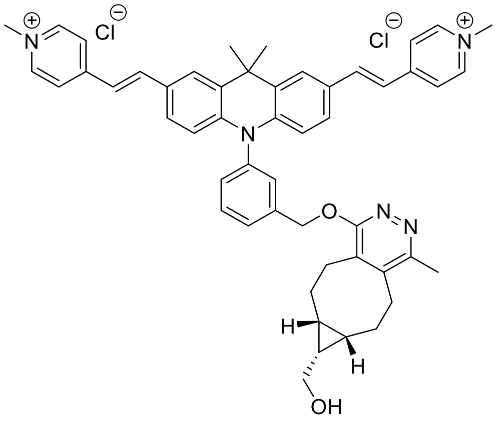

Chemical Formula: $C_{51}H_{53}N_5O_2^{2+}$

m/z: 383.7094

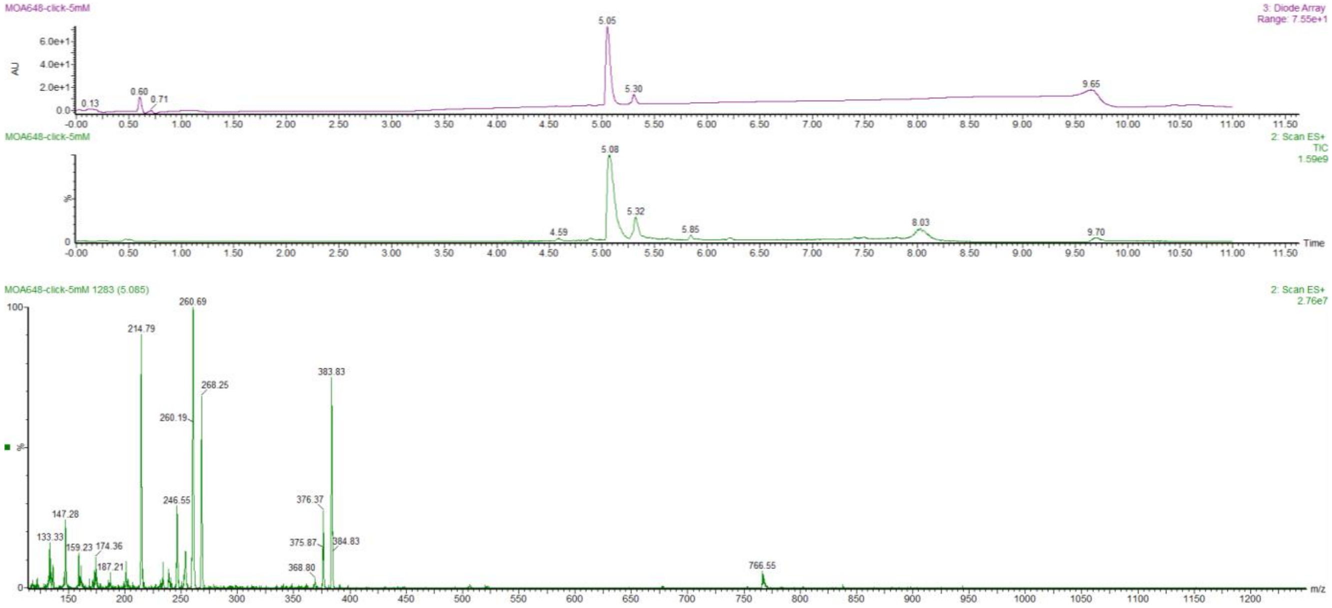

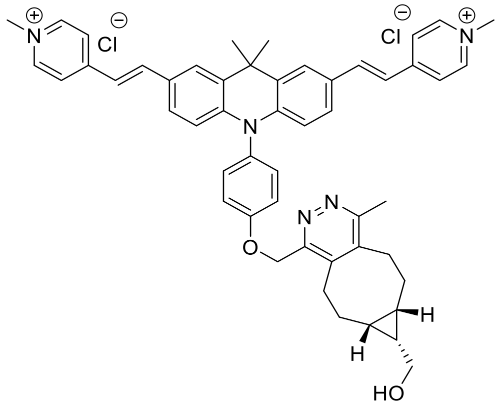

Chemical Formula: $C_{51}H_{53}N_5O_2^{2+}$
m/z: 383.7094

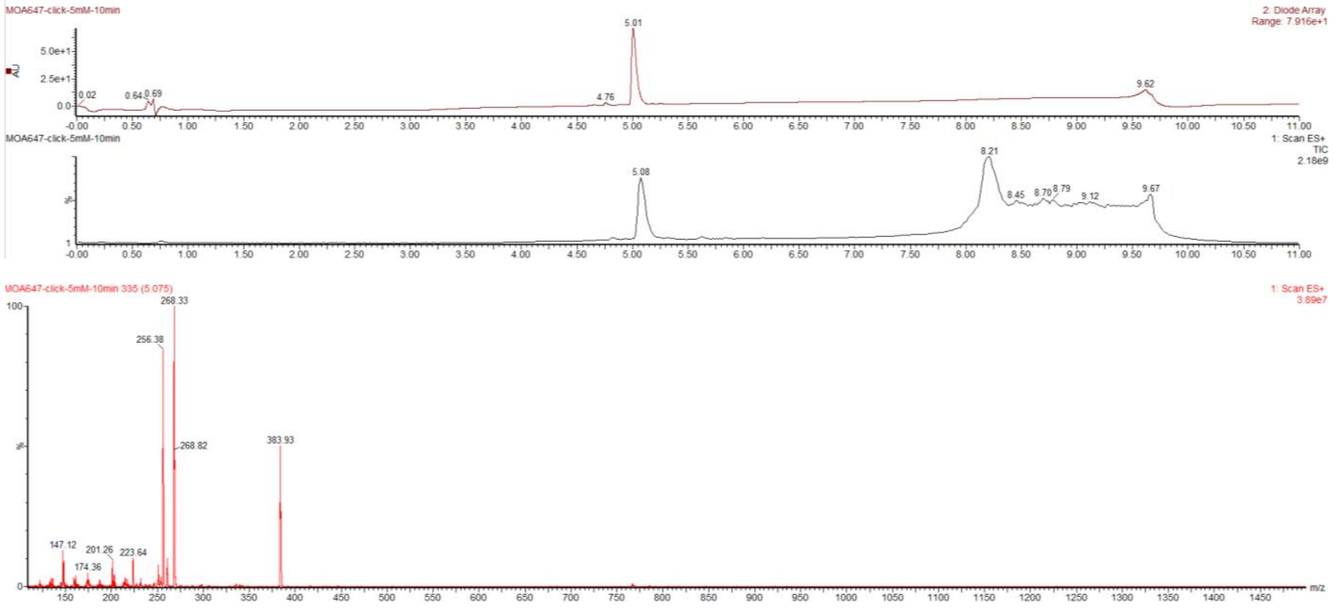

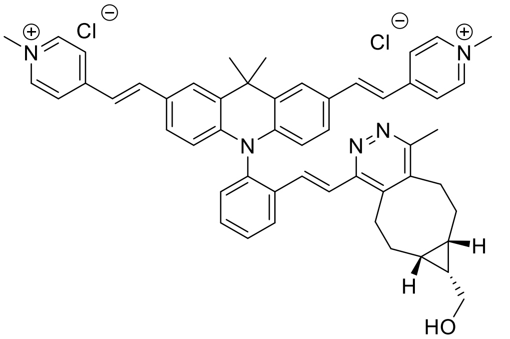

Chemical Formula: $C_{52}H_{53}N_5O^{2+}$
m/z: 381.7120

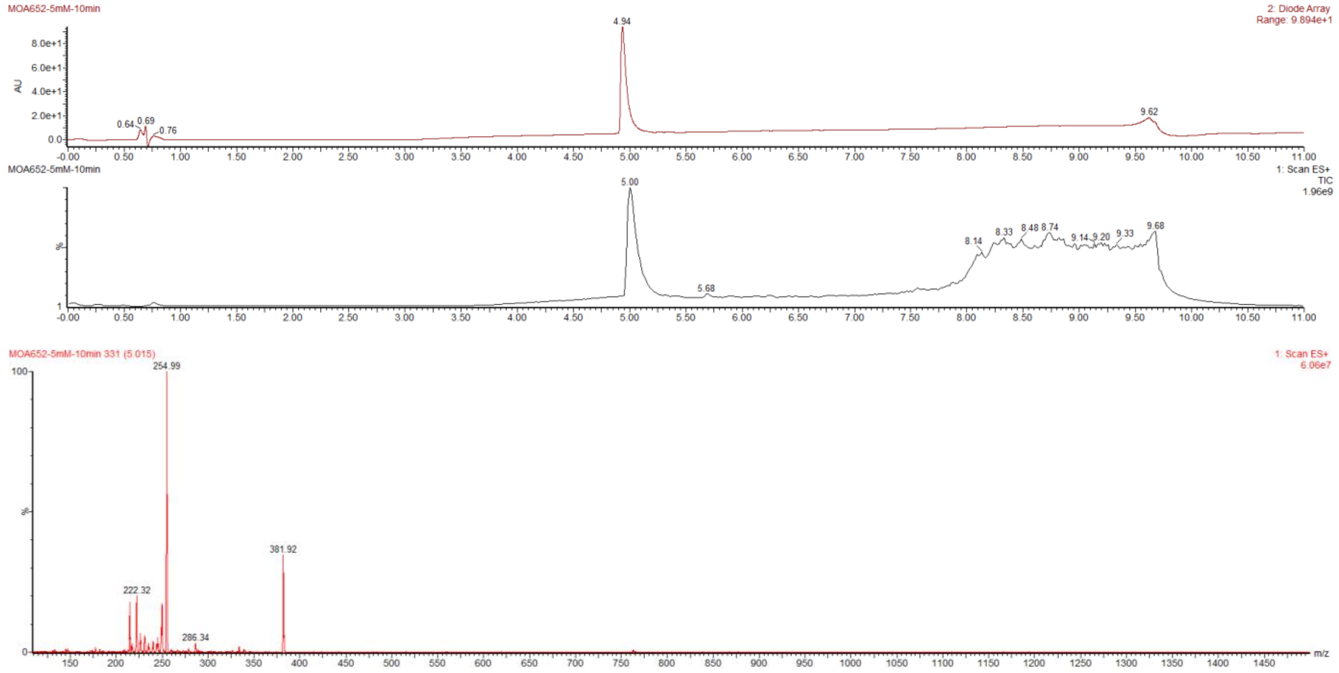

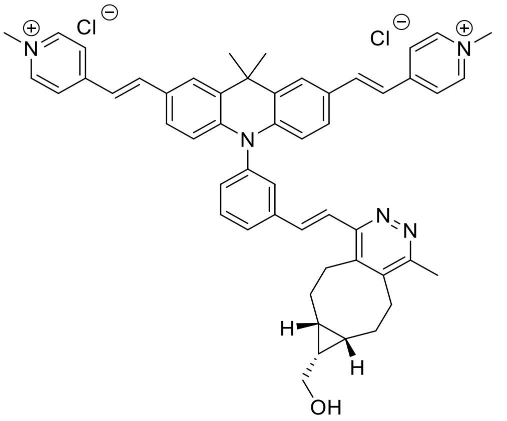

Chemical Formula: $C_{52}H_{53}N_5O^{2+}$
m/z: 381.7120

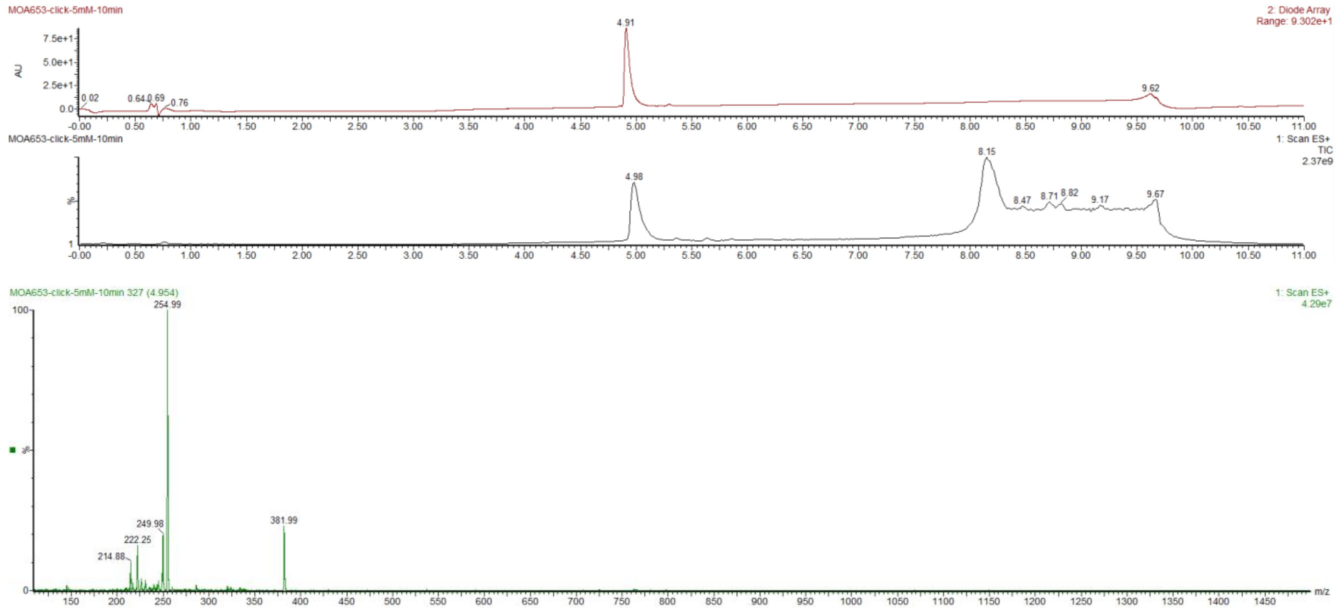

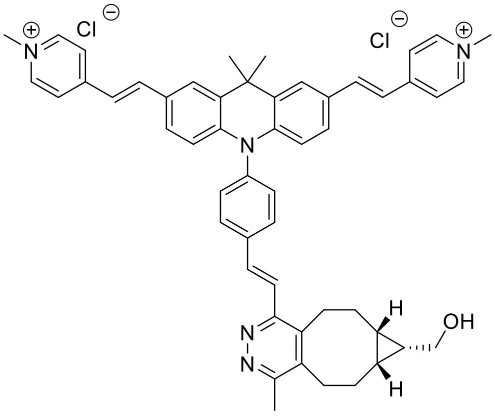

Chemical Formula: $C_{52}H_{53}N_5O^{2+}$
m/z: 381.7120

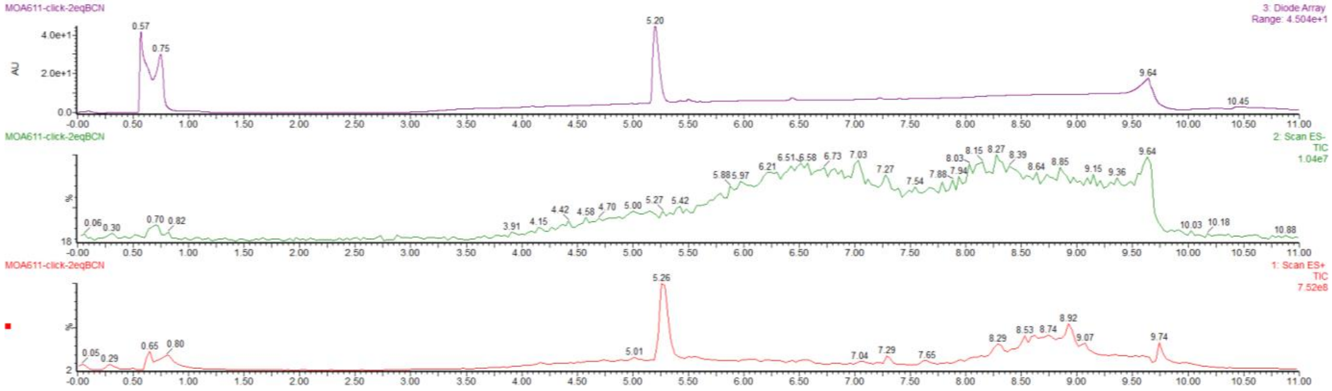

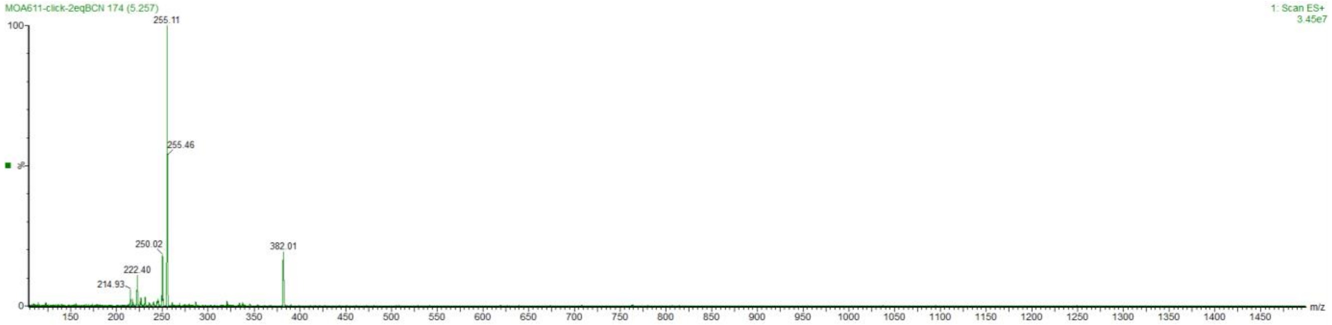

VIII. Photophysical measurements

Stock solutions were prepared in ACN/MeOH 1:1 and stored at -20 °C. Measurements were performed in 0.5 or 1 mL Hellma quartz cuvette (material code QS blue, 200– 2000 nm). Fluorescence and UV–visible spectra were respectively recorded using Spex FluoroMax-3 JobinYvon Horiba and Hitachi U-2900 apparatus. Measurements were performed at room temperature with solutions of A < 0.1 to avoid re-absorption of the emitted light, and data were corrected with a blank. Fluorescence quantum yields were measured according to the Crosby method using Rhodamine 6G in EtOH (λ_{ex} = 488 nm, Φ = 0.94) as reference.

Table S2: Photophysical properties of fluorogenic probes in cacodylate buffer (100 mM NaCl, pH = 7.4)

Compound	$\lambda_{MAX, abs}$ [nm]	$\lambda_{MAX, em}$ [nm]	ε [$M^{-1}cm^{-1}$]	Φ_F
**Acri-Py**	483	665	56,800	<0.005
**Acri-*o*vi**<br>(Clicked)	484<br>(490)	647<br>(648)	61,000<br>(67,720)	<0.005<br>(<0.005)
**Acri-*m*vi**<br>(Clicked)	485<br>(490)	650<br>(625)	52,600<br>(57,690)	<0.005<br>(<0.005)
**Acri-*p*vi**<br>(Clicked)	483<br>(490)	648<br>(650)	47,300<br>(51,100)	<0.005<br>(<0.005)
**Acri-*o*et**<br>(Clicked)	486<br>(490)	642<br>(644)	56,300<br>(55,840)	<0.005<br>(<0.005)
**Acri-*m*et**<br>(Clicked)	485<br>(485)	638<br>(643)	51,800<br>(54,130)	<0.005<br>(<0.005)
**Acri-*p*et**<br>(Clicked)	487<br>(490)	652<br>(646)	45,900<br>(45,940)	<0.005<br>(<0.005)

Table S3: Photophysical properties of fluorogenic probes and their Click adducts[a] in aqueous buffer with BSA[b].
[a] Properties of cycloadducts are presented in parentheses. [b] Measurements were performed in 10 mM sodium cacodylate buffer (100mM NaCl, pH 7.4) with BSA (100 equiv).

Compound	$\lambda_{MAX, abs}$ [nm]	$\lambda_{MAX, em}$ [nm]	ε [$M^{-1}cm^{-1}$]	Φ_F
**Acri-Py**	488	630	nd	0.15
**Acri-*o*vi**<br>(Clicked)	494<br>(496)	591<br>(593)	55,860<br>(67,260)	0.007<br>(0.16)
**Acri-*m*vi**<br>(Clicked)	493<br>(497)	605<br>(588)	49,300<br>(54,100)	0.025<br>(0.19)
**Acri-*p*vi**<br>(Clicked)	494<br>(500)	592<br>(590)	51,800<br>(54,600)	0.028<br>(0.14)
**Acri-*o*et**<br>(Clicked)	495<br>(498)	603<br>(602)	56,400<br>(57,200)	0.01<br>(0.13)
**Acri-*m*et**<br>(Clicked)	497<br>(496)	605<br>(601)	61,600<br>(54,400)	0.035<br>(0.15)
**Acri-*p*et**<br>(Clicked)	498<br>(498)	604<br>(601)	47,500<br>(48,200)	0.053<br>(0.15)

X. BSA Titration

Around 250 mg BSA (Sigma Aldrich) were solubilized in NaCaCo 10 mM buffer (4 mL). This solution was then filtered filtered with a 0.45 µm Nylon syringe filter. The concentration of this solution was determined by measuring its absorbance at 279 nm (ε(279 nm) = 44 355 L.mol^{-1}.cm^{-1}). Before fluorimetric titrations, UV titrations were performed to find an isobestic point. The excitation wavelength for the fluorimetric titration was thus the wavelength of the isobestic point. If no isobestic point is found, the excitation wavelength was chosen to minimize the variation of absorbance upon BSA addition.

The variation of the fluorescence signal of a fluorophore solution of fixed concentration (2 µM) was monitored upon addition of increasing quantities of BSA. The titration curves were then obtained by plotting F/F_0 versus the concentration of BSA, where F is the integrated fluorescence intensity of the BSA-dye complex and F_0 the initial integrated fluorescence intensity of the free dye.

Figure S3: Fluorimetric titrations of Acri-series in BSA

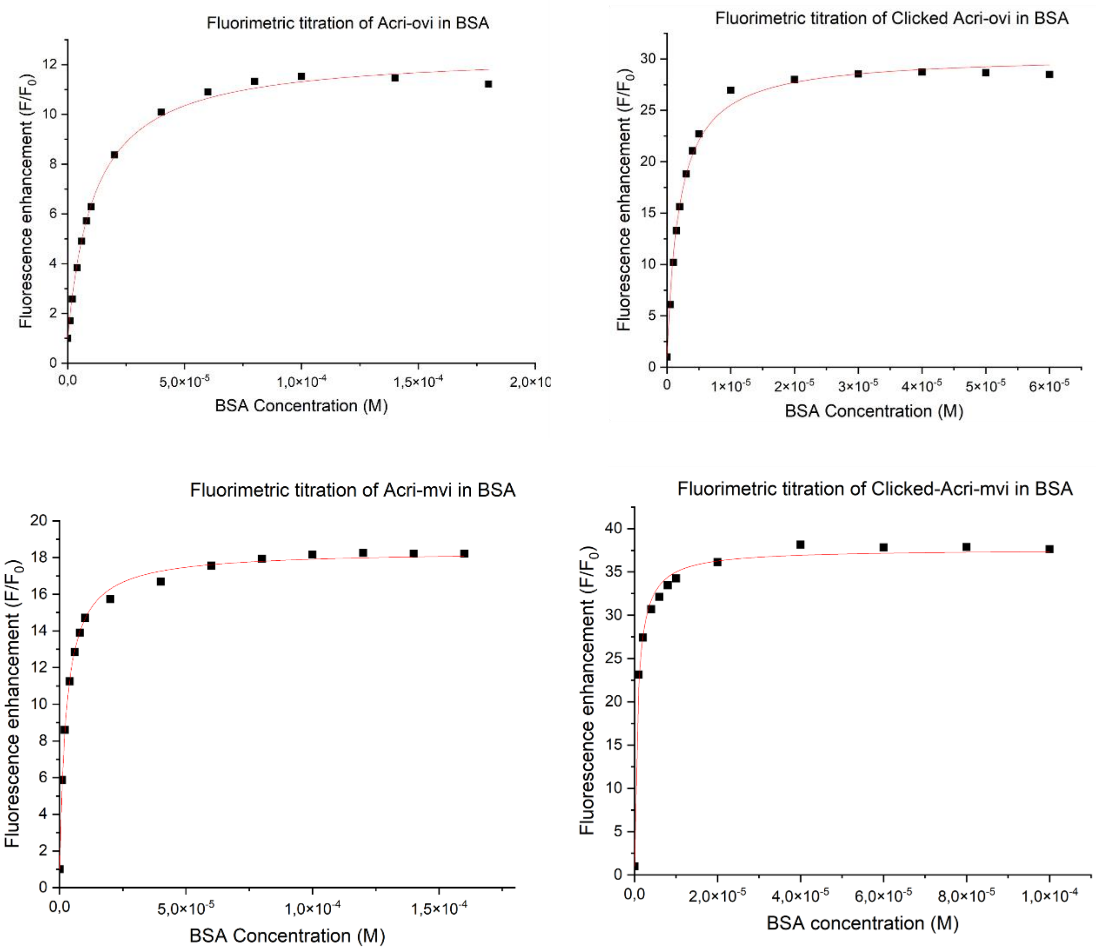

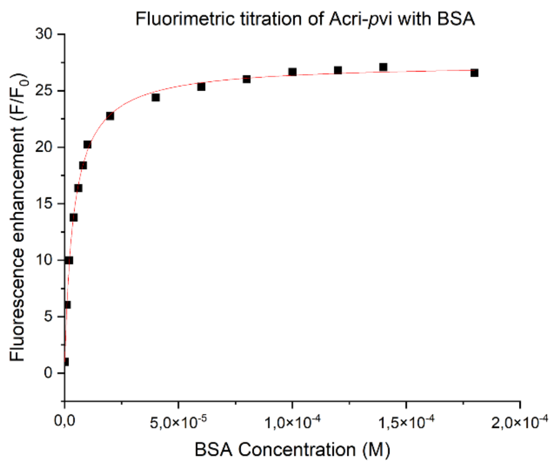
Fluorimetric titration of Acri-pvi with BSA
Fluorescence enhancement (F/F0)
BSA Concentration (M)
0
5
10
15
20
25
30
0,0
5,0×10-5
1,0×10-4
1,5×10-4
2,0×10-4

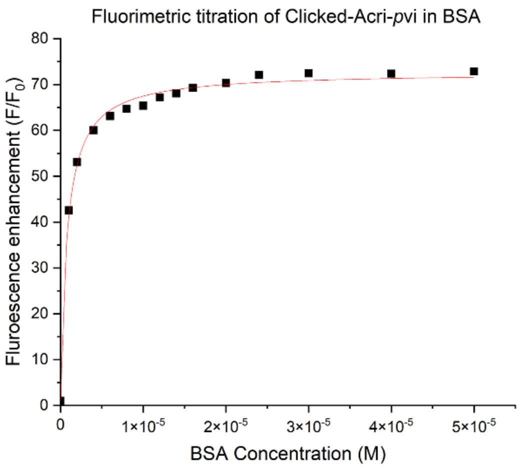
Fluorimetric titration of Clicked-Acri-pvi in BSA
Fluroescence enhancement (F/F0)
BSA Concentration (M)
0
10
20
30
40
50
60
70
80
0
1×10-5
2×10-5
3×10-5
4×10-5
5×10-5

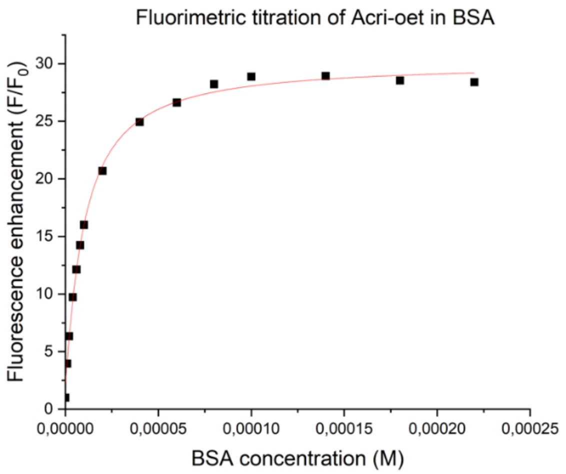
Fluorimetric titration of Acri-oet in BSA
Fluorescence enhancement (F/F0)
BSA concentration (M)
0
5
10
15
20
25
30
0,00000
0,00005
0,00010
0,00015
0,00020
0,00025

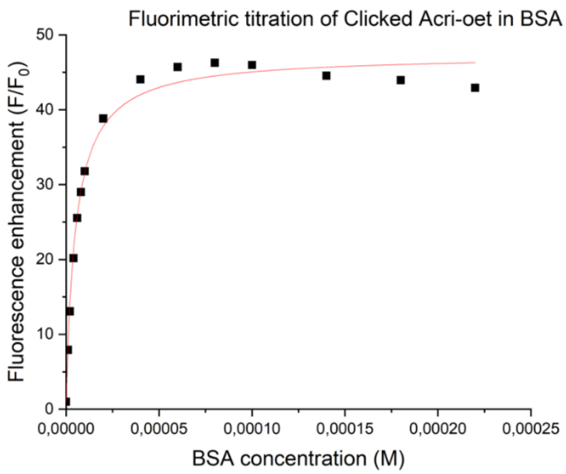
Fluorimetric titration of Clicked Acri-oet in BSA
Fluorescence enhancement (F/F0)
BSA concentration (M)
0
10
20
30
40
50
0,00000
0,00005
0,00010
0,00015
0,00020
0,00025

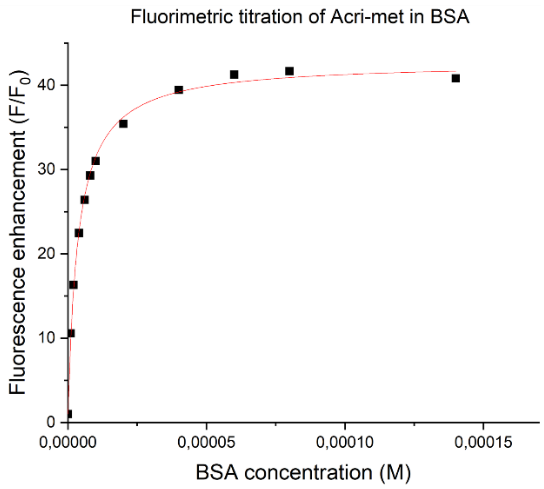
Fluorimetric titration of Acri-met in BSA
Fluorescence enhancement (F/F0)
BSA concentration (M)
0
10
20
30
40
0,00000
0,00005
0,00010
0,00015

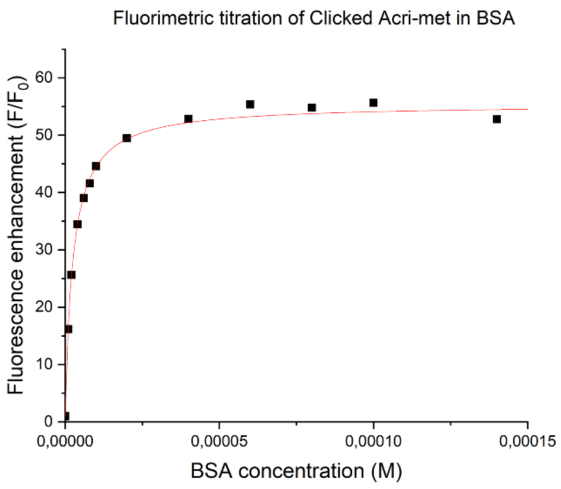
Fluorimetric titration of Clicked Acri-met in BSA
Fluorescence enhancement (F/F0)
BSA concentration (M)
0
10
20
30
40
50
60
0,00000
0,00005
0,00010
0,00015

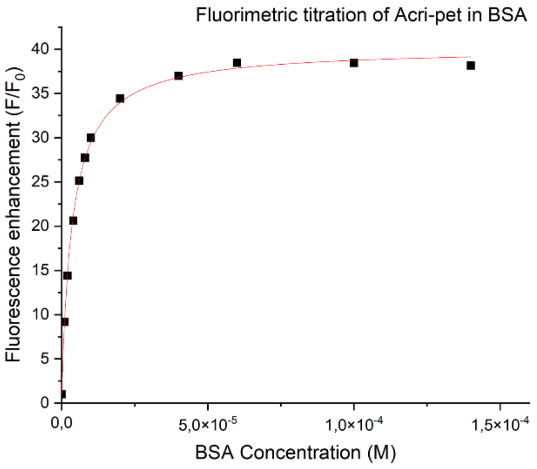

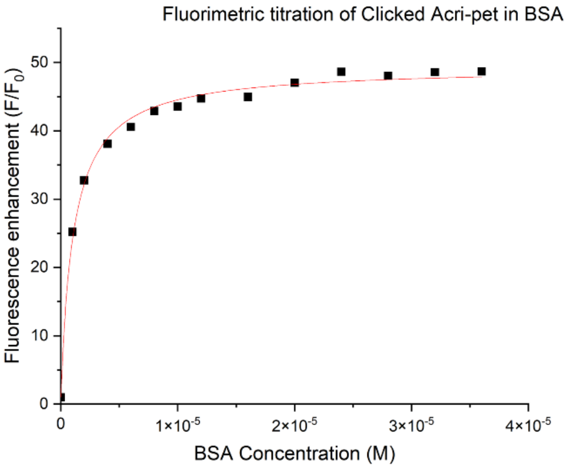

Table S4: Affinity constants of Acri-compounds in BSA

Compound	K_A (M^{-1})
Acri-*ovi*	$0.87x10^5$
Acri-*ovi* (Clicked)	$5.1x10^5$
Acri-*mvi*	$3.7x10^5$
Acri-*mvi* (Clicked)	$1.3x10^6$
Acri-*pvi*	$2.4x10^5$
Acri-*pvi* (Clicked)	$1.3x10^6$
Acri-*oet*	$1.0x10^5$
Acri-*oet* (Clicked)	$1.9x10^5$
Acri-*met*	$2.7x10^5$
Acri-*met* (Clicked)	$4.0x10^5$
Acri-*pet*	$2.7x10^5$
Acri-*pet* (Clicked)	$9.1x10^6$

XII. Kinetics

Measurements were performed in NaCaCo 10 mM in presence of 100 equivalents of BSA and at 37 °C. Reaction rates between fluorogenic probes and BCN were determined thanks to the method of apparent rate constants. The reaction rate of this reaction is: v = k[BCN][dye]. With a large excess of BCN, one can admit that $[BCN]=[BCN]_0$, so $v = k_{app}[dye]$ ($k_{app}=k[BCN]_0$). As a pseudo first order:

$$[dye]=[dye]_0 e^{-kappt}$$

$$\ln([dye]) = \ln([dye]_0) - k_{app}t$$

$$[dye]=[dye]_0(1-x), \text{ where x is the conversion}$$

$$-\ln(1-x) = k_{app}t + cst = k[BCN]_0 + cst$$

Correspondences between fluorescence intensity and the conversion were established calibrating the fluorescence intensity at 0 for t = 0 sec, and normalizing fluorescence intensity. Pseudo-first order reaction rates were then obtained by plotting -ln(1-x).

For each probe, the kinetic was performed with three different concentrations of BCN in order to perform a linear regression.

Figure S4: Rate constant determination for all fluorogenic probes in aqueous buffer with BSA (100 equiv)

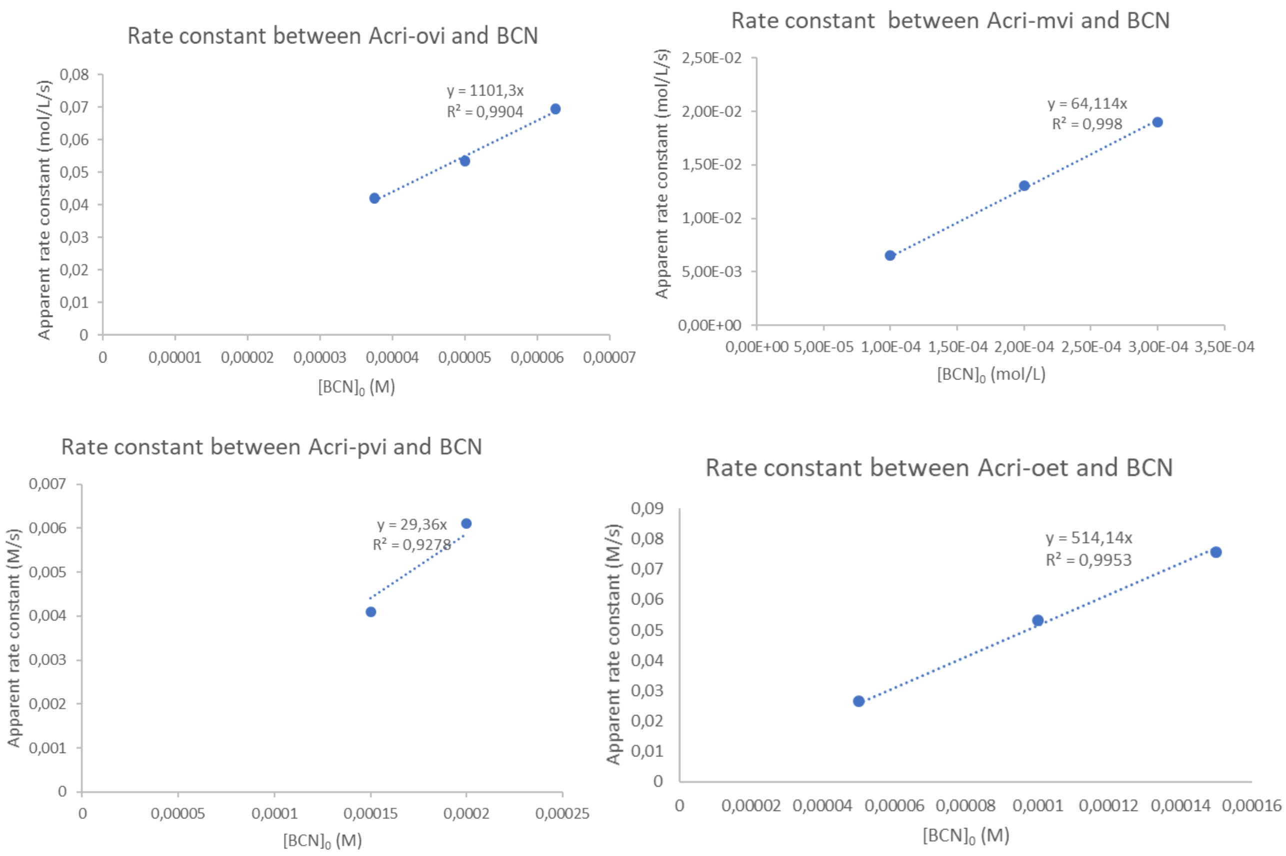

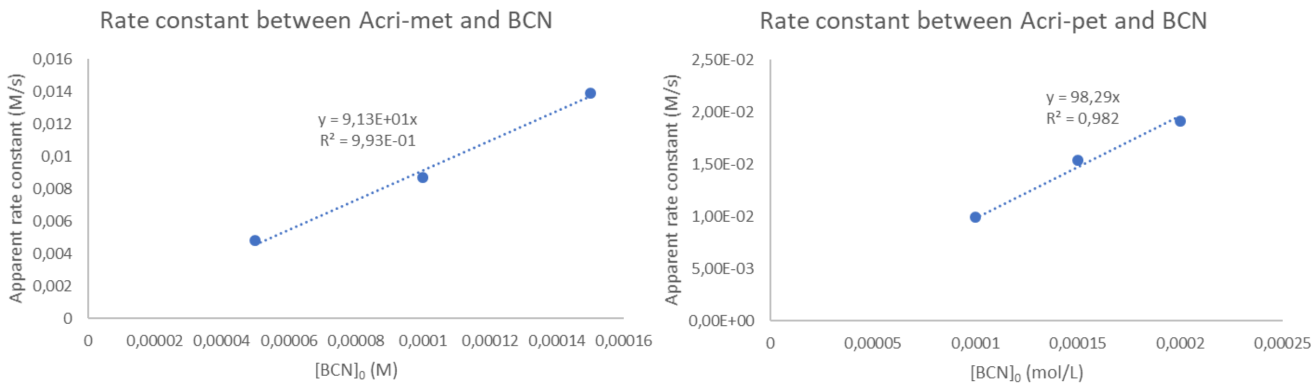

Figure S5: Turn-on determination between Acri-*ovi* and BCN in cell lysate

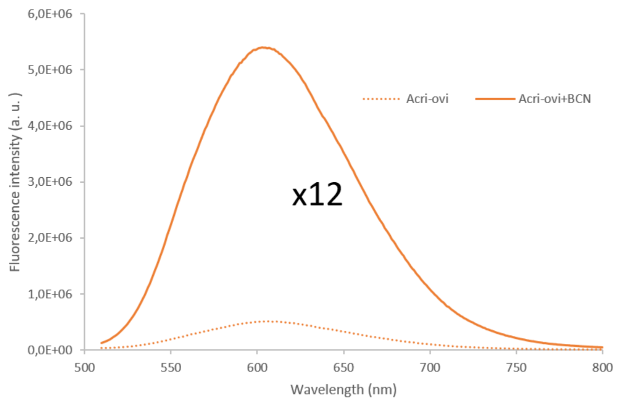

Figure S6: Rate constant determination for Acri-*ovi* with other dienophiles in aqueous buffer with BSA (100 equiv)

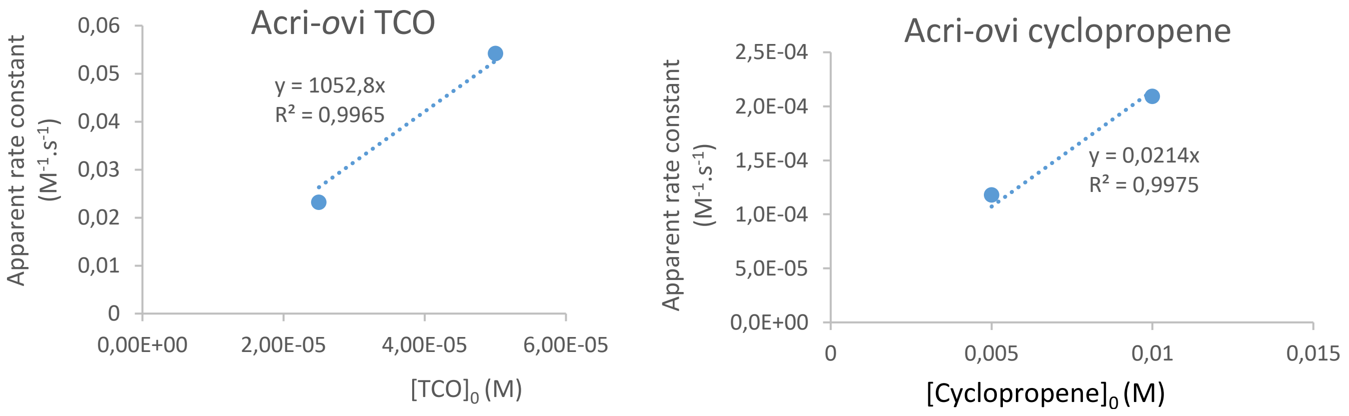

XIII. Two-photon excitation spectra

The two-photon excitation spectra were measured by the fluorescence method by using a Ti:sapphire femtosecond laser Insight DS (680–1300 nm) with pulse width < 120 fs and a repetition rate of 80 MHz (Spectra-Physics) The excitation beam was collimated over the cell length (Helma fluorescence semi-micro 10 × 4 mm) and the fluorescence, collected at 90◦ of the excitation beam, was focused into an optical fibre connected to a spectrometer (AvaSpec ULS from Avantes). The incident beam intensity was adjusted to ensure an intensity-squared dependence of the fluorescence over the whole spectral range reported. To avoid reabsorption of emitted light, micro cells are used and the focal point is very close to

the edge of the cuvette. This allows elimination of second order filter effects. The concentrations were 5.10^{-5} M for all probes. Calibration of the spectra was performed by comparison with the published Rhodamine B in MeOH in the 750–880 nm range and Fluorescein in 0.1 N NaOH solution in the 880–930 nm range and Styryl 9M in chloroform in the 930-1 000 nm range.[5,6]

Figure S7: Two-photon excitation spectra of Acri-*m*vi, Acri-*p*vi, Acri-*o*et, Acri-*m*et and Acri-*p*et and their Clicked adducts in glycerol

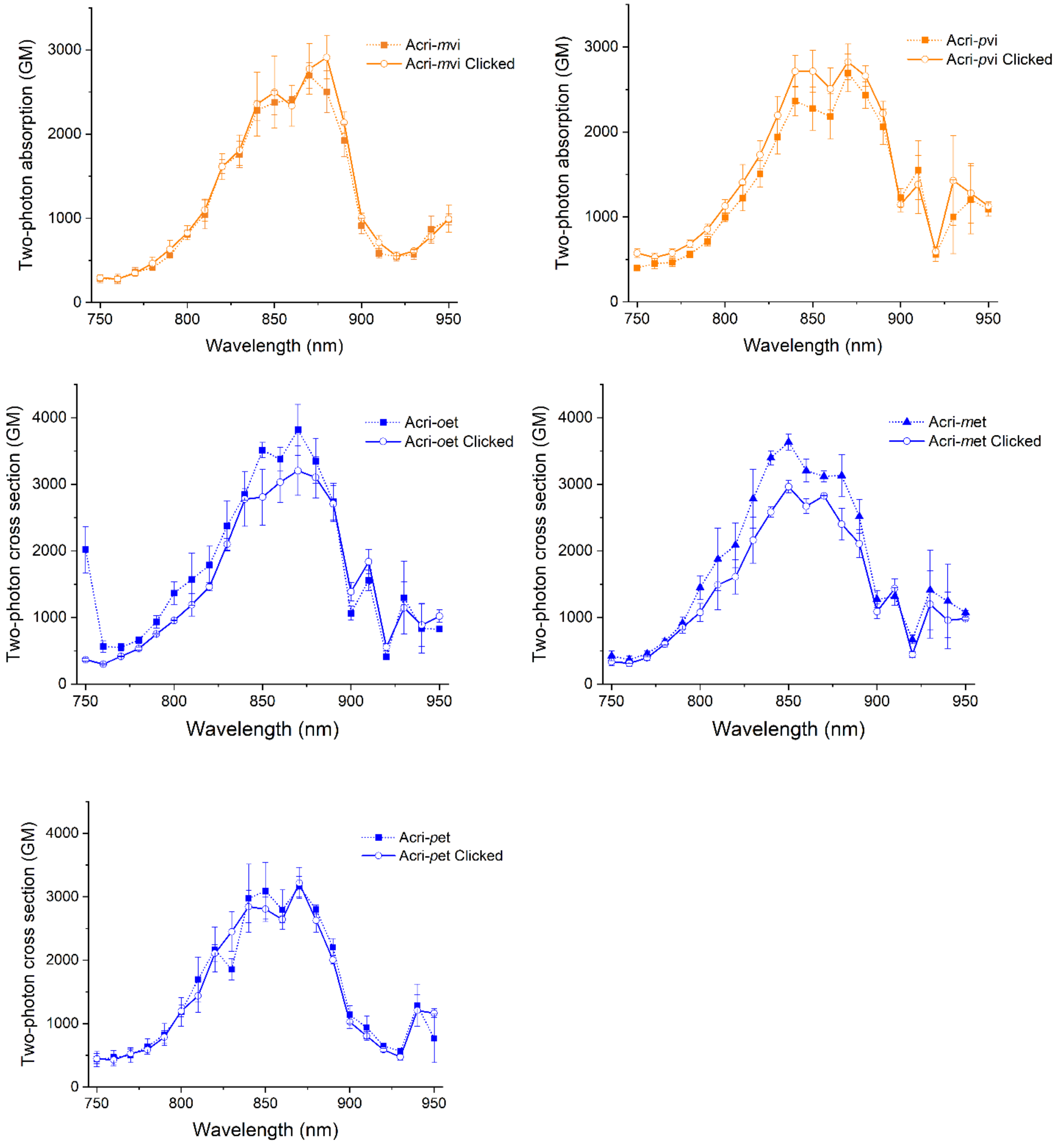

[5] C. Xu, W. W. Webb, *J. Opt. Soc. Am. B* 1996, **13**, 481– 491.

[6] N. S. Makarov, M. Drobizhev, A. Rebane, *Opt. Express* 2008, **16**, 4029– 4047.

XIV. Molecular modeling

All geometries were optimized at the B3LYP/6-31+g(d) level of calculation followed by a frequency calculation. In the case of minima all frequencies were real (no imaginary). The transition states were found with the QST2 procedure from Gaussian at the same level of theory followed by a frequency calculation to verify that one imaginary frequency is obtained. The reaction path was finally ascertained with an IRC calculation. A single point energy calculation with frequency was then done on the optimized geometries (minima and TS) at the APFD/6-311+g(d,p) level of calculation to obtain the thermal free energies at 25°C that were used to plot the reaction profiles. The reaction study was done on slightly simplified **Acri-Xvi** structures were the methyl groups of the pyridinium have been replaced by hydrogen (labelled **Acri-Xvi-H**) since the former tended to make transition states search difficult.

TDDFT on the **Acri-Xvi** and **clicked Acri-Xvi** geometries were done at the M06-2X/6-311+g(d,p) level of theory including acetonitrile solvent effect with IEFPCM model. The excitonic coupling energies were calculated with the transition charge from electrostatic potential (TrESP) method[7] following the procedure described by Tian Lu in the Multiwfn software[8]. All calculations were done with Gaussian 16 (Revision B.01) software.[9] Data were analyzed with GaussView 6.0 (Molecular orbitals), PyMOL (structure visualization, The PyMOL Molecular Graphics System, Version 2.1.1 Schrödinger, LLC) and GaussSum3.0 (vertical transitions and orbital energies[10]).

[7] *J. Phys. Chem. B* **2006**, *110*, 17268-17281

[8] Tian Lu, Feiwu Chen, Multiwfn: A Multifunctional Wavefunction Analyzer, *J. Comput. Chem.* **2012**, *33*, 580-592

[9] Gaussian 16, Revision B.01, M. J. Frisch, G. W. Trucks, H. B. Schlegel, G. E. Scuseria, M. A. Robb, J. R. Cheeseman, G. Scalmani, V. Barone, G. A. Petersson, H. Nakatsuji, X. Li, M. Caricato, A. V. Marenich, J. Bloino, B. G. Janesko, R. Gomperts, B. Mennucci, H. P. Hratchian, J. V. Ortiz, A. F. Izmaylov, J. L. Sonnenberg, D. Williams-Young, F. Ding, F. Lipparini, F. Egidi, J. Goings, B. Peng, A. Petrone, T. Henderson, D. Ranasinghe, V. G. Zakrzewski, J. Gao, N. Rega, G. Zheng, W. Liang, M. Hada, M. Ehara, K. Toyota, R. Fukuda, J. Hasegawa, M. Ishida, T. Nakajima, Y. Honda, O. Kitao, H. Nakai, T. Vreven, K. Throssell, J. A. Montgomery, Jr., J. E. Peralta, F. Ogliaro, M. J. Bearpark, J. J. Heyd, E. N. Brothers, K. N. Kudin, V. N. Staroverov, T. A. Keith, R. Kobayashi, J. Normand, K. Raghavachari, A. P. Rendell, J. C. Burant, S. S. Iyengar, J. Tomasi, M. Cossi, J. M. Millam, M. Klene, C. Adamo, R. Cammi, J. W. Ochterski, R. L. Martin, K. Morokuma, O. Farkas, J. B. Foresman, and D. J. Fox, Gaussian, Inc., Wallingford CT, 2016

[10] N. M. O'Boyle, A. L. Tenderholt and K. M. Langner. *J. Comp. Chem.* **2008**, *29*, 839-845.

Figure S8: Schematic representations (energy vs. reaction coordinate) of the reaction between **Acri-Xvi** (X= o, m or p) and BCN.

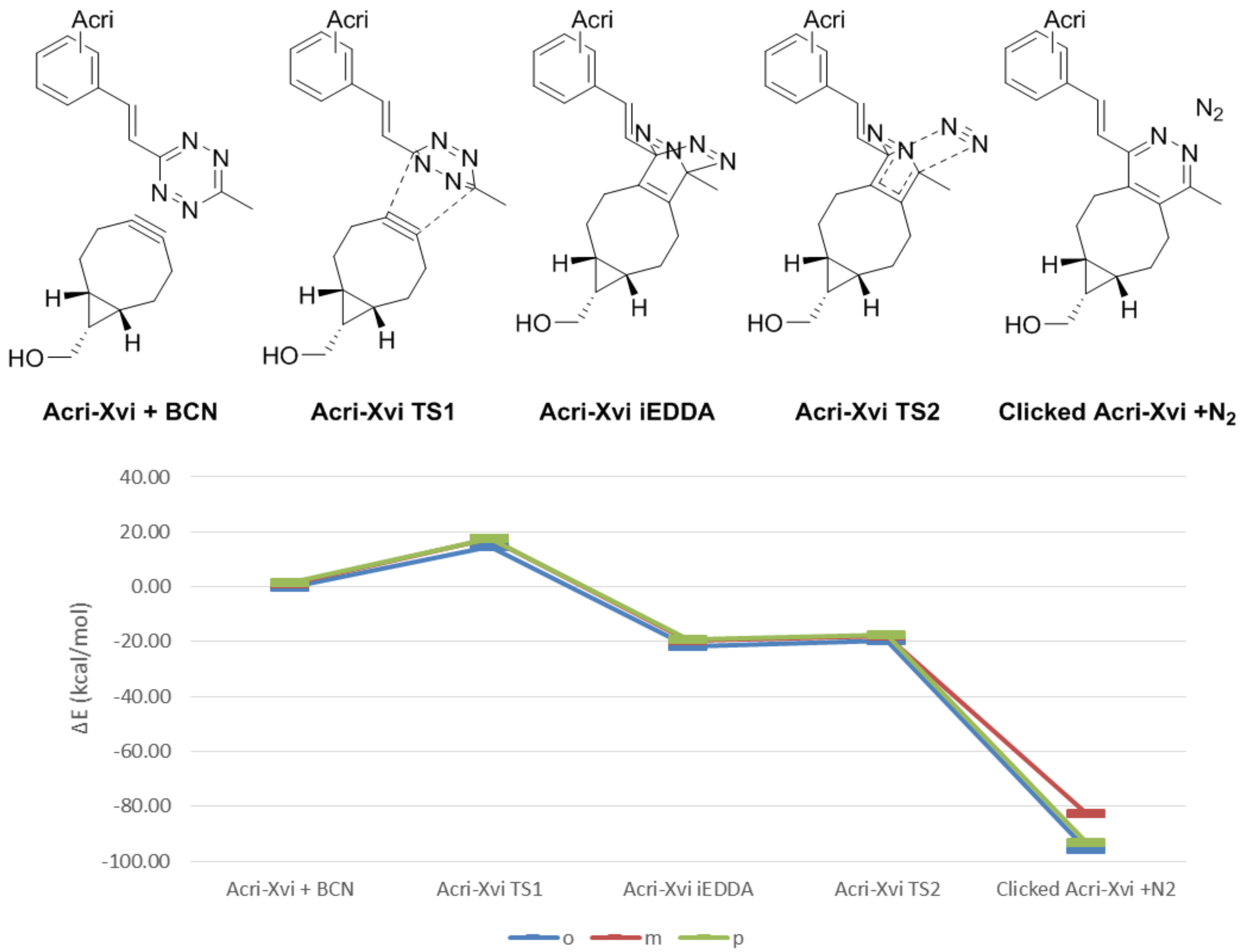

Table S5: Relative energies (kcal/mol) of the various intermediates of the reaction between **Acri-Xvi** (X= *o*, *m* or *p*) and BCN (**Acri-ovi** + BCN is the reference state)

	**Acri-Xvi + BCN**	**Acri-Xvi TS1**	**Acri-Xvi iEDDA**	**Acri-Xvi TS2**	**Clicked Acri-Xvi +N$_2$**
o	0.00	14.53	-21.62	-19.80	-95.90
m	1.19	17.54	-19.67	-17.98	-82.47
p	1.63	17.73	-19.23	-17.35	-93.32

Table S6: Calculated activation energies (kcal/mol) of TS1 and TS2 and relative rate constants for the first step (iEDDA reaction)

	**AE (TS1)**	**AE (TS2)**	**k_{rel} (TS1)**	**E_{LUMO} (eV)**
**Acri-ovi-H**	14.53	1.82	1.00	-3.30
**Acri-*m*vi-H**	16.35	1.69	0.05	-3.29
**Acri-*p*vi-H**	16.10	1.88	0.07	-3.29

Table S7: Comparison of calculated excitation and experimental absorption data and calculated excitonic coupling energy.

	Calculated (nm)	f	Experimental (nm)	Excitonic coupling energy (mEV)
**Acri-*o*vi**	565 / 465 (482)	0.0035 / 2.14 (2.22)	494 (496)	2.90
**Acri-*m*vi**	565 / 468 (483)	0.0040 / 2.16 (2.23)	493 (497)	1.35
**Acri-*p*vi**	565 / 458 (483)	0.0043 / 2.11 (2.24)	494 (500)	0.93

Table S8: Calculated transitions

Acri-*o*vi

No.	Wavelength (nm)	Osc. Strength	Symmetry	Major contribs
**1**	565	0.0035	Singlet-A	H-3->L+2 (87%), H-3->L+3 (12%)
**2**	465	2.1361	Singlet-A	HOMO->LUMO (88%)

Acri-*m*vi

No.	Wavelength (nm)	Osc. Strength	Symmetry	Major contribs
**1**	565	0.004	Singlet-A	H-3->L+2 (91%)
**2**	468	2.1575	Singlet-A	HOMO->LUMO (88%)

Acri-*p*vi

No.	Wavelength (nm)	Osc. Strength	Symmetry	Major contribs
**1**	565	0.0043	Singlet-A	H-3->L+2 (91%)
**2**	458	2.1122	Singlet-A	HOMO->LUMO (87%)

Clicked Acri-*o*vi

No.	Wavelength (nm)	Osc. Strength	Symmetry	Major contribs
**1**	482	2.2223	Singlet-A	HOMO->LUMO (88%)

Clicked Acri-*m*vi

No.	Wavelength (nm)	Osc. Strength	Symmetry	Major contribs
**1**	483	2.2292	Singlet-A	HOMO->LUMO (88%)

Clicked Acri-*p*vi

No.	Wavelength (nm)	Osc. Strength	Symmetry	Major contribs
**1**	483	2.243	Singlet-A	HOMO->LUMO (88%)

Figure S9. Molecular structures and energy levels of key states during the excitation and de-excitation processes of Acri-vi and Acri-vi-Clicked

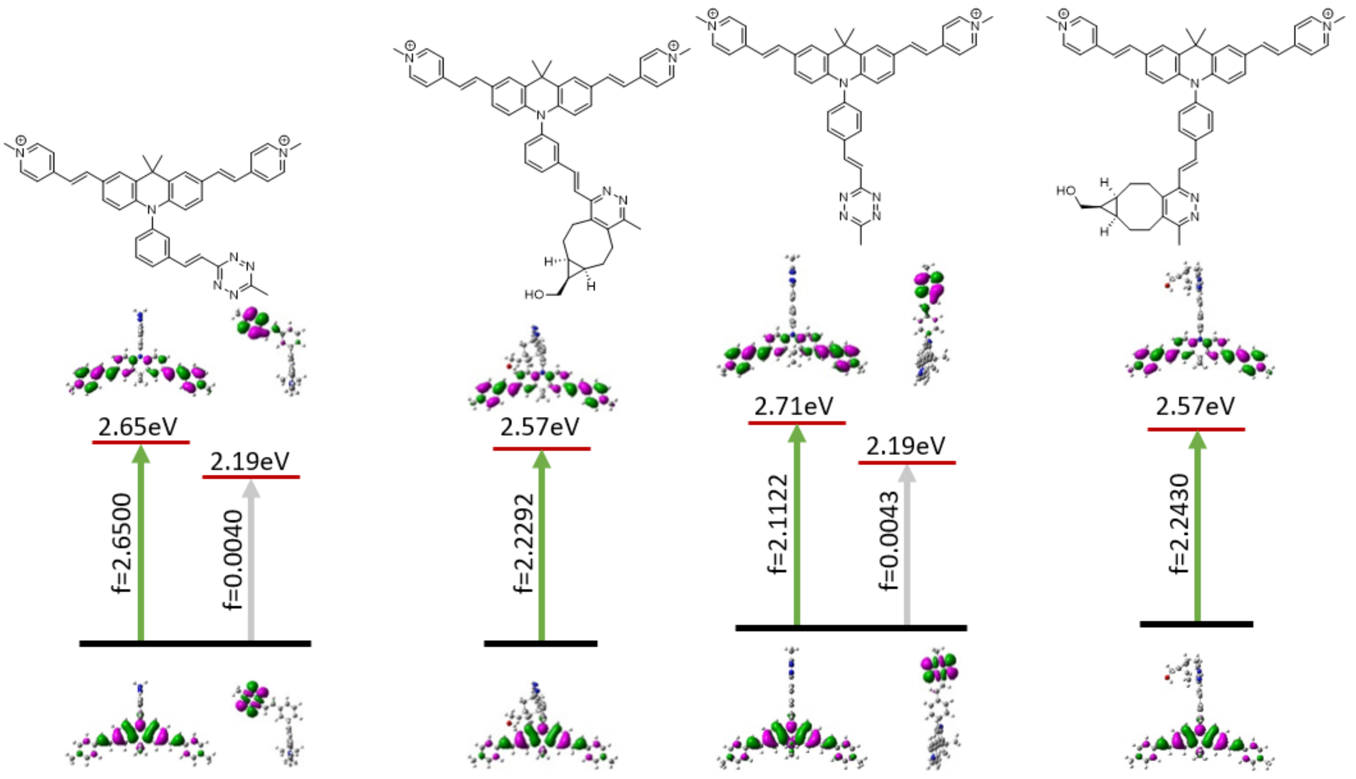

XV. Cartesian coordinates of calculated structures

BCN

Tag	Symbol	X	Y	Z
1	C	0.648069	-1.691942	-0.323378
2	C	2.204257	-1.631927	-0.239693
3	C	-0.043748	-0.942125	0.812705
4	C	2.552208	-0.205504	-0.254952
5	C	-0.355959	0.551053	0.882693
6	C	2.304224	0.983613	-0.205054
7	C	-0.034527	1.607197	-0.17127
8	C	1.42455	2.151706	-0.076835
9	C	-1.487398	-0.463911	0.753527
10	C	-2.328985	-0.591754	-0.496161
11	O	-3.464264	0.295846	-0.481315
12	H	0.337666	-2.746178	-0.292198
13	H	0.350663	-1.303145	-1.302749
14	H	2.6442	-2.184542	-1.07868
15	H	2.558164	-2.11645	0.680113
16	H	0.231092	-1.359423	1.78269
17	H	-0.25323	0.954887	1.891045
18	H	-0.723553	2.455768	-0.048478
19	H	-0.182638	1.220422	-1.185332

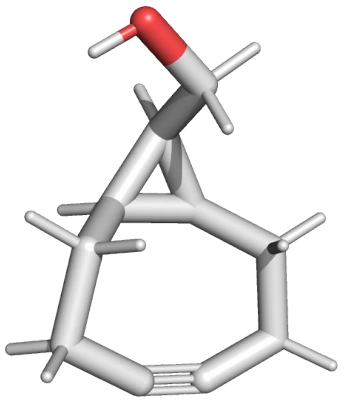

20	H	1.580463	2.661239	0.883365
21	H	1.59416	2.897946	-0.862437
22	H	-2.075916	-0.631118	1.655754
23	H	-1.744034	-0.42548	-1.408397
24	H	-2.762534	-1.59494	-0.560721
25	H	-3.136531	1.205815	-0.392948

Dinitrogen

Tag	Symbol	X	Y	Z
1	N	0	0	0.552449
2	N	0	0	-0.552449

Acri-ovi

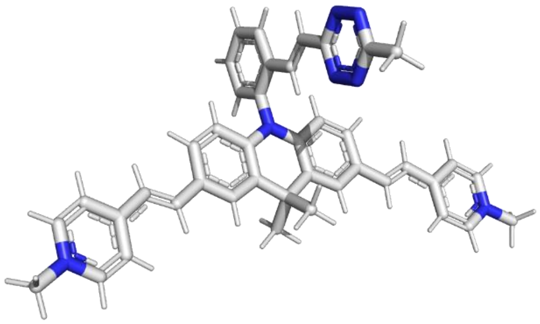

Tag	Symbol	X	Y	Z
1	C	1.264412	-1.180282	0.378222
2	C	0.000043	-1.852034	-0.171493
3	C	-1.264333	-1.180306	0.378237
4	C	1.222474	-0.089443	1.275384
5	N	0.000032	0.44065	1.706923
6	C	-1.222405	-0.08946	1.275392
7	C	-2.517683	-1.653196	-0.010537
8	C	-3.735151	-1.103048	0.437382
9	C	-2.433957	0.47899	1.737733
10	C	-3.659295	-0.013212	1.33052
11	H	-2.568642	-2.492761	-0.697821
12	H	-2.407994	1.315562	2.424897
13	H	-4.559593	0.456567	1.713657
14	C	2.517767	-1.653158	-0.010553
15	C	3.735229	-1.102996	0.437364
16	C	3.659363	-0.013168	1.330509
17	C	2.43402	0.479017	1.737729
18	H	2.568735	-2.492724	-0.697836
19	H	4.559657	0.456619	1.713649
20	H	2.408048	1.31558	2.424904
21	C	4.979336	-1.678674	-0.038035
22	C	6.241199	-1.2717	0.270393
23	H	4.852782	-2.522983	-0.713597
24	C	7.461212	-1.872001	-0.224399
25	H	6.3855	-0.426859	0.939019
26	C	-4.979252	-1.678746	-0.038007
27	C	-6.241119	-1.271789	0.27043
28	H	-4.852691	-2.523057	-0.713564
29	C	-7.461127	-1.872113	-0.224346
30	H	-6.385427	-0.426947	0.939054
31	C	7.517369	-2.990381	-1.092577

32	C	8.727593	-3.495884	-1.509235
33	N	9.902876	-2.946348	-1.10614
34	C	8.70941	-1.329302	0.170293
35	H	6.619343	-3.480142	-1.448729
36	H	8.799517	-4.350127	-2.170732
37	C	11.192321	-3.481983	-1.590142
38	H	11.023542	-4.460762	-2.036656
39	H	11.87878	-3.578262	-0.747846
40	H	11.609994	-2.802027	-2.336279
41	C	-7.517276	-2.990491	-1.092527
42	C	-8.727496	-3.496018	-1.509167
43	N	-9.902784	-2.946506	-1.106052
44	C	-9.892152	-1.872116	-0.275475
45	H	-8.799413	-4.35026	-2.170666
46	H	-6.619245	-3.480232	-1.448695
47	C	-11.192225	-3.482164	-1.590038
48	H	-11.023433	-4.460938	-2.036557
49	H	-11.609921	-2.802214	-2.336168
50	H	-11.878672	-3.578457	-0.747733
51	C	0.000028	1.500486	2.688537
52	C	0.000007	2.85648	2.284862
53	C	0.000037	1.147713	4.039609
54	C	0.000004	3.835565	3.300964
55	C	0.000022	3.484588	4.647641
56	C	0.000027	2.136062	5.024081
57	H	0.000052	0.09562	4.309528
58	H	0.000034	1.857261	6.073738
59	C	9.892236	-1.871955	-0.275567
60	H	8.75098	-0.473325	0.834955
61	H	10.859296	-1.475854	0.009567
62	C	-8.70933	-1.329441	0.170369
63	H	-8.750907	-0.473468	0.835035
64	H	-10.859215	-1.476035	0.009676
65	H	0.000019	4.262598	5.405652
66	H	0.000005	4.887286	3.033461
67	C	0.000006	3.194398	0.860091
68	C	-0.000139	4.436779	0.329595
69	H	0.000134	2.354714	0.171701
70	C	-0.00012	4.702003	-1.103831
71	H	-0.000286	5.332087	0.943865
72	N	0.000093	3.671069	-1.983315
73	N	0.000069	3.948839	-3.263837
74	N	-0.000203	5.998161	-1.482559
75	N	-0.000226	6.271043	-2.773385
76	C	-0.000178	5.246843	-3.643138
77	C	-0.000588	5.546147	-5.10874
78	H	-0.886293	5.111813	-5.585723

79	H	0.88088	5.105442	-5.587619
80	H	0.002931	6.625358	-5.273395
81	C	0.000063	-3.349765	0.241801
82	H	0.000066	-3.451923	1.332447
83	H	0.883202	-3.864318	-0.150492
84	H	-0.883061	-3.864344	-0.150491
85	C	0.000027	-1.742752	-1.72137
86	H	0.000026	-0.693855	-2.036921
87	H	-0.883172	-2.228969	-2.148243
88	H	0.883211	-2.228973	-2.148263

Acri-ovi-H

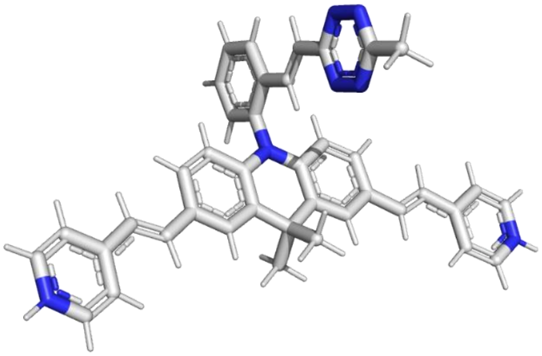

Tag	Symbol	X	Y	Z
1	C	1.263916	-1.376566	0.189642
2	C	-0.000262	-2.015681	-0.397496
3	C	-1.264397	-1.376397	0.189549
4	C	1.222164	-0.343188	1.152632
5	N	-0.00019	0.156088	1.619174
6	C	-1.222576	-0.343015	1.152532
7	C	-2.517289	-1.823422	-0.228527
8	C	-3.734912	-1.300732	0.251587
9	C	-2.434219	0.198364	1.646855
10	C	-3.659479	-0.266351	1.208815
11	H	-2.568534	-2.620176	-0.964992
12	H	-2.40799	0.991476	2.383729
13	H	-4.55987	0.180511	1.618191
14	C	2.516778	-1.823731	-0.228373
15	C	3.734436	-1.301185	0.251807
16	C	3.659072	-0.266813	1.209051
17	C	2.433843	0.198039	1.647032
18	H	2.56797	-2.620474	-0.964854
19	H	4.559494	0.179943	1.618476
20	H	2.407667	0.991151	2.383908
21	C	4.976118	-1.85017	-0.256708
22	C	6.240733	-1.46648	0.073573
23	H	4.845599	-2.652366	-0.980796
24	C	7.455828	-2.043623	-0.456307
25	H	6.389123	-0.66021	0.787219
26	C	-4.976631	-1.849601	-0.256963
27	C	-6.241219	-1.465782	0.073272
28	H	-4.846169	-2.651827	-0.981028
29	C	-7.456353	-2.042823	-0.45663
30	H	-6.389553	-0.659485	0.786899
31	C	7.495615	-3.127229	-1.374067
32	C	8.698218	-3.613181	-1.828573
33	N	9.862473	-3.062025	-1.403019

34	C	8.706326	-1.511881	-0.045663
35	H	6.589912	-3.597733	-1.735698
36	H	8.781599	-4.435984	-2.527379
37	C	-7.496214	-3.126443	-1.37437
38	C	-8.698849	-3.612296	-1.828897
39	N	-9.863065	-3.061027	-1.403383
40	C	-9.887378	-2.028424	-0.526796
41	H	-8.782286	-4.435108	-2.527685
42	H	-6.590543	-3.597035	-1.735969
43	C	-0.000169	1.135081	2.682042
44	C	0.000028	2.518858	2.388575
45	C	-0.000351	0.674449	4.000375
46	C	0.000027	3.412655	3.480507
47	C	-0.00016	2.954639	4.794515
48	C	-0.00035	1.580169	5.061228
49	H	-0.000502	-0.395986	4.184849
50	H	-0.000492	1.217567	6.084946
51	C	9.886856	-2.029442	-0.526412
52	H	8.746067	-0.685051	0.654707
53	H	10.862909	-1.655606	-0.244014
54	C	-8.706813	-1.510961	-0.046028
55	H	-8.746497	-0.684115	0.654325
56	H	-10.863404	-1.654497	-0.24443
57	H	-0.000155	3.669241	5.612579
58	H	0.000186	4.482308	3.29815
59	C	0.000265	2.971616	0.995922
60	C	0.00049	4.253804	0.57058
61	H	0.000295	2.191053	0.240981
62	C	0.000787	4.639853	-0.835004
63	H	0.000495	5.094606	1.257683
64	N	0.000892	3.688631	-1.799914
65	N	0.001192	4.07595	-3.05184
66	N	0.001115	5.963953	-1.100646
67	N	0.001419	6.347241	-2.362941
68	C	0.001379	5.401862	-3.317852
69	C	0.001379	5.82671	-4.752069
70	H	-0.884123	5.435212	-5.265113
71	H	0.883032	5.429155	-5.266992
72	H	0.00486	6.916126	-4.822656
73	C	-0.000377	-3.534156	-0.068749
74	H	-0.000423	-3.696797	1.01455
75	H	0.882904	-4.025928	-0.488894
76	H	-0.883697	-4.025807	-0.488952
77	C	-0.000191	-1.817522	-1.938525
78	H	-0.000116	-0.752138	-2.192953
79	H	-0.883224	-2.278085	-2.3931
80	H	0.882822	-2.278192	-2.393031

81	H	10.740794	-3.436269	-1.7504
82	H	-10.741411	-3.4352	-1.750778

Acri-ovi-H-TS1

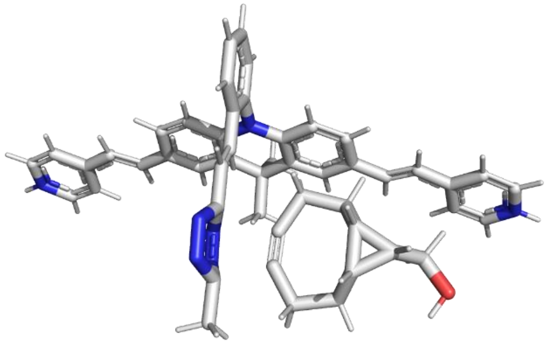

Tag	Symbol	X	Y	Z
1	C	-2.826482	1.728458	-0.032202
2	C	-1.70543	2.515451	-0.721863
3	C	-0.398319	2.425067	0.074941
4	C	-2.633732	1.023284	1.177394
5	N	-1.385028	1.00858	1.809996
6	C	-0.287458	1.697972	1.281992
7	C	0.739414	3.079801	-0.395467
8	C	1.985151	3.055352	0.263658
9	C	0.954377	1.662969	1.962304
10	C	2.062473	2.323486	1.468042
11	H	0.67243	3.642284	-1.322315
12	H	1.043867	1.110203	2.889114
13	H	2.991527	2.267058	2.026331
14	C	-4.095744	1.694125	-0.608299
15	C	-5.188624	0.998629	-0.052708
16	C	-4.963417	0.302674	1.154403
17	C	-3.717758	0.315678	1.751808
18	H	-4.26177	2.230508	-1.53823
19	H	-5.762196	-0.255015	1.632921
20	H	-3.574467	-0.228283	2.677116
21	C	-6.461996	1.035904	-0.744175
22	C	-7.624763	0.439169	-0.359687
23	H	-6.452033	1.613589	-1.666559
24	C	-8.875425	0.501047	-1.082159
25	H	-7.654478	-0.137551	0.561058
26	C	3.100535	3.769478	-0.325477
27	C	4.371547	3.850763	0.158435
28	H	2.861799	4.282206	-1.255538
29	C	5.460051	4.570398	-0.463815
30	H	4.62773	3.342837	1.084633
31	C	-9.058051	1.187414	-2.312577
32	C	-10.284588	1.195184	-2.932482
33	N	-11.336202	0.544934	-2.374193
34	C	-10.012639	-0.160322	-0.548491
35	H	-8.244639	1.716734	-2.792839
36	H	-10.474044	1.702888	-3.869787
37	C	5.347838	5.305556	-1.674245
38	C	6.438049	5.957666	-2.198731
39	N	7.635652	5.905875	-1.563575
40	C	7.802186	5.223147	-0.405236
41	H	6.404208	6.53026	-3.11692

42	H	4.410465	5.371754	-2.212126
43	C	-1.238826	0.308081	3.065994
44	C	-0.838174	-1.048034	3.087305
45	C	-1.498298	1.007982	4.246644
46	C	-0.714441	-1.66409	4.350443
47	C	-0.972656	-0.968293	5.528071
48	C	-1.366931	0.37441	5.48222
49	H	-1.802404	2.049123	4.186455
50	H	-1.570546	0.921186	6.398246
51	C	-11.222123	-0.126713	-1.203157
52	H	-9.941356	-0.703121	0.387563
53	H	-12.115512	-0.615133	-0.83505
54	C	6.737476	4.556389	0.155319
55	H	6.890527	4.015814	1.082793
56	H	8.793665	5.240331	0.029263
57	H	-0.868973	-1.47385	6.484024
58	H	-0.416797	-2.706121	4.411357
59	C	-0.575883	-1.7559	1.829786
60	C	-0.132989	-3.023355	1.700433
61	H	-0.765307	-1.193665	0.920167
62	C	0.092077	-3.681429	0.413918
63	H	0.092672	-3.646946	2.561251
64	N	-0.441155	-3.124017	-0.733616
65	N	-0.180214	-3.71889	-1.841337
66	N	0.225008	-5.058126	0.454222
67	N	0.474777	-5.653256	-0.656578
68	C	0.636157	-4.841096	-1.771847
69	C	0.861838	-5.559498	-3.074886
70	H	1.107252	-4.857973	-3.874606
71	H	-0.063685	-6.077472	-3.353061
72	H	1.651204	-6.307991	-2.98564
73	C	-2.128322	4.006032	-0.837481
74	H	-2.296463	4.440356	0.154051
75	H	-3.051766	4.107145	-1.416854
76	H	-1.355916	4.594729	-1.342904
77	C	-1.475064	1.930383	-2.142558
78	H	-1.17662	0.877999	-2.085798
79	H	-0.689962	2.481491	-2.670257
80	H	-2.386738	1.99596	-2.745036
81	C	4.267583	-1.973721	0.687572
82	C	2.805085	-2.419968	0.944726
83	C	5.23277	-3.143582	0.585461
84	C	2.329255	-3.271191	-0.160104
85	C	5.507071	-3.899178	-0.703436
86	C	2.490502	-3.899672	-1.225297
87	C	4.848098	-3.566041	-2.032173
88	C	3.497247	-4.288091	-2.253016

89	C	6.598765	-3.041703	-0.073352
90	C	7.116271	-1.764928	-0.697482
91	O	8.196018	-2.008294	-1.619053
92	H	4.563394	-1.328487	1.525953
93	H	4.287689	-1.347679	-0.209763
94	H	2.16979	-1.53403	1.054475
95	H	2.74194	-2.966405	1.895094
96	H	5.216948	-3.767679	1.479541
97	H	5.656461	-4.971476	-0.573884
98	H	5.509942	-3.876051	-2.853251
99	H	4.691026	-2.488735	-2.149135
100	H	3.652309	-5.374352	-2.223083
101	H	3.13313	-4.052807	-3.258462
102	H	7.391893	-3.598845	0.424752
103	H	6.324497	-1.196587	-1.199442
104	H	7.549217	-1.117661	0.071961
105	H	7.874536	-2.606847	-2.313164
106	H	8.429839	6.393065	-1.968673
107	H	-12.234391	0.563037	-2.848505

Acri-ovi-iEDDA

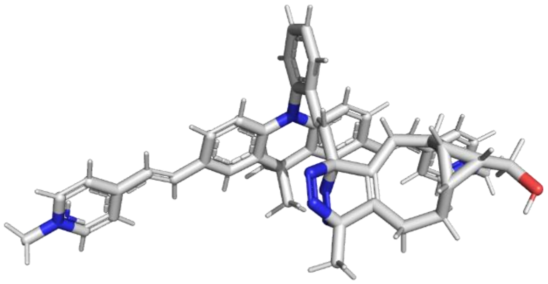

Tag	Symbol	X	Y	Z
1	C	2.376419	-2.112423	0.19532
2	C	1.327458	-2.998443	-0.487852
3	C	-0.033732	-2.876674	0.208088
4	C	2.086321	-1.319729	1.328792
5	N	0.800754	-1.297597	1.88201
6	C	-0.243416	-2.054476	1.338531
7	C	-1.120265	-3.604414	-0.276469
8	C	-2.408672	-3.560137	0.293097
9	C	-1.532054	-1.990526	1.922694
10	C	-2.588105	-2.722684	1.414778
11	H	-0.974511	-4.246433	-1.140591
12	H	-1.699404	-1.357652	2.785408
13	H	-3.556452	-2.639548	1.898113
14	C	3.679029	-2.083087	-0.302232
15	C	4.715121	-1.312403	0.262218
16	C	4.393393	-0.531004	1.392606
17	C	3.112505	-0.536238	1.910543
18	H	3.9192	-2.688479	-1.17165
19	H	5.14444	0.087102	1.874353
20	H	2.896016	0.072759	2.779344
21	C	6.034773	-1.369784	-0.33711
22	C	7.153757	-0.714337	0.077762
23	H	6.102871	-2.020053	-1.207471
24	C	8.45776	-0.804808	-0.541716

25	H	7.104959	-0.064489	0.947615
26	C	-3.460595	-4.367041	-0.295333
27	C	-4.756058	-4.4547	0.114516
28	H	-3.145146	-4.95484	-1.155577
29	C	-5.773986	-5.279425	-0.499028
30	H	-5.090685	-3.873834	0.970133
31	C	8.754907	-1.584496	-1.688775
32	C	10.029367	-1.618475	-2.202594
33	N	11.044253	-0.913339	-1.634153
34	C	9.540647	-0.08039	0.012896
35	H	7.995559	-2.173064	-2.188863
36	H	10.286356	-2.204163	-3.07689
37	C	12.391303	-0.95244	-2.240336
38	H	12.622258	-1.977857	-2.531309
39	H	13.120026	-0.609423	-1.50702
40	H	12.413409	-0.30146	-3.117587
41	C	-5.559122	-6.125992	-1.617107
42	C	-6.58501	-6.88149	-2.131952
43	N	-7.833834	-6.846333	-1.593033
44	C	-8.081851	-6.046651	-0.526064
45	H	-6.451414	-7.537244	-2.983855
46	H	-4.589146	-6.204081	-2.092653
47	C	-8.906392	-7.666196	-2.193696
48	H	-8.564895	-8.69932	-2.275075
49	H	-9.149752	-7.274344	-3.183712
50	H	-9.786152	-7.622344	-1.553531
51	C	0.564738	-0.533077	3.086268
52	C	0.184456	0.824571	3.0098
53	C	0.736245	-1.172367	4.317108
54	C	-0.028745	1.504224	4.226144
55	C	0.14179	0.870111	5.454585
56	C	0.527092	-0.474593	5.506332
57	H	1.035063	-2.216804	4.331245
58	H	0.658889	-0.974726	6.461415
59	C	10.799903	-0.14917	-0.540095
60	H	9.395189	0.540128	0.890542
61	H	11.64364	0.393674	-0.132563
62	C	-7.087868	-5.270254	0.027849
63	H	-7.334206	-4.649478	0.882506
64	H	-9.093516	-6.055823	-0.140301
65	H	-0.033826	1.423044	6.373206
66	H	-0.347394	2.541911	4.20869
67	C	0.014977	1.474137	1.698888
68	C	-0.045659	2.797596	1.484227
69	H	-0.050965	0.809767	0.843153
70	C	-0.21038	3.436961	0.138545
71	H	0.057889	3.505138	2.303447

72	N	0.095549	2.472635	-0.971506
73	N	0.140396	2.993276	-2.088608
74	N	0.856919	4.523345	0.001236
75	N	0.899979	5.033509	-1.114984
76	C	-0.12142	4.477296	-2.093685
77	C	0.176262	5.026357	-3.474789
78	H	-0.472764	4.57566	-4.229675
79	H	1.21301	4.786327	-3.730058
80	H	0.059336	6.112237	-3.506769
81	C	1.79724	-4.478323	-0.426687
82	H	1.905133	-4.808839	0.612147
83	H	2.762535	-4.603575	-0.927961
84	H	1.078314	-5.138136	-0.923326
85	C	1.181272	-2.562876	-1.971873
86	H	0.852564	-1.520289	-2.040335
87	H	0.449407	-3.18792	-2.49359
88	H	2.133576	-2.657421	-2.503663
89	C	-4.011181	4.490264	0.7801
90	C	-2.567862	3.989517	0.908816
91	C	-4.116475	5.997064	0.651695
92	C	-1.553732	4.119621	-0.214642
93	C	-4.059724	6.643662	-0.711439
94	C	-1.501461	4.687514	-1.438821
95	C	-3.90083	5.777992	-1.944828
96	C	-2.439419	5.497037	-2.319046
97	C	-5.347831	6.695941	0.099878
98	C	-6.616398	5.97288	-0.291215
99	O	-7.427769	6.749864	-1.193747
100	H	-4.528163	4.170686	1.695417
101	H	-4.512782	3.969878	-0.040062
102	H	-2.61361	2.924525	1.169842
103	H	-2.127305	4.473995	1.793682
104	H	-3.615086	6.528919	1.460689
105	H	-3.520731	7.58892	-0.777211
106	H	-4.334522	6.298224	-2.810484
107	H	-4.450366	4.836472	-1.859105
108	H	-1.952777	6.468189	-2.492111
109	H	-2.453173	5.010329	-3.30233
110	H	-5.55663	7.664806	0.553104
111	H	-6.416027	4.989092	-0.731301
112	H	-7.250153	5.817786	0.587893
113	H	-6.895007	6.952944	-1.98021

Acri-ovi-H-iEDDA

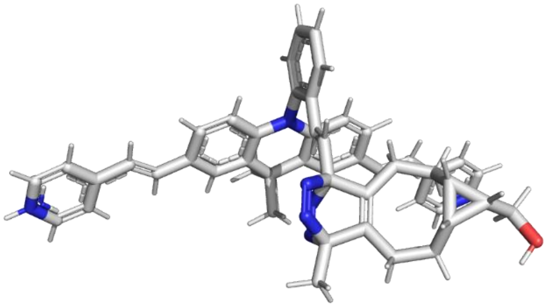

Tag	Symbol	X	Y	Z
1	C	-2.69978	1.986908	0.000948
2	C	-1.710777	2.910689	-0.720589
3	C	-0.363433	2.953761	0.010729
4	C	-2.377523	1.308007	1.198149
5	N	-1.111817	1.437809	1.780653
6	C	-0.119554	2.239655	1.206167
7	C	0.674673	3.72766	-0.506649
8	C	1.946201	3.83295	0.092893
9	C	1.151461	2.331031	1.824739
10	C	2.158838	3.106426	1.284243
11	H	0.503539	4.28518	-1.423084
12	H	1.344008	1.784059	2.739222
13	H	3.11585	3.142632	1.794977
14	C	-3.977054	1.801744	-0.526812
15	C	-4.956875	0.979559	0.065706
16	C	-4.605837	0.3194	1.262981
17	C	-3.349123	0.481191	1.813077
18	H	-4.241827	2.316095	-1.446246
19	H	-5.314482	-0.32723	1.770548
20	H	-3.10783	-0.038652	2.731782
21	C	-6.248012	0.855998	-0.581146
22	C	-7.310273	0.114118	-0.159611
23	H	-6.343582	1.429447	-1.501333
24	C	-8.581121	0.007512	-0.839957
25	H	-7.232741	-0.461008	0.759314
26	C	2.950201	4.664793	-0.540091
27	C	4.221488	4.891396	-0.105443
28	H	2.619123	5.145148	-1.459035
29	C	5.197444	5.725491	-0.769441
30	H	4.566122	4.424351	0.813446
31	C	-8.893027	0.654899	-2.06564
32	C	-10.127214	0.490924	-2.647706
33	N	-11.063436	-0.292097	-2.055328
34	C	-9.602268	-0.796344	-0.268271
35	H	-8.17398	1.286361	-2.572221
36	H	-10.411765	0.960697	-3.580636
37	C	4.966926	6.41318	-1.991057

38	C	5.953967	7.186103	-2.554137
39	N	7.162054	7.300447	-1.947515
40	C	7.439767	6.667565	-0.782372
41	H	5.827624	7.728567	-3.482551
42	H	4.018028	6.347073	-2.508451
43	C	-0.836597	0.762399	3.029493
44	C	-0.3282	-0.55546	3.033518
45	C	-1.094077	1.447591	4.219874
46	C	-0.079876	-1.145719	4.28929
47	C	-0.335616	-0.465769	5.477608
48	C	-0.846362	0.837253	5.449013
49	H	-1.488636	2.458652	4.171235
50	H	-1.045616	1.372591	6.372774
51	C	-10.824096	-0.932024	-0.885471
52	H	-9.430146	-1.316947	0.667312
53	H	-11.63149	-1.534315	-0.488572
54	C	6.481194	5.883147	-0.183935
55	H	6.722897	5.384564	0.748229
56	H	8.430728	6.817023	-0.372906
57	H	-0.130516	-0.950416	6.428113
58	H	0.330708	-2.149556	4.336392
59	C	-0.073533	-1.25242	1.761603
60	C	0.197254	-2.559142	1.617368
61	H	-0.12659	-0.639357	0.867764
62	C	0.435454	-3.236178	0.301311
63	H	0.231489	-3.228419	2.473592
64	N	-0.104939	-2.422789	-0.841673
65	N	-0.089231	-3.010943	-1.925245
66	N	-0.393131	-4.519349	0.278074
67	N	-0.378133	-5.096908	-0.805837
68	C	0.467473	-4.410469	-1.865145
69	C	0.229827	-5.093037	-3.197492
70	H	0.748897	-4.575156	-4.007741
71	H	-0.84294	-5.073575	-3.412887
72	H	0.554624	-6.136017	-3.175558
73	C	-2.304651	4.345619	-0.772664
74	H	-2.471612	4.734007	0.237936
75	H	-3.261291	4.353654	-1.305212
76	H	-1.628554	5.030018	-1.295529
77	C	-1.488925	2.389534	-2.167021
78	H	-1.07134	1.377048	-2.155525
79	H	-0.799669	3.038671	-2.716402
80	H	-2.431279	2.365405	-2.723489
81	C	4.392203	-3.481827	0.803333
82	C	2.885891	-3.25185	0.971718
83	C	4.775663	-4.947424	0.750898
84	C	1.874079	-3.655591	-0.086823

85	C	4.795731	-5.674126	-0.572575
86	C	1.887736	-4.297557	-1.274886
87	C	4.43397	-4.92859	-1.841221
88	C	2.933931	-4.949376	-2.163995
89	C	6.097316	-5.436215	0.182099
90	C	7.19218	-4.514547	-0.304152
91	O	8.098945	-5.178766	-1.205866
92	H	4.871777	-3.015071	1.674745
93	H	4.756474	-2.928796	-0.06636
94	H	2.737421	-2.180607	1.15821
95	H	2.578508	-3.741332	1.908524
96	H	4.411298	-5.513695	1.608393
97	H	4.443302	-6.705643	-0.564206
98	H	4.928554	-5.408134	-2.697782
99	H	4.797717	-3.897524	-1.828918
100	H	2.639282	-6.002477	-2.280438
101	H	2.819169	-4.512916	-3.164515
102	H	6.500698	-6.320307	0.675432
103	H	6.794729	-3.6115	-0.781877
104	H	7.818898	-4.194987	0.534675
105	H	7.58113	-5.531634	-1.94814
106	H	7.878241	7.876947	-2.379445
107	H	-11.968431	-0.402996	-2.503259

Acri-ovi-H-TS2

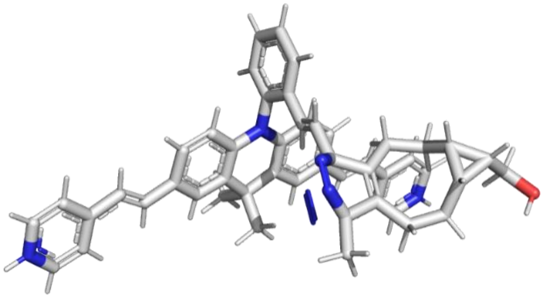

Tag	Symbol	X	Y	Z
1	C	2.493106	-2.054729	-0.051904
2	C	1.420641	-2.869455	-0.785512
3	C	0.092495	-2.84836	-0.019018
4	C	2.249868	-1.404006	1.179008
5	N	0.991148	-1.462984	1.787746
6	C	-0.070444	-2.166635	1.208762
7	C	-1.011112	-3.524786	-0.537538
8	C	-2.272844	-3.560877	0.090296
9	C	-1.330382	-2.188948	1.855625
10	C	-2.404368	-2.866916	1.31238
11	H	-0.902703	-4.056951	-1.478334
12	H	-1.460887	-1.66618	2.794935
13	H	-3.349279	-2.853761	1.846205
14	C	3.769523	-1.946297	-0.602901
15	C	4.822985	-1.228868	-0.000757
16	C	4.549106	-0.593525	1.229654
17	C	3.295325	-0.680252	1.802622
18	H	3.973578	-2.439979	-1.548836
19	H	5.31626	-0.024672	1.745236
20	H	3.114504	-0.181642	2.746603

21	C	6.10808	-1.181275	-0.669428
22	C	7.235915	-0.552263	-0.23492
23	H	6.140164	-1.716492	-1.616717
24	C	8.500412	-0.519537	-0.934138
25	H	7.223185	-0.021647	0.713487
26	C	-3.348698	-4.295065	-0.545593
27	C	-4.627196	-4.432852	-0.095221
28	H	-3.071149	-4.772948	-1.48333
29	C	-5.674605	-5.173159	-0.761675
30	H	-4.922995	-3.957881	0.836504
31	C	8.733614	-1.118556	-2.201087
32	C	9.970021	-1.036097	-2.795655
33	N	10.983425	-0.380004	-2.176728
34	C	9.598898	0.153612	-0.337696
35	H	7.951186	-1.648258	-2.729951
36	H	10.196545	-1.474167	-3.759353
37	C	-5.504828	-5.882873	-1.980603
38	C	-6.559008	-6.557759	-2.548024
39	N	-7.775659	-6.553595	-1.947959
40	C	-7.996409	-5.897316	-0.783466
41	H	-6.480851	-7.112748	-3.474242
42	H	-4.55035	-5.912677	-2.491107
43	C	0.796742	-0.821727	3.069232
44	C	0.400598	0.532014	3.137
45	C	1.023855	-1.572232	4.225692
46	C	0.228578	1.090898	4.419423
47	C	0.455425	0.345794	5.574421
48	C	0.856144	-0.992208	5.483097
49	H	1.333088	-2.609245	4.129227
50	H	1.031135	-1.578431	6.380508
51	C	10.819907	0.210873	-0.968505
52	H	9.487951	0.633246	0.628544
53	H	11.685066	0.711707	-0.552786
54	C	-6.969297	-5.209156	-0.180207
55	H	-7.165678	-4.689716	0.751241
56	H	-8.999052	-5.950974	-0.378678
57	H	0.310727	0.806546	6.547661
58	H	-0.103795	2.120346	4.511727
59	C	0.17286	1.301866	1.902884
60	C	0.128522	2.640777	1.811868
61	H	0.050598	0.715881	0.996519
62	C	-0.09209	3.403198	0.546901
63	H	0.304198	3.270243	2.681943
64	N	0.646537	2.518646	-0.742756
65	N	0.667742	3.139626	-1.75046
66	N	0.710413	4.598879	0.555689
67	N	0.742783	5.239024	-0.528829

68	C	-0.034012	4.681181	-1.605943
69	C	0.264555	5.390912	-2.907159
70	H	-0.167505	4.865791	-3.76196
71	H	1.349243	5.436351	-3.041554
72	H	-0.117547	6.415914	-2.893572
73	C	1.906	-4.339396	-0.919982
74	H	2.069899	-4.786492	0.066597
75	H	2.845479	-4.391111	-1.479878
76	H	1.168827	-4.948091	-1.453651
77	C	1.200837	-2.265142	-2.19963
78	H	0.854292	-1.228475	-2.129466
79	H	0.455404	-2.838811	-2.759686
80	H	2.129305	-2.277411	-2.779334
81	C	-4.062383	3.302654	0.654428
82	C	-2.565024	3.075327	0.892487
83	C	-4.460677	4.765094	0.652966
84	C	-1.47949	3.668663	0.002153
85	C	-4.427746	5.54087	-0.642764
86	C	-1.44549	4.368027	-1.165533
87	C	-3.994237	4.834413	-1.911202
88	C	-2.480127	4.887743	-2.157031
89	C	-5.759764	5.263721	0.042144
90	C	-6.822614	4.352504	-0.527444
91	O	-7.694611	5.0436	-1.443259
92	H	-4.580812	2.784418	1.473024
93	H	-4.376224	2.792781	-0.259463
94	H	-2.400234	1.990443	0.919393
95	H	-2.343073	3.41124	1.915288
96	H	-4.140819	5.298822	1.548219
97	H	-4.085822	6.574215	-0.583156
98	H	-4.452744	5.324676	-2.781656
99	H	-4.343155	3.799295	-1.939982
100	H	-2.229002	5.940227	-2.343688
101	H	-2.291063	4.380055	-3.113355
102	H	-6.193485	6.125703	0.548763
103	H	-6.394883	3.472124	-1.021017
104	H	-7.483064	3.995519	0.269393
105	H	-7.146768	5.433967	-2.144082
106	H	-8.542062	-7.057598	-2.384565
107	H	11.889521	-0.328315	-2.633494

Clicked Acri-ovi

Tag	Symbol	X	Y	Z
1	C	-2.467149	-1.701985	0.186761
2	C	-1.343929	-2.416424	-0.576039
3	C	-0.039944	-2.404027	0.231945
4	C	-2.23755	-0.990907	1.389145
5	N	-0.967105	-0.947987	1.965699
6	C	0.103431	-1.668079	1.432438
7	C	1.073854	-3.093013	-0.238924
8	C	2.326106	-3.100536	0.414478
9	C	1.351017	-1.661046	2.10565
10	C	2.432586	-2.358336	1.612859
11	H	0.987772	-3.649581	-1.167892
12	H	1.458502	-1.099022	3.024547
13	H	3.366194	-2.324545	2.165246
14	C	-3.759023	-1.692794	-0.330654
15	C	-4.84332	-1.021642	0.27677
16	C	-4.578254	-0.321203	1.47563
17	C	-3.309685	-0.305303	2.014068
18	H	-3.950921	-2.218771	-1.261588
19	H	-5.366721	0.217721	1.991174
20	H	-3.131225	0.241813	2.930992
21	C	-6.139673	-1.079042	-0.351396
22	C	-7.301449	-0.50445	0.087882
23	H	-6.16432	-1.652383	-1.277416
24	C	-8.577248	-0.583374	-0.568056
25	H	-7.293506	0.068299	1.011643
26	C	3.412912	-3.848577	-0.166602
27	C	4.687931	-3.969731	0.315811
28	H	3.159161	-4.363058	-1.092795
29	C	5.747518	-4.726651	-0.291065
30	H	4.956563	-3.459775	1.237324
31	C	-8.827155	-1.268942	-1.790173
32	C	-10.082242	-1.287925	-2.345746
33	N	-11.136402	-0.654234	-1.75797
34	C	-9.705359	0.060999	0.010223
35	H	-8.036311	-1.789382	-2.317131
36	H	-10.295472	-1.803518	-3.275235
37	C	-12.484281	-0.743316	-2.356381
38	H	-12.395631	-0.751631	-3.443801
39	H	-13.068794	0.126352	-2.054092
40	H	-12.981883	-1.657013	-2.018514
41	C	5.631323	-5.47567	-1.495818
42	C	6.702519	-6.167974	-2.00339
43	N	7.916205	-6.167149	-1.382768
44	C	8.070161	-5.465758	-0.226142

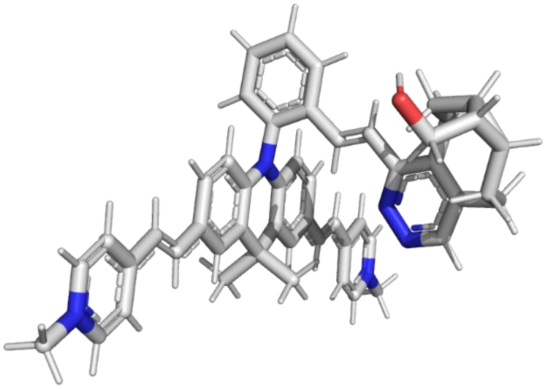

45	H	6.636722	-6.74543	-2.918511
46	H	4.698884	-5.518216	-2.046056
47	C	9.030644	-6.969361	-1.927978
48	H	8.974145	-6.970328	-3.017532
49	H	8.96898	-7.995231	-1.553265
50	H	9.977248	-6.522713	-1.621586
51	C	-10.941773	0.011504	-0.586562
52	H	-9.60357	0.609846	0.940752
53	H	-11.81259	0.495642	-0.159601
54	C	7.029783	-4.756521	0.323189
55	H	7.207581	-4.209894	1.243549
56	H	9.053689	-5.500031	0.228318
57	C	-1.10241	-1.669101	-1.918656
58	H	-0.800232	-0.630128	-1.75078
59	H	-2.012698	-1.665541	-2.528368
60	H	-0.315172	-2.162903	-2.499057
61	C	-1.763409	-3.8838	-0.857974
62	H	-1.939109	-4.430075	0.07485
63	H	-0.991916	-4.414241	-1.424201
64	H	-2.678696	-3.926004	-1.456046
65	C	-0.733475	-0.075901	3.100683
66	C	-0.29792	1.250859	2.876128
67	C	-0.927656	-0.578874	4.388364
68	C	-0.106045	1.731729	1.505513
69	C	-0.07918	2.050884	4.016638
70	C	-0.696364	0.23538	5.497541
71	H	-1.256855	-1.607089	4.513136
72	C	0.581005	2.825514	1.116361
73	H	-0.542553	1.132455	0.713943
74	C	-0.271174	1.554976	5.303607
75	H	0.227645	3.084585	3.887776
76	H	-0.849765	-0.153043	6.499991
77	C	0.742516	3.20607	-0.301241
78	H	1.07172	3.449264	1.856114
79	H	-0.097596	2.200858	6.159783
80	C	1.395743	4.40124	-0.705118
81	N	0.24107	2.315799	-1.184342
82	C	1.529059	4.597719	-2.088584
83	C	1.948932	5.40008	0.287962
84	N	0.334204	2.524644	-2.485818
85	C	2.274015	5.752656	-2.720754
86	C	0.960712	3.622047	-2.919959
87	C	3.488071	5.250729	0.530677
88	H	1.748382	6.413313	-0.077324
89	H	1.411244	5.322112	1.235421
90	C	3.8283	5.627704	-2.60033
91	H	1.995552	5.792152	-3.779235

92	H	1.951532	6.705983	-2.28456
93	H	1.006027	3.722761	-4.00208
94	C	4.285803	6.395	-0.076633
95	H	3.67694	5.228611	1.612487
96	H	3.823003	4.281427	0.146667
97	C	4.439491	6.572328	-1.574067
98	H	4.27359	5.857903	-3.576192
99	H	4.088605	4.58458	-2.395473
100	C	5.655831	6.232641	-0.72772
101	H	4.156571	7.32613	0.475612
102	H	4.406111	7.610039	-1.906672
103	C	6.373859	4.902819	-0.797618
104	H	6.34712	7.057093	-0.557503
105	O	7.119144	4.62611	0.392254
106	H	5.682813	4.074307	-1.005774
107	H	7.116309	4.917088	-1.602201
108	H	6.563245	4.837143	1.159047

Clicked Acri-ovi-H

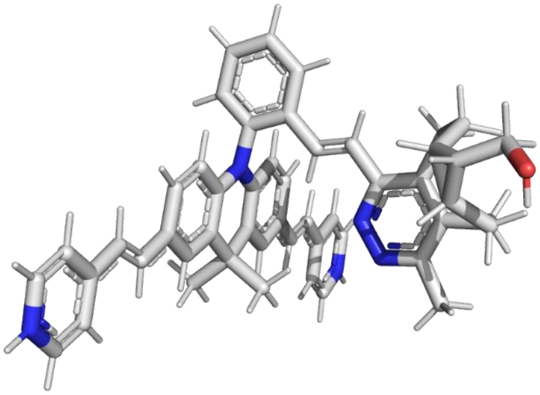

Tag	Symbol	X	Y	Z
1	C	0.355706	2.760523	-0.0443
2	C	1.602079	2.473363	-0.891161
3	C	2.656189	1.717339	-0.072076
4	C	0.166653	2.199859	1.242371
5	N	1.148304	1.399608	1.827244
6	C	2.386228	1.197308	1.217426
7	C	3.912469	1.470061	-0.615581
8	C	4.92517	0.740055	0.047338
9	C	3.384455	0.455174	1.898822
10	C	4.620658	0.23429	1.332604
11	H	4.133169	1.845293	-1.610739
12	H	3.17332	0.055144	2.882251
13	H	5.35307	-0.337067	1.893742
14	C	-0.667082	3.549863	-0.559353
15	C	-1.869849	3.824716	0.130311
16	C	-2.025947	3.246942	1.411843
17	C	-1.035901	2.454585	1.949673
18	H	-0.548538	3.974886	-1.551886
19	H	-2.927078	3.416176	1.992441
20	H	-1.179437	2.021286	2.931114
21	C	-2.856842	4.662619	-0.498374
22	C	-4.066151	5.052226	0.016222
23	H	-2.580735	5.013449	-1.491985
24	C	-5.024322	5.893439	-0.639594
25	H	-4.352532	4.713342	1.008347
26	C	6.190724	0.549391	-0.611495

27	C	7.288609	-0.112803	-0.126961
28	H	6.252116	0.99546	-1.603384
29	C	8.536587	-0.280771	-0.813001
30	H	7.242733	-0.55939	0.862801
31	C	-4.863015	6.447776	-1.94473
32	C	-5.832637	7.245629	-2.495173
33	N	-6.971898	7.523769	-1.802857
34	C	-6.239048	6.221542	0.032172
35	H	-3.974269	6.252905	-2.532505
36	H	-5.748927	7.685719	-3.482011
37	C	8.820067	0.215071	-2.120745
38	C	10.044903	0.008787	-2.701319
39	N	11.016283	-0.676372	-2.036919
40	C	10.805915	-1.174224	-0.788099
41	H	10.300156	0.368413	-3.691294
42	H	8.077896	0.76451	-2.686976
43	C	0.851794	0.714841	3.072281
44	C	0.253022	-0.566237	3.032898
45	C	1.157532	1.351392	4.276871
46	C	-0.016214	-1.176771	4.276099
47	C	0.285019	-0.547607	5.480694
48	C	0.874649	0.722421	5.489218
49	H	1.613923	2.337574	4.256108
50	H	1.113088	1.214288	6.427527
51	C	-7.183871	7.024069	-0.555007
52	H	-6.431128	5.835876	1.027833
53	H	-8.117298	7.295393	-0.075767
54	C	9.594589	-0.990068	-0.171314
55	H	9.45148	-1.39627	0.824425
56	H	11.632632	-1.706521	-0.332279
57	H	0.065783	-1.050537	6.418415
58	H	-0.457416	-2.168678	4.29578
59	C	-0.044118	-1.192107	1.742405
60	C	-0.777668	-2.302841	1.521249
61	H	0.355093	-0.70104	0.862071
62	C	-1.033678	-2.833639	0.16654
63	H	-1.222226	-2.830651	2.357641
64	N	-0.65145	-2.010754	-0.831156
65	N	-0.814868	-2.345267	-2.09612
66	C	-1.394865	-3.515095	-2.416416
67	C	-1.523295	-3.773741	-3.896163
68	H	-0.931248	-4.644037	-4.207036
69	H	-1.161802	-2.899981	-4.443027
70	H	-2.561465	-3.965663	-4.191215
71	C	2.203108	3.809891	-1.401319
72	H	2.505707	4.451119	-0.566647
73	H	1.482042	4.361237	-2.011939

74	H	3.079	3.634995	-2.03305
75	C	1.183943	1.587237	-2.099575
76	H	0.746817	0.638387	-1.771456
77	H	2.050591	1.364292	-2.731917
78	H	0.443435	2.105789	-2.718716
79	C	-3.477327	-5.564768	1.097453
80	C	-1.999474	-5.061266	1.032841
81	C	-4.476188	-4.599745	0.486494
82	C	-1.637417	-4.094657	-0.087309
83	C	-4.753978	-4.610802	-1.006955
84	C	-1.834903	-4.428932	-1.435471
85	C	-4.073773	-5.590393	-1.965567
86	C	-2.519807	-5.714424	-1.83592
87	C	-5.842538	-5.017497	-0.016758
88	C	-6.389185	-6.425149	0.092716
89	O	-7.487753	-6.643121	-0.793032
90	H	-3.726066	-5.736934	2.152731
91	H	-3.554347	-6.543071	0.616567
92	H	-1.336683	-5.934914	0.954281
93	H	-1.750946	-4.60909	1.993135
94	H	-4.447311	-3.608969	0.942038
95	H	-4.89379	-3.622766	-1.442963
96	H	-4.314914	-5.269621	-2.985983
97	H	-4.483858	-6.602521	-1.871838
98	H	-2.123893	-6.068927	-2.792595
99	H	-2.275871	-6.492793	-1.108053
100	H	-6.628923	-4.276686	0.123154
101	H	-5.612004	-7.185909	-0.065478
102	H	-6.800619	-6.588852	1.093852
103	H	-7.195908	-6.475968	-1.70331
104	H	11.9167	-0.818586	-2.483138
105	H	-7.677949	8.11678	-2.226987

Acri-mvi

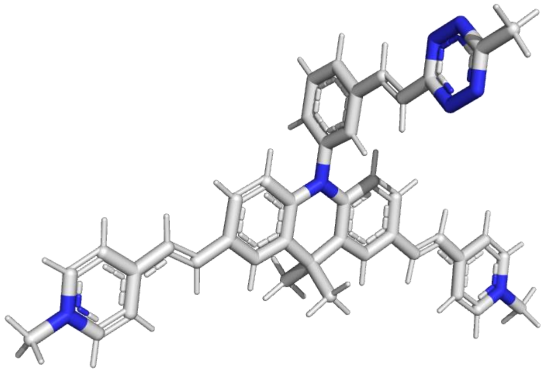

Tag	Symbol	X	Y	Z
1	C	1.277445	-1.769292	0.152649
2	C	0.016196	-2.509588	-0.309489
3	C	-1.25106	-1.781483	0.155522
4	C	1.230117	-0.555901	0.8761
5	N	0.005779	0.030218	1.215644
6	C	-1.21365	-0.568258	0.879813
7	C	-2.501667	-2.318973	-0.147484
8	C	-3.722048	-1.724914	0.231402
9	C	-2.428753	0.047105	1.268555
10	C	-3.651293	-0.515229	0.954768
11	H	-2.548467	-3.248381	-0.707514

12	H	-2.407757	0.976915	1.822748
13	H	-4.553952	-0.004396	1.274754
14	C	2.532422	-2.295144	-0.152556
15	C	3.747921	-1.689207	0.223231
16	C	3.66731	-0.478844	0.944436
17	C	2.440142	0.072233	1.260216
18	H	2.586826	-3.224869	-0.711393
19	H	4.565819	0.041309	1.261084
20	H	2.411404	1.002871	1.812693
21	C	4.993041	-2.334085	-0.147383
22	C	6.254535	-1.919966	0.15438
23	H	4.868155	-3.249042	-0.724043
24	C	7.473036	-2.595251	-0.235438
25	H	6.398637	-1.015901	0.740509
26	C	-4.962027	-2.379686	-0.1392
27	C	-6.226632	-1.974272	0.161364
28	H	-4.830112	-3.293938	-0.715438
29	C	-7.440338	-2.657095	-0.230216
30	H	-6.377336	-1.070977	0.746992
31	C	7.525053	-3.769737	-1.029805
32	C	8.730488	-4.34586	-1.351256
33	N	9.908428	-3.816453	-0.922828
34	C	8.720916	-2.075903	0.185258
35	H	6.625708	-4.241914	-1.405774
36	H	8.802367	-5.240855	-1.957456
37	C	11.178643	-4.463654	-1.311088
38	H	11.145279	-5.51823	-1.032334
39	H	11.99863	-3.97399	-0.788294
40	H	11.318911	-4.366383	-2.389729
41	C	-7.484037	-3.831774	-1.024773
42	C	-8.685561	-4.414654	-1.348781
43	N	-9.867332	-3.891991	-0.922708
44	C	-9.869053	-2.768273	-0.163302
45	H	-8.751209	-5.309828	-1.955402
46	H	-6.581266	-4.29877	-1.399033
47	C	-11.133223	-4.544969	-1.315313
48	H	-11.09167	-5.601757	-1.046354
49	H	-11.275687	-4.438847	-2.392865
50	H	-11.956088	-4.066273	-0.786887
51	C	0.000267	1.283864	1.932782
52	C	-0.011884	2.477081	1.217064
53	C	0.005088	1.28502	3.333204
54	C	-0.020171	3.713858	1.892892
55	C	-0.016541	3.704295	3.301674
56	C	-0.003174	2.503969	4.013836
57	H	-0.017556	2.433565	0.132668
58	H	0.014197	0.342995	3.873558

59	H	0.000013	2.517492	5.09977
60	C	9.902199	-2.692602	-0.163669
61	H	8.766956	-1.180235	0.795153
62	H	10.86798	-2.314636	0.147982
63	C	-8.692013	-2.144969	0.188141
64	H	-8.744407	-1.249581	0.797959
65	H	-10.837625	-2.395792	0.146284
66	H	-0.023593	4.648981	3.839111
67	C	-0.034691	5.004122	1.199847
68	C	-0.013863	5.204468	-0.135931
69	H	-0.064596	5.878666	1.846287
70	C	-0.03378	6.520443	-0.761098
71	H	0.020457	4.380413	-0.842694
72	N	-0.002377	6.552966	-2.110985
73	N	-0.082792	7.635751	0.007275
74	N	-0.102287	8.800882	-0.591953
75	N	-0.022573	7.729349	-2.707885
76	C	-0.073092	8.833201	-1.943513
77	C	-0.097552	10.171867	-2.610786
78	H	-0.068181	10.053256	-3.695661
79	H	0.760927	10.772725	-2.29043
80	H	-1.004979	10.719413	-2.332526
81	C	0.023633	-3.947043	0.279773
82	H	0.024699	-3.917925	1.374803
83	H	0.908204	-4.501768	-0.04914
84	H	-0.856449	-4.509984	-0.047204
85	C	0.014958	-2.587247	-1.86155
86	H	0.009759	-1.583736	-2.300846
87	H	-0.866788	-3.124969	-2.225558
88	H	0.901027	-3.116594	-2.227337

Acri-mvi-H

Tag	Symbol	X	Y	Z
1	C	1.269507	-2.006822	0.028159
2	C	0.006929	-2.741147	-0.439522
3	C	-1.25884	-2.012601	0.028559
4	C	1.224403	-0.799924	0.762941
5	N	0.001446	-0.215718	1.109099
6	C	-1.218963	-0.805699	0.763632
7	C	-2.510312	-2.544035	-0.279779
8	C	-3.729518	-1.948718	0.102229
9	C	-2.432715	-0.188235	1.154018
10	C	-3.656279	-0.743693	0.833648
11	H	-2.559428	-3.469475	-0.846072
12	H	-2.409361	0.737075	1.715475
13	H	-4.557802	-0.232646	1.156252

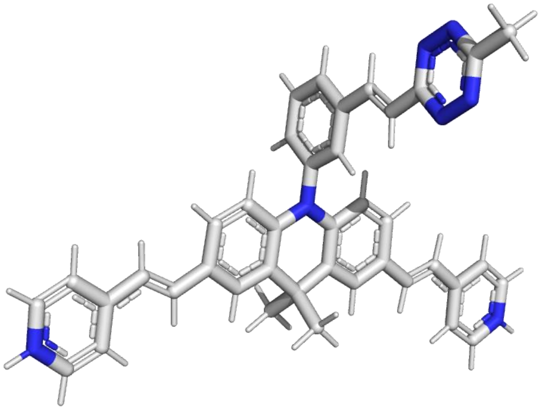

14	C	2.523277	-2.532687	-0.280331
15	C	3.739873	-1.931886	0.101415
16	C	3.661408	-0.726733	0.832105
17	C	2.435426	-0.176574	1.152401
18	H	2.576431	-3.458095	-0.84632
19	H	4.560717	-0.211389	1.154058
20	H	2.407945	0.749005	1.713247
21	C	4.982963	-2.577491	-0.270061
22	C	6.245844	-2.159882	0.025321
23	H	4.856741	-3.495416	-0.841507
24	C	7.463049	-2.837335	-0.359891
25	H	6.389953	-1.249759	0.601778
26	C	-4.969858	-2.59923	-0.269896
27	C	-6.234535	-2.185578	0.023383
28	H	-4.839728	-3.51725	-0.840315
29	C	-7.448981	-2.867498	-0.362664
30	H	-6.382451	-1.27511	0.598332
31	C	7.508745	-4.029302	-1.131792
32	C	8.713375	-4.609046	-1.4505
33	N	9.874656	-4.046652	-1.030987
34	C	8.710624	-2.296068	0.047575
35	H	6.605486	-4.508385	-1.488288
36	H	8.800693	-5.516034	-2.03522
37	C	-7.489633	-4.061292	-1.132013
38	C	-8.691903	-4.645421	-1.45162
39	N	-9.855653	-4.085619	-1.035521
40	C	-9.879049	-2.94735	-0.301349
41	H	-8.775362	-5.554034	-2.034374
42	H	-6.584278	-4.538514	-1.485673
43	C	-0.001442	1.018734	1.859442
44	C	-0.00738	2.231313	1.177254
45	C	-0.000755	0.980041	3.259332
46	C	-0.013049	3.448473	1.887981
47	C	-0.014897	3.399103	3.295918
48	C	-0.007541	2.179197	3.97408
49	H	-0.011492	2.21836	0.092063
50	H	0.003428	0.022949	3.772593
51	H	-0.008062	2.16211	5.059954
52	C	9.893211	-2.910011	-0.294158
53	H	8.746481	-1.387359	0.638098
54	H	10.866897	-2.534708	-0.005523
55	C	-8.69896	-2.329067	0.041211
56	H	-8.738682	-1.419104	0.629552
57	H	-10.854424	-2.574318	-0.015487
58	H	-0.020992	4.328203	3.859911
59	C	-0.020381	4.757999	1.232037
60	C	0.021688	4.99733	-0.09678

61	H	-0.063075	5.61347	1.902778
62	C	0.004965	6.331393	-0.682632
63	H	0.072212	4.194681	-0.826827
64	N	0.058873	6.405104	-2.030117
65	N	-0.063637	7.42251	0.118318
66	N	-0.083022	8.605133	-0.445473
67	N	0.03859	7.598985	-2.591323
68	C	-0.034355	8.67862	-1.79487
69	C	-0.061347	10.036615	-2.421719
70	H	-0.017465	9.951112	-3.50921
71	H	0.788201	10.634111	-2.072551
72	H	-0.976529	10.568454	-2.138539
73	C	0.010251	-4.181847	0.141814
74	H	0.010314	-4.158827	1.236976
75	H	0.893817	-4.736782	-0.189393
76	H	-0.870888	-4.740737	-0.189208
77	C	0.006891	-2.809726	-1.991986
78	H	0.004583	-1.803715	-2.425478
79	H	-0.875802	-3.343199	-2.359858
80	H	0.891892	-3.339226	-2.360054
81	H	10.754552	-4.491437	-1.275749
82	H	-10.733719	-4.53374	-1.28075

Acri-mvi-H TS1

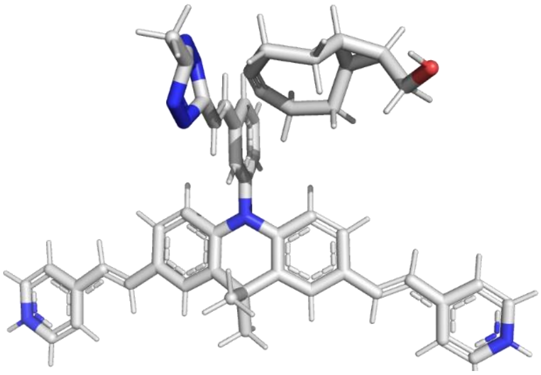

Tag	Symbol	X	Y	Z
1	C	-3.943703	0.412862	-0.439072
2	C	-3.361143	1.563058	-1.267515
3	C	-2.387883	2.358033	-0.390366
4	C	-3.130089	-0.223688	0.530144
5	N	-1.892355	0.335167	0.877784
6	C	-1.610722	1.674766	0.577097
7	C	-2.138428	3.713835	-0.586254
8	C	-1.122839	4.420134	0.094733
9	C	-0.567333	2.354947	1.244953
10	C	-0.326428	3.696074	1.006782
11	H	-2.730661	4.262482	-1.310347
12	H	0.047094	1.829361	1.965239
13	H	0.479214	4.181659	1.548277
14	C	-5.205841	-0.122614	-0.682885
15	C	-5.687613	-1.288135	-0.047766
16	C	-4.828831	-1.932136	0.867719
17	C	-3.580201	-1.409786	1.152713
18	H	-5.853206	0.356317	-1.409247
19	H	-5.137158	-2.841263	1.373918
20	H	-2.950104	-1.915162	1.873852
21	C	-7.016735	-1.761677	-0.381159

22	C	-7.642659	-2.866634	0.112365
23	H	-7.542428	-1.143918	-1.107079
24	C	-8.973445	-3.305446	-0.242151
25	H	-7.130155	-3.497147	0.834159
26	C	-0.94824	5.830584	-0.191002
27	C	-0.011083	6.666953	0.336828
28	H	-1.659307	6.231318	-0.911186
29	C	0.126811	8.072011	0.026853
30	H	0.713752	6.281385	1.04902
31	C	-9.828653	-2.625094	-1.150028
32	C	-11.08312	-3.113586	-1.42708
33	N	-11.517851	-4.25426	-0.835024
34	C	-9.484703	-4.492872	0.34446
35	H	-9.520655	-1.711095	-1.642063
36	H	-11.772243	-2.632099	-2.109329
37	C	-0.729754	8.787636	-0.851991
38	C	-0.524724	10.125369	-1.090941
39	N	0.501175	10.77757	-0.488767
40	C	1.349003	10.148331	0.36006
41	H	-1.149441	10.714422	-1.750373
42	H	-1.561317	8.305568	-1.350492
43	C	-1.017799	-0.38896	1.77213
44	C	-0.020845	-1.1923	1.227879
45	C	-1.180124	-0.282005	3.159813
46	C	0.847999	-1.919755	2.066948
47	C	0.676638	-1.80456	3.459883
48	C	-0.324681	-0.99531	4.000382
49	H	0.069263	-1.246978	0.147528
50	H	-1.964465	0.3494	3.56683
51	H	-0.439346	-0.921391	5.077935
52	C	-10.746685	-4.946106	0.037766
53	H	-8.882994	-5.06034	1.045735
54	H	-11.178653	-5.845025	0.459034
55	C	1.179643	8.809867	0.629071
56	H	1.868014	8.32343	1.311313
57	H	2.137581	10.750595	0.793074
58	H	1.336889	-2.357446	4.123193
59	C	1.914923	-2.789162	1.559293
60	C	2.217271	-3.029379	0.267265
61	H	2.506989	-3.288229	2.32405
62	C	3.288262	-3.923097	-0.17206
63	H	1.669291	-2.561506	-0.546462
64	N	3.253968	-4.300542	-1.503196
65	N	3.835571	-4.816833	0.730526
66	N	4.800487	-5.550246	0.305931
67	N	4.213182	-5.044643	-1.92374
68	C	5.209971	-5.348221	-1.006161

69	C	6.290627	-6.271748	-1.499952
70	H	6.650123	-5.967253	-2.484682
71	H	5.873245	-7.281617	-1.590777
72	H	7.126779	-6.316081	-0.799679
73	C	-4.458361	2.45575	-1.877755
74	H	-5.07474	2.929696	-1.105712
75	H	-5.111026	1.875728	-2.535752
76	H	-4.020852	3.239305	-2.502246
77	C	-2.539231	0.935085	-2.43528
78	H	-1.741467	0.28526	-2.060252
79	H	-2.081376	1.725761	-3.040998
80	H	-3.195209	0.33651	-3.077954
81	C	5.889132	-0.233452	-0.073115
82	C	4.750194	-1.275513	0.073778
83	C	7.12166	-0.584132	0.744599
84	C	5.20653	-2.601829	-0.380803
85	C	8.235048	-1.488035	0.24351
86	C	6.081583	-3.390603	-0.790092
87	C	8.2342	-2.139599	-1.129914
88	C	7.519332	-3.511817	-1.161048
89	C	8.498419	-0.003252	0.465896
90	C	8.768671	0.970909	-0.65914
91	O	10.168289	1.04136	-0.991486
92	H	5.490679	0.733406	0.263362
93	H	6.12341	-0.120832	-1.136006
94	H	3.879071	-0.944249	-0.50336
95	H	4.425324	-1.328885	1.120518
96	H	6.893373	-0.694372	1.805195
97	H	8.6695	-2.134158	1.006917
98	H	9.270839	-2.314883	-1.450698
99	H	7.780129	-1.491787	-1.886816
100	H	8.021229	-4.201593	-0.470748
101	H	7.624162	-3.942104	-2.162768
102	H	9.085408	0.232081	1.353413
103	H	8.187302	0.739265	-1.559315
104	H	8.505774	1.987501	-0.349551
105	H	10.465568	0.152659	-1.246894
106	H	0.635905	11.766169	-0.679472
107	H	-12.448773	-4.598462	-1.051349

Acri-mvi iEDDA

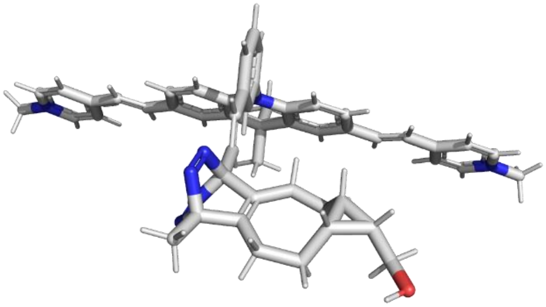

Tag	Symbol	X	Y	Z
1	C	-3.643379	1.481576	0.10449
2	C	-3.058891	2.812303	-0.385433
3	C	-1.653002	3.039658	0.183842
4	C	-2.93052	0.598406	0.947335
5	N	-1.637737	0.90737	1.383258
6	C	-1.007735	2.102854	1.023192
7	C	-0.964477	4.209858	-0.134309
8	C	0.330889	4.508234	0.33416
9	C	0.294933	2.382653	1.50447
10	C	0.948235	3.553338	1.170396
11	H	-1.444736	4.938959	-0.780663
12	H	0.793994	1.669284	2.148036
13	H	1.945069	3.721611	1.565669
14	C	-4.92694	1.107646	-0.292561
15	C	-5.549594	-0.093412	0.102771
16	C	-4.812165	-0.95379	0.944081
17	C	-3.536707	-0.615709	1.353527
18	H	-5.487364	1.773597	-0.942767
19	H	-5.232331	-1.895893	1.28181
20	H	-2.995404	-1.297458	1.997177
21	C	-6.892069	-0.373287	-0.368749
22	C	-7.659559	-1.45682	-0.066464
23	H	-7.301866	0.388338	-1.029888
24	C	-8.998346	-1.697898	-0.558009
25	H	-7.270843	-2.222048	0.600537
26	C	0.946039	5.759937	-0.06289
27	C	2.180809	6.215868	0.286559
28	H	0.324951	6.378046	-0.709088
29	C	2.757375	7.474999	-0.130655
30	H	2.813845	5.613148	0.932728
31	C	-9.692499	-0.848014	-1.45651
32	C	-10.96742	-1.154698	-1.86936
33	N	-11.605537	-2.273928	-1.433815
34	C	-9.697745	-2.853846	-0.132826
35	H	-9.244302	0.059878	-1.841146
36	H	-11.521052	-0.525669	-2.555662
37	C	-12.956473	-2.594242	-1.939042
38	H	-13.522795	-1.669664	-2.052226
39	H	-13.458969	-3.2427	-1.222278
40	H	-12.873118	-3.100702	-2.903806
41	C	2.113803	8.415529	-0.975127
42	C	2.735431	9.593274	-1.315274
43	N	3.979805	9.898177	-0.858877
44	C	4.632124	9.022796	-0.053198

45	H	2.26897	10.330786	-1.956917
46	H	1.121611	8.238308	-1.37133
47	C	4.627399	11.156307	-1.283951
48	H	3.884713	11.95469	-1.289925
49	H	5.046462	11.028972	-2.285065
50	H	5.420721	11.402389	-0.579251
51	C	-0.939713	-0.028735	2.234929
52	C	-0.147336	-1.016342	1.655319
53	C	-1.07208	0.066536	3.624917
54	C	0.538989	-1.94563	2.460493
55	C	0.396461	-1.843818	3.857159
56	C	-0.397212	-0.84963	4.433478
57	H	-0.076062	-1.05582	0.572734
58	H	-1.695205	0.844016	4.057091
59	H	-0.491182	-0.789854	5.51401
60	C	-10.974829	-3.115116	-0.575837
61	H	-9.237245	-3.553725	0.555933
62	H	-11.529999	-3.991379	-0.264752
63	C	4.053158	7.829768	0.317791
64	H	4.611728	7.164908	0.967698
65	H	5.621132	9.315972	0.276699
66	H	0.915508	-2.553628	4.496372
67	C	1.391363	-3.015072	1.913342
68	C	1.681128	-3.226172	0.62002
69	H	1.816654	-3.685739	2.656456
70	C	2.538162	-4.344624	0.109136
71	H	1.278476	-2.584545	-0.160171
72	N	1.839074	-4.955862	-1.099984
73	N	2.618568	-5.472791	1.103783
74	N	3.165854	-6.486465	0.666168
75	N	2.386362	-5.969937	-1.527554
76	C	3.633478	-6.372391	-0.764744
77	C	4.081501	-7.73713	-1.248756
78	H	4.342587	-7.716407	-2.30966
79	H	3.256435	-8.443417	-1.114295
80	H	4.938708	-8.101551	-0.677721
81	C	-3.986239	3.973165	0.068428
82	H	-4.056843	4.009101	1.161016
83	H	-4.995961	3.84972	-0.335967
84	H	-3.607783	4.938691	-0.282197
85	C	-2.978765	2.789486	-1.937078
86	H	-2.33035	1.976062	-2.280661
87	H	-2.577708	3.732842	-2.321889
88	H	-3.970563	2.646196	-2.378276
89	C	5.732982	-2.024044	-0.509013
90	C	4.360218	-2.590157	-0.127917
91	C	6.87576	-2.644056	0.270537

92	C	3.988326	-4.046881	-0.344341
93	C	7.553801	-3.888463	-0.251198
94	C	4.58631	-5.160709	-0.81925
95	C	7.08281	-4.505251	-1.55281
96	C	5.952415	-5.527711	-1.375507
97	C	8.322349	-2.574853	-0.189427
98	C	8.777131	-1.809305	-1.410956
99	O	10.059226	-2.261886	-1.888269
100	H	5.690081	-0.947656	-0.292586
101	H	5.881356	-2.102298	-1.589122
102	H	3.604908	-1.986744	-0.650424
103	H	4.19411	-2.378009	0.937766
104	H	6.737072	-2.584875	1.350339
105	H	7.848861	-4.627878	0.49348
106	H	7.913021	-5.057039	-2.015936
107	H	6.78824	-3.746497	-2.282719
108	H	6.343665	-6.336899	-0.7423
109	H	5.785702	-5.99327	-2.355735
110	H	9.049622	-2.515424	0.620064
111	H	8.045039	-1.852499	-2.225746
112	H	8.930753	-0.754447	-1.161415
113	H	9.991085	-3.210939	-2.083803

Acri-mvi-H iEDDA

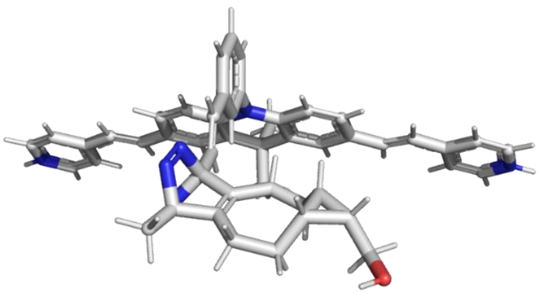

Tag	Symbol	X	Y	Z
1	C	-3.996214	1.040407	-0.007094
2	C	-3.628484	2.451974	-0.480753
3	C	-2.285487	2.898709	0.10979
4	C	-3.176453	0.289772	0.866772
5	N	-1.968507	0.809482	1.342503
6	C	-1.523726	2.084217	0.978993
7	C	-1.779314	4.157354	-0.211272
8	C	-0.55699	4.658586	0.280819
9	C	-0.292914	2.569289	1.486252
10	C	0.179545	3.822325	1.14751
11	H	-2.351687	4.795747	-0.878232
12	H	0.294445	1.950327	2.152522
13	H	1.128158	4.148058	1.562394
14	C	-5.1843	0.456997	-0.445271
15	C	-5.609353	-0.832465	-0.065986
16	C	-4.770857	-1.555075	0.810117
17	C	-3.586778	-1.006992	1.263268
18	H	-5.825515	1.019209	-1.118071
19	H	-5.040552	-2.552526	1.142292
20	H	-2.964164	-1.586049	1.933364
21	C	-6.862001	-1.337291	-0.590967

22	C	-7.426147	-2.552485	-0.342095
23	H	-7.380678	-0.64647	-1.253302
24	C	-8.678398	-3.022474	-0.88888
25	H	-6.921802	-3.25417	0.317084
26	C	-0.132256	5.98233	-0.127215
27	C	1.010046	6.629623	0.237821
28	H	-0.824374	6.484861	-0.800317
29	C	1.398454	7.951132	-0.199549
30	H	1.712985	6.143014	0.908914
31	C	-9.500859	-2.272876	-1.772202
32	C	-10.67905	-2.799273	-2.244981
33	N	-11.069681	-4.043968	-1.871889
34	C	-9.140179	-4.317785	-0.535933
35	H	-9.225121	-1.276651	-2.094859
36	H	-11.339584	-2.269069	-2.919364
37	C	0.629787	8.765048	-1.07443
38	C	1.078398	10.009951	-1.444759
39	N	2.263315	10.477071	-0.976884
40	C	3.037427	9.747579	-0.137662
41	H	0.530169	10.665814	-2.109014
42	H	-0.320775	8.431079	-1.471104
43	C	-1.158254	0.007637	2.231559
44	C	-0.201335	-0.849382	1.694047
45	C	-1.345209	0.104473	3.615008
46	C	0.601091	-1.642334	2.536825
47	C	0.407434	-1.535931	3.927061
48	C	-0.553493	-0.674396	4.46065
49	H	-0.089922	-0.891324	0.614966
50	H	-2.096987	0.779231	4.01386
51	H	-0.685596	-0.61011	5.536918
52	C	-10.326517	-4.805305	-1.032901
53	H	-8.559307	-4.942092	0.133906
54	H	-10.718382	-5.785348	-0.791991
55	C	2.626827	8.495934	0.258919
56	H	3.260828	7.927703	0.930531
57	H	3.965386	10.20251	0.184808
58	H	1.018053	-2.138044	4.595229
59	C	1.628855	-2.571182	2.036189
60	C	1.930154	-2.817423	0.751724
61	H	2.18269	-3.099375	2.808816
62	C	2.977624	-3.785429	0.291093
63	H	1.396349	-2.324422	-0.057283
64	N	2.378138	-4.628637	-0.829829
65	N	3.316947	-4.782263	1.366819
66	N	4.044554	-5.700879	0.985135
67	N	3.106041	-5.546393	-1.201604
68	C	4.43338	-5.622439	-0.471315

69	C	5.134211	-6.907459	-0.864928
70	H	5.348686	-6.931478	-1.936107
71	H	4.476603	-7.749971	-0.629766
72	H	6.067149	-7.038413	-0.311511
73	C	-4.736247	3.443359	-0.028886
74	H	-4.82606	3.454442	1.062821
75	H	-5.707538	3.163641	-0.449726
76	H	-4.511518	4.461063	-0.364295
77	C	-3.529123	2.458262	-2.031227
78	H	-2.753436	1.764599	-2.373467
79	H	-3.283708	3.457791	-2.404475
80	H	-4.479524	2.160929	-2.486022
81	C	5.606488	-0.935648	-0.67846
82	C	4.397558	-1.730642	-0.171998
83	C	6.884606	-1.235318	0.079415
84	C	4.321262	-3.245282	-0.256317
85	C	7.783219	-2.359795	-0.377092
86	C	5.117207	-4.254552	-0.670494
87	C	7.397323	-3.179854	-1.591536
88	C	6.510316	-4.388352	-1.263493
89	C	8.264218	-0.91769	-0.472868
90	C	8.495751	-0.195353	-1.780332
91	O	9.81715	-0.432126	-2.304004
92	H	5.350393	0.12494	-0.548624
93	H	5.722842	-1.081667	-1.755414
94	H	3.512725	-1.337155	-0.690975
95	H	4.239277	-1.465718	0.88313
96	H	6.782633	-1.103725	1.156885
97	H	8.257354	-2.949762	0.407423
98	H	8.304725	-3.594344	-2.053327
99	H	6.923965	-2.569332	-2.36522
100	H	7.082059	-5.036199	-0.583526
101	H	6.41029	-4.970598	-2.188772
102	H	8.998208	-0.63593	0.281859
103	H	7.747986	-0.45641	-2.53815
104	H	8.447055	0.887767	-1.628573
105	H	9.930243	-1.390637	-2.413679
106	H	2.578607	11.399159	-1.265271
107	H	-11.943934	-4.415645	-2.2326

Acri-mvi-H TS2

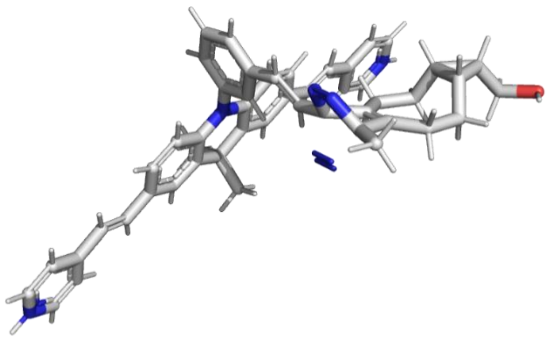

Tag	Symbol	X	Y	Z
1	C	-3.975116	1.04414	-0.023723
2	C	-3.602788	2.453586	-0.500067
3	C	-2.272766	2.908794	0.113022
4	C	-3.172487	0.304449	0.875007
5	N	-1.977583	0.833111	1.373554
6	C	-1.527574	2.104759	1.006034
7	C	-1.762695	4.165395	-0.209816
8	C	-0.551441	4.673868	0.301874
9	C	-0.308346	2.59738	1.533566
10	C	0.168829	3.847799	1.191709
11	H	-2.322849	4.796267	-0.894107
12	H	0.265991	1.986361	2.21829
13	H	1.108051	4.179767	1.622704
14	C	-5.151038	0.452394	-0.483205
15	C	-5.579638	-0.835426	-0.102361
16	C	-4.758226	-1.547114	0.798477
17	C	-3.586768	-0.990471	1.273383
18	H	-5.779226	1.006422	-1.174811
19	H	-5.031722	-2.542584	1.133405
20	H	-2.977406	-1.561332	1.962393
21	C	-6.818791	-1.349854	-0.649721
22	C	-7.380616	-2.567034	-0.405292
23	H	-7.327847	-0.665718	-1.326342
24	C	-8.619936	-3.047366	-0.972153
25	H	-6.884162	-3.262619	0.266214
26	C	-0.121029	5.994334	-0.11075
27	C	1.014912	6.645837	0.266439
28	H	-0.8024	6.49015	-0.799589
29	C	1.408477	7.964098	-0.176183
30	H	1.708435	6.165346	0.951609
31	C	-9.433395	-2.305445	-1.870147
32	C	-10.59976	-2.841504	-2.361098
33	N	-10.987069	-4.088464	-1.99219
34	C	-9.077845	-4.345418	-0.624169
35	H	-9.160203	-1.30739	-2.189317
36	H	-11.253319	-2.317435	-3.04698
37	C	0.64943	8.771913	-1.064996
38	C	1.102376	10.01397	-1.439552
39	N	2.282402	10.484163	-0.962516
40	C	3.047107	9.760696	-0.109548
41	H	0.56139	10.665159	-2.114257
42	H	-0.297188	8.43557	-1.469004
43	C	-1.18858	0.044153	2.292808
44	C	-0.217354	-0.818298	1.791041

45	C	-1.415553	0.153159	3.669416
46	C	0.559624	-1.604434	2.663821
47	C	0.321208	-1.4905	4.046673
48	C	-0.652192	-0.621669	4.544446
49	H	-0.078683	-0.875232	0.715809
50	H	-2.178713	0.831112	4.040235
51	H	-0.817534	-0.549815	5.615629
52	C	-10.252138	-4.842748	-1.139646
53	H	-8.50364	-4.964025	0.056635
54	H	-10.640542	-5.825295	-0.903382
55	C	2.631817	8.512105	0.291784
56	H	3.258241	7.948721	0.974489
57	H	3.971697	10.21768	0.219624
58	H	0.908163	-2.090907	4.737226
59	C	1.599804	-2.537734	2.200966
60	C	2.013499	-2.71273	0.934761
61	H	2.069406	-3.128721	2.983545
62	C	3.051423	-3.696101	0.512902
63	H	1.575341	-2.139622	0.121829
64	N	2.297995	-4.67222	-0.683774
65	N	3.349457	-4.674888	1.524198
66	N	4.067779	-5.636419	1.148806
67	N	2.992265	-5.577151	-1.019109
68	C	4.469168	-5.609715	-0.241991
69	C	5.120484	-6.91859	-0.630532
70	H	5.279276	-6.985411	-1.709261
71	H	4.465713	-7.740647	-0.326538
72	H	6.081171	-7.046151	-0.123347
73	C	-4.722134	3.44434	-0.076051
74	H	-4.832477	3.462813	1.013678
75	H	-5.684416	3.158192	-0.513025
76	H	-4.494413	4.460443	-0.414376
77	C	-3.474024	2.449798	-2.048334
78	H	-2.690108	1.75608	-2.371065
79	H	-3.224337	3.447418	-2.423737
80	H	-4.41474	2.14681	-2.519178
81	C	5.590942	-0.957642	-0.840811
82	C	4.371758	-1.711852	-0.296026
83	C	6.873696	-1.261369	-0.09259
84	C	4.306884	-3.230596	-0.198757
85	C	7.739785	-2.414569	-0.542465
86	C	5.082916	-4.271942	-0.602056
87	C	7.301361	-3.245049	-1.731775
88	C	6.397261	-4.427196	-1.355878
89	C	8.249759	-0.985481	-0.675369
90	C	8.475062	-0.292889	-1.999852
91	O	9.78693	-0.558213	-2.533848

92	H	5.355227	0.111059	-0.742639
93	H	5.692057	-1.137685	-1.913624
94	H	3.503903	-1.389177	-0.889084
95	H	4.175224	-1.329864	0.715189
96	H	6.791721	-1.105306	0.983278
97	H	8.215353	-3.000945	0.24386
98	H	8.182601	-3.68855	-2.216536
99	H	6.820248	-2.634338	-2.499681
100	H	7.01286	-5.120522	-0.767733
101	H	6.173084	-4.970051	-2.285016
102	H	9.002575	-0.70553	0.061328
103	H	7.714892	-0.559318	-2.743306
104	H	8.442832	0.793703	-1.869876
105	H	9.887733	-1.520338	-2.621811
106	H	2.601147	11.403989	-1.254361
107	H	-11.852674	-4.467124	-2.366276

Clicked Acri-mvi

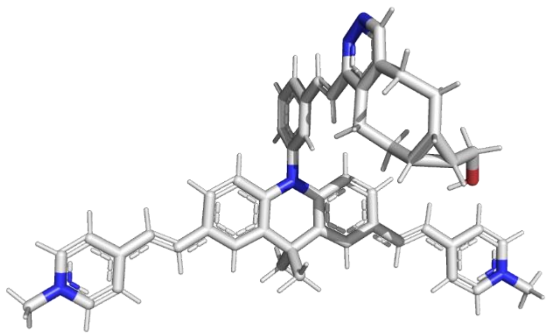

Tag	Symbol	X	Y	Z
1	C	-2.790644	1.87629	0.16969
2	C	-1.824979	2.968394	-0.310202
3	C	-0.443213	2.805594	0.337582
4	C	-2.409774	0.873339	1.093998
5	N	-1.122678	0.841617	1.630036
6	C	-0.154495	1.78082	1.270855
7	C	0.57915	3.695087	0.016222
8	C	1.877485	3.630327	0.567574
9	C	1.137888	1.707283	1.849788
10	C	2.126618	2.604318	1.507234
11	H	0.374921	4.486488	-0.699943
12	H	1.355892	0.933121	2.573913
13	H	3.099421	2.505143	1.977926
14	C	-4.097806	1.851674	-0.309047
15	C	-5.055241	0.885845	0.071954
16	C	-4.63692	-0.109243	0.984916
17	C	-3.350818	-0.114994	1.480064
18	H	-4.408488	2.617582	-1.014591
19	H	-5.320193	-0.886243	1.312401
20	H	-3.056401	-0.888183	2.177959
21	C	-6.387005	0.964272	-0.477117
22	C	-7.44146	0.133546	-0.213595
23	H	-6.538996	1.789716	-1.171795
24	C	-8.760865	0.243604	-0.774039
25	H	-7.30518	-0.69163	0.480603
26	C	2.867183	4.594923	0.151017
27	C	4.172799	4.663678	0.551871

28	H	2.504869	5.331145	-0.565751
29	C	5.139949	5.63368	0.113385
30	H	4.548525	3.93033	1.260606
31	C	-9.174785	1.2399	-1.701714
32	C	-10.461784	1.269285	-2.179296
33	N	-11.392659	0.354113	-1.787739
34	C	-9.762751	-0.691822	-0.396803
35	H	-8.490504	2.00274	-2.053707
36	H	-10.799345	2.018636	-2.886233
37	C	-12.753166	0.38421	-2.364149
38	H	-13.046298	1.420136	-2.540754
39	H	-13.451303	-0.067518	-1.658539
40	H	-12.770047	-0.171321	-3.306278
41	C	4.879914	6.691786	-0.801085
42	C	5.869939	7.572339	-1.162015
43	N	7.133719	7.473617	-0.662038
44	C	7.425636	6.474514	0.21478
45	H	5.695386	8.385691	-1.857121
46	H	3.896923	6.835314	-1.233758
47	C	8.188032	8.404774	-1.115665
48	H	7.745632	9.383511	-1.306746
49	H	8.652334	8.023212	-2.029622
50	H	8.941525	8.501584	-0.333195
51	C	-11.037533	-0.618658	-0.904463
52	H	-9.533566	-1.483662	0.308856
53	H	-11.814032	-1.322772	-0.628217
54	C	6.470757	5.567978	0.608221
55	H	6.75471	4.793076	1.312739
56	H	8.445716	6.438967	0.579765
57	C	-2.400554	4.359733	0.072833
58	H	-2.514176	4.452335	1.157981
59	H	-3.381165	4.520139	-0.38698
60	H	-1.742848	5.165024	-0.270199
61	C	-1.679208	2.871674	-1.853737
62	H	-1.273463	1.89838	-2.149265
63	H	-1.010693	3.649666	-2.236256
64	H	-2.646734	3.001431	-2.349323
65	C	-0.783496	-0.184812	2.598468
66	C	-0.176449	-1.358331	2.160749
67	C	-1.062055	0.030826	3.952268
68	C	0.186583	-2.363406	3.081109
69	H	0.021785	-1.480488	1.099945
70	C	-0.724416	-0.96455	4.871856
71	H	-1.532798	0.95609	4.272148
72	C	0.872884	-3.603119	2.705978
73	C	-0.110109	-2.14097	4.441707
74	H	-0.93526	-0.819022	5.927503

75	C	1.303383	-3.952359	1.475299
76	H	1.078978	-4.294434	3.519464
77	H	0.156769	-2.903428	5.168732
78	C	2.063599	-5.185094	1.196606
79	H	1.129933	-3.287543	0.634651
80	C	2.451828	-5.565461	-0.114944
81	N	2.396068	-5.900173	2.291841
82	C	3.281376	-6.692829	-0.212477
83	C	2.015663	-4.795227	-1.34356
84	N	3.120671	-6.998397	2.188206
85	C	3.909388	-7.182141	-1.499132
86	C	3.564056	-7.368597	0.982875
87	C	3.087433	-3.790088	-1.878067
88	H	1.776186	-5.507503	-2.141321
89	H	1.080578	-4.269411	-1.134609
90	C	5.062817	-6.259579	-2.014282
91	H	4.303219	-8.187982	-1.319928
92	H	3.150732	-7.287774	-2.284861
93	H	4.175734	-8.267944	0.980048
94	C	3.726925	-4.256256	-3.178085
95	H	2.606099	-2.817591	-2.055672
96	H	3.839611	-3.616907	-1.101382
97	C	4.675895	-5.435905	-3.235125
98	H	5.919913	-6.886946	-2.288256
99	H	5.410631	-5.62685	-1.191877
100	C	5.196085	-4.038243	-3.530551
101	H	3.042256	-4.185027	-4.023928
102	H	4.559699	-6.057877	-4.123048
103	C	6.138283	-3.278963	-2.623505
104	H	5.389355	-3.830061	-4.582299
105	O	5.997804	-1.858836	-2.777946
106	H	6.006161	-3.552461	-1.568526
107	H	7.178104	-3.493894	-2.888356
108	H	5.0495	-1.654005	-2.778938

Clicked Acri-mvi-H

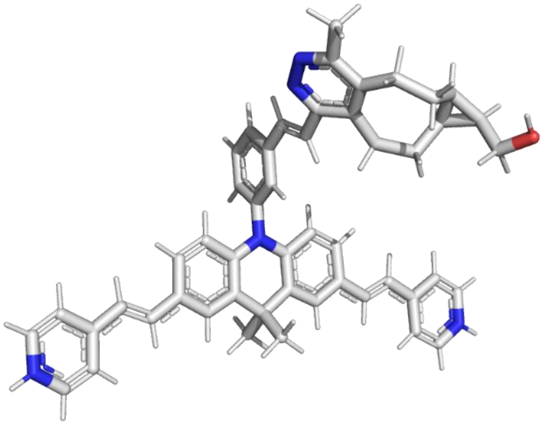

Tag	Symbol	X	Y	Z
1	C	-3.767772	0.860507	-0.147564
2	C	-3.423711	2.269717	-0.648009
3	C	-2.16181	2.805939	0.041181
4	C	-2.986834	0.182905	0.820395
5	N	-1.849097	0.775655	1.368991
6	C	-1.437521	2.05451	0.999264
7	C	-1.691914	4.077726	-0.271782
8	C	-0.541714	4.655633	0.311536
9	C	-0.275305	2.6117	1.59258
10	C	0.160232	3.875717	1.260229
11	H	-2.235922	4.668617	-1.003684
12	H	0.278872	2.035105	2.321952
13	H	1.053568	4.258565	1.743112
14	C	-4.88761	0.203866	-0.649677
15	C	-5.283697	-1.091268	-0.247086
16	C	-4.482011	-1.738	0.72197
17	C	-3.366332	-1.117841	1.240128
18	H	-5.498177	0.708978	-1.393224
19	H	-4.730246	-2.733528	1.075309
20	H	-2.77036	-1.635682	1.98021
21	C	-6.463909	-1.676043	-0.831585
22	C	-6.994632	-2.910253	-0.564999
23	H	-6.966605	-1.041996	-1.560977
24	C	-8.17583	-3.465177	-1.162622
25	H	-6.501397	-3.552292	0.159947
26	C	-0.153234	5.986157	-0.080437
27	C	0.911674	6.711081	0.385845
28	H	-0.800449	6.440781	-0.829513
29	C	1.267675	8.039589	-0.023876
30	H	1.562444	6.271368	1.137073
31	C	-8.983937	-2.804281	-2.134496
32	C	-10.099283	-3.411709	-2.652281
33	N	-10.454719	-4.661194	-2.245173
34	C	-8.60118	-4.770524	-0.778543
35	H	-8.739472	-1.809688	-2.487217
36	H	-10.739996	-2.945025	-3.39136
37	C	0.562217	8.807916	-0.996982
38	C	0.973314	10.074089	-1.326586
39	N	2.066933	10.621058	-0.727968
40	C	2.781262	9.937783	0.206671
41	H	0.466088	10.690583	-2.059533
42	H	-0.31264	8.41331	-1.499253
43	C	-1.093075	0.049156	2.374204
44	C	-0.078528	-0.814553	1.969924

45	C	-1.420169	0.210742	3.724165
46	C	0.649578	-1.556563	2.922727
47	C	0.304246	-1.395456	4.280401
48	C	-0.708595	-0.522156	4.67699
49	H	0.124846	-0.929217	0.909093
50	H	-2.220015	0.885482	4.01589
51	H	-0.950347	-0.416274	5.730748
52	C	-9.724204	-5.342205	-1.321555
53	H	-8.035147	-5.333345	-0.043824
54	H	-10.078748	-6.331062	-1.054968
55	C	2.403799	8.668255	0.565092
56	H	2.989136	8.144067	1.313142
57	H	3.637294	10.448297	0.632331
58	H	0.844077	-1.966701	5.031033
59	C	1.717171	-2.498595	2.569766
60	C	2.345481	-2.597594	1.379949
61	H	2.022639	-3.181414	3.358979
62	C	3.360702	-3.639086	1.098833
63	H	2.095163	-1.902795	0.584455
64	N	3.192393	-4.737371	1.840553
65	N	4.014983	-5.761934	1.705781
66	C	5.002645	-5.711598	0.808983
67	C	5.822827	-6.98018	0.778082
68	H	5.852464	-7.435472	-0.218918
69	H	5.374738	-7.691896	1.473835
70	H	6.862695	-6.808933	1.084269
71	C	-4.612882	3.220931	-0.340585
72	H	-4.804714	3.27478	0.736121
73	H	-5.528588	2.877754	-0.832622
74	H	-4.409983	4.234402	-0.701399
75	C	-3.179526	2.217703	-2.181365
76	H	-2.344691	1.551889	-2.424037
77	H	-2.947861	3.212128	-2.576527
78	H	-4.067844	1.85551	-2.708926
79	C	5.398131	-1.50787	-1.507327
80	C	4.362337	-2.09175	-0.538438
81	C	6.801701	-1.441714	-0.943894
82	C	4.405979	-3.47647	0.126148
83	C	7.713134	-2.630025	-1.142509
84	C	5.265975	-4.579433	-0.034584
85	C	7.16503	-3.834374	-1.878412
86	C	6.458736	-4.844565	-0.963911
87	C	8.026199	-1.310071	-1.831451
88	C	7.96757	-1.097499	-3.330343
89	O	9.218129	-1.37044	-3.96389
90	H	5.04843	-0.489702	-1.733219
91	H	5.366832	-2.037365	-2.461554

92	H	3.393955	-2.0296	-1.056542
93	H	4.286054	-1.377041	0.292054
94	H	6.861609	-0.927398	0.015995
95	H	8.370204	-2.889005	-0.312159
96	H	7.993515	-4.39234	-2.338204
97	H	6.518236	-3.547246	-2.710483
98	H	7.242525	-5.254524	-0.315675
99	H	6.162028	-5.692577	-1.597626
100	H	8.850144	-0.75543	-1.383615
101	H	7.167191	-1.685426	-3.800245
102	H	7.769646	-0.043195	-3.551642
103	H	9.484173	-2.279594	-3.753063
104	H	2.355541	11.559725	-0.984968
105	H	-11.283787	-5.094169	-2.639698

Acri-pvi

Tag	Symbol	X	Y	Z
1	C	-1.244261	-1.994648	0.723544
2	C	-0.000012	-2.621688	1.361883
3	C	1.244242	-1.994653	0.723554
4	C	-1.215807	-0.62645	0.358978
5	N	-0.000001	0.072824	0.378825
6	C	1.215808	-0.626441	0.359024
7	C	2.451075	-2.678075	0.595798
8	C	3.645869	-2.061959	0.164227
9	C	2.408114	0.017827	-0.038896
10	C	3.597164	-0.683778	-0.13173
11	H	2.49428	-3.730377	0.854984
12	H	2.396569	1.071083	-0.290594
13	H	4.489035	-0.151963	-0.447646
14	C	-2.451104	-2.678058	0.595781
15	C	-3.645873	-2.061945	0.16414
16	C	-3.597135	-0.683785	-0.131908
17	C	-2.408081	0.017807	-0.039059
18	H	-2.494337	-3.730345	0.855016
19	H	-4.488981	-0.151986	-0.447925
20	H	-2.396497	1.071044	-0.290837
21	C	-4.847674	-2.869042	0.065462
22	C	-6.078197	-2.463338	-0.352569
23	H	-4.713402	-3.908362	0.35987
24	C	-7.252146	-3.304513	-0.439291
25	H	-6.229605	-1.431603	-0.659406
26	C	4.84766	-2.869071	0.065537
27	C	6.07819	-2.463376	-0.352481
28	H	4.713373	-3.908401	0.359906
29	C	7.252126	-3.304567	-0.439215

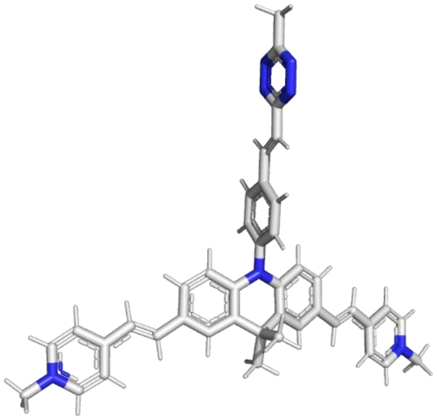

30	H	6.229608	-1.431646	-0.659332
31	C	-7.289014	-4.675765	-0.077076
32	C	-8.449983	-5.401054	-0.195747
33	N	-9.597591	-4.836093	-0.659884
34	C	-8.46742	-2.756249	-0.91469
35	H	-6.412478	-5.187954	0.300292
36	H	-8.508448	-6.449306	0.071534
37	C	-10.817665	-5.662614	-0.77041
38	H	-10.628067	-6.494747	-1.451181
39	H	-11.627739	-5.048235	-1.159994
40	H	-11.087093	-6.041833	0.217292
41	C	7.28903	-4.675765	-0.076809
42	C	8.44998	-5.40108	-0.195519
43	N	9.597534	-4.836193	-0.659873
44	C	9.604766	-3.527357	-1.014817
45	H	8.508464	-6.449297	0.071896
46	H	6.412539	-5.187886	0.300759
47	C	10.817593	-5.662733	-0.770431
48	H	10.627942	-6.494911	-1.451132
49	H	11.087087	-6.041887	0.217278
50	H	11.627645	-5.04839	-1.160115
51	C	-0.00001	1.501628	0.178015
52	C	-0.000123	2.344861	1.295427
53	C	0.000104	2.044707	-1.109697
54	C	-0.000107	3.726501	1.127067
55	C	0.000012	4.296362	-0.162961
56	C	0.000119	3.42895	-1.273801
57	H	0.00019	1.388999	-1.975513
58	H	0.000218	3.846602	-2.27716
59	C	-9.604854	-3.527213	-1.014655
60	H	-8.522885	-1.714441	-1.211174
61	H	-10.544486	-3.129618	-1.377358
62	C	8.467351	-2.756371	-0.91482
63	H	8.522787	-1.714603	-1.211449
64	H	10.544359	-3.129817	-1.377678
65	H	-0.000164	4.359824	2.008486
66	H	-0.000202	1.912678	2.291878
67	C	0.000063	5.740416	-0.408249
68	C	-0.000225	6.718934	0.523109
69	H	0.000372	6.035064	-1.455599
70	C	-0.000101	8.141386	0.2066
71	H	-0.000575	6.502963	1.587449
72	N	-0.000601	8.997076	1.251632
73	N	-0.000474	10.291864	0.998379
74	C	0.000131	10.6922	-0.284233
75	N	0.0006	9.835672	-1.330628
76	N	0.000499	8.548328	-1.086068

77	C	0.000321	12.158143	-0.582253
78	H	-0.000553	12.732185	0.346351
79	H	-0.882701	12.426674	-1.172802
80	H	0.884428	12.426763	-1.171147
81	C	0.000002	-4.158187	1.255857
82	H	-0.000045	-4.495976	0.213471
83	H	-0.873295	-4.582821	1.758535
84	H	0.873365	-4.582794	1.758447
85	C	-0.000006	-2.239664	2.87436
86	H	-0.000001	-1.153642	3.014233
87	H	0.890536	-2.648543	3.365702
88	H	-0.890551	-2.648534	3.365704

Acri-pvi-H

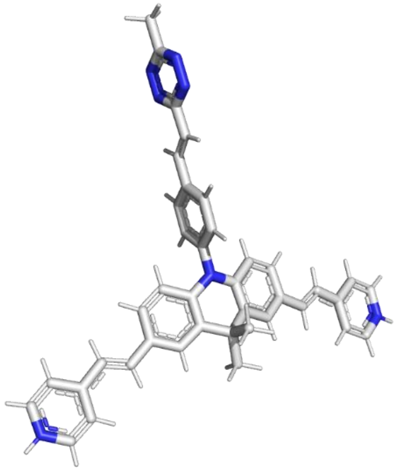

Tag	Symbol	X	Y	Z
1	C	2.393622	-1.057257	0.638911
2	C	2.928262	0.238296	1.258124
3	C	2.183525	1.42243	0.632832
4	C	1.02076	-1.14563	0.301728
5	N	0.222369	0.007052	0.329128
6	C	0.814821	1.277727	0.298226
7	C	2.758862	2.682257	0.490569
8	C	2.033121	3.821462	0.078685
9	C	0.062373	2.411563	-0.081821
10	C	0.657258	3.656333	-0.185676
11	H	3.809502	2.814851	0.724538
12	H	-0.991148	2.310381	-0.311124
13	H	0.044502	4.499641	-0.488016
14	C	3.172453	-2.203476	0.503866
15	C	2.649781	-3.449176	0.092771
16	C	1.266651	-3.517884	-0.176547
17	C	0.470705	-2.390694	-0.07698
18	H	4.229448	-2.15733	0.742422
19	H	0.805031	-4.452796	-0.478153
20	H	-0.584353	-2.468726	-0.308121
21	C	3.553419	-4.578089	-0.012641
22	C	3.242431	-5.848563	-0.393551
23	H	4.585514	-4.347974	0.24539
24	C	4.177391	-6.947169	-0.489681
25	H	2.216826	-6.095016	-0.655755
26	C	2.734141	5.085697	-0.03135
27	C	2.210798	6.28937	-0.396317
28	H	3.793687	5.029703	0.211465
29	C	2.950504	7.527808	-0.493802
30	H	1.154103	6.365883	-0.639075
31	C	5.562907	-6.850808	-0.190642

32	C	6.381069	-7.948501	-0.3101
33	N	5.87219	-9.138748	-0.715899
34	C	3.704632	-8.218534	-0.908772
35	H	6.00934	-5.919996	0.135859
36	H	7.441839	-7.926363	-0.094535
37	C	4.341844	7.653738	-0.236054
38	C	4.966456	8.872035	-0.354418
39	N	4.258634	9.970433	-0.719339
40	C	2.929745	9.911378	-0.976717
41	H	6.023291	9.018496	-0.170599
42	H	4.943831	6.802534	0.056111
43	C	-1.204069	-0.113711	0.145824
44	C	-2.03176	-0.179695	1.272741
45	C	-1.758766	-0.164218	-1.135868
46	C	-3.410505	-0.293807	1.119574
47	C	-3.992243	-0.344313	-0.164076
48	C	-3.139876	-0.279541	-1.28457
49	H	-1.114394	-0.114064	-2.008722
50	H	-3.567162	-0.318957	-2.283039
51	C	4.559605	-9.290955	-1.014546
52	H	2.657651	-8.360566	-1.152665
53	H	4.245468	-10.27784	-1.330455
54	C	2.264431	8.711969	-0.871184
55	H	1.201001	8.685051	-1.08157
56	H	2.449022	10.839139	-1.260422
57	H	-4.032302	-0.34548	2.007707
58	H	-1.589897	-0.141827	2.264176
59	C	-5.434131	-0.460806	-0.393511
60	C	-6.401794	-0.503213	0.548107
61	H	-5.736643	-0.514713	-1.437228
62	C	-7.822777	-0.619825	0.246715
63	H	-6.177111	-0.448481	1.609257
64	N	-8.669456	-0.632877	1.298895
65	N	-9.962863	-0.732824	1.058468
66	C	-10.370953	-0.816486	-0.218985
67	N	-9.522909	-0.810282	-1.272248
68	N	-8.236991	-0.712041	-1.040391
69	C	-11.835763	-0.924771	-0.502521
70	H	-12.402173	-0.920437	0.430751
71	H	-12.050292	-1.849216	-1.050182
72	H	-12.165288	-0.08758	-1.127929
73	C	4.456193	0.367651	1.114631
74	H	4.767737	0.390182	0.064368
75	H	4.965126	-0.463474	1.610261
76	H	4.816884	1.276665	1.603589
77	C	2.584499	0.213408	2.779607
78	H	1.506213	0.121335	2.946604

79	H	2.927925	1.13813	3.2577
80	H	3.080203	-0.636308	3.263226
81	H	4.738796	10.861715	-0.80173
82	H	6.493277	-9.937571	-0.799885

Acri-pvi-H TS1

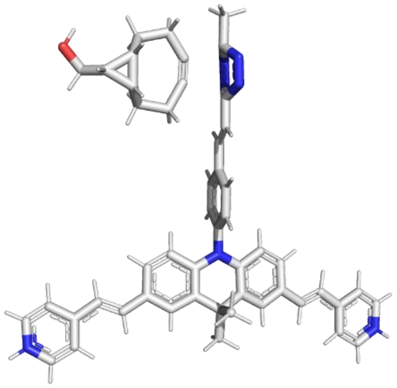

Tag	Symbol	X	Y	Z
1	C	-3.6039	2.1295	0.6075
2	C	-4.7266	1.3017	1.2419
3	C	-4.7075	-0.1018	0.6271
4	C	-2.3876	1.492	0.2599
5	N	-2.3003	0.0932	0.2932
6	C	-3.4645	-0.6878	0.2832
7	C	-5.8527	-0.8841	0.5037
8	C	-5.8248	-2.2373	0.1006
9	C	-3.4106	-2.0507	-0.0857
10	C	-4.5642	-2.8092	-0.172
11	H	-6.8179	-0.4525	0.7452
12	H	-2.4587	-2.5102	-0.3212
13	H	-4.4786	-3.8499	-0.468
14	C	-3.6767	3.5131	0.4692
15	C	-2.588	4.3061	0.0451
16	C	-1.3734	3.6463	-0.2382
17	C	-1.2766	2.2702	-0.136
18	H	-4.6014	4.0228	0.7165
19	H	-0.4979	4.2055	-0.5527
20	H	-0.3377	1.7881	-0.3784
21	C	-2.7733	5.7403	-0.0556
22	C	-1.8477	6.6645	-0.4367
23	H	-3.7731	6.0807	0.2082
24	C	-2.0728	8.0899	-0.5211
25	H	-0.8451	6.3419	-0.7052
26	C	-7.0792	-2.9578	0.0046
27	C	-7.255	-4.2597	-0.3567
28	H	-7.9558	-2.3624	0.2536
29	C	-8.5278	-4.9391	-0.4462
30	H	-6.391	-4.871	-0.6039
31	C	-3.3033	8.7281	-0.2096
32	C	-3.4277	10.0929	-0.3136
33	N	-2.3737	10.8461	-0.7162
34	C	-1.0082	8.9311	-0.9385
35	H	-4.168	8.1636	0.1159
36	H	-4.3422	10.6263	-0.0867
37	C	-9.7841	-4.3273	-0.1898
38	C	-10.9482	-5.0496	-0.2999
39	N	-10.9089	-6.3583	-0.6551

40	C	-9.741	-6.9946	-0.9128
41	H	-11.9287	-4.6291	-0.1163
42	H	-9.8596	-3.2854	0.0947
43	C	-1.0185	-0.5418	0.0993
44	C	-0.2567	-0.8945	1.2191
45	C	-0.5371	-0.8058	-1.1856
46	C	0.9816	-1.5093	1.0553
47	C	1.4874	-1.7866	-0.2314
48	C	0.7031	-1.4227	-1.3442
49	H	-1.1292	-0.5317	-2.0541
50	H	1.0718	-1.6272	-2.3461
51	C	-1.177	10.2934	-1.0287
52	H	-0.0431	8.5069	-1.1925
53	H	-0.3953	10.9739	-1.3415
54	C	-8.552	-6.3097	-0.8153
55	H	-7.6278	-6.8368	-1.025
56	H	-9.8089	-8.039	-1.1897
57	H	1.5532	-1.775	1.9391
58	H	-0.6396	-0.6859	2.214
59	C	2.7816	-2.4349	-0.4706
60	C	3.6599	-2.8533	0.4636
61	H	3.0351	-2.5912	-1.5174
62	C	4.9291	-3.5136	0.1617
63	H	3.4716	-2.722	1.526
64	N	5.5395	-4.1618	1.2213
65	N	6.6802	-4.7071	0.9934
66	C	7.202	-4.5415	-0.2831
67	N	6.2888	-4.5572	-1.3299
68	N	5.143	-4.022	-1.1065
69	C	8.5064	-5.2427	-0.5507
70	H	9.2368	-5.0371	0.2338
71	H	8.9166	-4.9562	-1.521
72	H	8.325	-6.324	-0.5673
73	C	-6.1022	1.9808	1.1045
74	H	-6.3887	2.1145	0.0554
75	H	-6.1037	2.9593	1.5924
76	H	-6.8774	1.3937	1.6043
77	C	-4.4066	1.1563	2.7618
78	H	-3.4346	0.6787	2.9241
79	H	-5.1745	0.5457	3.2509
80	H	-4.3874	2.1434	3.2382
81	C	6.6558	0.6601	0.0535
82	C	5.7517	-0.5907	0.2043
83	C	7.7948	0.69	1.0596
84	C	6.5392	-1.8194	-0.0017
85	C	9.1347	0.01	0.8354
86	C	7.6086	-2.4308	-0.1975

87	C	9.4824	-0.7706	-0.4219
88	C	9.0808	-2.2637	-0.3552
89	C	9.0473	1.5314	0.8772
90	C	9.2804	2.4097	-0.3319
91	O	10.6704	2.75	-0.4949
92	H	6.0176	1.5426	0.1975
93	H	7.0198	0.7041	-0.9775
94	H	4.9279	-0.5321	-0.5158
95	H	5.2913	-0.6015	1.2011
96	H	7.4346	0.6488	2.0881
97	H	9.5709	-0.4333	1.7311
98	H	10.5701	-0.742	-0.5785
99	H	9.0284	-0.3269	-1.3139
100	H	9.5979	-2.7459	0.4843
101	H	9.4306	-2.763	-1.2648
102	H	9.4294	1.9897	1.7891
103	H	8.9011	1.9561	-1.2551
104	H	8.7725	3.3709	-0.2041
105	H	11.1772	1.9258	-0.5807
106	H	-2.485	11.8533	-0.7839
107	H	-11.78	-6.8751	-0.7331

Acri-pvi iEDDA

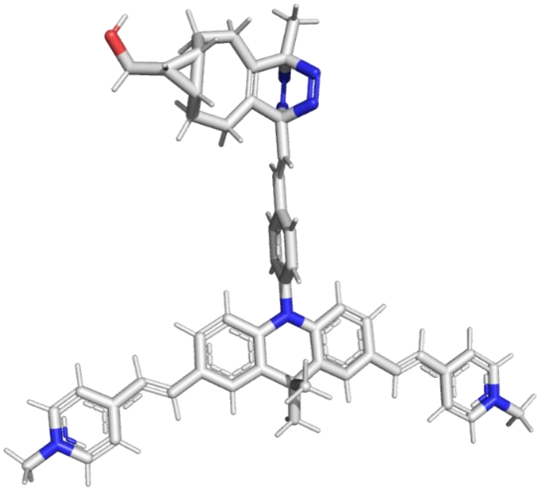

Tag	Symbol	X	Y	Z
1	C	-3.443311	2.087348	0.639518
2	C	-4.484156	1.191652	1.319525
3	C	-4.406471	-0.207598	0.699645
4	C	-2.207056	1.525622	0.23644
5	N	-2.032867	0.134458	0.258477
6	C	-3.146877	-0.715996	0.297941
7	C	-5.506369	-1.058487	0.625368
8	C	-5.414447	-2.407627	0.219536
9	C	-3.02746	-2.072727	-0.078117
10	C	-4.13536	-2.90094	-0.112649
11	H	-6.484385	-0.686646	0.910755
12	H	-2.061173	-2.472594	-0.359235
13	H	-4.000327	-3.934491	-0.415556
14	C	-3.606935	3.464347	0.509986
15	C	-2.588889	4.323592	0.042347
16	C	-1.349549	3.73986	-0.29423
17	C	-1.164135	2.37182	-0.202428
18	H	-4.550103	3.915271	0.798788
19	H	-0.523881	4.351667	-0.643724
20	H	-0.208619	1.948882	-0.486651
21	C	-2.867494	5.744448	-0.050047
22	C	-2.017384	6.723396	-0.46592

23	H	-3.875842	6.022414	0.251874
24	C	-2.336118	8.131883	-0.549509
25	H	-1.005914	6.465102	-0.768594
26	C	-6.626023	-3.204604	0.183059
27	C	-6.736912	-4.519822	-0.151628
28	H	-7.526373	-2.659703	0.461561
29	C	-7.970151	-5.275536	-0.178732
30	H	-5.848012	-5.084096	-0.421962
31	C	-3.59308	8.696726	-0.211631
32	C	-3.806485	10.049584	-0.323755
33	N	-2.829429	10.88992	-0.760306
34	C	-1.34863	9.041294	-0.998831
35	H	-4.415401	8.085409	0.139292
36	H	-4.754741	10.50999	-0.073253
37	C	-3.104803	12.338924	-0.848557
38	H	-3.994411	12.499551	-1.460075
39	H	-2.250904	12.833167	-1.308972
40	H	-3.264247	12.738362	0.155194
41	C	-9.252493	-4.735991	0.099871
42	C	-10.373157	-5.529201	0.047649
43	N	-10.295694	-6.850067	-0.269785
44	C	-9.088514	-7.404673	-0.542256
45	H	-11.365419	-5.145863	0.25264
46	H	-9.386861	-3.692348	0.356075
47	C	-11.53284	-7.656694	-0.319925
48	H	-12.1998	-7.246681	-1.081052
49	H	-12.020207	-7.631039	0.656582
50	H	-11.274836	-8.683515	-0.574044
51	C	-0.726271	-0.419703	-0.006536
52	C	0.112783	-0.734211	1.067901
53	C	-0.294893	-0.641335	-1.316701
54	C	1.378391	-1.266719	0.833325
55	C	1.835224	-1.497817	-0.479418
56	C	0.973335	-1.175039	-1.545224
57	H	-0.946725	-0.397793	-2.150918
58	H	1.302453	-1.344361	-2.56738
59	C	-1.613632	10.389697	-1.093548
60	H	-0.361879	8.68818	-1.278558
61	H	-0.874541	11.104027	-1.434145
62	C	-7.934891	-6.652523	-0.505095
63	H	-6.992768	-7.140319	-0.730633
64	H	-9.086005	-8.459339	-0.787602
65	H	2.010169	-1.502144	1.684211
66	H	-0.230047	-0.559417	2.083786
67	C	3.162368	-2.053317	-0.794912
68	C	4.111268	-2.408063	0.085635
69	H	3.368044	-2.176045	-1.855667

70	C	5.445702	-2.983324	-0.28134
71	H	3.949348	-2.311151	1.156517
72	N	5.684802	-4.213085	0.595059
73	N	6.695432	-4.843335	0.29545
74	C	7.477635	-4.250683	-0.865003
75	N	6.464149	-4.154679	-1.976267
76	N	5.447428	-3.520773	-1.684156
77	C	8.563525	-5.226992	-1.271266
78	H	9.285301	-5.379746	-0.465379
79	H	9.092772	-4.883838	-2.163578
80	H	8.098394	-6.19062	-1.500846
81	C	-5.90354	1.785112	1.245269
82	H	-6.24375	1.901722	0.210227
83	H	-5.943307	2.761391	1.735916
84	H	-6.618618	1.151156	1.776676
85	C	-4.089337	1.065515	2.823517
86	H	-3.084154	0.647229	2.941405
87	H	-4.796791	0.409909	3.344425
88	H	-4.10838	2.051969	3.301311
89	C	7.443046	0.388848	0.496256
90	C	6.381042	-0.706532	0.347568
91	C	8.471562	0.089303	1.568754
92	C	6.714022	-2.108267	-0.133002
93	C	9.719103	-0.687153	1.221134
94	C	7.825052	-2.805381	-0.454626
95	C	9.932958	-1.162556	-0.201723
96	C	9.321175	-2.539768	-0.492224
97	C	9.825199	0.776791	1.629063
98	C	10.243295	1.884559	0.689304
99	O	11.673862	2.053294	0.652377
100	H	6.900497	1.305873	0.764541
101	H	7.905174	0.591761	-0.473398
102	H	5.603626	-0.319781	-0.323568
103	H	5.878327	-0.814533	1.320853
104	H	8.024258	-0.122742	2.54022
105	H	10.071373	-1.397541	1.969232
106	H	11.01056	-1.2706	-0.389813
107	H	9.576039	-0.434721	-0.935623
108	H	9.788022	-3.257457	0.198243
109	H	9.673305	-2.838155	-1.487846
110	H	10.203712	0.937794	2.638324
111	H	9.864542	1.73238	-0.328068
112	H	9.85965	2.846803	1.043581
113	H	12.071105	1.207578	0.386928

Acri-pvi-H iEDDA

Tag	Symbol	X	Y	Z
1	C	-3.865574	1.968549	0.620657
2	C	-4.848196	1.014943	1.308613
3	C	-4.683621	-0.38094	0.698096
4	C	-2.595438	1.482086	0.224042
5	N	-2.335587	0.105102	0.255016
6	C	-3.394874	-0.81198	0.297575
7	C	-5.727904	-1.299407	0.630256
8	C	-5.552587	-2.640698	0.224839
9	C	-3.190919	-2.160107	-0.073567
10	C	-4.245258	-3.054852	-0.10685
11	H	-6.726628	-0.989269	0.917671
12	H	-2.201153	-2.5006	-0.351022
13	H	-4.045958	-4.07971	-0.403591
14	C	-4.116122	3.330467	0.474969
15	C	-3.153335	4.246868	-0.002432
16	C	-1.877283	3.739796	-0.326961
17	C	-1.605556	2.387929	-0.218971
18	H	-5.086821	3.723876	0.756592
19	H	-1.09081	4.399094	-0.680084
20	H	-0.624026	2.023329	-0.49506
21	C	-3.52212	5.643812	-0.120551
22	C	-2.734525	6.666957	-0.55579
23	H	-4.546884	5.86252	0.174221
24	C	-3.142277	8.048585	-0.671691
25	H	-1.708499	6.46622	-0.852545
26	C	-6.710723	-3.511668	0.188511
27	C	-6.744201	-4.817692	-0.198099
28	H	-7.637803	-3.036616	0.504233
29	C	-7.922413	-5.654358	-0.221994
30	H	-5.829589	-5.303839	-0.527354
31	C	-4.437526	8.53374	-0.347182
32	C	-4.740963	9.866225	-0.493265
33	N	-3.803721	10.734505	-0.949904
34	C	-2.20486	9.005278	-1.142602
35	H	-5.214425	7.873949	0.018102
36	H	-5.712313	10.284075	-0.260883
37	C	-9.215913	-5.231422	0.186146
38	C	-10.283421	-6.095194	0.12821
39	N	-10.11149	-7.36405	-0.319909
40	C	-8.901656	-7.824094	-0.72015
41	H	-11.287442	-5.821775	0.42735
42	H	-9.394278	-4.228028	0.552134
43	C	-0.996648	-0.369026	-0.005748
44	C	-0.141963	-0.626889	1.071355

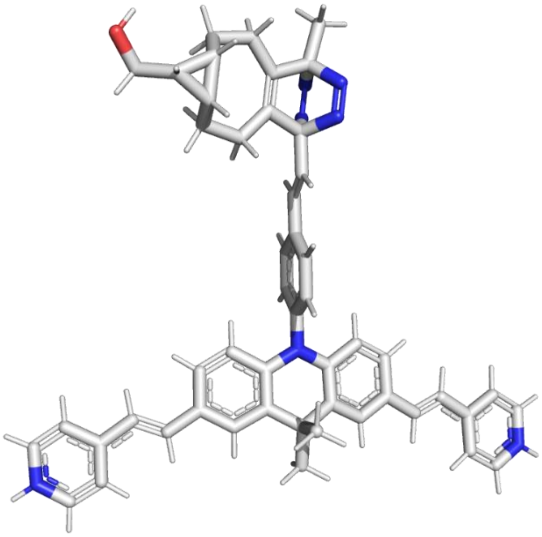

45	C	-0.550558	-0.569419	-1.314346
46	C	1.153789	-1.083317	0.840876
47	C	1.625607	-1.293102	-0.470135
48	C	0.747826	-1.026857	-1.53856
49	H	-1.214245	-0.369005	-2.150663
50	H	1.088073	-1.180913	-2.559424
51	C	-2.55171	10.329925	-1.273371
52	H	-1.19806	8.701358	-1.407316
53	H	-1.871717	11.093805	-1.628721
54	C	-7.805891	-6.993647	-0.678309
55	H	-6.845981	-7.379918	-1.002701
56	H	-8.862379	-8.850685	-1.061869
57	H	1.797211	-1.276144	1.693716
58	H	-0.496353	-0.468431	2.085928
59	C	2.982975	-1.772729	-0.781534
60	C	3.947645	-2.074243	0.101696
61	H	3.197683	-1.885288	-1.841681
62	C	5.311679	-2.578094	-0.261573
63	H	3.77849	-1.985453	1.172146
64	N	5.616586	-3.786605	0.623742
65	N	6.659549	-4.363943	0.328342
66	C	7.40888	-3.738623	-0.83656
67	N	6.391823	-3.705598	-1.948235
68	N	5.342494	-3.125081	-1.660628
69	C	8.545987	-4.657874	-1.235818
70	H	9.275399	-4.764537	-0.429359
71	H	9.055419	-4.293574	-2.13131
72	H	8.133785	-5.647183	-1.457155
73	C	-6.301748	1.518264	1.231084
74	H	-6.64967	1.603492	0.195567
75	H	-6.401481	2.49489	1.712501
76	H	-6.975329	0.846247	1.769814
77	C	-4.445514	0.924348	2.812989
78	H	-3.416202	0.570293	2.932914
79	H	-5.11059	0.229573	3.338907
80	H	-4.52601	1.910961	3.283925
81	C	7.123973	0.902508	0.491646
82	C	6.122406	-0.249261	0.349002
83	C	8.166248	0.665824	1.566528
84	C	6.530922	-1.634882	-0.120242
85	C	9.453747	-0.045249	1.224947
86	C	7.677875	-2.273774	-0.436671
87	C	9.693958	-0.518805	-0.19436
88	C	9.157637	-1.92895	-0.474858
89	C	9.481238	1.425138	1.622022
90	C	9.84132	2.545495	0.673291
91	O	11.261883	2.783147	0.629614

92	H	6.532686	1.790789	0.753585
93	H	7.575633	1.123784	-0.478923
94	H	5.32736	0.090435	-0.326956
95	H	5.623186	-0.376807	1.321734
96	H	7.730431	0.437253	2.539454
97	H	9.843083	-0.730219	1.978365
98	H	10.776039	-0.569651	-0.381075
99	H	9.298672	0.183183	-0.933754
100	H	9.660758	-2.615371	0.221876
101	H	9.526876	-2.215911	-1.467601
102	H	9.850312	1.613657	2.630001
103	H	9.466777	2.368182	-0.341565
104	H	9.412628	3.490386	1.02258
105	H	11.698532	1.955285	0.369785
106	H	-4.048211	11.715524	-1.052142
107	H	-10.913329	-7.986229	-0.357196

Acri-pvi-H TS2

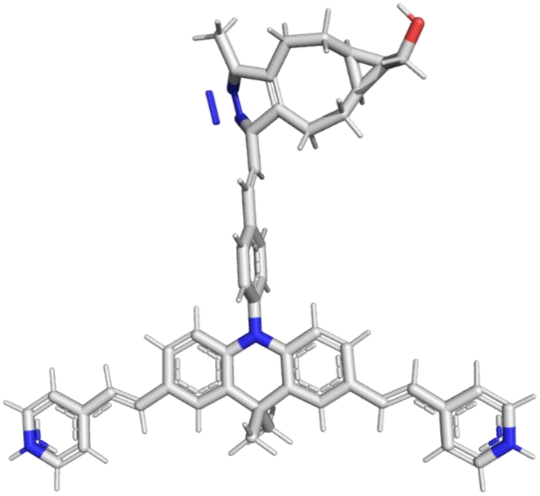

Tag	Symbol	X	Y	Z
1	C	-3.880339	2.168667	-0.008684
2	C	-5.153548	1.314199	0.015608
3	C	-4.814175	-0.180886	-0.021209
4	C	-2.583847	1.607181	-0.062907
5	N	-2.401599	0.220968	-0.095447
6	C	-3.485832	-0.662263	-0.072377
7	C	-5.845273	-1.119097	-0.004715
8	C	-5.635721	-2.51285	-0.033221
9	C	-3.252882	-2.059421	-0.100884
10	C	-4.298259	-2.962054	-0.081545
11	H	-6.871842	-0.76586	0.032312
12	H	-2.237751	-2.433667	-0.139242
13	H	-4.06824	-4.022539	-0.106067
14	C	-3.985833	3.558468	0.021837
15	C	-2.87669	4.428503	0.001337
16	C	-1.595785	3.837325	-0.052972
17	C	-1.455433	2.46354	-0.084384
18	H	-4.974719	4.006176	0.063366
19	H	-0.700149	4.450177	-0.070768
20	H	-0.460425	2.038993	-0.126165
21	C	-3.111309	5.857987	0.035711
22	C	-2.172817	6.846014	0.020311
23	H	-4.162743	6.136694	0.076509
24	C	-2.448631	8.264265	0.055047
25	H	-1.118722	6.584716	-0.02231
26	C	-6.787036	-3.392366	-0.012966
27	C	-6.780408	-4.754986	-0.02218

28	H	-7.744577	-2.875412	0.012845
29	C	-7.954778	-5.597139	-0.004743
30	H	-5.833083	-5.28738	-0.043198
31	C	-3.751734	8.827351	0.117305
32	C	-3.922608	10.190685	0.145657
33	N	-2.845339	11.014597	0.114793
34	C	-1.362091	9.17808	0.025977
35	H	-4.637311	8.205057	0.143957
36	H	-4.893064	10.668473	0.192662
37	C	-9.289653	-5.110723	0.016587
38	C	-10.350541	-5.984095	0.030957
39	N	-10.13256	-7.323017	0.02513
40	C	-8.882707	-7.845701	0.005066
41	H	-11.385151	-5.665093	0.046914
42	H	-9.506253	-4.049793	0.02102
43	C	-1.060782	-0.311909	-0.157075
44	C	-0.362299	-0.583574	1.025093
45	C	-0.462751	-0.556655	-1.39434
46	C	0.930523	-1.09717	0.968405
47	C	1.555296	-1.352555	-0.269273
48	C	0.832378	-1.073212	-1.444752
49	H	-1.00792	-0.345404	-2.309955
50	H	1.291768	-1.263003	-2.411547
51	C	-1.579274	10.5361	0.05583
52	H	-0.341748	8.814013	-0.021155
53	H	-0.784008	11.270642	0.034518
54	C	-7.791812	-7.007748	-0.01011
55	H	-6.799214	-7.443984	-0.026308
56	H	-8.809193	-8.925934	0.001751
57	H	1.451553	-1.301066	1.898715
58	H	-0.834602	-0.391813	1.984501
59	C	2.918658	-1.893346	-0.394939
60	C	3.75928	-2.194025	0.608355
61	H	3.250335	-2.055924	-1.41899
62	C	5.126386	-2.766854	0.432642
63	H	3.464998	-2.068442	1.648214
64	N	5.418978	-3.665532	1.519761
65	N	6.471087	-4.346378	1.393866
66	C	7.222205	-4.116367	0.185703
67	N	6.06853	-4.498515	-0.996805
68	N	5.070362	-3.86869	-0.90004
69	C	8.321411	-5.143713	0.034668
70	H	9.099021	-5.002071	0.790974
71	H	8.781082	-5.101411	-0.955292
72	H	7.892036	-6.140198	0.174032
73	C	-6.025734	1.667386	-1.220772
74	H	-5.485694	1.455447	-2.149969

75	H	-6.299365	2.727488	-1.217844
76	H	-6.954037	1.086915	-1.224253
77	C	-5.947186	1.622509	1.315261
78	H	-5.349991	1.381489	2.201386
79	H	-6.872591	1.039088	1.357167
80	H	-6.221858	2.681096	1.365078
81	C	6.988316	0.679777	-0.238617
82	C	5.953622	-0.431949	-0.028496
83	C	8.046922	0.738321	0.844549
84	C	6.324275	-1.905833	0.088724
85	C	9.311339	-0.073477	0.689849
86	C	7.462647	-2.64101	-0.041827
87	C	9.486604	-0.932109	-0.546231
88	C	8.916377	-2.349194	-0.3954
89	C	9.377521	1.447855	0.65541
90	C	9.733207	2.247521	-0.577125
91	O	11.156829	2.419973	-0.719554
92	H	6.422255	1.621614	-0.246023
93	H	7.42834	0.595776	-1.235033
94	H	5.218152	-0.348102	-0.838736
95	H	5.387947	-0.183942	0.880765
96	H	7.630793	0.804767	1.850083
97	H	9.710047	-0.530784	1.595531
98	H	10.557779	-1.065029	-0.754411
99	H	9.071878	-0.453398	-1.436648
100	H	9.528042	-2.854933	0.363171
101	H	9.131392	-2.88357	-1.331625
102	H	9.778014	1.90162	1.561798
103	H	9.323268	1.805874	-1.492834
104	H	9.337202	3.264957	-0.495792
105	H	11.565646	1.539382	-0.751402
106	H	-10.930266	-7.951206	0.035515
107	H	-2.993057	12.018952	0.135543

Clicked Acri-pvi

Tag	Symbol	X	Y	Z
1	C	-3.021576	2.273765	0.018236
2	C	-4.31698	1.482382	-0.206154
3	C	-4.072275	-0.025824	-0.063977
4	C	-1.784182	1.647057	0.302777
5	N	-1.685225	0.25797	0.395663
6	C	-2.79591	-0.5684	0.221873
7	C	-5.132652	-0.914392	-0.219433
8	C	-5.004805	-2.316404	-0.107097
9	C	-2.643375	-1.973836	0.334678
10	C	-3.716097	-2.824274	0.175713
11	H	-6.11913	-0.51478	-0.438686
12	H	-1.667392	-2.388235	0.552083
13	H	-3.548346	-3.892042	0.273621
14	C	-3.040416	3.663921	-0.056944
15	C	-1.90098	4.475759	0.135371
16	C	-0.682209	3.817364	0.417333
17	C	-0.62667	2.44272	0.498033
18	H	-3.981943	4.16097	-0.274548
19	H	0.231059	4.381749	0.576161
20	H	0.318948	1.963837	0.716767
21	C	-2.03664	5.908798	0.039028
22	C	-1.052978	6.845917	0.198916
23	H	-3.046382	6.250846	-0.18555
24	C	-1.22261	8.270099	0.099942
25	H	-0.041014	6.518783	0.423151
26	C	-6.174642	-3.142434	-0.27967
27	C	-6.245053	-4.506363	-0.199543
28	H	-7.08804	-2.588679	-0.49424
29	C	-7.431798	-5.299162	-0.371516
30	H	-5.341834	-5.071968	0.014195
31	C	-2.451256	8.929405	-0.183551
32	C	-2.516611	10.298696	-0.260582
33	N	-1.41615	11.080497	-0.072751
34	C	-0.102422	9.124271	0.29112
35	H	-3.365771	8.372303	-0.348955
36	H	-3.441334	10.822496	-0.474532
37	C	-1.536098	12.553003	-0.108861
38	H	-2.27937	12.835399	-0.855955
39	H	-0.573111	12.984189	-0.384558
40	H	-1.838733	12.925462	0.874089
41	C	-8.726398	-4.781836	-0.65658
42	C	-9.804486	-5.618828	-0.805557
43	N	-9.683126	-6.971464	-0.690441
44	C	-8.462728	-7.508024	-0.415972

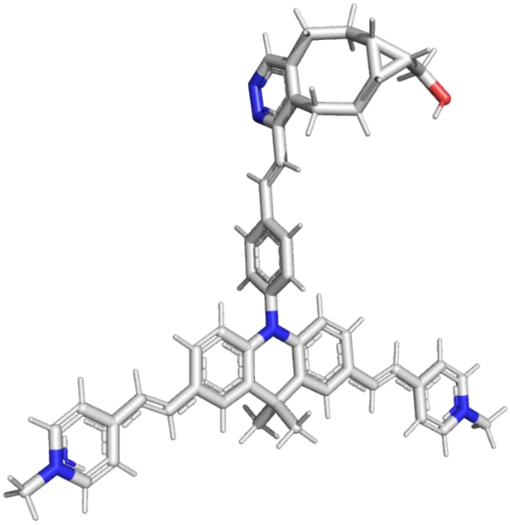

45	H	-10.799638	-5.247235	-1.021987
46	H	-8.897325	-3.717551	-0.76663
47	C	-10.876029	-7.836559	-0.801916
48	H	-11.558585	-7.415028	-1.541358
49	H	-11.377144	-7.905472	0.167956
50	H	-10.568419	-8.830744	-1.127905
51	C	-0.221157	10.490422	0.203436
52	H	0.874309	8.704618	0.508777
53	H	0.621476	11.157027	0.347048
54	C	-7.352438	-6.714093	-0.257968
55	H	-6.402315	-7.192998	-0.044893
56	H	-8.421247	-8.587936	-0.331776
57	C	-4.848754	1.776254	-1.636014
58	H	-4.121436	1.471533	-2.395805
59	H	-5.051842	2.843995	-1.768253
60	H	-5.783968	1.239413	-1.825612
61	C	-5.371575	1.926925	0.844348
62	H	-5.019368	1.729196	1.862133
63	H	-6.317723	1.394861	0.702186
64	H	-5.584566	2.997431	0.759846
65	C	-0.400192	-0.338151	0.701398
66	C	-0.034388	-0.56042	2.030397
67	C	0.473548	-0.686405	-0.334954
68	C	1.206501	-1.126335	2.317403
69	H	-0.715291	-0.290724	2.833546
70	C	1.711701	-1.249913	-0.039609
71	H	0.181099	-0.514897	-1.367767
72	C	2.11205	-1.479573	1.294743
73	H	1.488049	-1.295695	3.353366
74	H	2.371993	-1.515572	-0.859474
75	C	3.403198	-2.054091	1.6796
76	C	4.424955	-2.379686	0.859441
77	H	3.551374	-2.216807	2.744355
78	C	5.710956	-2.920077	1.33947
79	H	4.329256	-2.220571	-0.209785
80	C	6.764533	-3.292405	0.463001
81	N	5.812313	-3.009186	2.682704
82	C	7.956826	-3.712769	1.073763
83	C	6.63389	-3.225329	-1.0438
84	N	6.919143	-3.448965	3.25066
85	C	9.226945	-4.055693	0.326452
86	C	7.95662	-3.776514	2.47393
87	C	7.277115	-1.947694	-1.678101
88	H	7.111611	-4.110003	-1.479807
89	H	5.581696	-3.29491	-1.329611
90	C	9.965229	-2.805757	-0.255066
91	H	9.893049	-4.57842	1.020893

92	H	9.017554	-4.76313	-0.485708
93	H	8.838037	-4.116536	3.012905
94	C	8.546974	-2.262206	-2.454926
95	H	6.552265	-1.487426	-2.36401
96	H	7.461045	-1.206851	-0.893105
97	C	9.832586	-2.674423	-1.766094
98	H	11.032732	-2.886063	-0.015455
99	H	9.613722	-1.907623	0.262043
100	C	9.767344	-1.348021	-2.505201
101	H	8.341166	-2.79367	-3.384647
102	H	10.394005	-3.447215	-2.291912
103	C	9.805553	-0.000191	-1.818894
104	H	10.284857	-1.327025	-3.463585
105	O	9.211037	1.026343	-2.621337
106	H	9.327411	-0.026812	-0.830456
107	H	10.841691	0.319701	-1.670368
108	H	8.355023	0.702042	-2.942867

Clicked Acri-pvi-H

Tag	Symbol	X	Y	Z
1	C	4.357552	-1.072934	0.056464
2	C	5.11563	0.249013	0.229072
3	C	4.184678	1.44927	0.017725
4	C	2.978735	-1.128844	-0.251775
5	N	2.231282	0.041211	-0.418529
6	C	2.81148	1.307006	-0.28764
7	C	4.694981	2.741914	0.129899
8	C	3.919241	3.906914	-0.038846
9	C	2.014515	2.464888	-0.464006
10	C	2.551672	3.731508	-0.341998
11	H	5.748321	2.869239	0.363262
12	H	0.963059	2.361845	-0.70026
13	H	1.9	4.587042	-0.487995
14	C	5.038778	-2.279768	0.208327
15	C	4.430775	-3.544485	0.072594
16	C	3.053088	-3.566778	-0.234827
17	C	2.34796	-2.389611	-0.392326
18	H	6.098726	-2.254772	0.444593
19	H	2.524397	-4.507405	-0.352095
20	H	1.292376	-2.438245	-0.627683
21	C	5.241616	-4.73133	0.25479
22	C	4.834675	-6.028536	0.160071
23	H	6.284031	-4.527725	0.492965
24	C	5.678189	-7.186073	0.35258
25	H	3.797278	-6.250508	-0.076158
26	C	4.559347	5.19879	0.106034

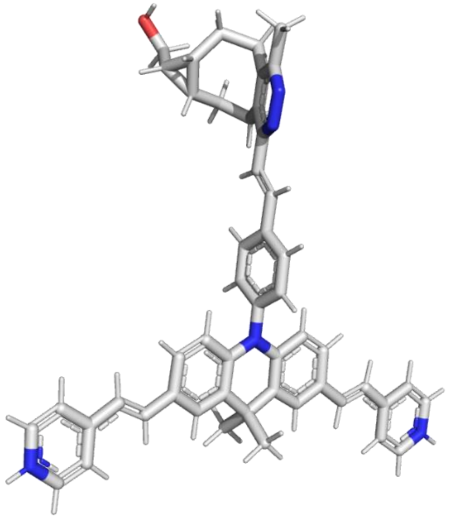

27	C	3.967435	6.423388	0.021353
28	H	5.627193	5.147993	0.310574
29	C	4.641991	7.692511	0.172768
30	H	2.899201	6.491775	-0.167034
31	C	7.063275	-7.126391	0.664045
32	C	7.790418	-8.27953	0.836695
33	N	7.191983	-9.490327	0.709284
34	C	5.109999	-8.481589	0.229253
35	H	7.579301	-6.18078	0.773836
36	H	8.846261	-8.287123	1.075843
37	C	6.039975	7.839368	0.380513
38	C	6.599311	9.08794	0.511496
39	N	5.819363	10.195923	0.444458
40	C	4.480987	10.118149	0.248627
41	H	7.65788	9.251694	0.668201
42	H	6.697852	6.981155	0.435844
43	C	0.826574	-0.060122	-0.737968
44	C	-0.122729	-0.100396	0.290102
45	C	0.415707	-0.115935	-2.071086
46	C	-1.477436	-0.196181	-0.015407
47	C	-1.918003	-0.257446	-1.354553
48	C	-0.942884	-0.213386	-2.371222
49	H	1.154987	-0.083207	-2.866452
50	H	-1.256336	-0.256578	-3.411237
51	C	5.875987	-9.609811	0.410273
52	H	4.058544	-8.598097	-0.009162
53	H	5.486771	-10.616702	0.328415
54	C	3.879558	8.888765	0.110936
55	H	2.807154	8.847128	-0.044558
56	H	3.941951	11.056305	0.209953
57	H	-2.194023	-0.21999	0.799737
58	H	0.204125	-0.05369	1.32519
59	C	-3.329525	-0.362181	-1.74429
60	C	-4.391591	-0.464563	-0.917685
61	H	-3.516474	-0.358584	-2.815935
62	C	-5.791452	-0.53046	-1.379922
63	H	-4.230273	-0.491171	0.154437
64	N	-6.007603	-0.038248	-2.614589
65	N	-7.222764	-0.035054	-3.145745
66	C	-8.272592	-0.489161	-2.44504
67	C	-9.600211	-0.417195	-3.156173
68	H	-10.338414	0.156097	-2.583842
69	H	-10.021801	-1.416515	-3.321862
70	H	-9.465169	0.064003	-4.127856
71	C	6.271058	0.311843	-0.807619
72	H	5.879035	0.268089	-1.829554
73	H	6.965918	-0.523118	-0.671077

74	H	6.844261	1.238345	-0.700153
75	C	5.704721	0.312207	1.66555
76	H	4.906609	0.270809	2.414804
77	H	6.268787	1.238299	1.81697
78	H	6.387817	-0.524106	1.846327
79	C	-7.369228	-1.130859	1.996484
80	C	-6.586578	-1.699799	0.770317
81	C	-7.780519	0.320708	1.83091
82	C	-6.848159	-1.056578	-0.58429
83	C	-9.055792	0.690213	1.096535
84	C	-8.132923	-1.008133	-1.137968
85	C	-10.004889	-0.355161	0.506018
86	C	-9.341562	-1.473958	-0.362216
87	C	-8.923465	0.962544	2.594068
88	C	-9.733627	0.240087	3.644429
89	O	-10.997712	0.885201	3.890404
90	H	-6.725015	-1.233597	2.879145
91	H	-8.244717	-1.754296	2.194765
92	H	-6.811734	-2.771932	0.681442
93	H	-5.52027	-1.654058	0.989216
94	H	-6.936263	0.986106	1.649432
95	H	-8.971426	1.581048	0.476624
96	H	-10.734743	0.180994	-0.111351
97	H	-10.589867	-0.86161	1.281901
98	H	-10.099207	-1.868605	-1.045654
99	H	-9.054149	-2.310795	0.279335
100	H	-8.766086	2.008834	2.854908
101	H	-9.899634	-0.813931	3.391042
102	H	-9.214272	0.267451	4.607735
103	H	-11.475734	0.94801	3.046942
104	H	6.252076	11.109978	0.538984
105	H	7.747115	-10.330244	0.843698

XVI. Click in cell lysate

Preparation of human Lysozyme-BCN

0,6 mL of human lysozyme (300 µM in 100 mM NaH2PO4, 25 mM NaOAc, pH 8,5) was incubated with BCN-NHS ester (60 µL, 10 mM in DMSO, final concentration 1 mM) overnight on a rocker at room temperature. Excess BCN-NHS ester was removed by protein spin column using 0.25 M NH_3H_2O solution as eluent.

For MALDI experiment: An aliquot of the mixture containing BCN-modified lysozyme (Lysozyme-BCN) was subjected to buffer exchange to a 100mM ammonium acetate pH 6.5 using vivaspin 6 centrifugal concentrators (3 kDa, Sartorius) and further characterized by MALDI-TOF/TOF.

A MALDI-TOF/TOF UltrafleXtreme mass spectrometer (Bruker Daltonics, Bremen) was used for all experiments. Mass spectra were obtained in linear positive ion mode. The laser intensity was set just above the ion generation threshold to obtain peaks with the highest possible signal-to-noise (S/N) ratio without significant peak broadening. All data were processed using the FlexAnalysis software package (Bruker Daltonics).

Figure S10. MALDI analysis of human lysozyme and BCN tagged lysozyme

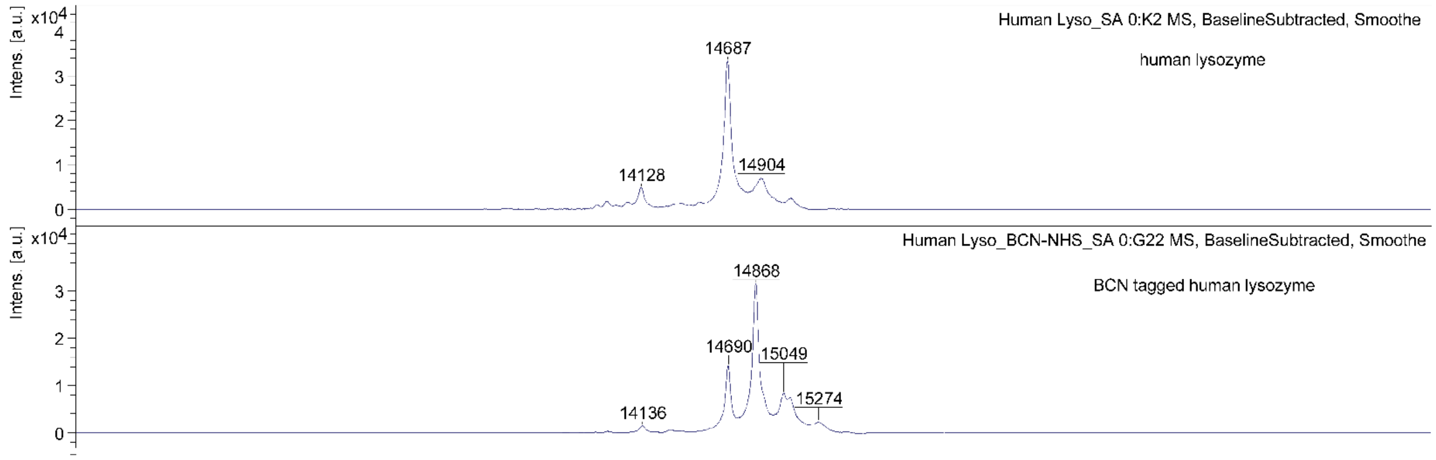

For the click reaction: The mixture containing BCN-modified lysozyme was washed 3 times with PBS (1mL), concentrated in 300 µL of PBS using vivaspin 6 centrifugal concentrators (3 kDa, Sartorius).

A549 lung carcinoma cells were cultured in Dulbecco's modified eagle media (DMEM, GIBCO) supplemented with 10% Fetal Bovine Serum (FBS, GIBCO) and 1% Penicillin/Streptomycin (Life Technologies) at 37 °C under 5% CO_2, 95% air and 100% humidity. A549 cells were washed in PBS and lysed on ice for 20 minutes in RIPA buffer (50 mM Tris pH 7.5, 150 mM NaCl, 5 mM EDTA, 0.1% SDS, 1% NP-40, 0.5% sodium deoxycholate) and centrifuged at 11 000g for 15 minutes at 4°C.

For IEDDA, 1µL of the lysozyme-BCN mixture (0.8 mg/mL), 7 µL (3 mg/mL) of A549 lysate, and 5 µL of **Acri-*ovi*** and 7 µL of RIPA lysis buffer 1x were mixed and stirred at r.t. for 90 minutes.

Then, laemmli buffer 4x was mixed with the sample, heated for 5 min at 95°C and analyzed by SDS-PAGE using 4-20% gradient polyacrylamide gel.

After electrophoric migration, the gel was subjected to fluorescence analysis (typhoon) before Coomassie brilliant blue staining.

Figure S11: Fluorogenic reaction of Acri-*ovi* with BCN-lysozyme in aqueous buffer

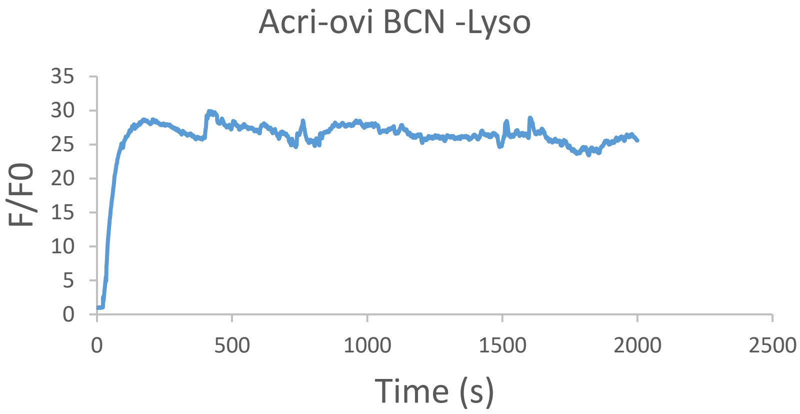

XVII. MTT Assay

A549 lung carcinoma cells were seeded in 24-well plates from TPP (50 000 cells/well). After 24h, cells were incubated with solutions of various concentrations of both probes (for 0.313–10 µM) for 24h. Afterwards, 500 µL of MTT solution (previously sterilized with a 0.22 µm filter, 5 mg/mL in PBS) were added in each well. After 3h, the medium was removed and 500 µL of DMSO were added to dissolve MTT formazan. Optical densities were then measured at 562 nm with a FLUOStar Omega plate reader from BMG Labtech. Every experiment was conducted in duplicate and reproduced three times. Results are represented as means ± SEM.

Figure S12: Cytotoxity assay of all probes on A549 cells

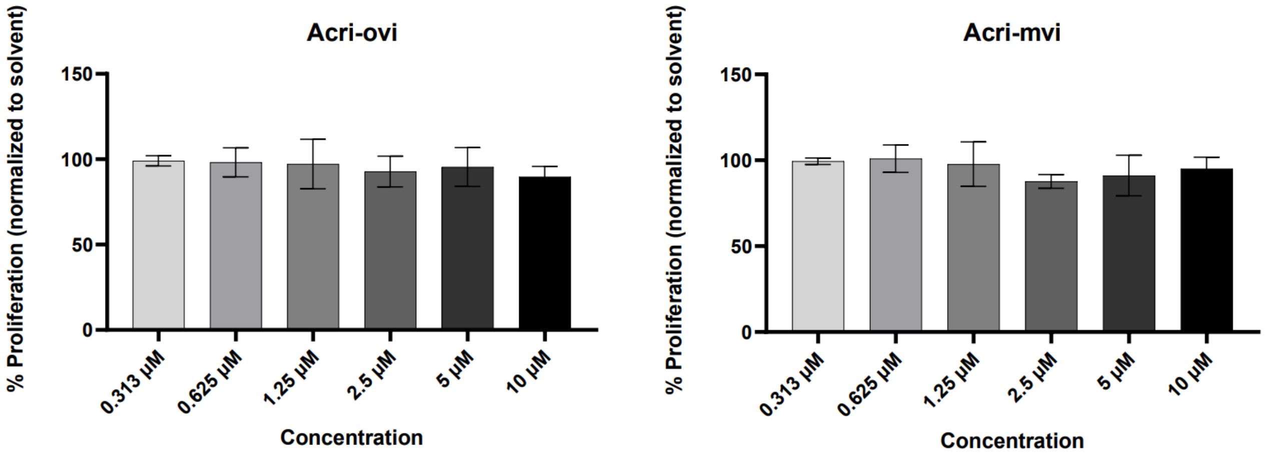

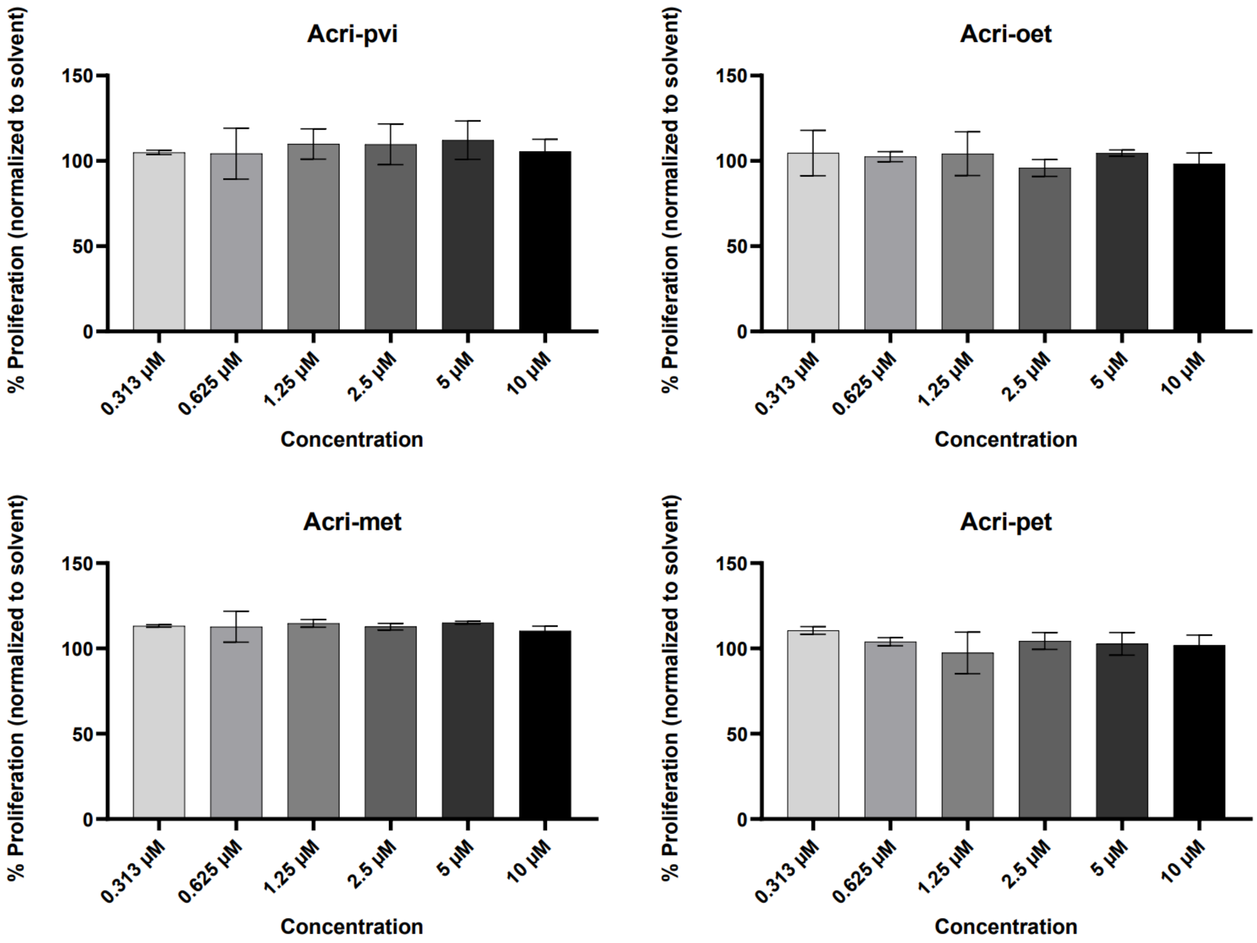

XVIII. Live-cell imaging

Colocalization experiments: A549 lung carcinoma cells were cultured in Dulbecco's modified eagle media (DMEM, GIBCO) supplemented with 10% Fetal Bovine Serum (FBS, GIBCO) and 1% Penicillin/Streptomycin (Life Technologies) at 37 °C under 5% CO_2, 95% air and 100% humidity. Cells were seeded on a 8-well µ-slide from Ibidi (20 000 cells/well). After 24h, cells were incubated 1h with MitoTrackerDeep Red (20 nM). Cell culture media was replaced with DMEM supplemented with 10% Fetal Bovine Serum (FBS, GIBCO) and 1% Penicillin/Streptomycin containing AcriPy (2µM). After 30 min incubation, fluorescence microscopy was performed.

Figure S13: Colocalization experiments with Acri-Py with Mitotracker

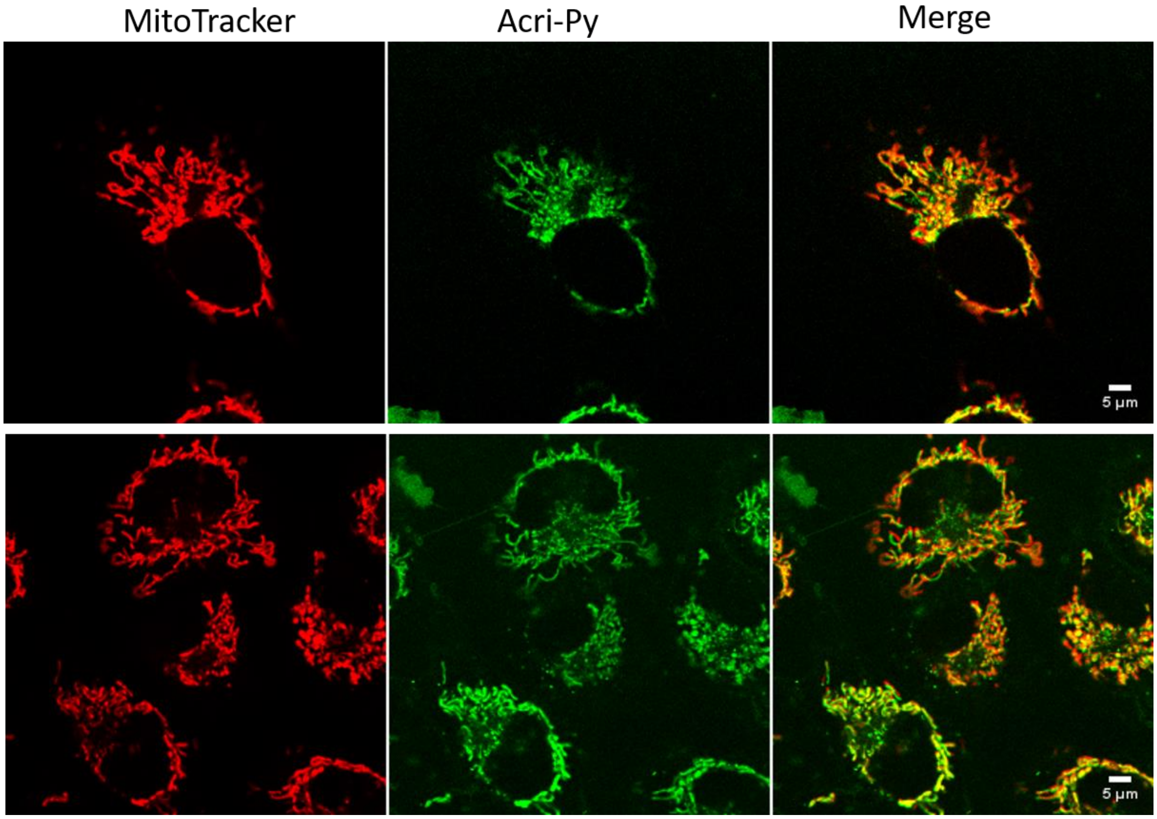

A549 lung carcinoma cells were cultured in Dulbecco's modified eagle media (DMEM, GIBCO) supplemented with 10% Fetal Bovine Serum (FBS, GIBCO) and 1% Penicillin/Streptomycin (Life Technologies) at 37 °C under 5% CO_2, 95% air and 100% humidity. Cells were seeded on a 8-well µ-slide from Ibidi (20 000 cells/well). After 24h, cells were incubated 3h with the fluorogenic compound (2µM). After 3h, cell culture media was replaced with DMEM supplemented with 10% Fetal Bovine Serum (FBS, GIBCO) and 1% Penicillin/Streptomycin containing nothing or an excess of BCN (50 µM). After 30 min incubation, fluorescence microscopy was performed.

The fluorescence imaging (confocal and biphotonic) was performed using a confocal laser scanning microscope DMI 6000 with a SP5-AOBS unit (both Leica) equipped with a 63x (NA = 1.4) objective (oil immersion), an argon gas laser, a 405 nm diode and helium neon gas laser (633 nm) for one-photon excitation and a Chameleon Ti:Saph laser (Coherent) delivering pulses in the 100 to 200 fs range at an 80 MHz repetition rate with a tunability ranging from 705 to 980 nm for two-photon excitation. The excitation laser power was measured after the objective by a thermal head laser power meter (PM100 S302 ThorLabs). The images were visualized and processed using Image J software (Rasband W.S., U.S. National Institutes of Health, Bethesda, Maryland,USA).

Colocalization experiments: A549 lung carcinoma cells were cultured in Dulbecco's modified eagle media (DMEM, GIBCO) supplemented with 10% Fetal Bovine Serum (FBS, GIBCO) and 1% Penicillin/Streptomycin (Life Technologies) at 37 °C under 5% CO_2, 95% air and 100% humidity. Cells were seeded on a 8-well µ-slide from Ibidi (20 000 cells/well). After 24h, cells were incubated 3h with the fluorogenic compound (2µM). After 3h, cell culture media was replaced with DMEM supplemented with 10% Fetal Bovine Serum (FBS, GIBCO) and 1% Penicillin/Streptomycin containing nothing or an excess of BCN (10 µM) and the organelle tracker (20 nM). After 30 min incubation, fluorescence microscopy was performed.

Figure S14: Colocalization experiments with Acri-*ovi* + BCN with Organelle Trackers

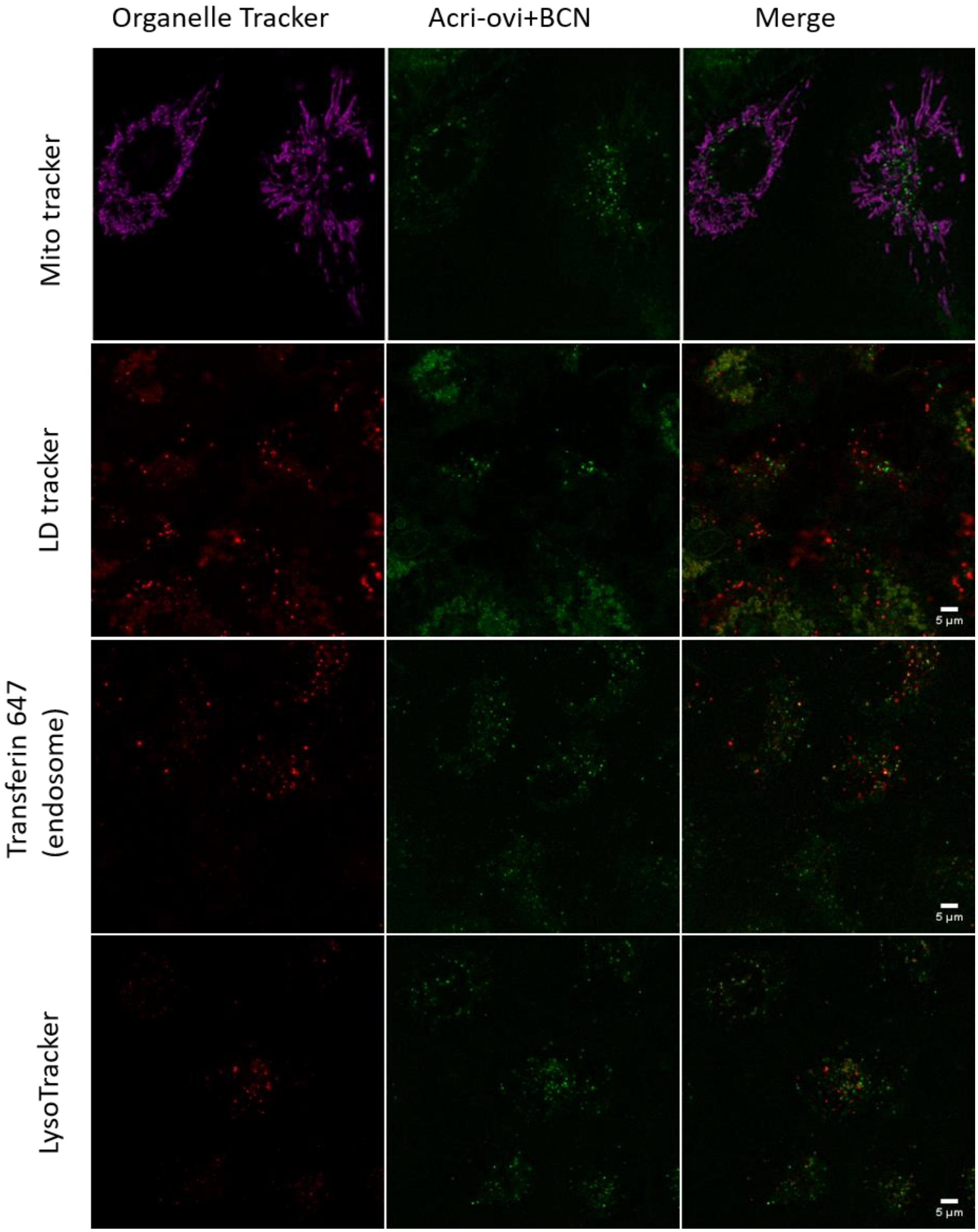

Supplement: SC-014-D3SC01754K-s001 [file SC-014-D3SC01754K-s001.pdf]
